# Synthesis of Indofulvin Pseudo‐Natural Products Yields a New Autophagy Inhibitor Chemotype

**DOI:** 10.1002/advs.202102042

**Published:** 2021-08-04

**Authors:** Annina Burhop, Sukdev Bag, Michael Grigalunas, Sophie Woitalla, Pia Bodenbinder, Lukas Brieger, Carsten Strohmann, Axel Pahl, Sonja Sievers, Herbert Waldmann

**Affiliations:** ^1^ Max Planck Institute of Molecular Physiology Department of Chemical Biology Dortmund 44227 Germany; ^2^ Technical University Dortmund Faculty of Chemistry Chemical Biology Dortmund 44227 Germany; ^3^ Technical University Dortmund Faculty of Chemistry Inorganic Chemistry Dortmund 44227 Germany; ^4^ Compound Management and Screening Center Dortmund 44227 Germany

**Keywords:** autophagy, cell painting, oxa‐Pictet‐Spengler reaction, pseudo‐natural products

## Abstract

Chemical and biological limitations in bioactive compound design based on natural product (NP) structure can be overcome by the combination of NP‐derived fragments in unprecedented arrangements to afford “pseudo‐natural products” (pseudo‐NPs). A new pseudo‐NP design principle is described, i.e., the combination of NP‐fragments by transformations that are not part of current biosynthesis pathways. A collection of indofulvin pseudo‐NPs is obtained from 2‐hydroxyethyl‐indoles and ketones derived from the fragment‐sized NP griseofulvin by means of an iso‐oxa‐Pictet‐Spengler reaction. Cheminformatic analysis indicates that the indofulvins reside in an area of chemical space sparsely covered by NPs, drugs, and drug‐like compounds and they may combine favorable properties of these compound classes. Biological evaluation of the compound collection in different cell‐based assays and the unbiased high content cell painting assay reveal that the indofulvins define a new autophagy inhibitor chemotype that targets mitochondrial respiration.

## Graphical abstract

Indofulvin pseudo‐natural products are obtained by combination of indole‐ and griseofulvin fragments by an iso‐oxa‐Pictet‐Spengler reaction and define a novel autophagy inhibitor chemotype.

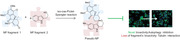

## 1 Introduction

For the design and discovery of small bioactive molecules for chemical biology and medicinal chemistry research, biological relevance is a key requirement. Such relevance for instance is assured if inspiration is drawn from natural product (NP) structure since NPs define the fraction of biologically relevant chemical space explored by nature through evolution.^[^ ^1^ ^]^ This reasoning underlies the rationale of the biology‐oriented synthesis (BIOS)^[^ ^2^ ^]^ and the complexity‐to‐diversity (CtD)^[^ ^3^ ^]^ approaches. We have recently introduced the pseudo‐natural product (pseudo‐NP) concept in which biosynthetically unrelated NP fragments are combined through *de novo* syntheses to afford novel scaffolds with arrangements of NP fragments not found in nature.^[^ ^4^, ^5^, ^6^ ^]^ Pseudo‐NPs therefore resemble NPs but explore chemical space not covered by nature. Furthermore, pseudo‐NPs retain biological relevance yet may result in unexpected and/or novel bioactivities.^[^ ^7^, ^8^, ^9^, ^10^, ^11^ ^]^

We reasoned that novel pseudo‐NPs could be obtained if NP‐fragments were combined in transformations that are not part of the biosynthetic repertoire. For instance, the Pictet‐Spengler (PS) reaction^[^ ^12^ ^]^ is a key transformation in polycyclic‐alkaloid biosynthesis from indole derivatives and carbonyl compounds. However, the analogous oxa‐PS reaction and, in particular, the isomeric iso‐oxa‐Pictet‐Spengler reaction could enable the synthesis of structurally diverse and complex indoles^[^ ^13^, ^14^, ^15^ ^]^ but are not employed by nature in existing biosynthetic pathways.

**Figure 1:**
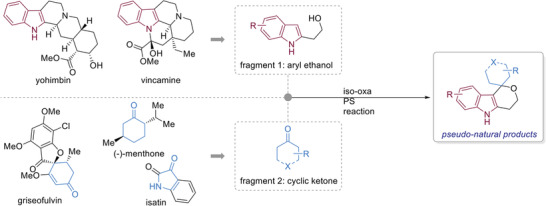
Design of a pseudo‐NP collection combining a natural product‐derived indole fragment and readily available NP‐derived fragments with carbonyl groups.

Here we describe the development of an iso‐oxa‐PS reaction and its application for the synthesis of pseudo‐NPs through the combination of 2‐hydroxyethyl indoles and different ketones (**Figure** **1**). Biological evaluation of the compound collection in different cell‐based assays revealed that indofulvins define a new autophagy inhibitor chemotype that targets mitochondrial respiration.

## 2 Results and Discussion

### 2.1 Reaction Optimization and Substrate Scope

In comparison to the PS reaction,^[^ ^12^ ^]^ the oxa‐PS reaction and, even more so, the iso‐oxa‐PS are underdeveloped for employing complex ketone substrates and frequently require long reaction times with high temperatures and/or give poor yields.^[^ ^13^, ^14^, ^16^, ^17^, ^18^, ^19^, ^20^, ^21^, ^22^ ^]^ For the identification of suitable reaction conditions, 2‐hydroxyethyl indole **1a** and the hindered cyclic ketone (‐)‐menthone **2e** were subjected to cyclization at 22 °C in the presence of different acids, including TsOH, CF_3_COOH, TfOH, and BF_3_·OEt_2_. However, the desired spirocyclic compounds were not formed in viable yields even after extended reaction times (Table S1, Supporting Information). After substantial experimentation, it was found that triflic acid absorbed on silica gel (TfOH⋅SiO_2_)^[^ ^23^ ^]^ is an excellent catalyst for the iso‐oxa PS transformation. In the presence of the TfOH⋅SiO_2_ catalyst (6.5 mol%) in DCM, PS‐adduct **3e** was isolated in 96% yield as a single diastereomer after only 30 min of reaction time at ambient temperature. Furthermore, TfOH⋅SiO_2_ is easy to synthesize, handle, and can be readily removed through filtration which adds to the operational simplicity of the developed procedure.

**Figure 2:**
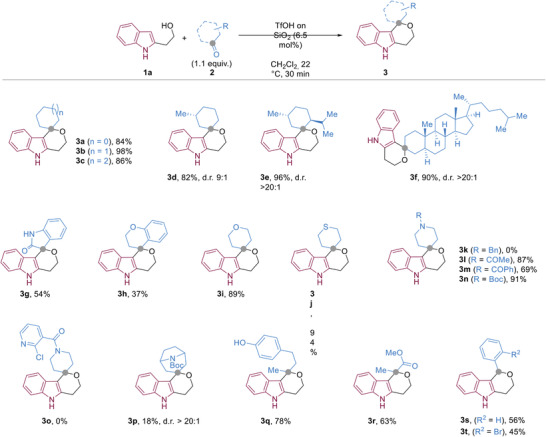
Substrate scope of the iso‐oxa PS reaction showing isolated yields. Diasteriomeric ratios were determined by ^1^H NMR of the crude reaction mixtures. Stereochemical assignment of the diastereomeric products was not possible using 2D‐NMR techniques.

The scope of ketones for the developed reaction was explored by employing **1a** in the presence TfOH⋅SiO_2_ (6.5 mol%) in DCM at 22 °C for 30 min (**Figure** **2**). Aliphatic ketones with various ring sizes were suitable substrates and provided the desired products in high yields (**3a**–**3c**). Reaction with enantioenriched cyclohexyl‐derived ketones afforded spirocyclic products in high yields with good to excellent diastereoselectivities (**3d**–**3f**). Various heterocyclic ketones, including isatin and chromanone, afforded the desired products in moderate to excellent yields (**3g**–**3j**). Substrates containing basic moieties such as pyridines or tertiary amines were not suitable (**3k** and **3o**); however, amide‐ and *N*‐Boc‐containing compounds were tolerated (**3l**–**3n**). The tolerance of an *N*‐Boc functionality highlights the mild nature of the developed conditions relative to other oxa‐PS reactions in which Boc‐protected substrates either fail,^[^ ^17^ ^]^ decompose,^[^ ^24^ ^]^ or are not reported. Employing a challenging tropanone derivative successfully afforded **3p** albeit in a low yield. The reaction conditions were also amenable to an acyclic ketone (**3q**), methyl pyruvate (**3r**), and aromatic aldehydes (**3s** and **3t**).

### 2.2 Synthesis of Indofulvins

**Figure 3:**
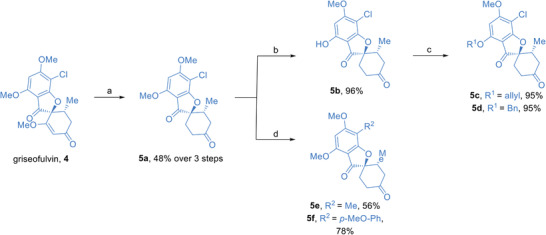
Synthesis of griseofulvin‐derived fragments to be used in iso‐oxa‐PS reactions. a) 1. griseofulvin **4** (1 equiv.), Pd/C (10% w/w), H_2_, EtOAc, 22 °C, 54 h, 2. 2 m H_2_SO_4_, AcOH, 80 °C, 16 h, 3. Pd/C (10% w/w), H_2_, EtOAc, 22 °C, 6 h, b) MgI_2_ (1 equiv.), Et_2_O/toluene, 80 °C, 20 h, c) alkyl bromide (1.05 equiv.), K_2_CO_3_ (2.5 equiv.), DMF, 65 °C, 3 h, d) boronic acid (1 equiv.), Pd(OAc)_2_ (3 mol%), XPhos (6 mol%), K_3_PO_4_ (2 equiv.), THF, 80 °C, 24 h.

We were able to simultaneously synthesize a pseudo‐NP collection and explore the scope of nucleophiles for the developed conditions by reacting ketones **5a–f** derived^[^ ^25^ ^]^ from the NP griseofulvin **4** (**Figure** **3**) with various hydroxyethyl‐derived heterocycles. Griseofulvin is readily isolated from *Penicillium griseofulvum* ^[^ ^26^ ^]^ and is commercially available. The NP itself is fragment‐sized^[^ ^27^, ^28^ ^]^ and has been identified as modulator of tubulin polymerization.^[^ ^29^ ^]^

**Figure 4:**
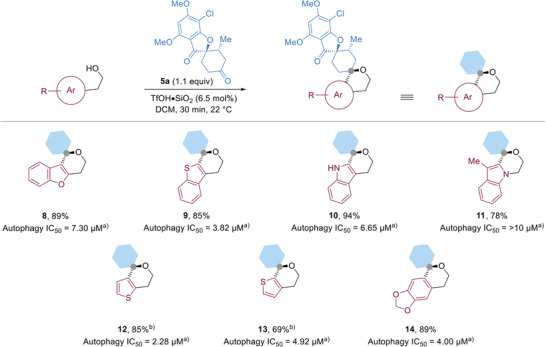
The synthesis pseudo‐NPs by combining various aromatic nucleophiles and **5a** via an iso‐oxa‐PS reaction. The configuration of the compounds was assumed to have the same configuration. All reactions had >20:1 dr as determined by crude ^1^H NMR. a) Half maximum inhibitory concentration in the autophagy assay monitoring formation of LC3‐II punctae after autophagy induction by means of amino acid starvation; data are represented as mean values out of three replicates. b) The reaction was conducted at 50 °C for 2 h.

Griseofulvin‐derived ketone **5a** could be effectively combined with various *α*‐hydroxyethyl indole derivatives (**1**) bearing different substitution patterns (**7a**–**7e**), halogens (**7f**–**7g**), electron donating and electron withdrawing groups (**7h**–**7k**), and *N*‐substitutions (**7l–7m**) in high to excellent isolated yields (**Table** **1**). Other griseofulvin‐derived compounds (**5b**–**5f**) were also suitable substrates and reacted rapidly to afford **7n**–**7s** in moderate to excellent yields. From the combination of the indole‐containing fragments and griseofulvin derivatives via the developed iso‐oxa PS reaction, 19 compounds were synthesized that were termed indofulvins (**7**). Other heterocyclic substrates were also excellent nucleophiles for the developed reaction including benzofuran (**8**), and benzothiophene (**9**) derivatives as well as regioisomeric indole (**10**–**11**), thiophene (**12**–**13**) derivatives (**Figure** **4**). An electron‐rich phenyl derivative was also compatible and provided the desired product in an excellent yield (**14**, Figure 4). All examples employing griseofulvin‐derived ketones resulted in the formation of a single diastereomer (d.r. > 20 :1, Table 1 and Figure 4).

**Table 1:** The synthesis of an indofulvin collection via an iso‐oxa‐PS reaction, and the activity of the resulting pseudo‐NPs in an autophagy inhibition assay

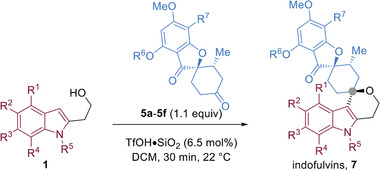										
Entry	Compound	R^1^	R^2^	R^3^	R^4^	R^5^	R^6^	R^7^	Yield [%]^a)^	IC_50_ [× 10^−6^ m]^b)^
1	**7a**	H	H	H	H	H	Me	Cl	92	2.08
2	**7b**	Me	H	H	H	H	Me	Cl	89	3.03
3	**7c**	H	Me	H	H	H	Me	Cl	94	0.82
4	**7d**	H	H	Me	H	H	Me	Cl	85	4.16
5	**7e**	H	H	H	Me	H	Me	Cl	87	1.01
6	**7f**	H	F	H	H	H	Me	Cl	80	1.41
7	**7g**	H	Cl	H	H	H	Me	Cl	79	4.18
8	**7h**	H	OMe	H	H	H	Me	Cl	91	>10
9	**7i**	H	OBn	H	H	H	Me	Cl	95	>10
10	**7j**	H	CO_2_Me	H	H	H	Me	Cl	81	>10
11	**7k**	H	OH	H	H	H	Me	Cl	83	>10
12	**7l**	H	Me	H	H	Me	Me	Cl	94	7.42
13	**7m**	H	Me	H	H	Bn	Me	Cl	93	>10
14	**7n**	H	Me	H	H	H	H	Cl	90	3.32
15	**7o**	H	Me	H	H	H	allyl	Cl	83	4.67
16	**7p**	H	Me	H	H	H	Bn	Cl	85	>10
17	**7q**	H	Me	H	H	H	Me	H	–^c)^	>10
18	**7r**	H	Me	H	H	H	Me	Me	89	3.69
19	**7s**	H	Me	H	H	H	Me	*p*‐OMe‐Ph	75	6.90

^a)^ Isolated yield of the iso‐oxa‐PS reaction;

^b)^ Half maximum inhibitory concentration in the autophagy assay monitoring formation of LC3‐II punctae after autophagy induction by means of amino acid starvation. Mean values, *n* = 3;

^c)^ **7q** was obtained through dehalogenation of **7c** (see Supporting Information for details). The structure of **7c** was determined via crystal structure analysis (Figure S3, Supporting Information), and the configuration of all other indofulvins were assumed to have the same configuration. Diasteriomeric ratios were determined by ^1^H NMR of the crude reaction mixtures and for all examples were >20:1.

### 2.3 Cheminformatic Analysis of Indofulvins

**Figure 5:**
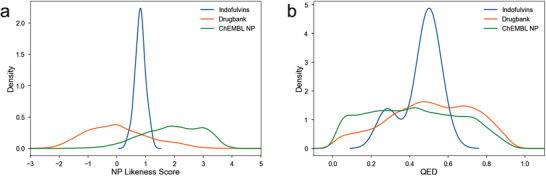
Cheminformatic analyses of the indofulvin pseudo‐NP collection. a) NP‐likeness score^[^ ^30^ ^]^ and b) quantitative estimation of drug‐likeness [QED] of the indofulvin pseudo‐NPs (blue curve) compared to the DrugBank^[^ ^32^ ^]^ compound collection (orange curve) and ChEMBL NPs^[^ ^31^ ^]^ (green curve).

The structural and physiochemical properties of the indofulvins were compared to NPs, drugs, and drug‐like compounds by cheminformatic analyses. The NP‐likeness of the pseudo‐NP collection was calculated by employing the NP‐likeness score introduced by Ertl et al.^[^ ^30^ ^]^ This analysis compares the connectivities of the new pseudo‐NP class to NPs compiled in the ChEMBL database^[^ ^31^ ^]^ and compounds in Drug Bank,^[^ ^32^ ^]^ which are characteristic for marketed and experimental drugs. The indofulvins display a narrow score distribution in an area that is only sparsely covered by NPs and drugs, i.e., they fall between both sets (**Figure** **5**a). Thus, the connectivity of the pseudo‐NP class differs from both reference sets yet may reflect a combination of their properties. This conclusion was further supported by a quantitative estimation of drug‐likeness analysis which revealed that indofulvins are at the interface of drug‐ and NP‐like physiochemical characteristics (Figure 5b). These analyses indicate that the indofulvin pseudo‐NP class may unite favorable properties of drugs and drug‐like compounds as well as NPs.

### 2.4 Biological Evaluation of Indofulvins

Since the structure of pseudo‐NPs is derived from the unprecedented combination of NP fragments, their bioactivities may also differ from the modes of action of the guiding NPs. Therefore, by analogy to the biological investigation of NPs themselves, pseudo‐NPs should be evaluated in various biological assays covering a wide range of biological programs and phenomena.

It should be noted that the design and biological goal of pseudo‐NPs is fundamentally different from NP‐hybridization and NP‐analog strategies. The NP‐hybridization strategy combines two pharmacophores into a chimeric, bifunctional molecule. While these hybrids can have an enhancement in therapeutic value due to synergistic effects, the biological effects, mode of actions, and/or targets are likely the same as the individual NP components.^[^ ^33^, ^34^, ^35^, ^36^ ^]^ Similarly, NP‐derivatization strategies aim to retain the native bioactivity of the NP while enhancing its properties and/or improving synthetic tractability.^[^ ^37^ ^]^ In contrast to these two strategies, pseudo‐NPs are designed to generate new bioactivities, mode of actions, and/or targets through fragment combination while simultaneously losing the inherent bioactivity of the individual fragments or fragment‐sized NPs.^[^ ^5^, ^6^ ^]^

The indofulvins were subjected to cell‐based assays monitoring different signaling pathways like the Wnt‐ and the Hedgehog pathways as well as assays monitoring the formation of reactive oxygen species, T‐cell proliferation, and metabolic processes like glucose uptake, and intracellular nutrient generation through recycling by means of autophagy. Gratifyingly, the indofulvins proved to define a novel chemotype for inhibitors of autophagic flux (Table 1). In autophagy, unused or damaged proteins and organelles are recycled for nutrient supply in response to stress.^[^ ^38^ ^]^ Autophagy is involved in the establishment of various diseases, particularly in cancer and neurodegeneration, and novel small molecule autophagy inhibitors may inspire drug discovery programs.^[^ ^39^, ^40^, ^41^ ^]^

For the identification of autophagy inhibitors, MCF7 cells were stably transfected with eGFP‐tagged LC3 protein, the key regulator of autophagy. Upon autophagy induction by treatment with either the mTOR inhibitor rapamycin or by amino acid starvation, eGFP‐LC3 is lipidated and integrated into newly formed autophagosomes which can be identified and quantified as punctae using automated image acquisition and analysis.^[^ ^42^ ^]^ Chloroquine (CQ) is employed as inhibitor of autophagosome‐lysosome fusion and, thereby, LC3 degradation, to enhance the dynamic range of the assay.^[^ ^43^ ^]^

The indofulvins inhibited amino acid starvation‐induced autophagy in the low‐ to sub‐micromolar range (Table 1). Rapamycin‐induced autophagy was not affected by treatment with the pseudo‐NPs, suggesting the compounds act upstream or independently of mTOR.^[^ ^44^ ^]^ Notably, the fragment‐sized NP griseofulvin **4**, saturated griseofulvin derivative **5a**, and indole‐containing fragment **3b** did not inhibit autophagy. This indicates that bioactivity of the pseudo‐NPs is a result of fragment combination and not of an individual fragment.

**Figure 6:**
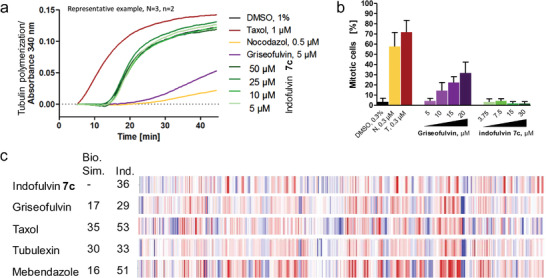
Influence of indofulvin **7c** on microtubules. a) Influence of **7c** on the in vitro tubulin polymerization. Data are representative of *n* = 3. b) Quantification of phospho‐histone H3 as a marker of mitotic cells upon treatment of MCF7 cells with **7c** for 24 h. Nocodazole (N), taxol (T), griseofulvin were used as controls. Data are mean values ± SD, *n* = 3. c) Morphological fingerprints of **7c** [10 × 10^−6^ m] and tubulin interacting references. The biological similarity (Bio. Sim.) is referred to **7c**. Induction (Ind.) as percentage of significantly changed features over DMSO control. Blue: decreased parameter, red: increased parameter.

To further substantiate that bioactivity of the newly synthesized indofulvin pseudo‐NPs differs from the biological mode of action of the guiding NPs, we investigated possible modulation of tubulin polymerization by indofulvins, since griseofulvin inhibits tubulin polymerization.^[^ ^29^ ^]^ We compared the tubulin‐stabilizing NP taxol,^[^ ^45^ ^]^ the tubulin polymerization inhibitor nocodazole^[^ ^46^ ^]^ and griseofulvin to indofulvin **7c** in an in vitro tubulin polymerization assay^[^ ^47^ ^]^ (**Figure** **6**a). While the two NPs and the synthetic nocodazole displayed the expected modulating effect, indofulvin **7c** did not affect tubulin polymerization at concentrations up to 50 × 10^−6^ m. Additionally, interference with microtubule cytoskeleton causes mitotic arrest that can be detected by means of phospho‐histone H3 as a marker (Figure 6b). While taxol, nocodazole, and griseofulvin arrested a higher fraction of cells in mitosis, indofulvin **7c** did not increase the percentage of phospho‐histone H3‐positive cells.^[^ ^48^ ^]^ These findings clearly demonstrate that bioactivity of the pseudo‐NP **7c** differs from the native bioactivity of griseofulvin.

Additionally, potential tubulin activity was investigated by morphological profiling by means of the Cell Painting Assay (CPA).^[^ ^49^, ^50^, ^51^ ^]^ In this assay, high‐content image analysis of 579 characteristic features covers a wide range of bioactivities, thereby enabling identification of biological mode of action and even targets by comparison to reference compound profiles (see Supporting Information for details).^[^ ^8^, ^9^, ^52^ ^]^ High similarities indicate that compounds may share mode‐of‐action and/or similar targets. A measure for similarity by the fraction of shared features is represented by the biological similarity (Bio. Sim., see Supporting Information for determination). Furtermore, the induction value (Ind., see Supporting Information for determination) was introduced as percentage of significantly changed features upon compound treatment compared to DMSO.

Indofulvin **7c** induced morphological changes but showed low biological similarity to its parent NP griseofulvin^[^ ^29^ ^]^ (Bio. Sim. ≤ 35%) and other references such as taxol,^[^ ^45^ ^]^ tubulexin,^[^ ^47^ ^]^ mebendazole^[^ ^53^ ^]^ that are known to interact with tubulin (Figure 6c). These major phenotypic differences indicate also on the morphological level a novel bioactivity of the pseudo‐NP as a result from the fragment combination rather than the original fragment alone.

**Figure 7:**
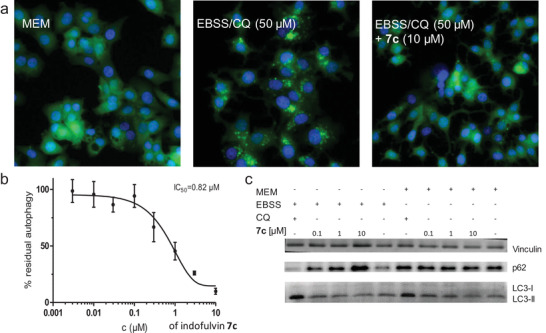
Influence of indofulvin **7c** on autophagy. a) Detection of autophagy in MCF7‐EGFP‐LC3 cells under fed conditions (MEM) and upon amino acid starvation (EBSS) in presence or absence of the compound. CQ: chloroquine. Representative images for *n* = 3 are shown. b) Dose‐dependent autophagy inhibition^[^ ^20^ ^]^ of indofulvin **7c**. Accumulation of EGFP‐LC3 upon amino acid starvation was quantified as a measure of autophagy. Data are mean values ± SD, *n* = 3. c) Detection of p62 and LC3 upon treatment with **7c** for 3 h. Vinculin was used as a control. Representative image for *n* = 3 is shown.

The indofulvins showed a structure‐dependent correlation with activity in the autophagy assay (Table 1). Unsubstituted indofulvin **7a** displayed an IC_50_ value of 2.1 × 10^−6^ m. Introduction of a methyl group in the 5‐position yielded compound **7c** with an IC_50_ = 820 × 10^−9^ m (Table 1, entry 3; **Figure** **7**a,b) and was the most potent compound in the collection. Small substituents, e.g. a fluorine in **7f**, in the 5‐position provided similar potency, whereas more sterically demanding groups, such as a benzyl ether or a methyl ester, reduced the activity (Table 1, entries 6–11). Derivatization of the indole nitrogen (Table 1, entries 12 and 13) or replacement by an oxygen (**8**, Figure 4) led to a loss in potency. In general, changes of substituents on the griseofulvin framework (Table 1, entries 15–19) and all other aromatic fragments combined with **5a** (Figure 4) lead to weaker autophagy inhibition than **7c**.

In order to further validate indofulvin **7c** as an autophagy inhibitor, we evaluated its influence on the level of the autophagy marker proteins p62 and LC3‐II.^[^ ^38^ ^]^ The chaperone p62 binds ubiquitinated proteins and, thereby, induces their autophagic clearance. Since p62 is co‐degraded with its cargo, an increase in p62 level upon compound treatment indicates inhibition of autophagic flux.^[^ ^54^ ^]^ Treatment with indofulvin **7c** increased the stability of the chaperone, as assessed by western blot (Figure 7c and Figure S1, Supporting Information). As described above, indofulvin **7c** induced a dose‐dependent reduction of EGFP‐LC3‐II puncta upon autophagy induction by amino acid starvation in the initial assay.^[^ ^42^ ^]^ By analogy, the level of the membrane‐bound lipidated LC3‐II protein was also decreased upon treatment with the compound (Figure 7c and Figure S1, Supporting Information), thereby further confirming autophagy inhibition.

**Figure 8:**
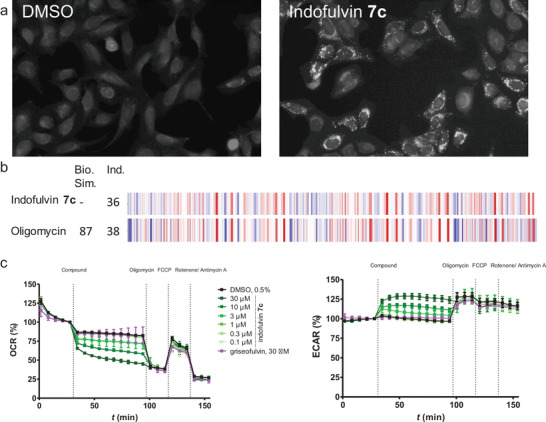
Influence of indofulvin **7c** on mitochondrial respiration. a) MitoTracker images for U‐2OS cells after treatment with 10 × 10^−6^ m indofulvin **7c** for 24 h. b) Morphological fingerprints of **7c** and Oligomycin. The biological similarity (Bio. Sim.) is in reference to **7c**. Induction (Ind.) as percentage of significantly changed features over DMSO control. Blue: decreased parameter, red: increased parameter. c) Influence of indofulvin **7c** on mitochondrial respiration in MCF7 cells. The assay was performed in a Seahorse XFe96 Analyzer, and oxygen consumption rate (OCR) and extracellular acidification rate (ECAR) were measured time‐dependently. Data are mean values ± SD, *n* = 3.

The obtained cell painting data, in particular the microscope images recorded for staining with the Mito Tracker Deep Red dye, indicate significant morphological changes of the mitochondria compared to the DMSO control (**Figure** **8**a).^[^ ^49^ ^]^ Since mitochondria have been shown to play a role in autophagy^[^ ^55^, ^56^ ^]^ and the changes of the features induced by indofulvin **7c** were found to share a high biosimilarity of 87% to oligomycin (Figure 8b), which targets mitochondrial respiration,^[^ ^57^ ^]^ pseudo‐NP **7c** may share a similar mode‐of‐action. Interestingly, other inhibitors of mitochondrial respiration with different targets than oligomycin showed low biosimilarities to **7c** (≤ 43%, Figure S2, Supporting Information).

To further investigate the influence of indofulvin **7c** on mitochondrial respiration, a Mito Stress Test employing the Seahorse XF analyzer was performed. This assay monitors the modulation of the oxygen consumption rate (OCR) as a measure of mitochondrial respiration^[^ ^9^, ^58^ ^]^ and extracellular acidification rate (ECAR) reflecting glycolysis respectively. Treatment with indofulvin **7c** dose‐dependently reduced the oxygen consumption rate and increased the rate of extracellular acidification (Figure 8c). These cellular effects correlate with autophagy inhibition, i.e., indofulvin analogs that did not inhibit autophagy also did not affect mitochondrial respiration, and for weaker autophagy inhibitors the impact on mitochondria was reduced (Figure S3, Supporting Information). These results suggest that autophagy inhibition by indofulvin **7c** may be mediated by modulation of mitochondrial function.

## 3 Conclusion

In conclusion, we have described a new principle for the design and synthesis of pseudo‐NPs with a biological mode of action that is different from its individual fragments. The development of mild, robust, and operationally simple conditions for the underdeveloped iso‐oxa‐Pictet‐Spengler reaction enabled the synthesis of a collection of indofulvin pseudo‐NPs which inhibit autophagy possibly by modulation of mitochondrial function. Notably, inhibition of autophagy by small molecules is an intensively pursued field of research,^[^ ^39^, ^59^ ^]^ and the indofulvins define an unprecedented autophagy inhibitor chemotype. Our results demonstrate that the combination of NP fragments in novel arrangements may give rise to new bioactivity and provide further proof of principle for the pseudo‐NP concept.

## 4 Experimental Section

### General Conditions for the Iso‐Oxa‐Pictet‐Spengler Reaction

An oven‐dried microwave vial was charged with a *β*‐aryl ethanol derivative (1.0 eq.) and anhydrous dichloromethane. After TfOH∙SiO_2_ (6.5 mol%) and a carbonyl substrate (1.1–1.5 eq.) were added, the tube was flushed with argon and the reaction mixture was stirred at room temperature for 30 min. The mixture was then filtered to remove the catalyst. The tube and the filtrate were rinsed with ethyl acetate. The organic layers were combined, concentrated in vacuo and subsequently purified via silica chromatography to afford the desired product.

### Biological Experiments

Details for all biological experiments can be found in the Supporting Information.

### Statistical Analysis

All continuous variables are expressed as mean ± SD from three biological replicates. The data sets were analyzed with one‐way ANOVA testing employing Prism softare across groups. The significance was defined as p ≤ 0.05.

## Conflict of Interest

The authors declare no conflict of interest.

## Supporting information

Supporting Information

## Data Availability

The data that supports the findings of this study are available in the supporting information of this article.
