# Supplementary material for: Synthesis of Indofulvin Pseudo‐Natural Products Yields a New Autophagy Inhibitor Chemotype
Source: Adv Sci (Weinh). 2021 Aug 4;8(19):2102042. doi: 10.1002/advs.202102042 (PMC8498912; doi:10.1002/advs.202102042)

© 2021 Wiley-VCH GmbH

ADVANCED SCIENCE
Open Access

Supporting Information

for *Adv. Sci.*, DOI: 10.1002/advs202102042

Synthesis of Indofulvin Pseudo-Natural Products Yields a New Autophagy Inhibitor Chemotype

*Annina Burhop, Sukdev Bag, Michael Grigalunas, Sophie Woitalla, Pia Bodenbinder, Axel Pahl, Sonja Sievers, Lukas Brieger, Carsten Strohmann, and Herbert Waldmann**

Supporting Information

Synthesis of Indofulvin Pseudo-Natural Products Yields a New Autophagy Inhibitor Chemotype

*Annina Burhop, Sukdev Bag, Michael Grigalunas, Sophie Woitalla, Pia Bodenbinder, Lukas Brieger, Carsten Strohmann, Axel Pahl, Sonja Sievers, and Herbert Waldmann**

Contents

Supplementary Tables and Figures 3
General methods for the chemical synthesis 6
General Procedures 6
Chemical Synthesis 7
2-Indolylethanol derivatives **1a-k** 7
Griseofulvin derivatives **5a-f** 14
Triflic acid immobilized on silica (TfOH·SiO_2) 21
Iso-oxa-Pictet-Spengler reaction 21
Indofulvins 32
X-ray analysis of compound **7c** 58
General methods for biological experiments 61
Cell Culture of Mammalian Cells 61
Cell Painting Assay Experimental and Analyses 66
References 71
LC-MS Traces of Indofulvins 72
NMR spectra 91

Supplementary Tables and Figures

Table S1: Method development of the iso-oxa-Pictet-Spengler reaction on indolylethanol **1a** and (-)-menthone **2e** by screening various different reaction conditions. * yield was determined by NMR.

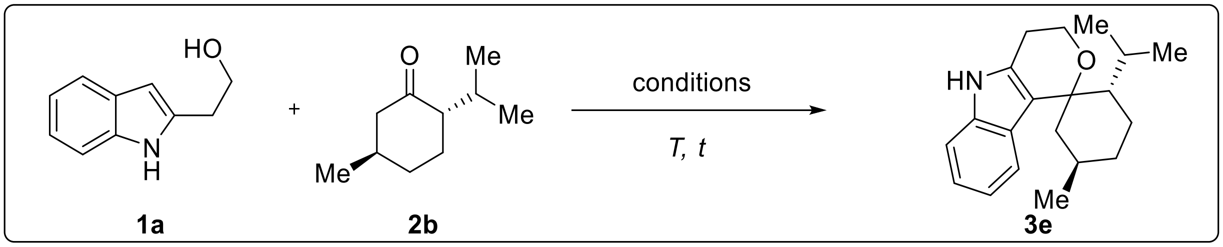

Entry	Conditions	Additives	*t* [h]	Yield* **3e** [%]
1	TsOH (10 mol%), MeOH	-	120	no conversion
2	TsOH (10 mol%), MeOH	Na_2SO_4	120	traces
3	TFA (10 mol%), MeOH	Na_2SO_4	120	10
4	$BF_3{\cdot}Et_2O$ (10 mol%), MeCN	Na_2SO_4	120	23
5	TfOH· SiO_2 (6.5 mol%), CH_2Cl_2	-	0.5	96
6	TfOH (10 mol%), CH_2Cl_2	-	120	traces
7	SiO_2 (10 mol%), CH_2Cl_2	-	120	no conversion

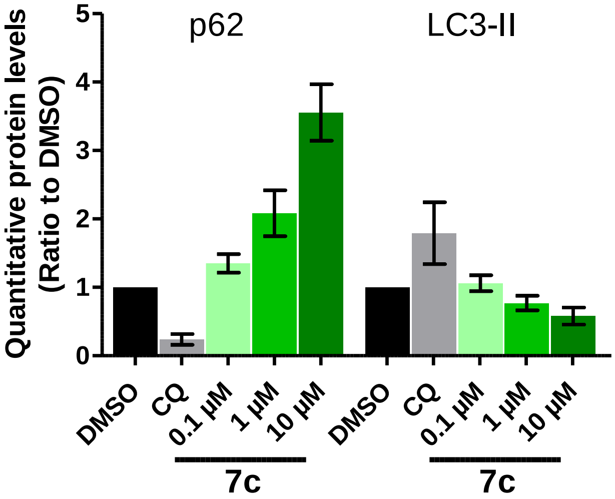

Figure S1: Quantification of the western blot for autophagy relevant proteins p62 and LC3-II under starvation conditions (-MEM) upon treatment with 7c for 3 h. Comparison to DMSO control as negative control (ratio to DMSO). Normalization to Vinculin, which was used as a control. Data are mean values ± SD, n=3. The significance was identified as $p < 0.0001$.

Compound	Bio. Sim.	Ind.
**7c**	-	36
**FCCP**	0	33
**Rotenone**	25	52
**Aumitin**	30	36
**PFP**	43	26
**Antimycin A**	0	23
**Oligomycin**	87	38

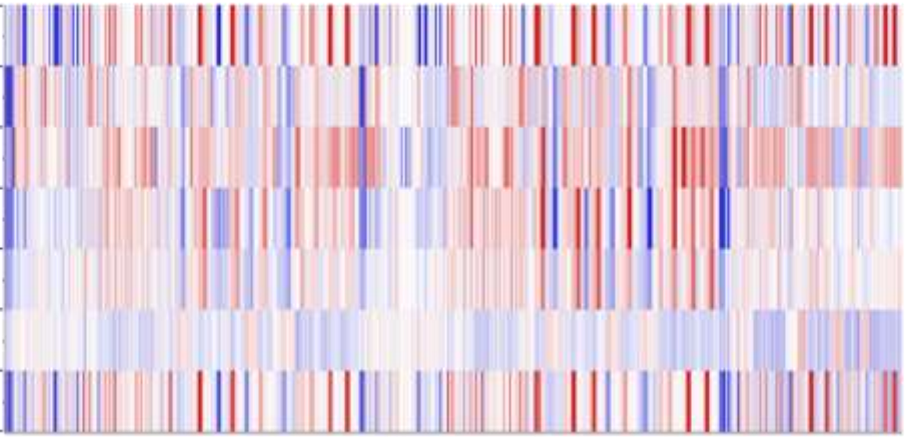

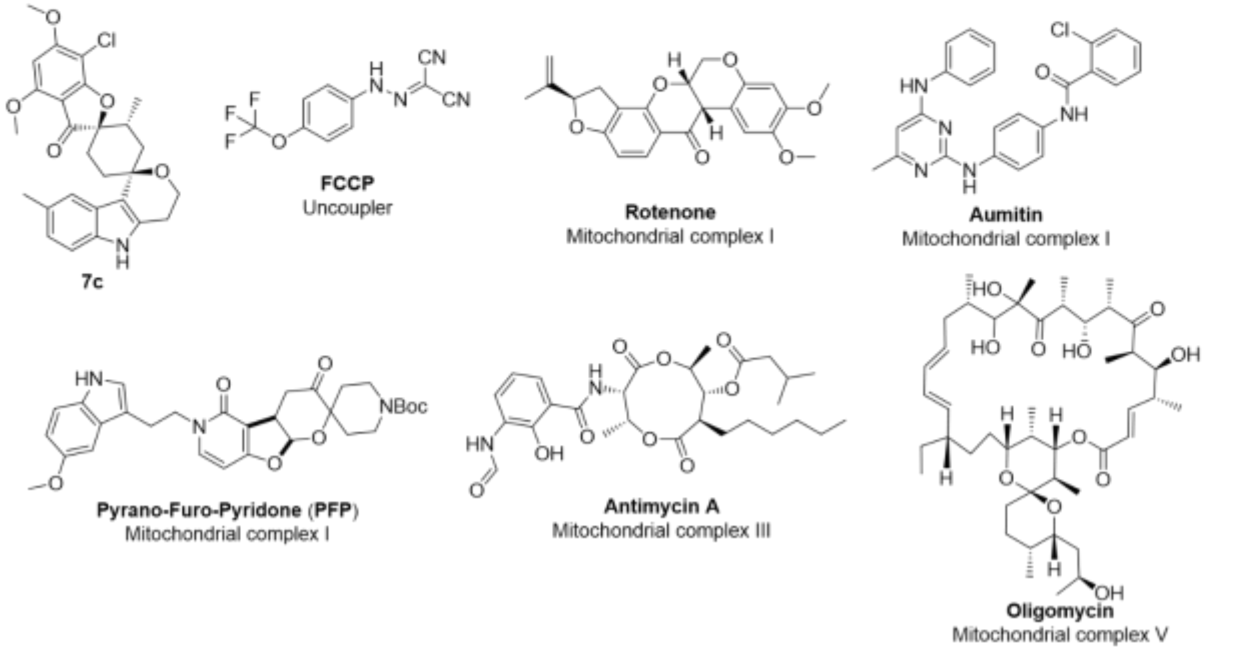

Figure S2: Comparison of indofulvin **7c** to reference compounds that affect mitochondrial respiration via the cell painting assay. Biosimilarities are with respect to **7c**. Indofulvin **7c** has a high biosimilarity to the mitochondrial complex V inhibitor oligomycin and has low biosimilarities to other inhibitors of mitochondrial respiration with different modes of action. The targets of the compounds are indicated below the compounds' names. Bio. Sim. = biosimilarity; Ind. = induction.

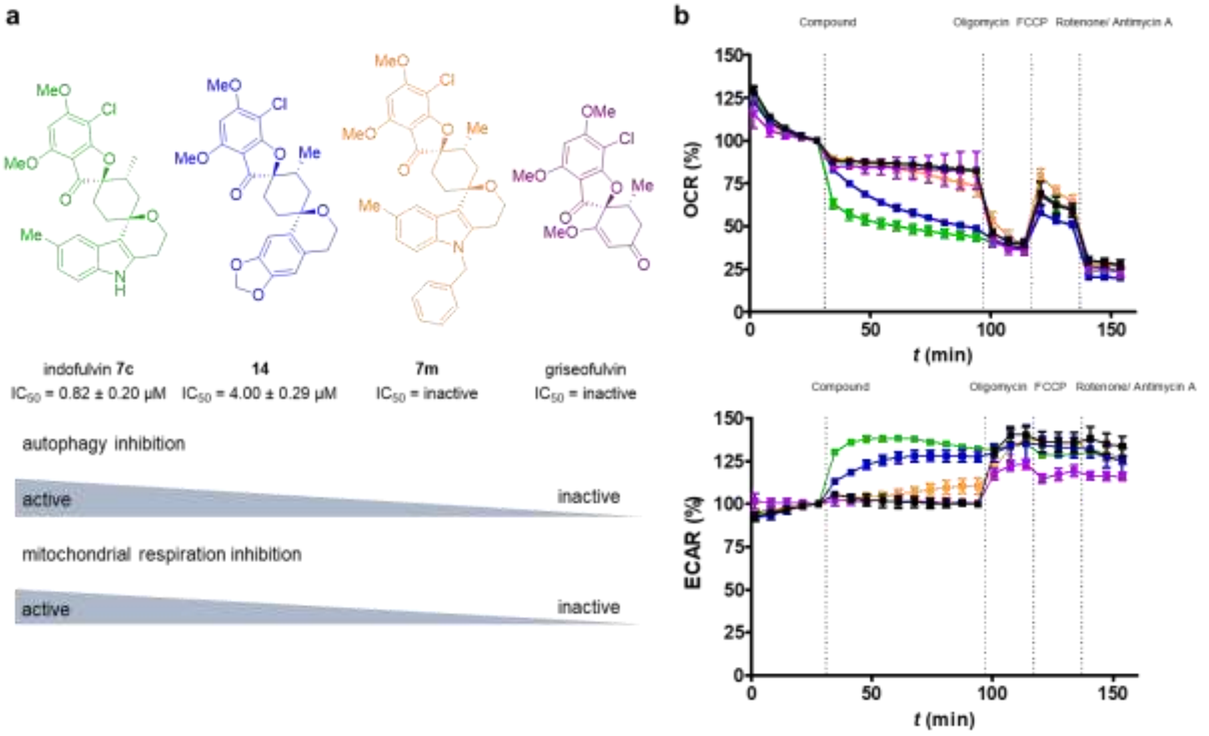

Figure S3: Correlation between the compound's autophagy inhibition and impact on the mitochondrial respiration. a: Schematic visualization of the coherence between indofulvin **7c**, compound 409397, indofulvin **7m**, and the NP griseofulvin in autophagy inhibition and mitochondrial respiration. b: Influence of indofulvin **7c**, compound 409397, indofulvin **7m**, and the NP griseofulvin on mitochondrial respiration in MCF7 cells. The assay was performed in a Seahorse XFe96 Analyzer, and oxygen consumption rate (OCR) and extracellular acidification rate (ECAR) were measured time-dependently. (n=3, N=2)

General methods for the chemical synthesis

General Procedures

All reactions with potential air or moisture sensitive reagents and/or intermediates were carried out under an argon atmosphere, and all the glassware was dried by heat gun under high vacuum prior to use. Commercial reagents were received from Sigma Aldrich, Alfa Aesar, Acros, Fisher Scientific, Merck and TCI and applied without further purification. Dry solvents were directly used from Fischer Scientific, Sigma Aldrich and/or Acros without further treatment.

Qualitative thin layer chromatography (TLC) was performed on silica coated aluminium plates (Merck 60 F254) and visualized by UV irradiation (254 nm) and/or by a potassium permanganate stain (1.5 g $KMnO_4$, 10 g K_2CO_3, 1.25 mL of 10% aqueous NaOH solution and 200 mL of water) with additional heating employing a heat gun.

Purification of crude products was achieved through flash column chromatography (FC, silica gel 60, 0.035, 0.070 mm) or automated medium pressure liquid chromatography (MPLC, Grace Reveleris X2) using the indicated solvents. Challenging separations were carried out on an Agilent 1100 preparative HPLC system equipped with a mass detector (columns: Nuleodur C18 gravity VP 125/10 5 µm, Nucleodur C18 gravity VP 125/21 5 µm, Nucleodur C4 gravity VP 125/10 5 µm). Appropriate gradient systems were applied by mixing water (+ 0.1% TFA) and either acetonitrile or methanol.

NMR spectra were recorded on Bruker AV 400 Avance III HD (NanoBay), Agilent Technologies DD2, Bruker AV 500 Avance III HD (Prodigy), Bruker AV 600 Avance III HD (CryoProbe) or Bruker AV 700 Avance III HD (CryoProbe) spectrometers. Data is reported in ppm with reference to the used deuterated solvent.[1] Isolated products as well as diastereomers could be assigned based on 2D NMR correlations ($^1H/^1H$ COSY, $^1H/^1H$ NOESY, $^1H/^{13}C$ HSQC, $^1H/^{13}C$ HMBC).

High-resolution mass spectrometry (HRMS) was performed on an LTQ Orbitrap mass spectrometer coupled to an Accela HPLC-System (HPLC column: Hypersyl GOLD, 50 mm x 1 mm, particle size 1.9 µm, ionization method: electron spray ionization (ESI)).

Chemical Synthesis

2-Indolylethanol derivatives **1a-k**

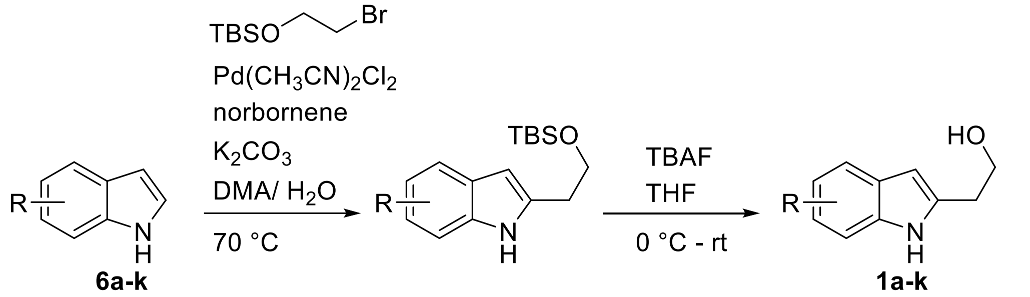

The synthesis of different 2-indolylethanol derivatives is based on the work reported by Bach et. al.[2]

Indole **6a-k** (1 eq.), norbornene (1.61 g, 0.02 mol, 2 eq.), potassium carbonate (2.36 g, 0.02 mol, 2 eq.), palladium(II) chloride diacetonitrile complex (0.22 g, 0.85 mmol, 10 mol%) and a solution of dimethylacetamide (11.2 mL) and water (0.1 mL) was added to a three-neck flask. The resulting reaction mixture was briefly evacuated and backfilled with argon (3x) and (2-bromoethoxy)(*tert*-butyl)dimethylsilane (1.83 mL, 0.01 mol, 1 eq.) was added. The mixture was heated to 70 °C and stirred for 20 h under argon. After completion, the solution was cooled to room temperature and diethyl ether (150 mL) was added. The mixture was filtrated, dried over $MgSO_4$ and concentrated *in vacuo* with the water bath set to 70 °C. The TBS-protected product was purified via flash column chromatography and directly submitted to the next step.

The TBS-protected 2-indole ethanol (1 eq.) was dissolved in dry THF (0.1 M) and cooled to 0 °C. After adding TBAF in THF (1 M, 12 mL, 2.5 eq.) dropwise, the solution was stirred until completion (rt, 4 h). Aqueous $NaHCO_3$ (10 mL) was added and the aqueous phase was extracted with ethyl acetate (5x, 15 mL). After the organic phases were combined, dried over $MgSO_4$ and concentrated, the products **1a-k** were purified via flash column chromatography (EtOAc in CycHex).

2-(1*H*-indol-2-yl)ethan-1-ol **1a**

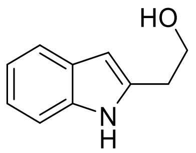

1a

682 mg, 81%. **^{1}H NMR** (400 MHz, Chloroform-*d*) δ 8.35 (s, 1H), 7.47 (ddt, *J* = 7.7, 1.5, 0.8 Hz, 1H), 7.25 (dq, *J* = 8.0, 0.9 Hz, 1H), 7.15 – 6.95 (m, 2H), 6.22 (dq, *J* = 1.8, 0.9 Hz, 1H), 3.90 (t, *J* = 5.8 Hz, 2H), 2.94 (td, *J* = 5.8, 0.8 Hz, 2H) ppm. **^{13}C NMR** (176 MHz, Chloroform-*d*) δ 137.24, 136.29, 128.72, 121.43, 120.08, 119.88, 110.93, 100.26, 77.52, 77.34, 77.16, 62.21, 31.35 ppm. **HRMS**-ESI (*m/z*): $[M + H]^+$ calculated for $C_{10}H_{11}NO^+$ 162.0841; found 162.0841.

2-(4-methyl-1*H*-indol-2-yl)ethan-1-ol **1b**

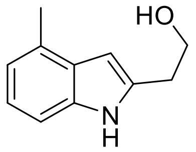

1b

281 mg, 67%. **^{1}H NMR** (600 MHz, Chloroform-*d*) δ 8.42 (s, 1H), 7.17 (d, *J* = 8.1 Hz, 1H), 7.05 (t, *J* = 7.6 Hz, 1H), 6.88 (d, *J* = 7.1 Hz, 1H), 6.31 (s, 1H), 3.97 (t, *J* = 5.8 Hz, 2H), 3.03 (t, *J* = 5.8 Hz, 2H), 2.53 (s, 3H) ppm. **^{13}C NMR** (151 MHz, Chloroform-*d*) δ 136.4, 135.9, 129.5, 128.5, 121.6, 120.0, 108.3, 99.0, 62.5, 31.4, 18.9 ppm. **HRMS**-ESI (*m/z*): $[M + H]^+$ calculated for $C_{11}H_{13}NO^+$ 176.0997; found 176.0996.

2-(5-methyl-1*H*-indol-2-yl)ethan-1-ol **1c**

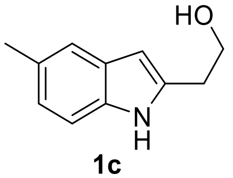

1c

775 mg, 79%. **^{1}H NMR** (700 MHz, Chloroform-*d*) δ 8.31 (s, 1H), 7.34 (s, 1H), 7.20 (d, *J* = 8.2 Hz, 1H), 6.97 (d, *J* = 8.1 Hz, 1H), 6.21 (s, 1H), 3.93 (t, *J* = 5.8 Hz, 2H), 2.98 (t, *J* = 5.8 Hz, 2H), 2.44 (s, 3H) ppm. **^{13}C NMR** (176 MHz, Chloroform-*d*) δ 137.3, 134.7, 129.2, 129.1, 123.1, 119.9, 110.5, 100.1, 62.6, 31.6, 21.8 ppm. **HRMS**-ESI (*m/z*): $[M + H]^+$ calculated for $C_{11}H_{13}NO^+$ 176.0997; found 176.0995.

2-(6-methyl-1*H*-indol-2-yl)ethan-1-ol **1d**

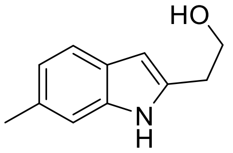

1d

592 mg, 74%. **^{1}H NMR** (400 MHz, Chloroform-*d*) δ 8.25 (s, 1H), 7.43 (d, *J* = 8.0 Hz, 1H), 7.11 (dq, *J* = 1.6, 0.8 Hz, 1H), 6.93 (ddt, *J* = 8.0, 1.5, 0.6 Hz, 1H), 6.24 (dd, *J* = 2.1, 1.0 Hz, 1H), 4.04 – 3.80 (m, 2H), 2.97 (t, *J* = 5.8 Hz, 2H), 2.46 (t, *J* = 0.7 Hz, 3H) ppm. **^{13}C NMR** (151 MHz Chloroform-*d*) δ 136.1, 128.5, 123.1, 122.3, 120.3, 119.4, 118.0, 101.2, 62.8, 31.7, 17.1 ppm. **HRMS**-ESI (*m/z*): $[M + H]^+$ calculated for $C_{11}H_{13}NO^+$ 176.0997; found 176.0995.

2-(7-methyl-1*H*-indol-2-yl)ethan-1-ol **1e**

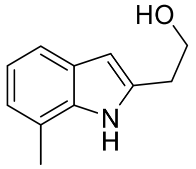

1e

468 mg, 78%. **^{1}H NMR** (400 MHz, Chloroform-*d*) *δ* 8.32 (s, 1H), 7.40 (ddt, *J* = 7.8, 1.3, 0.7 Hz, 1H), 7.07 – 6.97 (m, 1H), 6.94 (dp, *J* = 7.1, 0.9 Hz, 1H), 6.31 (dt, *J* = 2.1, 0.8 Hz, 1H), 3.98 (t, *J* = 5.2 Hz, 2H), 3.04 (td, *J* = 5.8, 0.8 Hz, 2H), 2.56 – 2.40 (m, 3H) ppm. **^{13}C NMR** (151 MHz, Chloroform-*d*) *δ* 136.2, 133.6, 129.2, 123.1, 120.5, 120.1, 117.9, 99.2, 61.7, 30.1, 16.4 ppm. **HRMS**-ESI (*m/z*): $[M + H]^+$ calculated for $C_{11}H_{13}NO^+$ 176.0997; found 176.0992.

2-(5-fluoro-1*H*-indol-2-yl)ethan-1-ol **1f**

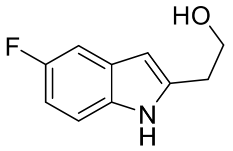

1f

310 mg, 71%. **^{1}H NMR** (700 MHz, Chloroform-*d*) *δ* 7.22 (dd, *J* = 8.7, 4.4 Hz, 1H), 7.18 (dd, *J* = 9.6, 2.4 Hz, 1H), 6.87 (td, *J* = 9.1, 2.5 Hz, 1H), 6.25 (s, 1H), 3.99 (t, *J* = 5.7 Hz, 2H), 3.01 (t, *J* = 5.7 Hz, 2H) ppm. **^{13}C NMR** (176 MHz, Chloroform-*d*) *δ* 158.9, 139.4, 132.9, 129.2, 111.4, 109.8, 105.1, 100.8, 62.6, 31.5 ppm. **HRMS**-ESI (*m/z*): $[M + H]^+$ calculated for $C_{10}H_{10}FNO^+$ 180.0746; found 180.0744.

2-(5-chloro-1*H*-indol-2-yl)ethan-1-ol **1g**

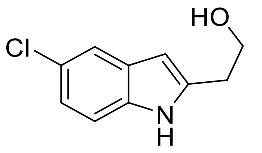

1g

213 mg, 60%. **^{1}H NMR** (700 MHz, Chloroform-d) *δ* 8.60 (s, 1H), 7.49 (d, *J* = 2.0 Hz, 1H), 7.21 (d, *J* = 8.6 Hz, 1H), 7.07 (dd, *J* = 8.6, 2.0 Hz, 1H), 6.22 (s, 1H), 3.96 (t, *J* = 5.7 Hz, 2H), 2.98 (t, *J* = 5.7 Hz, 2H) ppm. **^{13}C NMR** (176 MHz, Chloroform-*d*) *δ* 134.6, 129.7, 125.3, 121.6, 119.4, 111.6, 100.0, 62.3, 31.2 ppm. **HRMS**-ESI (*m/z*): $[M + H]^+$ calculated for $C_{10}H_{10}ClNO^+$ 196.0451 and 198.0421; found 196.0450 and 198.0419.

2-(5-methoxy-1*H*-indol-2-yl)ethan-1-ol **1h**

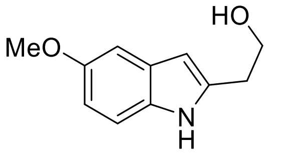

1h

355 mg, 83%. **^{1}H NMR** (700 MHz, Chloroform-*d*) *δ* 8.33 (s, 1H), 7.20 (d, *J* = 8.7 Hz, 1H), 7.02 (d, *J* = 2.3 Hz, 1H), 6.80 (dd, *J* = 8.7, 2.3 Hz, 1H), 6.22 (s, 1H), 3.95 (t, *J* = 5.8 Hz, 2H), 3.84 (s, 3H), 2.99 (t, *J* = 5.8 Hz, 2H) ppm. **^{13}C NMR** (176 MHz, Chloroform-*d*) *δ* 154.5, 138.1, 131.6, 129.3, 111.6, 102.3, 100.5, 62.6, 56.2, 31.6, 27.2 ppm. **HRMS**-ESI (*m/z*): $[M + H]^+$ calculated for $C_{11}H_{13}NO_2^+$ 192.0946; found 192.0945.

2-(5-(benzyloxy)-1*H*-indol-2-yl)ethan-1-ol **1i**

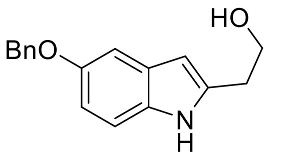

1i

291 mg, 85%. **^{1}H NMR** (700 MHz, Chloroform-*d*) δ 8.33 (s, 1H), 7.47 (d, J = 7.5 Hz, 2H), 7.38 (t, J = 7.5 Hz, 2H), 7.31 (t, J = 7.5 Hz, 1H), 7.21 (d, J = 8.7 Hz, 1H), 7.10 (d, J = 2.3 Hz, 1H), 6.88 (dd, J = 8.7, 2.3 Hz, 1H), 6.21 (s, 1H), 5.10 (s, 2H), 3.95 (t, J = 5.8 Hz, 2H), 2.99 (t, J = 5.8 Hz, 2H) ppm. **^{13}C NMR** (176 MHz, Chloroform-*d*) δ 152.3, 136.8, 136.8, 130.4, 127.9, 127.5, 126.7, 126.5, 111.0, 110.2, 102.7, 99.2, 70.0, 30.3, 25.9 ppm. **HRMS**-ESI (*m/z*): $[M + H]^+$ calculated for $C_{17}H_{17}NO_2^+$ 268.1259; found 268.1255.

methyl 2-(2-hydroxyethyl)-1*H*-indole-5-carboxylate **1j**

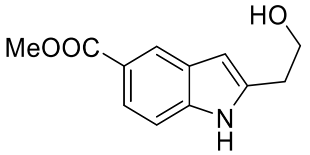

1j

61 mg, 23%. **^{1}H NMR** (500 MHz, Chloroform-*d*) δ 8.82 (s, 1H), 8.30 (s, 1H), 7.83 (s, 1H), 7.32 (s, 1H), 6.36 (s, 1H), 4.00 (s, 2H), 3.92 (s, 3H), 3.03 (s, 2H) ppm. **^{13}C NMR** (126 MHz, Chloroform-*d*) δ 168.8, 139.3, 139.1, 128.4, 123.2, 123.1, 122.0, 110.6, 101.8, 62.6, 52.2 ppm. **HRMS**-ESI (*m/z*): $[M + H]^+$ calculated for $C_{12}H_{13}NO_3^+$ 220.0895; found 220.0895.

2-(2-hydroxyethyl)-1*H*-indol-5-ol **1k**

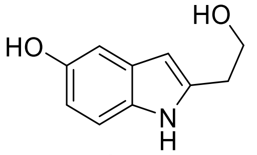

1k

172 mg, 67%. **^{1}H NMR** (700 MHz, Methanol-d4) *δ* 7.11 (d, *J* = 8.6 Hz, 1H), 6.84 (d, *J* = 2.4 Hz, 1H), 6.60 (dd, *J* = 8.6, 2.4 Hz, 1H), 6.07 (s, 1H), 4.12 (q, *J* = 7.1 Hz, 1H), 3.87 (t, *J* = 7.1 Hz, 2H), 2.98 – 2.92 (m, 2H), 2.04 (s, 1H), 1.26 (t, *J* = 7.1 Hz, 1H) ppm. **^{13}C NMR** (176 MHz, Methanol-d4) *δ* 151.6, 139.2, 133.2, 131.3, 112.2, 111.5, 105.2, 100.2, 63.0, 33.1 ppm. **HRMS**-ESI (*m/z*): [M + H]$^+$ calculated for $C_{10}H_{11}NO_2^+$ 178.0790; found 178.0790.

Griseofulvin derivatives **5a-f**

(2*R*,2'*R*)-7-Chloro-4,6-dimethoxy-2'-methyl-3H-spiro[benzofuran-2,1'-cyclohexane]-3,4'-dione **5a**

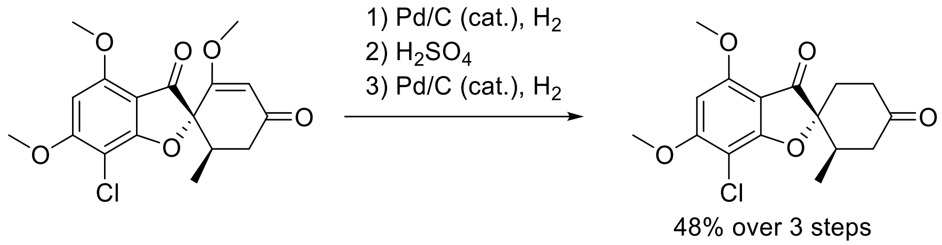

To an oven-dried three-neck flask was added griseofulvin (10.583 g, 30 mmol), 10% by weight Pd/C (500mg) and anhydrous ethyl acetate (100 ml) under an atmosphere of Ar. The reaction mixture was purged with H_2 for 10 min, then a balloon of H_2 was attached and the reaction was stirred at 22 °C. After 54 h, the reaction was purged with Ar for 10 min. The mixture was filtered through Celite and washed with dichloromethane. The eluent was concentrated and used in the next step without further purification.

To 500 ml flask containing the crude hydrogenated product was added AcOH (200 ml) and an aqueous solution of 2*M* H_2SO_4 (60 ml). The reaction was heated to 80 °C for 16 h. After this time, the reaction was cooled to room temperature and diluted with EtOAc (200 ml) and poured into ice water (200 ml). The organic layer was washed three times with water, dried over Na_2SO_4 and concentrated. The crude product was used in the next step without further purification.

To an oven-dried three-neck flask was added the above crude enone, 10% by weight Pd/C (600mg) and anhydrous ethyl acetate (80 ml) under an atmosphere of Ar. The reaction mixture was purged with H_2 for 10 min, then a balloon of H_2 was attached and the reaction was stirred at 22 °C. After 6 h, the reaction was purged with Ar for 10 min. The mixture was filtered through Celite, washed with dichloromethane, and concentrated. Purification by MPLC (silica gel, 10-36% EtOAc in CyHex) afforded 4.7g (48% over three steps) of the title compound **5b** as a white solid.

^{1}H NMR (400 MHz, Chloroform-*d*) δ 6.10 (s, 1H), 4.00 (s, 3H), 3.97 (s, 3H), 2.98 (dt, *J* = 15.2, 8.9 Hz, 1H), 2.87 (dd, *J* = 16.0, 12.5 Hz, 1H), 2.54 – 2.42 (m, 3H), 2.20 (dd, *J* = 8.8, 5.2 Hz, 2H), 0.92 (d, *J* = 6.5 Hz, 3H) ppm. **^{13}C NMR** (101 MHz, $CDCl_3$) δ 209.1, 197.4, 168.0, 164.4, 157.8, 105.5, 97.4, 90.5, 89.2, 57.0, 56.4, 43.9, 38.4, 36.2, 31.18 15.1 ppm. **HRMS (ESI)** calcd for $C_{16}H_{16}ClO_5$ [M + H] 325.08373 and 327.08078, found 325.08375 and 327.08073. $[\alpha]_D^{20}$ +30.1 (*c* 0.3 , $CHCl_3$).

(2*R*,2'*R*)-7-chloro-4-hydroxy-6-methoxy-2'-methyl-3H-spiro[benzofuran-2,1'-cyclohexane]-3,4'-dione **5b**

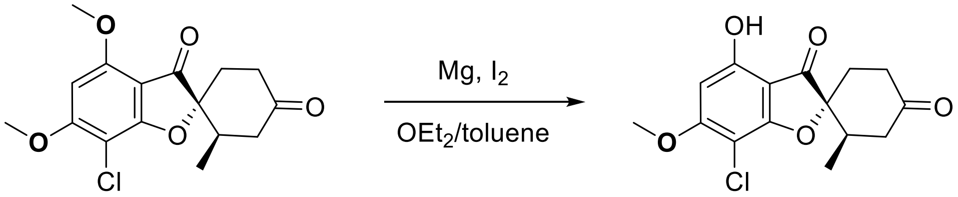

5a **5b**

A modified procedure was used to synthesized different griseofulvin derivatives based on a study by Rønnest, et. al.[3]

A solution of MgI_2 was prepared by adding magnesium turnings (124 mg, 5.2 mmol) and iodine (216 mg, 1.72 mmol) to anhydrous Et_2O (8 mL) and toluene (4 mL) in an oven-dried Schlenk tube under an atmosphere of Ar. This mixture was heated at 60 °C for 3 h and light was excluded. The mixture was cooled to room temperature and stirring was stopped. To an oven-dried pressure vessel under an atmosphere of Argon was added the griseofulvin ketone (290 mg, 0.89 mmol). 11 ml of the MgI_2 solution was taken up in a syringe and added to pressure vessel through an HPLC filter to get rid of the residual solid Mg. The pressure vessel was sealed and heated to 80 °C for 20 h. After this time, the solution was cooled to room temperature and 0.2 M H2SO4 (15 ml) was added. The mixture was extracted 3 times with EtOAc, dried (Na_2SO4) and concentrated. The crude material was recrystallized with EtOAc and heptane to provide 267 mg (96%) of **5b** as a white solid.

^{1}H NMR (700 MHz, Chloroform-*d*) δ 7.92 (s, 1H), 6.16 (s, 1H), 3.97 (s, 3H), 2.93 (dt, *J* = 16.1, 8.5 Hz, 1H), 2.80 (dd, *J* = 14.8, 10.7 Hz, 1H), 2.64 – 2.47 (m, 3H), 2.26 (dd, *J* = 8.5, 5.6 Hz, 2H), 0.97 (d, *J* = 6.8 Hz, 3H) ppm. **^{13}C NMR** (176 MHz, $CDCl_3$) δ 208.5, 201.0, 165.8, 165.5, 156.3, 104.2, 96.7, 93.4, 91.6, 57.3, 44.1, 38.3, 36.3, 30.8, 15.2 ppm. **HRMS (ESI)** calculated for $C_{15}H_{15}ClO_5$ [M + H] 311.06808 and 313.06513, found 311.06820 and 313.06522. **$[\alpha]_D^{20}$** +31.2 (*c* 0.39, $CHCl_3$).

(2*R*,2'*R*)-4-(allyloxy)-7-chloro-2',6-dimethyl-3*H*-spiro[benzofuran-2,1'-cyclohexane]-3,4'-dione **5c**

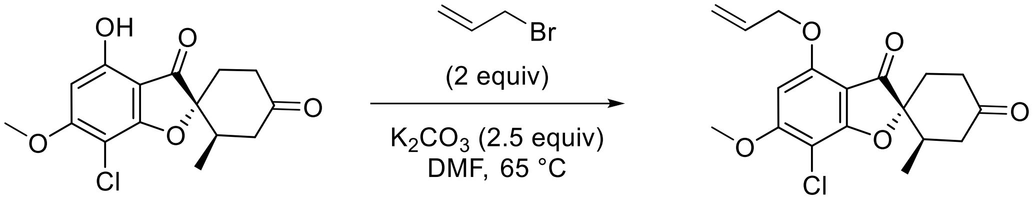

A mixture of phenol **5b** (300 mg, 0.97 mmol, 1 equiv.) and anhydrous K_2CO_3 (34 mg, 2.41 mmol, 2.5 equiv.), and allyl bromide (167 µl, 1.93 mmol, 2 equiv.) in dry DMF (6 mL) was stirred for 3 h at 65 °C under an atmosphere of Ar. The reaction mixture was cooled to room temperature, filtered and quenched with ice water (10 ml). The resulting mixture was extracted with ethyl acetate (2 x 20 ml) and the combined organic layers were washed with water (2 x 5 ml) followed by brine (10 ml). The organic phase was dried over Na_2SO_4 and concentrated to afford 320 mg (95%) of the title compound as a white solid. No further purification was needed.

^{1}H NMR (500 MHz, Chloroform-*d*) δ 6.14 – 6.01 (m, 2H), 5.51 (dq, *J* = 17.4, 1.6 Hz, 1H), 5.36 (dq, *J* = 10.5, 1.4 Hz, 1H), 4.71 (d, *J* = 5.2 Hz, 2H), 3.96 (s, 3H), 3.06 – 2.95 (m, 1H), 2.94 – 2.82 (m, 1H), 2.57 – 2.42 (m, 3H), 2.26 – 2.15 (m, 2H), 0.93 (d, *J* = 6.5 Hz, 3H) ppm. **^{13}C NMR** (126 MHz, $CDCl_3$) δ 209.2, 197.2, 168.0, 164.1, 156.8, 131.8, 118.8, 105.7, 97.4, 90.6, 90.4, 70.1, 56.9, 43.9, 38.4, 36.2, 31.2, 15.2 ppm. **HRMS (ESI)** calcd for $C_{18}H_{19}ClO_5$ [M + H] 351.09938 and 353.09643, found 351.09966 and 353.09689; **$[\alpha]_D^{20}$** +37.3 (*c* 0.26, $CHCl_3$).

(2*R*,2'*R*)-4-(benzyloxy)-7-chloro-2',6-dimethyl-3H-spiro[benzofuran-2,1'-cyclohexane]-3,4'-dione **5d**

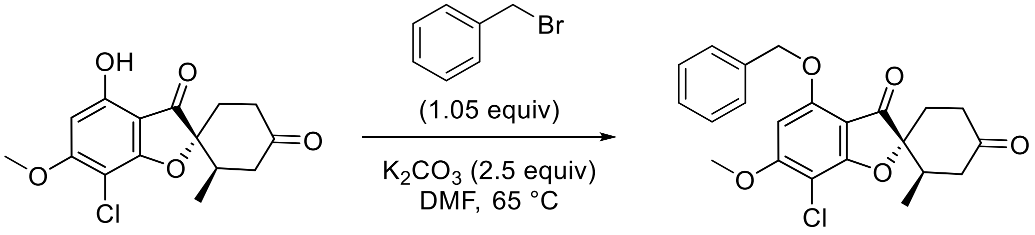

5b **5d**

A mixture of phenol **5b** (100 mg, 0.32 mmol, 1 equiv.) and anhydrous K_2CO_3 (111 mg, 0.80 mmol, 2.5 equiv.), and benzyl bromide (40 µl, 0.34 mmol, 1.05 equiv.) in dry DMF (2 mL) was stirred for 3 h at 65 °C under an atmosphere of Ar. The reaction mixture was cooled to room temperature, filtered and quenched with ice water (10 ml). The resulting mixture was extracted with ethyl acetate (2 x 20 ml) and the combined organic layers were washed with water (2 x 5 ml) followed by brine (10 ml). The organic phase was dried over Na_2SO_4 and concentrated. Purification by MPLC (silica gel, 5-30% EtOAc in CyHex) afforded 123 mg (95%) of the title compound **5d** as a fluffy white solid.

^{1}H NMR (700 MHz, Chloroform-*d*) δ 7.48 (d, *J* = 7.6 Hz, 2H), 7.41 (t, *J* = 7.5 Hz, 2H), 7.34 (t, *J* = 7.4 Hz, 1H), 6.15 (s, 1H), 5.29 (s, 2H), 3.91 (s, 3H), 3.02 (ddd, *J* = 16.1, 10.6, 6.9 Hz, 1H), 2.95 – 2.88 (m, 1H), 2.52 (tt, *J* = 15.5, 5.1 Hz, 3H), 2.30 – 2.19 (m, 2H), 0.97 (d, *J* = 6.5 Hz, 3H) ppm. **^{13}C NMR** (176 MHz, $CDCl_3$) δ 209.2, 197.2, 168.1, 164.1, 156.8, 135.7, 129.0, 128.4, 126.9, 106.1, 97.7, 91.4, 90.5, 71.3, 57.0, 44.1, 38.5, 36.3, 31.2, 15.2; HRMS (ESI) calcd for $C_{22}H_{21}ClO_5$ [M + H] 401.11503 and 403.11208, found 401.11474 and 403.11181. $[\alpha]_D^{20}$ +25.5 (*c* 0.52, $CHCl_3$).

General Procedure for the Suzuki coupling reactions

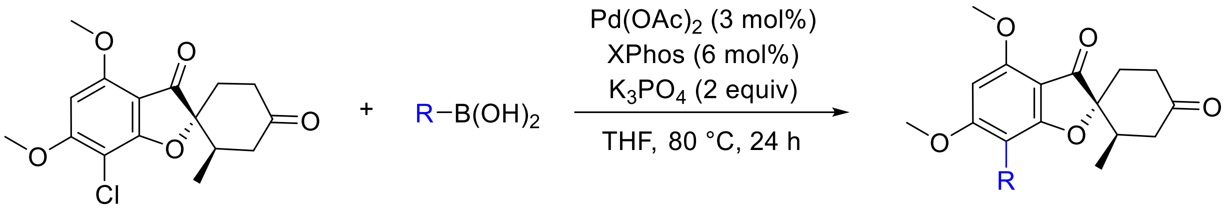

A clean, oven-dried reaction tube with previously placed magnetic stir-bar was charged with griseofulvin derivative (0.5 mmol), palladium (II) acetate (3 mol%), XPhos (6 mol%) and K_3PO_4 (2 eq.). If the boronic acid is solid, it was weighed with other solid reagents; otherwise it was added by syringe after solvent. The cap was fitted with a rubber septum and the reaction tube was evacuated and back filled with argon and this sequence was repeated three additional times. Next, THF was added to the reaction mixture. The reaction mixture was vigorously stirred (600 rpm on IKA RCT-Standard stirrer) on an oil bath/metal block at 80 ˚C temperature for 24 h. After completion of the reaction, the mixture was dried using rotary evaporator. The reaction mixture was extracted thrice with ethyl acetate (3x20 mL) and brine solution (3x10 mL). The organic layer was collected and dried over anhydrous Na_2SO_4. The solvent was evaporated under reduced pressure. The crude mixture was purified by column chromatography using silica gel and petroleum ether/ethyl acetate as the eluent.

(2*R*,2'*R*)-4,6-dimethoxy-2',7-dimethyl-3H-spiro[benzofuran-2,1'-cyclohexane]-3,4'-dione **5e**

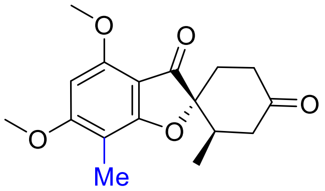

152 mg, 56%. **^{1}H NMR** (700 MHz, Chloroform-*d*) δ 6.03 (s, 1H), 3.95 (s, 3H), 3.93 (s, 3H), 3.04 (ddd, J = 15.0, 11.4, 6.5 Hz, 1H), 2.94 (dd, J = 14.4, 11.1 Hz, 1H), 2.50 – 2.40 (m, 3H), 2.21 – 2.11 (m, 2H), 2.03 (s, 3H), 0.91 (d, J = 6.7 Hz, 3H) ppm. **^{13}C NMR** (176 MHz, $CDCl_3$) δ 209.7, 198.5, 170.7, 166.9, 157.4, 104.1, 101.8, 88.8, 87.8, 56.1, 55.9, 44.0, 38.6, 36.3, 31.4, 15.1, 6.9 ppm. **HRMS** (ESI-TOF): calculated for $C_{17}H_{21}O_5$ [M+H$^+$], 305.1384; found, 305.1385. **$[\alpha]_D^{20}$** +36.2 (*c* 0.13, $CHCl_3$).

(2*R*,2'*R*)-4,6-dimethoxy-7-(4-methoxyphenyl)-2'-methyl-3*H*-spiro[benzofuran-2,1'-cyclohexane]-3,4'-dione **5f**

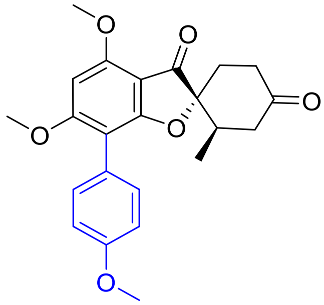

198 mg, 78%. **^{1}H NMR** (700 MHz, Chloroform-*d*) δ 7.39 (d, *J* = 8.3 Hz, 2H), 6.95 (d, *J* = 8.4 Hz, 2H), 6.18 (s, 1H), 4.02 (s, 3H), 3.90 (s, 3H), 3.83 (s, 3H), 3.07 – 2.99 (m, 1H), 2.94 (t, *J* = 14.2 Hz, 1H), 2.41 (dt, *J* = 19.6, 9.6 Hz, 3H), 2.15 (ddt, *J* = 37.5, 13.7, 7.0 Hz, 2H), 0.95 (d, *J* = 6.7 Hz, 3H) ppm. **^{13}C NMR** (176 MHz, $CDCl_3$) δ 209.5, 198.4, 170.1, 166.1, 158.7, 158.3, 131.7, 123.1, 113.5, 107.2, 104.7, 89.0, 88.6, 56.4, 56.1, 55.2, 43.9, 38.4, 36.2, 31.3, 26.9, 15.3 ppm. **HRMS** (ESI-TOF): calculated for $C_{23}H_{25}O_6$ [M+H$^+$], 397.1646; found, 397.1642. $[\alpha]_D^{20}$ +27.9 (*c* 0.147, $CHCl_3$).

(2*R*,2'*R*)-4,6-dimethoxy-2',8''-dimethyl-4'',5''-dihydro-3H,3''H-dispiro[benzofuran-2,1'-cyclohexane-4',1''-pyrano[4,3-b]indol]-3-one **7q**

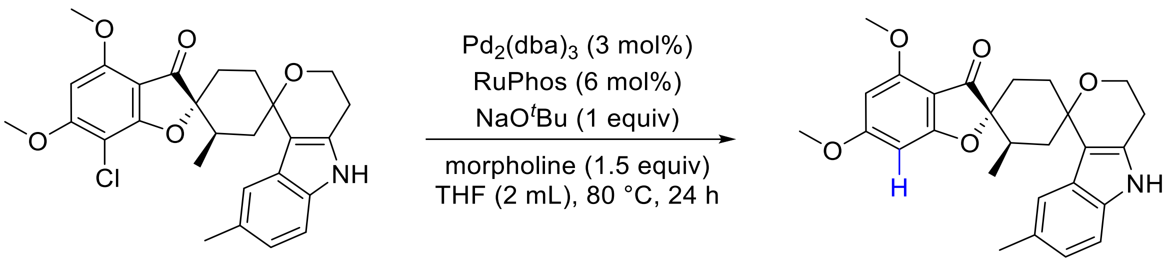

7c **7q**

The compound **7c** was prepared by following reaction condition. A clean, oven-dried reaction tube with previously placed magnetic stirbar was charged with griseofulvin-derivative **5a** (0.1 mmol), $Pd_2(dba)_3$ (3 mol%), RuPhos (6 mol%) and NaO^{t}Bu (1.5 equiv). The cap was fitted with a rubber septum and the reaction tube was evacuated and back filled with argon and this sequence was repeated three additional times. 2 mL of THF and followed by morpholine (1.5 equiv) were added to the reaction mixture. The reaction mixture was vigorously stirred (600 rpm on IKA RCT-Standard stirrer) on an oil bath of 80 ˚C temperature for 24 h. Then, the reaction mixture was dried using rotary evaporator. The reaction mixture was extracted thrice with ethyl acetate (3X20 mL) and brine solution (3X10 mL). The organic layer was collected and dried over anhydrous Na_2SO_4. The solvent was evaporated under reduced pressure. The crude mixture was purified by column chromatography using silica gel and cyclohexane/ethyl acetate (75/25, v/v) as the eluent to give the desired product **7r** as offwhite solid (18 mg, 41%).

^{1}H NMR (700 MHz, Chloroform-*d*) δ 7.93 (s, 1H), 7.72 (s, 1H), 7.19 (d, *J* = 8.2 Hz, 1H), 6.97 (dd, *J* = 8.2, 1.6 Hz, 1H), 6.16 (d, *J* = 1.8 Hz, 1H), 6.02 (d, *J* = 1.8 Hz, 1H), 4.06 – 3.98 (m, 2H), 3.96 (s, 3H), 3.88 (s, 3H), 3.00 (td, *J* = 14.3, 4.2 Hz, 1H), 2.94 (dd, *J* = 14.4, 13.0 Hz, 1H), 2.84 (ddd, *J* = 15.5, 6.6, 4.9 Hz, 1H), 2.77 (dt, *J* = 15.5, 5.0 Hz, 1H), 2.64 (ddt, *J* = 13.0, 6.8, 3.4 Hz, 1H), 2.54 (s, 3H), 2.40 (ddd, *J* = 14.1, 12.9, 4.3 Hz, 1H), 1.91 (ddt, *J* = 14.5, 4.6, 2.6 Hz, 1H), 1.84 (ddd, *J* = 14.4, 4.2, 2.6 Hz, 1H), 1.78 (ddd, *J* = 12.9, 4.2, 2.6 Hz, 1H), 0.84 (d, *J* = 6.9 Hz, 3H) ppm. **^{13}C NMR** (176 MHz, $CDCl_3$) δ 199.6, 174.9, 169.7, 158.8, 133.7, 130.8, 129.2, 125.0, 122.8, 119.6, 115.0, 110.1, 105.2, 92.4, 91.8, 88.4, 74.2, 58.6, 56.0, 55.9, 38.4, 33.6, 29.4, 28.6, 24.6, 21.8, 14.7 ppm. **135DEPT NMR** (176 MHz, $CDCl_3$) δ 122.8 (*sp*-CH), 119.6 (*sp*-CH), 110.1 (*sp*-CH), 92.4(*sp*-CH), 88.4(*sp*-CH), 58.6 (*sp3*-CH_2), 56.0 (*sp3*-CH_3), 55.9 (*sp3*-CH_3), 38.4 (*sp3*-CH_2), 33.6 (*sp3*-CH), 29.4 (*sp3*-CH_2), 28.6 (*sp3*-CH_2), 24.6 (*sp3*-CH_2), 21.8 (*sp3*-CH_3), 14.7 (*sp3*-CH_3) ppm. **HRMS** (ESI-TOF): calculated for $C_{27}H_{30}NO_5$ [M+H$^+$], 448.21185; found, 448.21152. **$[\alpha]_D^{20}$** +126.1 (*c* 0.134, $CHCl_3$).

Triflic acid immobilized on silica (TfOH·SiO₂)

A suspension of silica (10 g, predried at 110 °C for 30 min) in ethyl ether (100 mL) was generated. After triflic acid (0.45 mL, 5.0 mmol) was added carefully, the resulting mixture was stirred for 1 h under argon. The solvent was removed *in vacuo*. The residue was dried for 2 h at 110 °C to give the desired triflic acid immobilized on silica as a white powder (0.5 mmol/g).

Iso-oxa-Pictet-Spengler reaction

<u>General Procedure for the iso-oxa-Pictet-Spengler reaction</u>:

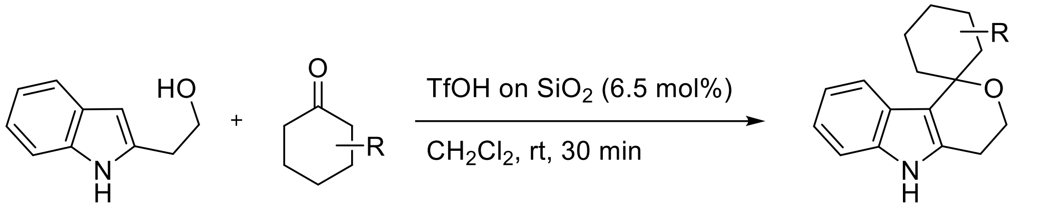

1a **2a-i** **3a-i**

An oven-dried microwave vial was charged with the corresponding *β*-aryl ethanol (1.0 eq.) and anhydrous dichloromethane (3 ml). After TfOH·SiO_2 (6.5 mol%) and cyclic ketone (1.1 – 1.5 eq.) were added, the tube was flushed with argon and the reaction mixture was stirred at room temperature for 30 min. The mixture was filtered to remove the catalyst and the tube and the filtrate were rinsed with ethyl acetate. The organic layers were combined, concentrated *in vacuo* and subsequently purified via flash column chromatography (EtOAc in CycHex (0-50%) to give the desired products **3a-l,** indofulvins **7a-q,** and aromatic derivatives **8**-**14**.

4',5'-dihydro-3'H-spiro[cyclopentane-1,1'-pyrano[4,3-b]indole] **3a**

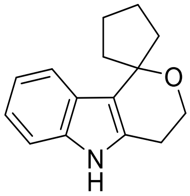

3a

38 mg (for 0.2 mmol), 84%. **^{1}H NMR** (500 MHz, Chloroform-*d*) δ 7.89 (s, 1H), 7.45 (dd, *J* = 7.8, 1.2 Hz, 1H), 7.32 (dt, *J* = 8.1, 1.0 Hz, 1H), 7.15 (ddd, *J* = 8.1, 7.1, 1.3 Hz, 1H), 7.10 (ddd, *J* = 8.1, 7.1, 1.1 Hz, 1H), 4.00 (t, *J* = 5.4 Hz, 2H), 2.80 (t, *J* = 5.4 Hz, 2H), 2.28 – 2.17 (m, 2H), 2.07 – 1.95 (m, 4H), 1.95 – 1.86 (m, 2H). **^{13}C NMR** (126 MHz, $CDCl_3$) δ 135.7, 131.4, 124.7, 121.1, 119.4, 118.5, 113.8, 110.9, 84.8, 59.6, 38.3, 24.6, 24.4. **HRMS**-ESI (*m/z*): $[M + H]^+$ calculated for $C_{15}H_{18}NO$ $[M+H]^+$: 228.1383, found 228.1388.

4',5'-dihydro-3'*H*-spiro[cyclohexane-1,1'-pyrano[4,3-*b*]indole] **3b**

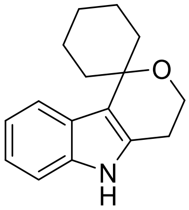

3b

23.7 mg, 98%. **^{1}H NMR** (500 MHz, Chloroform-*d*) *δ* 7.92 (s, 1H), 7.67 – 7.53 (m, 1H), 7.31 (dd, *J* = 7.4, 1.4 Hz, 1H), 7.20 – 7.07 (m, 2H), 4.02 (t, *J* = 5.4 Hz, 2H), 2.79 (t, *J* = 5.5 Hz, 2H), 2.14 – 2.02 (m, 2H), 1.97 (dq, *J* = 14.2, 2.1 Hz, 2H), 1.87 – 1.75 (m, 4H), 1.64 (dt, *J* = 12.7, 3.2 Hz, 2H) ppm. **^{13}C NMR** (126 MHz, Chloroform-*d*) *δ* 136.0, 131.2, 125.1, 121.3, 119.7, 119.4, 117.2, 111.2, 74.7, 58.6, 35.8, 26.1, 24.9, 22.0 ppm. **HRMS**-ESI (*m/z*): $[M + H]^+$ calculated for $C_{16}H_{19}NO$ $[M+H]^+$: 242.1467, found 242.1462.

4',5'-dihydro-3'H-spiro[cycloheptane-1,1'-pyrano[4,3-b]indole] **3c**

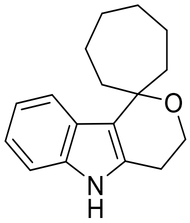

3c

44 mg (for 0.2 mmol), 86%. **^{1}H NMR** (500 MHz, Methylene Chloride-*d*$_2$) δ 7.84 (s, 1H), 7.45 (dd, *J* = 7.8, 1.2 Hz, 1H), 7.22 (dt, *J* = 8.0, 1.0 Hz, 1H), 7.00 (ddd, *J* = 8.1, 7.1, 1.3 Hz, 1H), 6.95 (ddd, *J* = 8.2, 7.1, 1.2 Hz, 1H), 3.88 (t, *J* = 5.5 Hz, 2H), 2.67 (t, *J* = 5.5 Hz, 2H), 2.08 (ddd, *J* = 14.8, 11.1, 2.4 Hz, 2H), 1.93 (ddt, *J* = 15.3, 7.4, 2.0 Hz, 2H), 1.84 – 1.74 (m, 2H), 1.69 (tdt, *J* = 9.3, 6.0, 3.4 Hz, 2H), 1.64 – 1.56 (m, 2H), 1.53 (dtd, *J* = 8.9, 6.6, 3.9 Hz, 2H). **^{13}C NMR** (126 MHz, CD_2Cl_2) δ 135.6, 130.0, 124.6, 120.8, 119.0, 119.0, 118.3, 110.7, 77.2, 58.1, 39.3, 28.0, 26.9, 24.3, 21.6. **HRMS**-ESI (*m/z*): [M + H]$^+$ calculated for $C_{17}H_{22}NO$ [M+H]$^+$: 256.1696, found 256.1701.

(3*S*)-3-methyl-4',5'-dihydro-3'*H*-spiro[cyclohexane-1,1'-pyrano[4,3-*b*]indole] **3d**

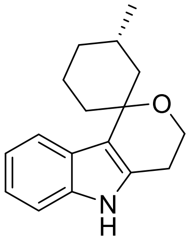

3d

20.9 mg (for 0.1 mmol), 82%, d.r. 9:1. **^{1}H NMR** (700 MHz, Chloroform-*d*) *δ* 7.78 (s, 1H), 7.33 (dd, *J* = 8.0, 1.0 Hz, 1H), 7.32 – 7.28 (m, 1H), 7.13 (d, *J* = 8.0 Hz, 1H), 6.89 (dd, *J* = 8.0, 1.0 Hz, 1H), 3.99 – 3.87 (m, 2H), 2.78 – 2.65 (m, 2H), 1.93 – 1.87 (m, 4H), 1.80 – 1.69 (m, 2H), 1.65 – 1.60 (m, 1H), 1.55 – 1.49 (m, 1H), 1.00 (d, *J* = 6.0 Hz, 1H), 0.87 (d, *J* = 6.0 Hz, 3H) ppm. **^{13}C NMR** (176 MHz, Chloroform-*d*) *δ* 133.2, 130.7, 127.8, 124.6, 121.5, 118.8, 115.9, 111.0, 74.4, 57.4, 43.8, 33.8, 27.7, 24.0, 21.8, 21.4, 20.1 ppm. **HRMS**-ESI (*m/z*): [M + H]$^+$ calculated for $C_{17}H_{21}NO$ [M+H]$^+$: 256.1624, found 256.1629. $[\alpha]_D^{20}$ +40.0 (*c* 0.1, $CHCl_3$).

(2*S*,5*R*)-2-isopropyl-5-methyl-4',5'-dihydro-3'*H*-spiro[cyclohexane-1,1'-pyrano[4,3-*b*]indole] **3e**

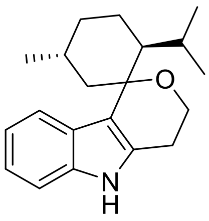

3e

28.6 mg (for 0.1 mmol), 96%. **^{1}H NMR** (500 MHz, Chloroform-*d*) δ 7.81 (s, 1H), 7.51 (d, J = 7.8 Hz, 1H), 7.32 (d, J = 8.0 Hz, 1H), 7.09 (dt, J = 28.6, 7.5 Hz, 2H), 4.06 (d, J = 17.0 Hz, 1H), 3.90 (d, J = 22.5 Hz, 1H), 2.99 (d, J = 32.8 Hz, 1H), 2.52 (d, J = 15.5 Hz, 1H), 2.13 (d, J = 16.5 Hz, 1H), 1.95 (d, J = 12.5 Hz, 1H), 1.87 (s, 2H), 1.77 – 1.66 (m, 1H), 1.50 (d, J = 62.0 Hz, 3H), 1.11 (td, J = 12.9, 3.8 Hz, 1H), 0.89 (d, J = 6.4 Hz, 3H), 0.82 (d, J = 6.9 Hz, 3H), 0.66 (d, J = 7.0 Hz, 3H) ppm. **^{13}C NMR** (126 MHz, Chloroform-*d*) δ 135.6, 131.8, 124.8, 121.0, 119.3, 119.1, 115.6, 110.8, 79.1, 58.1, 49.5, 43.6, 35.6, 27.9, 27.4, 24.5, 23.9, 22.4, 20.7, 18.8 ppm. **HRMS**-ESI (*m/z*): $[M + H]^+$ calculated for $C_{20}H_{27}NO$ $[M+H]^+$: 298.2093, found 298.1095. $[\alpha]_D^{20}$ -99.6 (*c* 0.51, $CHCl_3$).

(5*R*,8*S*,9*R*,10*R*,13*S*,14*R*,17*S*)-10,13-dimethyl-17-((*S*)-6-methylheptan-2-yl)-1,2,4,4',5,5',6,7,8,9,10,11,12,13,14,15,16,17-octadecahydro-3'*H*-spiro[cyclopenta[*a*]phenanthrene-3,1'-pyrano[4,3-*b*]indole] **3f**

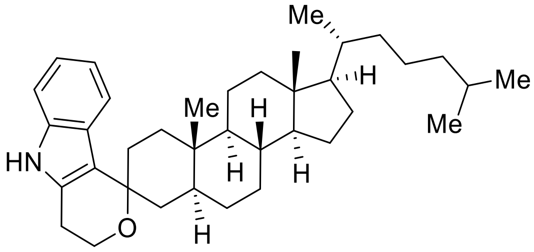

3f

47.7 mg (for 0.1 mmol), 90%. ^{1}**H NMR** (500 MHz, Chloroform-*d*) δ 7.84 (s, 1H), 7.55 (dd, *J* = 7.2, 1.7 Hz, 1H), 7.37 – 7.29 (m, 1H), 7.13 (pd, *J* = 7.2, 1.4 Hz, 2H), 4.10 – 3.89 (m, 2H), 2.90 – 2.68 (m, 2H), 2.25 – 2.15 (m, 1H), 2.15 – 2.05 (m, 1H), 2.00 (dt, *J* = 12.5, 3.4 Hz, 1H), 1.84 (ddt, *J* = 13.8, 6.3, 3.7 Hz, 2H), 1.69 (ddd, *J* = 16.1, 9.6, 3.5 Hz, 2H), 1.64 – 1.45 (m, 6H), 1.42 – 1.20 (m, 9H), 1.19 – 1.08 (m, 6H), 1.05 (s, 5H), 0.93 (d, *J* = 6.5 Hz, 4H), 0.88 (dd, *J* = 6.6, 2.4 Hz, 6H), 0.70 (s, 3H) ppm. ^{13}**C NMR** (126 MHz, Chloroform-*d*) δ 134.5, 129.9, 123.7, 120.0, 118.4, 117.6, 115.4, 109.9, 73.8, 57.2, 55.6, 55.2, 53.2, 41.6, 39.6, 39.1, 38.5, 37.6, 35.2, 34.8, 34.6, 34.4, 32.8, 31.0, 29.8, 27.5, 27.5, 27.0, 23.5, 23.2, 22.8, 21.8, 21.6, 20.0, 17.7, 11.1, 11.1 ppm. **HRMS**-ESI (*m/z*): $[M + H]^+$ calculated for $C_{37}H_{55}NO$ $[M+H]^+$: 530.4284, found 530.4276. $[\alpha]_D^{20}$ +28.3 (*c* 0.11, $CHCl_3$).

4',5'-dihydro-3'*H*-spiro[indoline-3,1'-pyrano[4,3-*b*]indol]-2-one **3g**

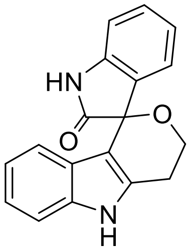

3g

15.7 mg (for 0.1 mmol), 54%. ^{1}**H NMR** (700 MHz, Methanol-*d4*) δ 7.39 – 7.26 (m, 2H), 7.14 – 6.93 (m, 4H), 6.83 – 6.67 (m, 1H), 6.60 – 6.48 (m, 1H), 4.85 – 4.77 (m, 1H), 4.27 (ddd, *J* = 7.5, 5.4, 2.7 Hz, 1H), 3.17 (ddd, *J* = 15.8, 8.7, 5.3 Hz, 1H), 2.97 (dt, *J* = 15.9, 4.0 Hz, 1H) ppm. ^{13}**C NMR** (176 MHz, Methanol-*d4*) δ 181.0, 143.5, 137.9, 135.6, 133.1, 131.5, 127.0, 125.9, 124.3, 122.5, 120.4, 118.5, 112.4, 111.6, 106.9, 79.3, 62.6, 24.8 ppm. **HRMS**-ESI (*m/z*): $[M + H]^+$ calculated for $C_{18}H_{14}N_2O_2$ $[M+H]^+$: 291.1055, found 291.1056.

4',5'-dihydro-3'*H*-spiro[chromane-4,1'-pyrano[4,3-*b*]indole] **3h**

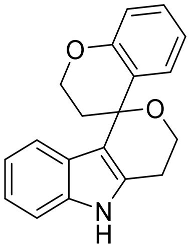

3h

10.8 mg (for 0.1 mmol), 37%. **^{1}H NMR** (500 MHz, Chloroform-*d*) δ 7.99 (s, 1H), 7.33 (d, *J* = 8.1 Hz, 1H), 7.15 (dddd, *J* = 38.1, 8.2, 7.1, 1.5 Hz, 2H), 6.99 – 6.89 (m, 3H), 6.85 (d, *J* = 7.9 Hz, 1H), 6.70 (td, *J* = 7.4, 1.2 Hz, 1H), 4.59 (ddd, *J* = 13.2, 10.8, 2.0 Hz, 1H), 4.38 (ddd, *J* = 10.8, 4.4, 2.2 Hz, 1H), 4.19 – 4.02 (m, 2H), 3.13 – 2.98 (m, 1H), 2.97 – 2.82 (m, 1H), 2.68 (ddd, *J* = 14.6, 13.5, 4.4 Hz, 1H), 2.26 (dt, *J* = 14.6, 2.1 Hz, 1H) ppm. **^{13}C NMR** (126 MHz, Chloroform-*d*) δ 155.2, 136.0, 133.2, 130.0, 125.2, 125.0, 121.9, 120.5, 120.1, 119.4, 117.3, 113.3, 111.1, 71.3, 62.9, 59.0, 33.8, 27.3, 24.7 ppm. **HRMS**-ESI (*m/z*): $[M + H]^+$ calculated for $C_{19}H_{17}NO_2$ $[M+H]^+$: 292.1259, found 292.1254.

2,3,4',5,5',6-hexahydro-3'*H*-spiro[pyran-4,1'-pyrano[4,3-*b*]indole] **3i**

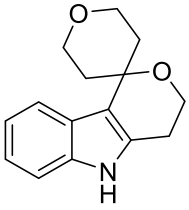

3i

21.7 mg (for 0.1 mmol), 89%. **^{1}H NMR** (500 MHz, Chloroform-*d*) δ 7.94 (s, 1H), 7.53 (d, *J* = 7.8 Hz, 1H), 7.29 – 7.22 (m, 1H), 7.06 (dtd, *J* = 21.8, 7.2, 1.2 Hz, 2H), 3.99 – 3.77 (m, 6H), 2.74 (t, *J* = 5.4 Hz, 2H), 2.45 – 2.32 (m, 2H), 1.71 (d, *J* = 14.1 Hz, 2H) ppm. **^{13}C NMR** (126 MHz, Chloroform-*d*) δ 135.6, 131.2, 124.5, 121.3, 119.6, 118.7, 115.2, 111.0, 71.8, 63.7, 58.6, 35.7, 24.5 ppm. **HRMS**-ESI (*m/z*): $[M + H]^+$ calculated for $C_{15}H_{17}NO_2$ $[M+H]^+$: 244.1259, found 244.1255.

2',3',4,5,5',6'-hexahydro-3H-spiro[pyrano[4,3-b]indole-1,4'-thiopyran] **3j**

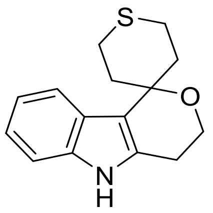

3j

49 mg (for 0.2 mmol), 94%. **^{1}H NMR** (400 MHz, Chloroform-*d*) δ 7.85 (s, 1H), 7.68 – 7.61 (m, 1H), 7.32 (dq, *J* = 8.2, 1.0 Hz, 1H), 7.19 – 7.09 (m, 2H), 4.00 (td, *J* = 5.4, 0.9 Hz, 2H), 3.30 – 3.19 (m, 2H), 2.80 (td, *J* = 5.5, 0.8 Hz, 2H), 2.49 – 2.37 (m, 4H), 2.27 – 2.14 (m, 2H). **^{13}C NMR** (126 MHz, $CDCl_3$) δ 135.5, 130.7, 124.4, 121.3, 119.6, 118.9, 116.3, 110.9, 72.5, 58.2, 36.3, 24.3, 23.8. **HRMS**-ESI (*m/z*): $[M + H]^+$ calculated for $C_{15}H_{18}NOS$ $[M+H]^+$: 260.1104, found 260.1107.

1-(4',5'-dihydro-3'H-spiro[piperidine-4,1'-pyrano[4,3-b]indol]-1-yl)ethan-1-one **3l**

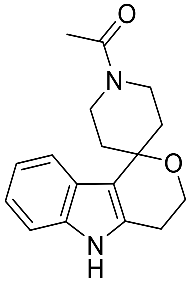

3l

49.5 mg (for 0.2 mmol), 87%. **^{1}H NMR** (700 MHz, Chloroform-*d*) δ 7.99 (s, 1H), 7.44 (dd, *J* = 8.0, 1.1 Hz, 1H), 7.33 (dt, *J* = 8.1, 0.9 Hz, 1H), 7.15 (ddd, *J* = 8.1, 7.1, 1.2 Hz, 1H), 7.09 (ddd, *J* = 8.1, 7.1, 1.1 Hz, 1H), 4.61 (ddt, *J* = 13.3, 4.7, 2.0 Hz, 1H), 4.03 (t, *J* = 5.4 Hz, 2H), 3.71 (ddt, *J* = 13.4, 4.6, 2.0 Hz, 1H), 3.58 (td, *J* = 13.3, 2.6 Hz, 1H), 3.03 (td, *J* = 13.2, 2.7 Hz, 1H), 2.89 – 2.78 (m, 2H), 2.31 – 2.21 (m, 2H), 2.19 (s, 3H), 1.92 (dt, *J* = 14.3, 2.5 Hz, 2H). **^{13}C NMR** (176 MHz, $CDCl_3$) δ 168.9, 135.6, 131.2, 124.2, 121.4, 119.7, 118.6, 114.5, 110.9, 72.3, 58.6, 42.4, 37.4, 35.7, 34.5, 24.4, 21.6. **HRMS**-ESI (*m/z*): $[M + H]^+$ calculated for $C_{17}H_{21}N_2O_2$ $[M+H]^+$: 285.1598, found 285.1602.

(4',5'-dihydro-3'H-spiro[piperidine-4,1'-pyrano[4,3-b]indol]-1-yl)(phenyl)methanone **3m**

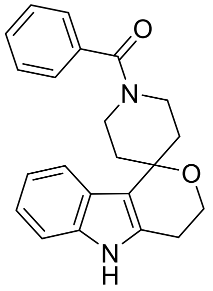

3m

48 mg (for 0.2 mmol), 69%. **^{1}H NMR** (700 MHz, Chloroform-*d*) δ 8.00 (s, 1H), 7.50 (ddd, *J* = 7.9, 3.2, 1.9 Hz, 3H), 7.46 – 7.41 (m, 3H), 7.33 (dt, *J* = 8.0, 1.0 Hz, 1H), 7.14 (dddd, *J* = 22.4, 8.2, 7.2, 1.2 Hz, 2H), 4.79 – 4.65 (m, 1H), 4.07 – 3.99 (m, 2H), 3.73 – 3.63 (m, 1H), 3.56 (d, *J* = 13.7 Hz, 1H), 3.34 – 3.20 (m, 1H), 2.82 (td, *J* = 5.4, 1.7 Hz, 2H), 2.44 – 2.34 (m, 1H), 2.31 – 2.20 (m, 1H), 2.02 (d, *J* = 14.6 Hz, 1H), 1.81 (d, *J* = 14.1 Hz, 1H). **^{13}C NMR** (176 MHz, $CDCl_3$) δ 170.4, 136.4, 135.6, 131.2, 129.5, 128.5, 126.8, 124.2, 121.4, 119.7, 118.5, 114.4, 111.0, 72.5, 58.7, 43.7, 38.2, 35.8, 34.7, 24.4. **HRMS**-ESI (*m/z*): $[M + H]^+$ calculated for $C_{22}H_{23}N_2O_2$ $[M+H]^+$: 347.1754, found 347.1758.

tert-butyl 4',5'-dihydro-3'H-spiro[piperidine-4,1'-pyrano[4,3-b]indole]-1-carboxylate **3n**

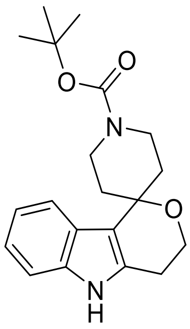

3n

62 mg (for 0.2 mmol), 91%. ^{1}H NMR (500 MHz, Chloroform-*d*) δ 8.26 (s, 1H), 7.47 (dd, *J* = 7.9, 1.1 Hz, 1H), 7.32 (dt, *J* = 8.1, 1.0 Hz, 1H), 7.14 (ddd, *J* = 8.1, 7.1, 1.2 Hz, 1H), 7.09 (ddd, *J* = 8.1, 7.0, 1.1 Hz, 1H), 4.21 – 3.89 (m, 4H), 3.39 – 3.06 (m, 2H), 2.82 (t, *J* = 6.0 Hz, 2H), 2.26 (td, *J* = 13.6, 5.0 Hz, 2H), 1.92 – 1.81 (m, 2H), 1.55 (s, 9H). **^{13}C NMR** (126 MHz, $CDCl_3$) δ 155.2, 135.6, 131.2, 124.3, 121.2, 119.5, 118.6, 114.8, 111.0, 79.5, 72.4, 58.6, 39.7 (d, *J* = 104.9 Hz), 34.8 (d, *J* = 59.3 Hz), 28.5, 24.4. **HRMS**-ESI (*m/z*): $[M + H]^+$ calculated for $C_{20}H_{27}N_2O_3$ $[M+H]^+$: 343.2016, found 343.2017.

tert-butyl 4',5'-dihydro-3'H-8-azaspiro[bicyclo[3.2.1]octane-3,1'-pyrano[4,3-b]indole]-8-carboxylate **3p**

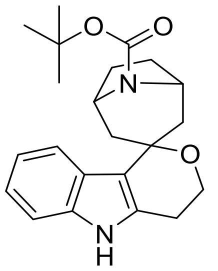

3p

13.3 mg (for 0.2 mmol), 18%. **^{1}H NMR** (700 MHz, Methylene Chloride-*d*$_2$) δ 8.01 (s, 1H), 7.40 (dd, *J* = 7.9, 1.0 Hz, 1H), 7.34 (dt, *J* = 8.1, 0.9 Hz, 1H), 7.13 (ddd, *J* = 8.2, 7.1, 1.1 Hz, 1H), 7.03 (ddd, *J* = 8.0, 7.1, 1.0 Hz, 1H), 4.27 (d, *J* = 30.0 Hz, 2H), 3.99 (t, *J* = 5.4 Hz, 2H), 2.80 (q, *J* = 5.1 Hz, 2H), 2.68 – 2.59 (m, 1H), 2.48 (d, *J* = 14.8 Hz, 1H), 2.24 (q, *J* = 10.6, 9.0 Hz, 2H), 2.03 – 1.90 (m, 4H), 1.60 (s, 9H). **^{13}C NMR** (176 MHz, CD_2Cl_2) δ 153.4, 135.6, 131.8, 124.2, 121.0, 119.2, 118.2, 115.4, 110.7, 78.9, 74.3, 58.0, 40.5, 39.5, 28.3, 27.7, 24.1. **HRMS**-ESI (*m/z*): $[M + H]^+$ calculated for $C_{22}H_{29}N_2O_3$ $[M+H]^+$: 369.2173, found 369.2176.

4-(2-(1-methyl-1,3,4,5-tetrahydropyrano[4,3-b]indol-1-yl)ethyl)phenol **3q**

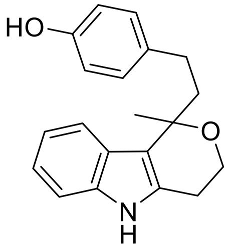

3q

48 mg (for 0.2 mmol), 78%. **^{1}H NMR** (600 MHz, Chloroform-*d*) δ 7.92 (s, 1H), 7.50 (dd, *J* = 7.8, 1.1 Hz, 1H), 7.34 (dt, *J* = 8.0, 0.9 Hz, 1H), 7.17 (ddd, *J* = 8.1, 7.0, 1.2 Hz, 1H), 7.12 (ddd, *J* = 8.1, 7.1, 1.1 Hz, 1H), 6.96 – 6.90 (m, 2H), 6.70 – 6.64 (m, 2H), 5.29 (s, 1H), 4.14 (ddt, *J* = 11.2, 5.3, 2.9 Hz, 1H), 4.05 (ddd, *J* = 11.3, 8.8, 3.9 Hz, 1H), 2.94 (ddd, *J* = 15.6, 8.8, 5.4 Hz, 1H), 2.71 – 2.63 (m, 2H), 2.34 (td, *J* = 12.9, 3.7 Hz, 1H), 2.29 – 2.16 (m, 2H), 1.66 (s, 3H). ^{13}C NMR (151 MHz, $CDCl_3$) δ 153.5, 135.7, 134.9, 131.2, 129.4, 124.7, 121.2, 119.6, 118.7, 115.1, 114.8, 110.9, 76.1, 59.1, 43.3, 29.4, 25.8, 24.3. **HRMS**-ESI (*m/z*): $[M + H]^+$ calculated for $C_{20}H_{22}NO_2$ $[M+H]^+$: 308.1645, found 308.1645.

methyl 1-methyl-1,3,4,5-tetrahydropyrano[4,3-b]indole-1-carboxylate **3r**

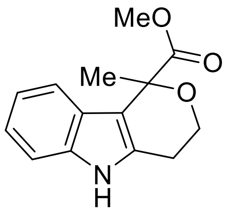

3r

31 mg (for 0.2 mmol), 63%. **^{1}H NMR** (600 MHz, Chloroform-*d*) δ 8.13 (s, 1H), 7.80 – 7.71 (m, 1H), 7.32 (dt, *J* = 8.0, 0.8 Hz, 1H), 7.23 – 7.11 (m, 2H), 4.30 – 4.20 (m, 2H), 3.76 (s, 3H), 3.01 (ddd, *J* = 16.0, 8.9, 7.2 Hz, 1H), 2.65 (dt, *J* = 15.9, 3.0 Hz, 1H), 1.87 (s, 3H). **^{13}C NMR** (151 MHz, $CDCl_3$) δ 174.2, 135.7, 131.9, 125.1, 121.5, 120.2, 119.9, 110.8, 110.3, 77.3, 61.6, 52.2, 25.8, 23.6. **HRMS**-ESI (*m/z*): $[M + H]^+$ calculated for $C_{14}H_{16}NO_3$ $[M+H]^+$: 246.1125, found 246.1127.

1-phenyl-1,3,4,5-tetrahydropyrano[4,3-b]indole **3s**

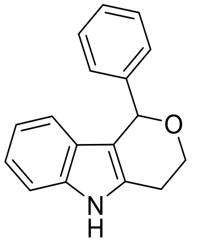

3s

28 mg (for 0.2 mmol), 56%. **^{1}H NMR** (500 MHz, Methylene Chloride-d_2) δ 8.03 (s, 1H), 7.32 – 7.27 (m, 2H), 7.27 – 7.21 (m, 4H), 6.97 (ddd, *J* = 8.2, 7.0, 1.2 Hz, 1H), 6.77 (ddd, *J* = 8.1, 7.0, 1.0 Hz, 1H), 6.68 (dd, *J* = 7.9, 1.1 Hz, 1H), 5.79 (t, *J* = 1.9 Hz, 1H), 4.10 (ddd, *J* = 11.2, 5.3, 4.0 Hz, 1H), 3.88 (ddd, *J* = 11.3, 8.4, 4.3 Hz, 1H), 2.98 (dddd, *J* = 15.8, 8.4, 5.3, 1.9 Hz, 1H), 2.74 (dtd, *J* = 16.0, 4.2, 1.8 Hz, 1H). **^{13}C NMR** (126 MHz, CD_2Cl_2) δ 141.4, 135.6, 132.4, 128.6, 128.2, 128.1, 125.4, 121.1, 119.3, 118.4, 110.6, 110.6, 76.6, 62.7, 24.2. **HRMS**-ESI (*m/z*): $[M + H]^+$ calculated for $C_{17}H_{16}NO$ $[M+H]^+$: 250.1226, found 250.1230.

1-(2-bromophenyl)-1,3,4,5-tetrahydropyrano[4,3-b]indole **3t**

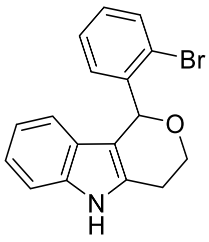

3t

29.5 mg (for 0.2 mmol), 45%. **^{1}H NMR** (500 MHz, Methylene Chloride-*d*$_2$) δ 8.06 (s, 1H), 7.62 – 7.54 (m, 1H), 7.25 (dt, *J* = 8.2, 0.9 Hz, 1H), 7.13 – 7.04 (m, 3H), 6.98 (ddd, *J* = 8.2, 7.0, 1.2 Hz, 1H), 6.79 (ddd, *J* = 8.0, 7.0, 1.0 Hz, 1H), 6.71 (dd, *J* = 7.9, 1.1 Hz, 1H), 6.26 (t, *J* = 1.8 Hz, 1H), 4.09 (ddd, *J* = 11.3, 5.2, 4.2 Hz, 1H), 3.90 (ddd, *J* = 11.3, 8.2, 4.3 Hz, 1H), 2.99 (dddd, *J* = 15.5, 8.2, 5.2, 1.8 Hz, 1H), 2.75 (dtd, *J* = 16.0, 4.3, 1.8 Hz, 1H). **^{13}C NMR** (126 MHz, CD_2Cl_2) δ 139.7, 135.6, 132.9, 132.8, 130.8, 129.7, 127.3, 125.1, 124.5, 121.3, 119.4, 118.4, 110.7, 109.9, 75.1, 62.7, 24.1. **HRMS**-ESI (*m/z*): $[M + H]^+$ calculated for $C_{17}H_{15}BrNO$ $[M+H]^+$: 328.0332, found 328.0335.

Indofulvins

(2*R*,2'*R*,4'*S*)-7-chloro-4,6-dimethoxy-2'-methyl-4'',5''-dihydro-3*H*,3''*H*-dispiro[benzofuran-2,1'-cyclohexane-4',1''-pyrano[4,3-*b*]indol]-3-one **7a**

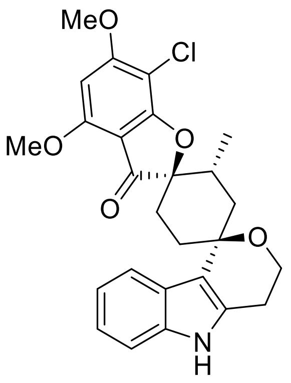

7a

43.0 mg, 92%. **^{1}H NMR** (400 MHz, Chloroform-*d*) *δ* 8.16 – 8.13 (m, 1H), 7.83 (s, 1H), 7.32 – 7.28 (m, 1H), 7.20 – 7.11 (m, 2H), 6.11 (s, 1H), 4.02 (d, *J* = 3.9 Hz, 8H), 3.04 – 2.70 (m, 5H), 2.51 (ddd, *J* = 14.1, 12.9, 4.2 Hz, 1H), 1.98 – 1.75 (m, 3H), 0.83 (d, *J* = 6.7 Hz, 3H) ppm. **^{13}C NMR** (126 MHz, Chloroform-*d*) *δ* 199.3, 168.5, 163.9, 157.5, 135.5, 130.8, 124.7, 121.3, 120.1, 120.0, 115.4, 110.5, 106.0, 93.1, 88.6, 74.0, 58.7, 56.9, 56.4, 38.4, 33.6, 31.0, 29.5, 28.6, 24.5, 14.8 ppm. **HRMS**-ESI (*m/z*): [M + H]$^+$ calculated for $C_{26}H_{26}ClNO_5$ [M+H]$^+$: 468.1500 and 470.1470, found 468.1500 and 470.1467. $[\alpha]_D^{20}$ +33.7 (*c* 0.2, $CHCl_3$).

(2*R*,2'*R*,4'*S*)-7-chloro-4,6-dimethoxy-2',9''-dimethyl-4'',5''-dihydro-3*H*,3''*H*-dispiro[benzofuran-2,1'-cyclohexane-4',1''-pyrano[4,3-*b*]indol]-3-one **7b**

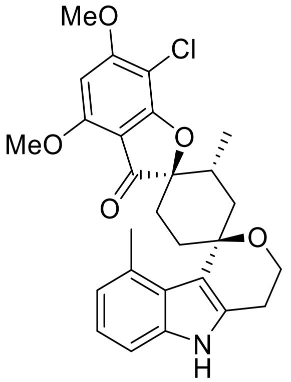

7b

42.8 mg, 89%. **^{1}H NMR** (700 MHz, Chloroform-*d*) δ 7.98 (s, 1H), 7.17 – 7.11 (m, 1H), 7.04 (t, *J* = 7.6 Hz, 1H), 6.94 (dt, *J* = 7.2, 1.1 Hz, 1H), 6.09 (s, 1H), 4.03 – 3.94 (m, 8H), 3.06 (s, 3H), 2.96 – 2.86 (m, 3H), 2.84 (dt, *J* = 5.4, 3.8 Hz, 2H), 2.58 – 2.53 (m, 1H), 2.01 (dt, *J* = 14.5, 2.3 Hz, 1H), 1.97 – 1.90 (m, 1H), 1.78 (ddd, *J* = 13.1, 4.2, 2.5 Hz, 1H), 0.83 (d, *J* = 6.1 Hz, 3H) ppm. **^{13}C NMR** (176 MHz, Chloroform-*d*) δ 198.3, 167.4, 162.8, 156.6, 135.1, 130.9, 129.3, 123.4, 121.6, 120.3, 114.6, 107.3, 104.8, 92.1, 87.6, 74.1, 56.4, 55.8, 55.3, 39.2, 32.3, 30.2, 27.5, 25.9, 24.1, 23.3, 13.2 ppm. **HRMS**-ESI (*m/z*): [M + H]$^+$ calculated for $C_{27}H_{28}ClNO_5$ [M+H]$^+$: 482.1656 and 484.1627, found 482.1653 and 484.1626.

(2*R*,2'*R*,4'*S*)-7-chloro-4,6-dimethoxy-2',8''-dimethyl-4'',5''-dihydro-3*H*,3''*H*-dispiro[benzofuran-2,1'-cyclohexane-4',1''-pyrano[4,3-*b*]indol]-3-one **7c**

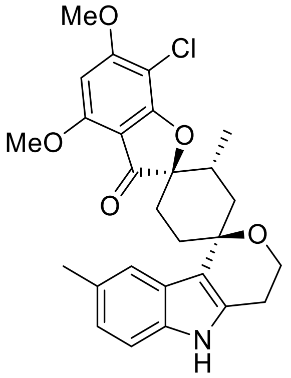

7c

45.3 mg, 94%. **^{1}H NMR** (400 MHz, Chloroform-*d*) *δ* 7.89 (s, 1H), 7.84 – 7.66 (m, 1H), 7.19 (dd, *J* = 8.1, 0.7 Hz, 1H), 6.97 (ddd, *J* = 8.3, 1.6, 0.6 Hz, 1H), 6.11 (s, 1H), 4.06 – 3.89 (m, 8H), 3.02 – 2.71 (m, 5H), 2.57 – 2.46 (m, 4H), 2.00 – 1.73 (m, 3H), 0.83 (d, *J* = 6.7 Hz, 3H). **^{13}C NMR** (176 MHz, Chloroform-*d*) *δ* 199.7, 168.8, 164.2, 157.8, 134.1, 131.2, 129.5, 125.2, 119.8, 115.2, 110.5, 106.4, 97.5, 93.5, 88.9, 74.4, 58.9, 57.2, 56.6, 38.7, 33.8, 29.8, 28.9, 27.2, 24.9, 22.1, 15.0 ppm. **HRMS**-ESI (*m/z*): [M + H]$^+$ calculated for $C_{27}H_{28}ClNO_5$ [M+H]$^+$: 482.1656 and 484.1627, found 482.1655 and 484.1626. **$[\alpha]_D^{20}$** +51.3 (*c* 0.32, $CHCl_3$).

(2*R*,2'*R*,4'*S*)-7-chloro-4,6-dimethoxy-2',7''-dimethyl-4'',5''-dihydro-3*H*,3''*H*-dispiro[benzofuran-2,1'-cyclohexane-4',1''-pyrano[4,3-*b*]indol]-3-one **7d**

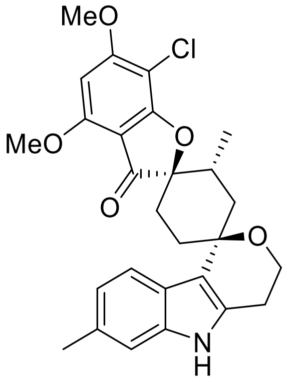

7d

40.9 mg, 85%. **^{1}H NMR** (400 MHz, Chloroform-*d*) δ 8.00 (d, *J* = 8.1 Hz, 1H), 7.74 (s, 1H), 7.08 (dd, *J* = 1.6, 0.8 Hz, 1H), 7.02 – 6.94 (m, 1H), 4.05 – 3.96 (m, 8H), 3.01 – 2.69 (m, 5H), 2.57 – 2.46 (m, 1H), 2.44 (s, 3H), 1.93 (ddt, *J* = 14.5, 4.5, 2.6 Hz, 1H), 1.81 (dddd, *J* = 26.8, 12.8, 4.1, 2.5 Hz, 2H), 0.82 (d, *J* = 6.7 Hz, 3H) ppm. **^{13}C NMR** (101 MHz, cdcl$_3$) δ 199.1, 168.4, 163.8, 157.4, 135.9, 131.0, 130.0, 122.5, 121.5, 119.5, 115.1, 110.5, 106.0, 97.1, 93.0, 88.6, 73.9, 58.6, 56.8, 56.3, 38.4, 33.5, 29.5, 28.5, 24.4, 21.6, 14.7 ppm. **HRMS**-ESI (*m/z*): [M + H]$^+$ calculated for $C_{27}H_{28}ClNO_5$ [M+H]$^+$: 482.1656 and 484.1627, found 482.1653 and 484.1624. **$[\alpha]_D^{20}$** +37.6 (*c* 0.11, $CHCl_3$).

(2*R*,2'*R*,4'*S*)-7-chloro-4,6-dimethoxy-2',6''-dimethyl-4'',5''-dihydro-3*H*,3''*H*-dispiro[benzofuran-2,1'-cyclohexane-4',1''-pyrano[4,3-*b*]indol]-3-one **7e**

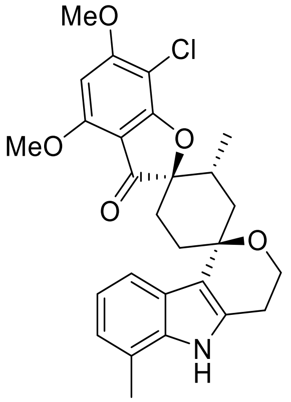

7e

42.1 mg, 87%. **^{1}H NMR** (400 MHz, Chloroform-*d*) δ 8.01 (d, *J* = 7.9 Hz, 1H), 7.79 (s, 1H), 7.10 (t, *J* = 7.6 Hz, 1H), 6.96 (dd, *J* = 7.2, 1.1 Hz, 1H), 6.11 (s, 1H), 4.07 – 3.97 (m, 8H [2 OMe]), 3.03 – 2.70 (m, 5H), 2.57 – 2.48 (m, 1H), 2.46 (s, 3H), 1.94 (ddt, *J* = 14.5, 4.5, 2.6 Hz, 1H), 1.82 (dddd, *J* = 28.0, 12.9, 4.1, 2.6 Hz, 2H), 0.82 (d, *J* = 6.8 Hz, 3H) ppm. **^{13}C NMR** (101 MHz, cdcl$_3$) δ 199.1, 168.4, 163.8, 157.4, 134.9, 130.4, 124.2, 122.0, 120.2, 119.5, 117.8, 115.8, 106.0, 97.1, 93.0, 88.6, 74.0, 58.6, 56.8, 56.3, 38.4, 33.5, 29.4, 28.5, 24.5, 14.7 ppm. **HRMS**-ESI (*m/z*): [M + H]$^+$ calculated for $C_{27}H_{28}ClNO_5$ [M+H]$^+$: 482.1656 and 484.1627, found 482.1654 and 484.1623. **$[\alpha]_D^{20}$** +36.9 (*c* 0.11, CHCl$_3$).

(2*R*,2'*R*,4'*S*)-7-chloro-8''-fluoro-4,6-dimethoxy-2'-methyl-4'',5''-dihydro-3*H*,3''*H*-dispiro[benzofuran-2,1'-cyclohexane-4',1''-pyrano[4,3-*b*]indol]-3-one **7f**

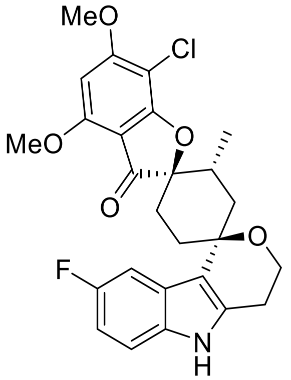

7f

38.8 mg, 80%. **^{1}H NMR** (700 MHz, Chloroform-*d*) δ 7.83 (dd, J = 9.9, 2.4 Hz, 2H), 7.20 (dd, J = 8.7, 4.3 Hz, 1H), 6.88 (td, J = 9.0, 2.4 Hz, 1H), 6.11 (s, 1H), 4.03 (s, 3H), 4.02 (s, 3H), 3.99 (s, 2H), 2.90 (td, J = 14.3, 4.2 Hz, 1H), 2.87 – 2.80 (m, 2H), 2.80 – 2.71 (m, 2H), 2.50 (td, J = 13.6, 4.2 Hz, 1H), 1.92 (d, J = 14.7 Hz, 1H), 1.84 (dt, J = 14.2, 3.5 Hz, 1H), 1.78 (dt, J = 12.9, 3.2 Hz, 1H), 0.83 (d, J = 6.8 Hz, 3H) ppm. **^{13}C NMR** (176 MHz, Chloroform-*d*) δ 199.4, 164.2, 158.2 (d, J = 235.1 Hz), 157.8, 133.0, 132.3, 125.4 (d, J = 10.0 Hz), 116.0 (d, J = 4.3 Hz), 111.3 (d, J = 9.7 Hz), 109.8 (d, J = 26.3 Hz), 106.4, 105.4 (d, J = 24.1 Hz), 97.5, 93.2, 89.0, 74.2, 58.9, 57.2, 56.7, 38.5, 33.9, 29.6, 28.9, 24.9, 15.0 ppm. **^{19}F NMR** (470 MHz, $CDCl_3$) δ -123.17. **HRMS**-ESI (*m/z*): $[M + H]^+$ calculated for $C_{26}H_{25}ClFNO_5$ $[M+H]^+$: 486.1405 and 488.1376, found 486.1401 and 488.1372. $[\alpha]_D^{20}$ +37.3 (*c* 0.12, $CHCl_3$).

(2*R*,2'*R*,4'*S*)-7-chloro-8''-chloro-4,6-dimethoxy-2'-methyl-4'',5''-dihydro-3*H*,3''*H*-dispiro[benzofuran-2,1'-cyclohexane-4',1''-pyrano[4,3-*b*]indol]-3-one **7g**

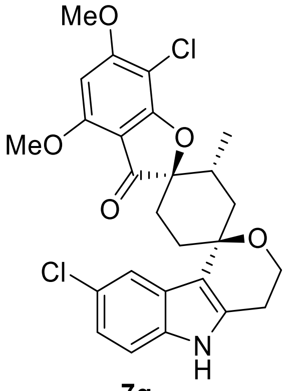

7g

39.6 mg, 79%. **^{1}H NMR** (700 MHz, Chloroform-*d*) δ 8.10 – 8.07 (m, 1H), 7.87 (s, 1H), 7.21 (d, J = 8.5 Hz, 1H), 7.10 (dd, J = 8.5, 1.9 Hz, 1H), 6.11 (s, 1H), 4.02 (d, J = 4.4 Hz, 8H), 2.89 (td, J = 14.3, 4.2 Hz, 1H), 2.87 – 2.81 (m, 2H), 2.80 – 2.71 (m, 2H), 2.49 (td, J = 13.6, 4.2 Hz, 1H), 1.92 (d, J = 14.8 Hz, 1H), 1.84 (dt, J = 14.2, 3.7 Hz, 1H), 1.81 – 1.76 (m, 1H), 0.83 (d, J = 6.8 Hz, 3H) ppm. **^{13}C NMR** (176 MHz, Chloroform-*d*) δ 199.3, 164.1, 157.7, 134.0, 132.5, 126.0, 125.8, 121.8, 119.3, 115.5, 111.6, 93.0, 88.8, 74.0, 58.6, 57.0, 56.5, 38.5, 33.7, 29.6, 28.6, 24.6, 14.8 ppm. **HRMS**-ESI (*m/z*): [M + H]$^+$ calculated for $C_{26}H_{25}Cl_2NO_5$ [M+H]$^+$: 502.1110 and 504.1080, found 502.1108 and 504.1079. $[\alpha]_D^{20}$ +34.0 (*c* 0.24, $CHCl_3$).

(2*R*,2'*R*,4'*S*)-7-chloro-8''-methoxy-4,6-dimethoxy-2'-methyl-4'',5''-dihydro-3*H*,3''*H*-dispiro[benzofuran-2,1'-cyclohexane-4',1''-pyrano[4,3-*b*]indol]-3-one **7h**

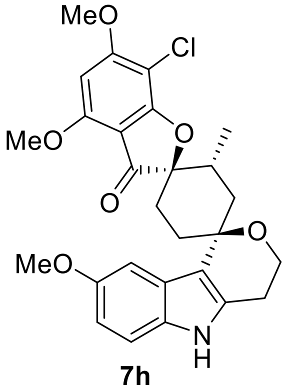

7h

45.3 mg, 91%. **^{1}H NMR** (700 MHz, Chloroform-*d*) δ 7.71 (s, 1H), 7.66 (d, *J* = 2.3 Hz, 1H), 7.19 (d, *J* = 8.7 Hz, 1H), 6.81 (dd, *J* = 8.7, 2.4 Hz, 1H), 6.09 (s, 1H), 4.03 (s, 3H), 4.01 (s, 4H), 4.00 (s, 3H), 2.95 (td, *J* = 14.4, 4.2 Hz, 1H), 2.91 – 2.86 (m, 1H), 2.86 – 2.80 (m, 1H), 2.79 – 2.72 (m, 2H), 2.51 (td, *J* = 13.7, 4.3 Hz, 1H), 1.93 (d, *J* = 14.8 Hz, 1H), 1.86 (dt, *J* = 14.3, 3.3 Hz, 1H), 1.81 – 1.76 (m, 1H), 0.83 (d, *J* = 6.8 Hz, 3H) ppm. **^{13}C NMR** (176 MHz, Chloroform-*d*) δ 199.4, 168.6, 164.0, 157.6, 154.4, 131.8, 130.7, 125.3, 115.4, 111.5, 111.3, 106.3, 102.2, 97.4, 93.2, 88.8, 74.1, 58.8, 56.4, 38.3, 33.7, 29.4, 28.7, 27.1, 24.7, 14.9 ppm. **HRMS**-ESI (*m/z*): $[M + H]^+$ calculated for $C_{27}H_{28}ClNO_6$ $[M+H]^+$: 498.1605 and 500.1576, found 498.1602 and 500.1573. $[\alpha]_D^{20}$ +30.1 (*c* 0.42, $CHCl_3$).

(2*R*,2'*R*,4'*S*)-7-chloro-8''-benzoyl-4,6-dimethoxy-2'-methyl-4'',5''-dihydro-3*H*,3''*H*-dispiro[benzofuran-2,1'-cyclohexane-4',1''-pyrano[4,3-*b*]indol]-3-one **7i**

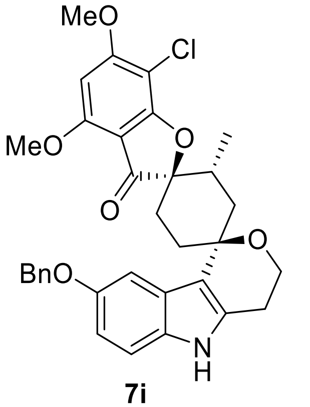

7i

54.5 mg, 95%. **^{1}H NMR** (500 MHz, Chloroform-*d*) *δ* 7.71 (s, 1H), 7.60 (d, *J* = 7.1 Hz, 2H), 7.37 (t, *J* = 7.5 Hz, 2H), 7.29 (t, *J* = 7.4 Hz, 1H), 7.19 (d, *J* = 8.7 Hz, 1H), 6.87 (dd, *J* = 8.7, 2.4 Hz, 1H), 6.09 (s, 1H), 5.39 – 5.28 (m, 2H), 3.99 (d, *J* = 18.4 Hz, 8H), 2.96 (td, *J* = 14.3, 4.2 Hz, 1H), 2.91 – 2.80 (m, 2H), 2.80 – 2.70 (m, 2H), 2.50 (td, *J* = 13.7, 4.3 Hz, 1H), 1.93 (d, *J* = 14.8 Hz, 1H), 1.85 (dt, *J* = 14.2, 3.2 Hz, 1H), 1.82 – 1.76 (m, 1H), 0.83 (d, *J* = 6.8 Hz, 3H) ppm. **^{13}C NMR** (126 MHz, Chloroform-*d*) *δ* 168.8, 164.2, 157.9, 153.7, 138.5, 132.0, 131.0, 128.6, 128.4, 127.9, 125.5, 115.7, 112.4, 111.6, 103.6, 97.5, 93.4, 89.0, 74.3, 71.1, 59.0, 57.2, 56.6, 38.6, 34.0, 29.6, 29.0, 25.0, 15.1 ppm. **HRMS**-ESI (*m/z*): $[M + H]^+$ calculated for $C_{33}H_{32}ClNO_6$ $[M+H]^+$: 574.1918 and 575.1952, found 574.1916 and 575.1950. $[\alpha]_D^{20}$ +26.8 (*c* 0.32, $CHCl_3$).

methyl-(2*R*,2'*R*,4'*S*)-7-chloro-4,6-dimethoxy-2'-methyl-3-oxo-4'',5''-dihydro-3*H*,3''*H*-dispiro[benzofuran-2,1'-cyclohexane-4',1''-pyrano[4,3-*b*]indole]-8''-carboxylate **7j**

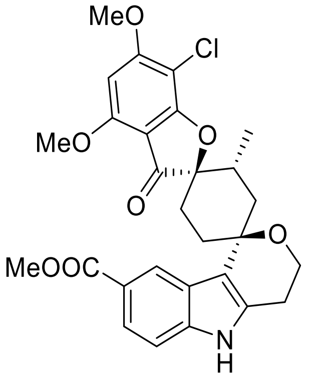

7j

42.6 mg, 81%. **^{1}H NMR** (700 MHz, Chloroform-*d*) δ 8.93 (s, 1H), 8.06 (s, 1H), 7.89 (dd, *J* = 8.5, 1.5 Hz, 1H), 7.32 (d, *J* = 8.5 Hz, 1H), 6.10 (s, 1H), 4.01 (d, *J* = 1.0 Hz, 9H), 2.99 (td, *J* = 14.3, 4.2 Hz, 1H), 2.94 – 2.88 (m, 1H), 2.88 – 2.78 (m, 2H), 2.78 – 2.71 (m, 1H), 2.50 (td, *J* = 13.6, 4.3 Hz, 1H), 1.93 (d, *J* = 14.8 Hz, 1H), 1.87 (dt, *J* = 14.3, 3.8 Hz, 1H), 1.80 (dt, *J* = 12.9, 3.9 Hz, 1H), 1.26 (s, 1H), 0.84 (d, *J* = 6.8 Hz, 3H) ppm. **^{13}C NMR** (176 MHz, Chloroform-*d*) δ 199.1, 168.5, 164.0, 157.8, 138.3, 132.4, 124.6, 123.2, 122.6, 122.2, 116.9, 110.4, 106.4, 93.0, 88.9, 74.1, 57.0, 56.4, 52.3, 38.6, 33.8, 30.0, 28.8, 24.6, 14.8 ppm. **HRMS**-ESI (*m/z*): $[M + H]^+$ calculated for $C_{28}H_{28}ClNO_7$ $[M+H]^+$: 526.1554 and 527.1525, found 526.1553 and 527.1522. $[\alpha]_D^{20}$ +32.6 (*c* 0.14, $CHCl_3$).

(2*R*,2'*R*,4'*S*)-7-chloro-8''-hydroxy-4,6-dimethoxy-2'-methyl-4'',5''-dihydro-3*H*,3''*H*-dispiro[benzofuran-2,1'-cyclohexane-4',1''-pyrano[4,3-*b*]indol]-3-one **7k**

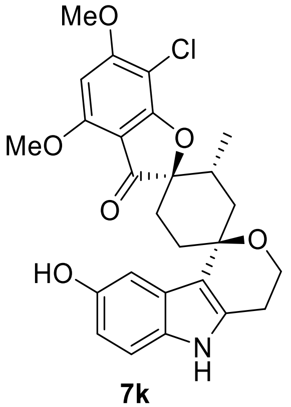

7k

40.1 mg, 83%. **^{1}H NMR** (700 MHz, Methanol-d4) δ 7.49 (d, J = 2.3 Hz, 1H), 7.13 (d, J = 8.6 Hz, 1H), 6.62 (dd, J = 8.6, 2.3 Hz, 1H), 6.41 (s, 1H), 4.06 (s, 3H), 4.04 (s, 3H), 2.90 (td, J = 14.4, 4.2 Hz, 1H), 2.85 – 2.74 (m, 3H), 2.67 – 2.60 (m, 1H), 2.42 (td, J = 13.7, 4.2 Hz, 1H), 2.05 – 2.00 (m, 1H), 1.90 (d, J = 14.4 Hz, 1H), 1.84 (d, J = 13.5 Hz, 1H), 1.76 (d, J = 11.7 Hz, 1H), 1.38 – 1.30 (m, 3H), 0.79 (d, J = 6.8 Hz, 3H) ppm. **^{13}C NMR** (176 MHz, Methanol-d4) δ 200.9, 169.7, 166.3, 159.5, 151.3, 112.2, 111.0, 104.3, 94.2, 90.7, 75.4, 60.0, 57.7, 56.9, 49.4, 39.3, 35.1, 30.6, 29.8, 25.4, 15.2 ppm. **HRMS**-ESI (*m/z*): [M + H]$^+$ calculated for $C_{26}H_{26}ClNO_6$ [M+H]$^+$: 484.1449 and 486.1419, found 484.1447 and 486.1416. $[\alpha]_D^{20}$ +106.6 (*c* 0.15, $CHCl_3$).

(2*R*,2'*R*,4'*S*)-7-chloro-4,6-dimethoxy-2',5'',8''-trimethyl-4'',5''-dihydro-3*H*,3''*H*-dispiro[benzofuran-2,1'-cyclohexane-4',1''-pyrano[4,3-*b*]indol]-3-one **7l**

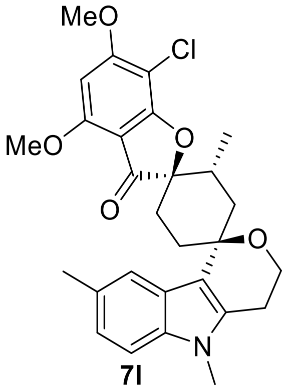

7l

46.6 mg, 94%. **¹H NMR** (400 MHz, Chloroform-*d*) δ 7.90 (s, 1H), 7.17 (d, *J* = 8.3 Hz, 1H), 7.01 (d, *J* = 8.3 Hz, 1H), 6.11 (s, 1H), 4.02 (dd, *J* = 4.0, 0.9 Hz, 7H), 3.60 (s, 2H), 3.04 – 2.85 (m, 2H), 2.86 – 2.69 (m, 3H), 2.55 (s, 3H), 2.54 – 2.45 (m, 1H), 1.92 (ddt, *J* = 14.5, 4.0, 2.6 Hz, 1H), 1.89 – 1.74 (m, 2H), 0.83 (d, *J* = 6.8 Hz, 3H) ppm. **¹³C NMR** (101 MHz, cdcl$_3$) δ 199.4, 168.5, 163.9, 157.4, 135.0, 132.5, 128.6, 124.5, 122.3, 119.4, 113.9, 108.3, 106.0, 97.2, 93.1, 88.5, 74.0, 58.6, 56.8, 56.2, 38.5, 33.5, 29.6, 28.9, 28.6, 23.3, 21.7, 14.7 ppm. **HRMS**-ESI (*m/z*): $[M + H]^+$ calculated for $C_{28}H_{30}ClNO_5$ $[M+H]^+$: 496.1813 and 498.1783, found 496.1810 and 498.1781. $[\alpha]_D^{20}$ +17.4 (*c* 0.12, $CHCl_3$).

(2*R*,2'*R*,4'*S*)-5''-benzyl-7-chloro-4,6-dimethoxy-2',8''-dimethyl-4'',5''-dihydro-3*H*,3''*H*-dispiro[benzofuran-2,1'-cyclohexane-4',1''-pyrano[4,3-*b*]indol]-3-one **7m**

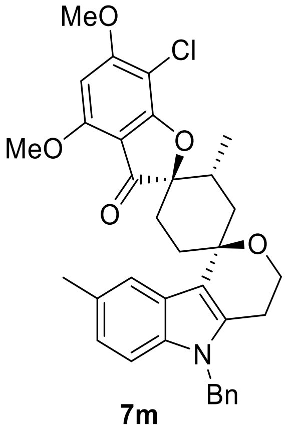

7m

53.1 mg, 93%. **^{1}H NMR** (700 MHz, Chloroform-*d*) *δ* 7.97 – 7.88 (m, 1H), 7.27 – 7.24 (m, 3H), 7.23 – 7.19 (m, 1H), 7.13 (d, *J* = 8.3 Hz, 1H), 7.03 – 6.98 (m, 2H), 6.96 (dd, *J* = 8.4, 1.6 Hz, 1H), 6.11 (s, 1H), 5.24 (d, *J* = 2.9 Hz, 2H), 4.03 (s, 3H), 4.02 (s, 3H), 4.01 (s, 2H), 3.00 (td, *J* = 14.3, 4.2 Hz, 1H), 2.96 – 2.85 (m, 1H), 2.79 – 2.64 (m, 2H), 2.55 (s, 3H), 2.54 – 2.49 (m, 1H), 1.96 (ddd, *J* = 11.9, 4.3, 2.0 Hz, 1H), 1.88 (ddd, *J* = 14.3, 4.1, 2.6 Hz, 1H), 1.80 (ddd, *J* = 12.9, 4.1, 2.6 Hz, 1H), 0.84 (d, *J* = 6.9 Hz, 3H) ppm. **^{13}C NMR** (176 MHz, Chloroform-*d*) *δ* 199.7, 168.8, 164.2, 157.8, 138.2, 135.2, 132.8, 129.3, 129.1, 127.6, 126.5, 125.2, 122.9, 119.9, 115.0, 109.2, 106.4, 97.6, 93.5, 88.9, 74.4, 58.9, 57.2, 56.6, 46.6, 38.9, 33.9, 29.9, 28.9, 23.8, 22.1, 15.1 ppm. **HRMS**-ESI (*m/z*): $[M + H]^+$ calculated for $C_{34}H_{34}ClNO_5$ $[M+H]^+$: 572.2126 and 573.2159, found 572.2123 and 573.2155. $[\alpha]_D^{20}$ +27.5 (*c* 0.24, $CHCl_3$).

(2*R*,2'*R*,4'*S*)-7-chloro-4-hydroxy-6-methoxy-2',8''-dimethyl-4'',5''-dihydro-3*H*,3''*H*-dispiro[benzofuran-2,1'-cyclohexane-4',1''-pyrano[4,3-*b*]indol]-3-one **7n**

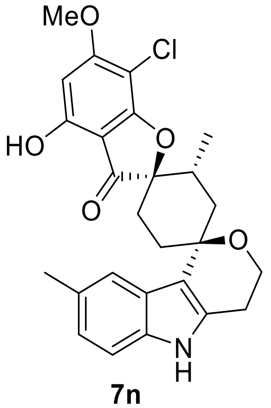

7n

42.1 mg, 90%. **^{1}H NMR** (400 MHz, Chloroform-*d*) δ 8.11 (s, 1H), 7.73 (dd, J = 1.7, 0.9 Hz, 1H), 7.64 (s, 1H), 7.11 (dd, J = 8.2, 0.7 Hz, 1H), 6.96 – 6.81 (m, 1H), 6.05 (s, 1H), 3.98 – 3.87 (m, 2H), 3.86 (s, 3H), 2.86 – 2.63 (m, 5H), 2.51 – 2.36 (m, 4H), 1.95 (s, 1H), 1.92 – 1.82 (m, 1H), 1.78 (dd, J = 10.1, 2.6 Hz, 1H), 1.71 (ddd, J = 12.9, 4.1, 2.7 Hz, 1H), 0.79 – 0.69 (m, 3H) ppm. **^{13}C NMR** (151 MHz, $CDCl_3$) δ 202.7, 166.3, 164.9, 156.2, 133.9, 131.1, 129.1, 125.0, 122.9, 119.2, 114.8, 110.5, 104.9, 96.2, 94.4, 92.8, 73.9, 58.8, 57.1, 38.6, 33.6, 29.7, 28.6, 24.6, 21.9, 14.9 ppm. **HRMS**-ESI (*m/z*): $[M + H]^+$ calculated for $C_{26}H_{26}ClNO_5$ $[M+H]^+$: 468.1500 and 470.1470, found 468.1497 and 470.1468. $[\alpha]_D^{20}$ +23.0 (*c* 0.14, $CHCl_3$).

(2*R*,2'*R*,4'*S*)-4-(allyloxy)-7-chloro-6-methoxy-2',8''-dimethyl-4'',5''-dihydro-3*H*,3''*H*-dispiro[benzofuran-2,1'-cyclohexane-4',1''-pyrano[4,3-*b*]indol]-3-one **7o**

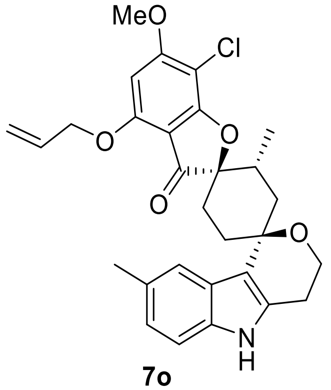

7o

42.1 mg, 83%. **^{1}H NMR** (400 MHz, Chloroform-*d*) δ 7.91 (s, 1H), 7.85 (s, 1H), 7.17 (d, *J* = 8.2 Hz, 1H), 6.97 (dd, *J* = 8.3, 1.4 Hz, 1H), 6.22 – 6.05 (m, 2H), 5.55 (dp, *J* = 17.3, 1.5 Hz, 1H), 5.40 (dp, *J* = 10.6, 1.4 Hz, 1H), 4.78 (dt, *J* = 5.2, 1.4 Hz, 2H), 4.00 (q, *J* = 5.5 Hz, 2H), 3.97 (s, 4H), 3.05 – 2.86 (m, 2H), 2.78 (qt, *J* = 15.6, 5.3 Hz, 3H), 2.53 (s, 4H), 2.00 – 1.74 (m, 4H), 0.85 (d, *J* = 6.7 Hz, 3H) ppm. **^{13}C NMR** (101 MHz, cdcl$_3$) δ 198.9, 168.4, 163.6, 156.5, 133.8, 132.3, 130.9, 128.9, 125.0, 122.7, 119.4, 118.6, 114.8, 110.3, 106.4, 97.2, 92.9, 90.4, 74.0, 70.2, 58.6, 56.7, 38.4, 33.6, 29.6, 28.6, 24.5, 21.7, 14.7 ppm. **HRMS**-ESI (*m/z*): $[M + H]^+$ calculated for $C_{29}H_{30}ClNO_5$ $[M+H]^+$: 508.1813 and 510.1783, found 508.1811 and 510.1782. $[\alpha]_D^{20}$ +44 (*c* 0.1, $CHCl_3$).

(2*R*,2'*R*,4'*S*)-4-(benzyloxy)-7-chloro-6-methoxy-2',8''-dimethyl-4'',5''-dihydro-3*H*,3''*H*-dispiro[benzofuran-2,1'-cyclohexane-4',1''-pyrano[4,3-*b*]indol]-3-one **7p**

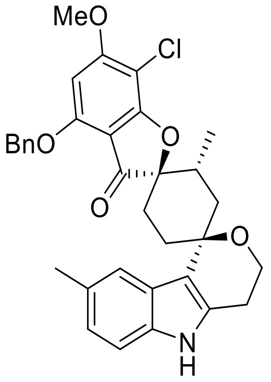

7p

47.4 mg, 85%. **^{1}H NMR** (400 MHz, Chloroform-*d*) δ 7.99 (s, 1H), 7.81 (s, 1H), 7.60 – 7.53 (m, 2H), 7.43 (td, *J* = 6.9, 6.0, 1.2 Hz, 2H), 7.35 (t, *J* = 7.6 Hz, 1H), 7.19 (d, *J* = 8.2 Hz, 1H), 6.99 (dd, *J* = 8.3, 1.6 Hz, 1H), 6.13 (s, 1H), 5.34 (s, 2H), 4.02 (hept, *J* = 5.8 Hz, 2H), 3.89 (s, 3H), 3.08 – 2.89 (m, 2H), 2.88 – 2.70 (m, 3H), 2.56 (s, 3H), 2.55 – 2.47 (m, 1H), 2.01 – 1.77 (m, 3H), 0.87 (d, *J* = 6.7 Hz, 3H) ppm. **^{13}C NMR** (101 MHz, $CDCl_3$) δ 198.6, 168.3, 163.4, 156.3, 136.0, 133.8, 130.9, 128.9, 128.8, 128.7, 128.1, 126.8, 126.8, 125.0, 122.7, 119.5, 114.9, 110.2, 106.7, 97.4, 92.9, 91.2, 74.0, 71.2, 58.6, 56.7, 38.4, 33.7, 29.6, 28.6, 24.5, 21.7, 14.8 ppm. **HRMS**-ESI (*m/z*): [M + H]$^+$ calculated for $C_{33}H_{32}ClNO_5$ [M+H]$^+$: 558.1969, found 558.1967. **$[\alpha]_D^{20}$** +38.7 (*c* 0.11, $CHCl_3$).

(2*R*,2'*R*)-7-chloro-4,6-dimethoxy-2'-methyl-3'',4''-dihydro-3*H*-dispiro[benzofuran-2,1'-cyclohexane-4',1''-pyrano[4,3-*b*]benzofuran]-3-one **8**

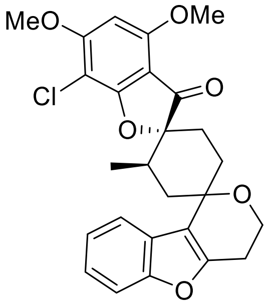

8

41.7 mg, 89%. **^{1}H NMR** (400 MHz, Chloroform-*d*) δ 8.13 – 8.00 (m, 1H), 7.47 – 7.38 (m, 1H), 7.30 – 7.24 (m, 2H), 6.11 (s, 1H), 4.10 – 3.93 (m, 9H), 2.95 – 2.66 (m, 5H), 2.49 (ddd, *J* = 14.1, 13.0, 4.2 Hz, 1H), 2.00 – 1.73 (m, 3H), 0.83 (d, *J* = 6.5 Hz, 3H) ppm. **^{13}C NMR** (126 MHz, Chloroform-*d*) δ 198.7, 168.5, 163.7, 157.2, 156.0, 154.3, 128.1, 123.7, 121.9, 118.7, 111.5, 110.0, 105.6, 92.1, 88.6, 73.0, 59.8, 56.5, 56.1, 36.8, 33.2, 28.3, 28.0, 26.8, 22.6, 14.8 ppm**.** **HRMS**-ESI (*m/z*): $[M + H]^+$ calculated for $C_{26}H_{25}O_6Cl$ $[M+H]^+$: 469.1340 and 471.1310, found 469.1339 and 471.1307. $[\alpha]_D^{20}$ +38.6 (*c* 0.18, $CHCl_3$).

(2R,2'R)-7-chloro-4,6-dimethoxy-2'-methyl-3'',4''-dihydro-3H-dispiro[benzofuran-2,1'-cyclohexane-4',1''-benzo[4,5]thieno[2,3-c]pyran]-3-one **9**

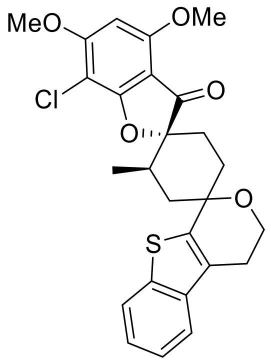

41.2 mg, 85%. **^{1}H NMR** (500 MHz, Chloroform-*d*) δ 7.83 (d, *J* = 7.9 Hz, 1H), 7.60 (d, *J* = 7.7 Hz, 1H), 7.36 (t, *J* = 7.5 Hz, 1H), 7.30 (t, *J* = 7.5 Hz, 1H), 6.09 (s, 1H), 4.13 – 4.03 (m, 2H), 4.01 (s, 3H), 3.99 (s, 3H), 2.92 – 2.80 (m, 2H), 2.77 – 2.68 (m, 1H), 2.65 (dd, *J* = 14.2, 4.0 Hz, 1H), 2.59 (dd, *J* = 14.1, 12.9 Hz, 1H), 2.47 (td, *J* = 13.5, 4.0 Hz, 1H), 2.14 – 2.07 (m, 1H), 2.05 – 2.00 (m, 1H), 1.78 (ddd, *J* = 13.0, 4.0, 2.7 Hz, 1H), 0.82 (d, *J* = 6.7 Hz, 3H) ppm. **^{13}C NMR** (126 MHz, $CDCl_3$) δ 198.6, 163.8, 157.4, 142.8, 138.7, 138.6, 127.1, 124.0, 123.9, 122.5, 120.7, 105.8, 97.0, 92.1, 88.6, 74.2, 59.0, 56.7, 56.1, 40.7, 33.6, 31.9, 28.5, 24.7, 14.5 ppm. **HRMS**-ESI (*m/z*): [M + H]$^+$ calculated for $C_{26}H_{25}O_5SCl$ [M+H]$^+$: 485.1111 and 487.1082, found 485.1115 and 487.1083. **$[\alpha]_D^{20}$** +34.5 (*c* 0.12, $CHCl_3$).

(2R,2'R)-7-chloro-4,6-dimethoxy-2'-methyl-4'',9''-dihydro-3H,3''H-dispiro[benzofuran-2,1'-cyclohexane-4',1''-pyrano[3,4-b]indol]-3-one **10**

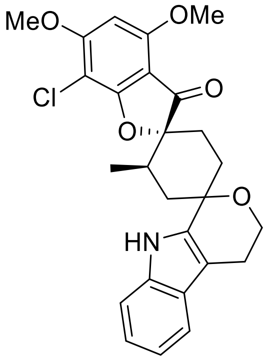

43.9 mg, 94%. **^{1}H NMR** (700 MHz, Chloroform-*d*) δ 8.18 (s, 1H), 7.49 (d, *J* = 7.8 Hz, 1H), 7.32 (d, *J* = 8.0 Hz, 1H), 7.16 (t, *J* = 7.6 Hz, 1H), 7.10 (t, *J* = 7.4 Hz, 1H), 6.09 (s, 1H), 4.06 – 3.98 (m, 5H), 3.97 (d, *J* = 1.3 Hz, 3H), 2.87 – 2.78 (m, 2H), 2.75 – 2.69 (m, 1H), 2.63 (td, *J* = 14.2, 4.2 Hz, 1H), 2.55 (t, *J* = 13.6 Hz, 1H), 2.49 (td, *J* = 13.5, 4.2 Hz, 1H), 2.06 (d, *J* = 14.8 Hz, 2H), 1.99 (d, *J* = 14.3 Hz, 1H), 1.80 (d, *J* = 13.0 Hz, 1H), 0.83 (d, *J* = 6.9 Hz, 3H) ppm. **^{13}C NMR** (176 MHz, $CDCl_3$) δ 199.4, 164.3, 157.7, 138.0, 135.8, 126.9, 121.8, 119.5, 118.2, 111.1, 107.2, 106.0, 97.3, 92.6, 88.9, 72.3, 60.2, 57.0, 56.3, 38.8, 33.8, 30.0, 28.7, 22.5, 14.8 ppm. **HRMS**-ESI (*m/z*): $[M + H]^+$ calculated for $C_{26}H_{27}ClNO_5$ $[M+H]^+$: 468.1572, found 468.1568. $[\alpha]_D^{20}$ +28.5 (*c* 0.63, $CHCl_3$).

(2R,2'R)-7-chloro-4,6-dimethoxy-2',10''-dimethyl-3'',4''-dihydro-3H-dispiro[benzofuran-2,1'-cyclohexane-4',1''-[1,4]oxazino[4,3-a]indol]-3-one **11**

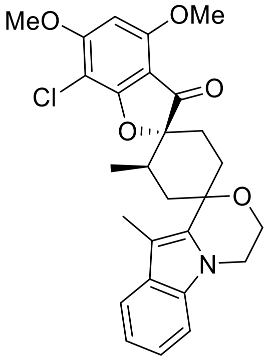

37.5 mg, 78%. **^{1}H NMR** (700 MHz, Chloroform-*d*) δ 7.49 (d, *J* = 7.8 Hz, 1H), 7.17 (d, *J* = 8.1 Hz, 1H), 7.11 (ddd, *J* = 8.0, 6.9, 1.2 Hz, 1H), 7.05 (ddd, *J* = 7.9, 6.9, 1.0 Hz, 1H), 6.03 (s, 1H), 4.06 – 3.98 (m, 4H), 3.94 (d, *J* = 9.0 Hz, 6H), 2.80 (td, *J* = 14.3, 4.1 Hz, 1H), 2.73 (dd, *J* = 14.1, 12.9 Hz, 1H), 2.66 (dtd, *J* = 12.9, 6.6, 3.8 Hz, 1H), 2.51 (s, 3H), 2.40 (ddd, *J* = 13.9, 13.0, 4.3 Hz, 1H), 2.03 – 1.85 (m, 2H), 1.70 (ddd, *J* = 13.0, 4.1, 2.6 Hz, 1H), 0.76 (d, *J* = 6.7 Hz, 3H) ppm. **^{13}C NMR** (176 MHz, $CDCl_3$) δ 199.0, 168.4, 164.0, 157.6, 134.8, 134.0, 128.7, 121.0, 119.2, 118.4, 108.2, 105.9, 104.6, 97.1, 92.5, 88.7, 74.5, 58.4, 56.8, 56.3, 41.7, 37.3, 33.1, 28.7, 28.1, 26.9, 14.6, 9.6 ppm. **HRMS**-ESI (*m/z*): $[M + H]^+$ calculated for $C_{27}H_{28}ClNO_5$ $[M+H]^+$: 482.1656 and 484.1627, found 482.1655 and 484.1627. $[\alpha]_D^{20}$ +33.8 (*c* 0.2, $CHCl_3$).

(2R,2'R)-7-chloro-4,6-dimethoxy-2'-methyl-6'',7''-dihydro-3H-dispiro[benzofuran-2,1'-cyclohexane-4',4''-thieno[3,2-c]pyran]-3-one **12**

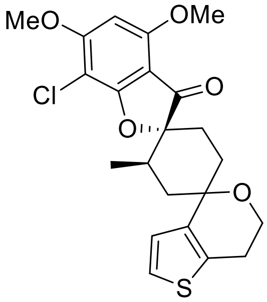

The thiophene derivative (1.0 eq.) was dissolved in anhydrous dichloromethane (3 mL) and subsequently, TfOH·SiO_2 (6.5 mol%) and cyclic ketone (1.5 eq.) were added. After flushing the reaction tube with argon, the reaction mixture was heated to 50 °C for 2 h. The reaction mixture was filtered, rinsed with ethyl acetate and concentrated *in vacuo*. The desired product was purified via flash column chromatography (EtOAc in CycHex (0-50%)).

36.9 mg, 85%. **^{1}H NMR** (500 MHz, Chloroform-*d*) δ 7.08 (d, *J* = 5.1 Hz, 1H), 6.99 (d, *J* = 5.2 Hz, 1H), 6.08 (s, 1H), 4.01 (s, 3H), 3.99 (s, 3H), 3.97 – 3.90 (m, 2H), 2.91 – 2.77 (m, 2H), 2.65 (ddd, *J* = 13.1, 6.7, 4.2 Hz, 1H), 2.56 (td, *J* = 14.1, 4.0 Hz, 1H), 2.49 (dd, *J* = 14.2, 12.9 Hz, 1H), 2.41 (ddd, *J* = 14.1, 12.8, 4.0 Hz, 1H), 1.95 – 1.88 (m, 1H), 1.84 (ddd, *J* = 14.2, 4.1, 2.6 Hz, 1H), 1.73 (ddd, *J* = 12.8, 4.0, 2.7 Hz, 1H), 0.79 (d, *J* = 6.8 Hz, 3H) ppm. **^{13}C NMR** (126 MHz, $CDCl_3$) δ 199.1, 164.0, 157.6, 141.0, 132.4, 124.8, 122.3, 106.0, 97.2, 92.7, 88.7, 74.9, 59.4, 56.9, 56.3, 39.4, 33.8, 30.6, 28.7, 25.9, 14.8 ppm. **HRMS**-ESI (*m/z*): $[M + H]^+$ calculated for $C_{22}H_{23}ClO_5S$ $[M+H]^+$: 435.0955 and 437.0925, found 435.0945 and 437.0934. $[\alpha]_D^{20}$ +30.1 (*c* 0.19, $CHCl_3$).

(2R,2'R)-7-chloro-4,6-dimethoxy-2'-methyl-4'',5''-dihydro-3H-dispiro[benzofuran-2,1'-cyclohexane-4',7''-thieno[2,3-c]pyran]-3-one **13**

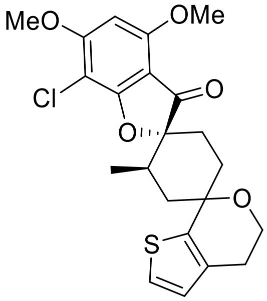

The thiophene derivative (1.0 eq.) was dissolved in anhydrous dichloromethane (3 mL) and subsequently, TfOH·SiO_2 (6.5 mol%) and cyclic ketone (1.5 eq.) were added. After flushing the reaction tube with argon, the reaction mixture was heated to 50 °C for 2 h. The reaction mixture was filtered, rinsed with ethyl acetate and concentrated *in vacuo*. The desired product was purified via flash column chromatography (EtOAc in CycHex (0-50%)).

30.0 mg, 69%. **^{1}H NMR** (500 MHz, Chloroform-*d*) δ 7.08 (d, *J* = 5.0 Hz, 1H), 6.69 (d, *J* = 5.0 Hz, 1H), 6.02 (s, 1H), 3.94 (s, 3H), 3.91 (s, 3H), 3.91 – 3.82 (m, 2H), 2.71 – 2.55 (m, 3H), 2.50 (td, *J* = 14.1, 3.9 Hz, 1H), 2.46 – 2.40 (m, 1H), 2.36 (td, *J* = 14.1, 13.6, 3.9 Hz, 1H), 2.04 – 1.98 (m, 1H), 1.93 (ddd, *J* = 14.2, 4.1, 2.7 Hz, 1H), 1.67 (ddd, *J* = 12.9, 3.9, 2.7 Hz, 1H), 0.73 (d, *J* = 6.8 Hz, 3H) ppm. **^{13}C NMR** (126 MHz, $CDCl_3$) δ 198.7, 163.8, 157.4, 141.7, 132.6, 126.5, 122.6, 105.8, 97.0, 92.2, 88.5, 74.3, 59.2, 56.7, 56.1, 41.4, 33.6, 32.6, 28.6, 26.4, 14.5 ppm. **HRMS**-ESI (*m/z*): $[M + H]^+$ calculated for $C_{22}H_{23}ClO_5S$ $[M+H]^+$: 435.0955 and 437.0925, found 435.0945 and 437.0921. $[\alpha]_D^{20}$ +24.3 (*c* 0.14, $CHCl_3$).

(2R,2'R)-7-chloro-4,6-dimethoxy-2'-methyl-7'',8''-dihydro-3H-dispiro[benzofuran-2,1'-cyclohexane-4',5''-[1,3]dioxolo[4,5-g]isochromen]-3-one **14**

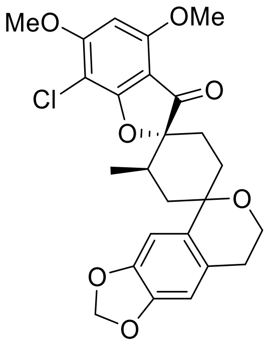

42.0 mg, 89%. ^{1}**H NMR** (700 MHz, Chloroform-*d*) δ 6.96 (s, 1H), 6.52 (s, 1H), 6.09 (s, 1H), 5.89 (s, 2H), 4.00 (s, 3H), 3.99 (s, 3H), 3.93 – 3.78 (m, 2H), 2.82 – 2.61 (m, 3H), 2.56 (td, *J* = 14.2, 4.1 Hz, 1H), 2.48 (dd, *J* = 14.3, 12.8 Hz, 1H), 2.45 – 2.39 (m, 1H), 1.92 (ddt, *J* = 14.3, 4.4, 2.6 Hz, 1H), 1.85 (ddd, *J* = 14.2, 4.2, 2.5 Hz, 1H), 1.72 (ddd, *J* = 12.8, 4.1, 2.6 Hz, 1H), 0.79 (d, *J* = 6.8 Hz, 3H). ^{13}**C NMR** (176 MHz, $CDCl_3$) δ 199.25, 168.58, 164.08, 157.66, 146.40, 146.06, 135.14, 126.78, 108.23, 106.26, 100.88, 97.32, 92.88, 88.82, 74.97, 59.15, 56.98, 56.35, 40.58, 34.04, 31.61, 29.79, 29.01, 14.86. **HRMS**-ESI (*m/z*): $[M + H]^+$ calculated for $C_{25}H_{25}O_7Cl$ $[M+H]^+$: 473.1289 and 475.1259, found 473.1288 and 475.1257. $[\alpha]_D^{20}$ +24.0 (*c* 0.1, $CHCl_3$).

General Procedure for the iso-oxa-Pictet-Spengler reaction on griseofulvin derivatives **5e-f**:

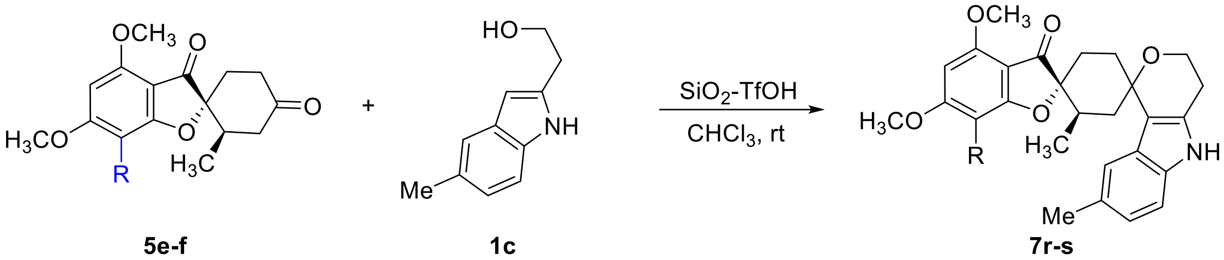

Griseofulvin ketone derivative **5e-f** (0.1 mmol) and 2-(5-methyl-1H-indol-2-yl)ethan-1-ol were added to an oven-dried reaction tube, followed by SiO_2-TfOH. 2 mL $CHCl_3$ was added in the solid mixture. The mixture was stirred at room temperature for 3 h (monitored by TLC until starting material was consumed). After completion of reaction, solvent was dried and crude mixture was purified by automated column chromatography, eluent: cyclohexane/ethyl acetate (70/30, v/v).

(2*R*,2'*R*)-4,6-dimethoxy-2',7,8''-trimethyl-4'',5''-dihydro-3H,3''H-dispiro[benzofuran-2,1'-cyclohexane-4',1''-pyrano[4,3-b]indol]-3-one **7r**

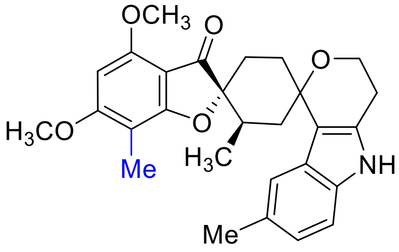

7r

42 mg, 89%. **^{1}H NMR** (600 MHz, Chloroform-*d*) δ 7.95 (s, 1H), 7.83 (s, 1H), 7.18 (d, *J* = 8.2 Hz, 1H), 6.97 (dd, *J* = 8.2, 1.6 Hz, 1H), 6.04 (s, 1H), 4.06 – 3.99 (m, 2H), 3.99 (s, 4H), 3.93 (s, 3H), 3.02 (td, *J* = 14.4, 4.2 Hz, 1H), 2.96 (dd, *J* = 14.4, 13.0 Hz, 1H), 2.86 – 2.79 (m, 1H), 2.76 (dt, *J* = 15.6, 5.0 Hz, 1H), 2.68 (ddt, *J* = 13.6, 11.2, 6.7 Hz, 1H), 2.53 (s, 3H), 2.43 (td, *J* = 13.5, 4.2 Hz, 1H), 2.08 (s, 3H), 1.92 (ddt, *J* = 14.4, 4.5, 2.5 Hz, 1H), 1.85 (ddd, *J* = 14.3, 4.2, 2.5 Hz, 1H), 1.78 (ddd, *J* = 12.9, 4.2, 2.6 Hz, 1H), 0.83 (d, *J* = 6.8 Hz, 3H) ppm. **^{13}C NMR** (151 MHz, $CDCl_3$) δ 200.62, 171.33, 166.56, 157.10, 133.83, 130.87, 129.10, 125.06, 122.81, 119.65, 115.06, 110.23, 104.76, 101.65, 91.24, 87.31, 74.39, 58.65, 56.08, 55.90, 38.47, 33.75, 29.60, 28.86, 24.59, 21.82, 14.81, 7.00 ppm. **135DEPT NMR** (151 MHz, $CDCl_3$) δ 122.81 (*sp*-CH), 119.65 (*sp*-CH), 110.23 (*sp*-CH), 87.31 (*sp*-CH), 58.65 (*sp3*-CH_2), 56.08 (*sp3*-CH_3), 55.90 (*sp3*-CH_3), 38.47 (*sp3*-CH_2), 33.75 (*sp3*-CH), 29.60 (*sp3*-CH_2), 28.86 (*sp3*-CH_2), 24.59 (*sp3*-CH_2), 21.82 (*sp3*-CH_3), 14.81 (*sp3*-CH_3), 7.00 (*sp3*-CH_3) ppm. **HRMS** (ESI-TOF): calculated for $C_{28}H_{32}NO_5$ [M+H$^+$], 462.2275; found, 462.2270. $[\alpha]_D^{20}$ +39.2 (*c* 0.15, $CHCl_3$).

(2*R*,2'*R*)-4,6-dimethoxy-7-(4-methoxyphenyl)-2',8''-dimethyl-4'',5''-dihydro-3H,3''H-dispiro[benzofuran-2,1'-cyclohexane-4',1''-pyrano[4,3-b]indol]-3-one **7s**

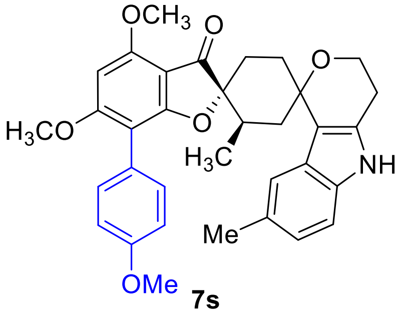

7s

42 mg, 75%. **¹H NMR** (600 MHz, Chloroform-*d*) δ 7.94 (s, 1H), 7.76 (s, 1H), 7.48 – 7.43 (m, 2H), 7.19 (d, *J* = 8.2 Hz, 1H), 7.01 – 6.95 (m, 3H), 6.15 (s, 1H), 4.06 (s, 3H), 4.01 – 3.92 (m, 2H), 3.90 (s, 3H), 3.86 (s, 3H), 2.99 (td, *J* = 14.4, 4.2 Hz, 1H), 2.97 – 2.90 (m, 1H), 2.81 (dt, *J* = 15.5, 5.4 Hz, 1H), 2.74 (dt, *J* = 15.5, 5.0 Hz, 1H), 2.64 (ddt, *J* = 13.6, 11.1, 6.7 Hz, 1H), 2.55 (s, 3H), 2.42 – 2.35 (m, 1H), 1.90 (ddt, *J* = 14.4, 4.6, 2.5 Hz, 1H), 1.85 – 1.79 (m, 1H), 1.79 – 1.74 (m, 1H), 0.87 (d, *J* = 6.9 Hz, 3H) ppm. **^{13}C NMR** (151 MHz, $CDCl_3$) *δ* 200.56, 170.75, 165.57, 158.56, 158.02, 133.79, 131.84, 130.85, 129.13, 125.05, 123.54, 122.83, 119.64, 115.07, 115.05, 113.46, 110.19, 106.90, 105.28, 91.52, 87.90, 74.29, 58.58, 56.30, 56.06, 55.23, 38.40, 33.52, 29.50, 28.62, 24.56, 21.85, 14.97 ppm. **HRMS** (ESI-TOF): calculated for $C_{34}H_{36}NO_6$ [M+H$^+$], 554.2537; found, 554.2532. $[\alpha]_D^{20}$ +75.4 (*c* 0.12, $CHCl_3$).

X-ray analysis of compound **7c**

The crystal structure of compound **7c** was determined using the *Bruker D8 Venture* four-circle diffractometer equipped with a *PHOTON II* CPAD detector by *Bruker AXS GmbH*. The X-ray radiation was generated by the *IµS/IµS* 3.0 microfocus source Mo (Λ = 0.71073 Å) from *Incoatec GmbH* equipped with HELIOS mirror optics and a single-hole collimator by *Bruker AXS GmbH*. The selected single crystal of **7c** was covered with an inert oil (perfluoropolyalkyl ether) and mounted on the *MicroMount* from *MiTeGen*. The APEX 3 Suite (v.2018.7-2) software integrated with SAINT (integration) and SADABS (adsorption correction) programs by *Bruker AXS GmbH* were used for data collection. The processing and finalization of the crystal structure were performed using the Olex2 program.[4] The crystal structures were solved by the ShelXT[5] structure solution program using the Intrinsic Phasing option, which were further refined by the ShelXL[6] refinement package using Least Squares minimization. The non-hydrogen atoms were anisotropically refined. The C-bound H atoms were placed in geometrically calculated positions, and a fixed isotropic displacement parameter was assigned to each atom according to the riding-model: C–H = 0.95–0.99 Å with Uiso(H) = 1.5Ueq(CH3) and 1.2Ueq(CH_2, CH) for other hydrogen atoms. The N-bound hydrogen atom on N1 was located on the Difference-Fourier-Map and refined independently.

The crystallographic data for the structure of **7c** has been published as supplementary publication number 2046918 in the Cambridge Crystallographic Data Centre. A copy of these data can be obtained for free by applying to CCDC, 12 Union Road, Cambridge CB2 IEZ, UK, fax: 144-(0)1223-336033 or e-mail: deposit@ccdc.cam.ac.uk.

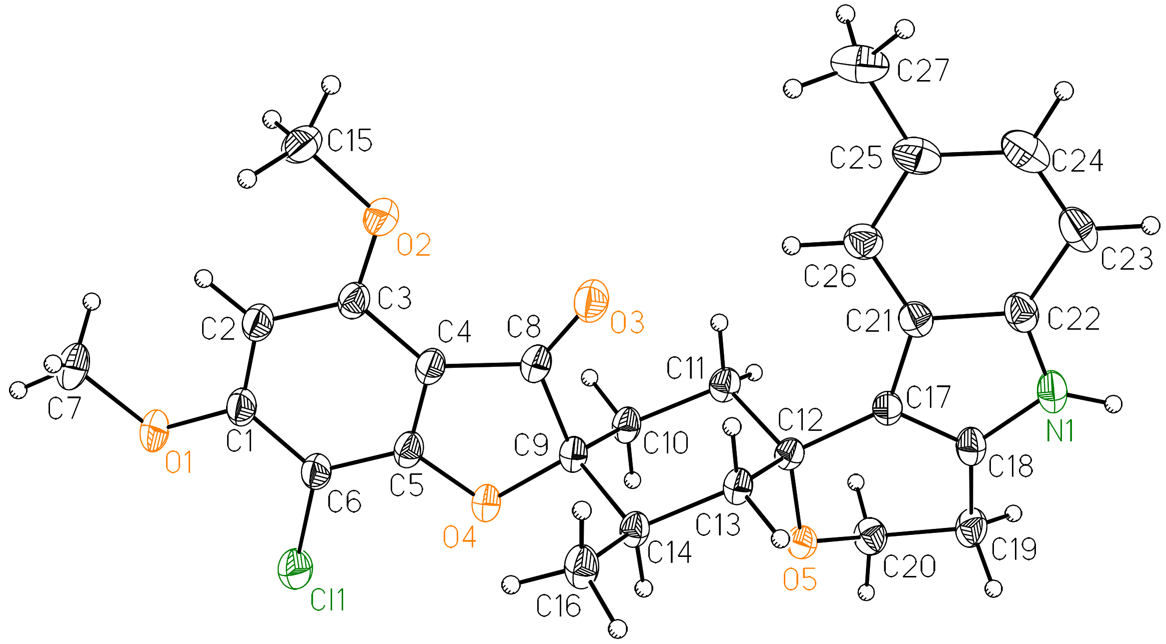

7c

Figure S3: Ortep plot of the molecular structure in the crystal of compound **7c**.[7] The displacement ellipsoids are drawn at the 50% probability level. Numbering scheme of hydrogen atoms are omitted for clarity. Crystallographic data have been deposited at the Cambridge Crystallographic Data Cetre and copies can be obtained on request, free of charge, by quoting the publication citation and the deposition number CCDC 2046918.

Table S2: Crystallographic data of compound **7c**.

Compound	**7c**
Empirical formula	$C_{27}H_{28}ClNO_5$
Formula weight	481.95
Temperature/K	100.01
Crystal system	orthorhombic
Space group	$P2_12_12_1$
a/Å	7.2790(5)
b/Å	18.0636(11)
c/Å	21.1324(13)
Volume/Å^3	2778.6(3)
Z	4
ρ_{calc}g/cm^3	1.152
μ/mm^{-1}	0.171
F(000)	1016.0
Crystal size/mm^3	0.411 × 0.142 × 0.114
Radiation	MoKα (λ = 0.71073)
2Θ range for data collection/°	5.92 to 61.11
Index ranges	$-10 \leq h \leq 10$, $-25 \leq k \leq 25$, $-30 \leq l \leq 30$
Reflections collected	526436
Independent reflections	8491 [R_{int} = 0.0424, R_{sigma} = 0.0082]
Data/restraints/parameters	8491/0/311
Goodness-of-fit on F^2	1.068
Final R indexes [I>=2σ (I)]	R_1 = 0.0338, wR_2 = 0.0987
Final R indexes [all data]	R_1 = 0.0347, wR_2 = 0.0998
Largest diff. peak/hole / e Å^{-3}	0.26/–0.31
Flack parameter	0.020(6)

General methods for biological experiments

Cell Culture of Mammalian Cells

Experiments with living mammalian cells were performed in a sterile environment in cell culture-approved clean benches with sterile equipment and media. All cells lines were cultured in a humidified atmosphere at 37 °C and 5% CO_2. Waste generated during the work with cells was collected and subsequently, sterilized with in an autoclave (134 °C, 15 min).

Human breast cancer MCF7 cells were cultured in Eagle’s dulbecco's modified eagle medium (DMEM) (PAN Biotech, cat# P04-03550) with the addition of 10% fetal bovine serum (FBS) (Invitrogen, cat# 10500-084), 1% sodium pyruvate (PAN Biotech, cat# P04-43100), 1% nonessential amino acids (PAN Biotech, cat# P08-32100), and 0.01 mg/mL bovine insulin (Sigma Aldrich, cat# I9278). The stably transfected MCF7 cells with eGFP-LC3 (MCF7/LC3) were incubated under the same conditions, but with the addition of 200 µg/mL G418 (Sigma Aldrich, Cat. No.: G8168) in the medium.

Passaging of Mammalian Cells

All solutions necessary were prewarmed to 37 °C in a water bath. The cells were grown to 70-80% confluence in culture flasks (75 cm^2). The medium was removed and the cells were washed with PBS (10 mL). For detachment, the cells were treated with the addition of a trypsin/EDTA solution (2 mL, 37 °C, 2 min). Subsequently, fresh medium was added and the desired volume of the cell suspension was transferred into a new tissue flask (75 cm^2). The flask accordingly filled up with medium to a final volume of 10 mL.

Cryo-conservation and thawing of cryo-conserved cells

For cryo-conservation, the cells were cultured until confluence in a tissue culture flask (175 cm^2). After trypsinization and centrifugation (350 g, 5 min), the cell pellet was resuspended in the respective medium (6 mL) with DMSO (5% v/v). The suspension was transferred to cryo-conservation vials and stored in a cell freezing container (CoolCell® LX) at -80 °C overnight. For long-term storage the cells were stored in liquid nitrogen.

Cryo-conserved cells were rapidly thawed in a water bath (37 °C, 2 min). After the transfer to the respective medium (10 mL), the suspension was centrifuged (350 g, 5 min). The supernatant was removed, the cell pellet resuspended in fresh medium (10 mL) and transferred to tissue culture flasks (25 cm^2).

Cell Counting

The cells were counted in an automated cell counter Countess™ II Automated Cell Counter according to the manufacturer's instructions. After the dilution of the cells with Trypan blue (ratio 1:1) to identify dead cells, 10 µL of the suspension were transferred to a Countess® Cell Counting Chamber Slide (Thermo Fisher Scientific, Cat. No. C10228) and the slide was inserted to the machine to directly give the cell number.

Autophagy assay: GFP-LC3 puncta formation assay

The GFP-LC3 puncta formation assay was performed by the Compound Management and Screening Center (COMAS) in a medium throughput manner based on the work of Balgi et. al.[8] Stably transfected MCF7 cells expressing eGFP-LC3 (400 cells/well in a volume of 25 µL per well) were seeded in 384 well-plates (Greiner: cat# 781080, lid cat# 656191). The cells were incubated overnight (37 °C, 5% CO_2) and subsequently washed with PBS (Biotek, ELx405, 3x). For the compound treatment, the stock solution (10 mM in DMSO, 25 µL) was added employing an echo dispenser (Labcyte). The respective medium (25 µL EBSS with chloroquine (50 µM) or standard medium supplemented with chloroquine (50 µM) and rapamycin (Biomol, cat# Cay13346, 100 nM)) were added with a Multidrop Combi (Thermo Scientific). After an incubation for 3 h (37 °C, 5% CO_2), the cells were fixed with 25 µL of a fixing solution (formaldehyde/PBS (1:4)). The nuclei were stained with 1:500 Hoechst (stock: 1 mg/ml, Sigma Aldrich cat# B2261-25mg) at ambient temperature for 20 min. After the cells were washed three times with PBS, fluorescent pictures were taken by an ImageXpress Micro XL (Molecular Devices, 4 sites per well, 20x magnification). The granularity setting of MetaXpress Software (Molecular Devices) was employed for an automated image analysis.

Immunoblot for Autophagy Markers

MCF7/LC3 cells (300,000 cells/ 2 mL medium) were seeded in 6-well plates and incubated overnight (37 °C, 5% CO_2). The next day the medium was removed and substituted by fresh medium or EBSS for starvation conditions. After compound addition (2 µL of the respective DMSO solution), the cells were incubated for 3 h (37 °C, 5% CO_2). The cells were washed with PBS (1x 1 mL), trypsinated and collected with a cell scraper. The cell suspension was centrifuged (4000 rpm, 5 min) and the pellet was washed with PBS (1 mL). Then 50 µL lysis buffer (50 mM PIPES, 50 mM NaCl, 5 mM EGTA, 5 mM $MgCl_2$, 0.1% NP-40, 0.1%TX-100, 0.1% Tween at pH=7.4) was added to the cells and the suspension was incubated for 30 min on ice while inverting the tube every 10 min. After another centrifugation (20 min, 14000 rpm, 4 °C), the supernatant was

collected. The protein concentration was measured via DC assay according to the "DC protein assay instruction manual". The BSA standard curve was detected for protein concentrations between 0 – 2.5 mg/mL. The absorbance was measured at 750 nm by a Tecan plate reader.

All samples were diluted with SDS leading buffer (5x, 50% v/v Glycerol, 250 mM Tris (pH 6.8), 10% w/v SDS, 500 mM DTE, 360 µM bromophenol blue) to a final concentration of 2% w/v and heated to 95 °C for 5 min. The samples were stored at -80 °C until loading them to an SDS gel.

To separate the proteins according to their size a sodium dodecyl sulfate–polyacrylamide gel electrophoresis (SDS-PAGE) was performed using a Mini-PROTEAN® Tetra Cell chamber (Bio Rad) according to the standard method[9]. The denaturated samples were loaded onto the SDS-gel (separation gel: 12.5%). The PageRuler™ Plus Prestained Protein Ladder (Thermo Fisher Scientific, Cat. No. 26619) was used as marker. The gel was initially run at a constant voltage of 80 V for 30 min and afterwards the electric voltage was increased to 120 V for another 60 min.

The separated proteins were transferred to a polyvinylidene fluoride (PVDF) membrane, which was shortly activated in methanol and subsequently equilibrated in transfer buffer for 15 min. The filter paper was also soaked in transfer buffer before transferring the parts to the Trans-Blot® SD Semi-Dry Transfer Cell (Bio-Rad) system, where it was blotted according to the manufactures instructions (25 V, 25 min).

The membrane was washed with water and stained with Ponceau S as control for the protein transfer. The staining was removed by repeatedly washing the membrane with water. The membrane was subsequently blocked with blocking buffer for 1 h at ambient temperature. The primary antibody in blocking solution (5% w/v milk powder in PBS-T) was added and the membrane was incubated overnight at 4 °C. The primary antibody solution was removed, the membrane was washed (3x 5 mL PBS-T, 10 min), and incubated with the secondary antibody, which was coupled to a near-infrared dye (IRDye 680RD or IRDye 800CW, Licor) in blocking solution (see table below). After the membrane was washed (3x 5 mL PBS-T, 10 min), the protein band were visualized on the ChemiDoc™ MP Imaging System, BioRad.

The autophagy marker p62 and LC3 were detected and vinculin was chosen as a loading control.

antibody	host	dilution	supplier	Cat. No.
LC3	rabbit	1:1000	Cell Signaling	2775
p62	rabbit	1:10000	MBL international	PM045
vinculin	mouse	1:10000	Sigma Aldrich	V9131
IRDye 680RD anti rabbit	goat	1:10000	LI-COR	P/N 926-68071
IRDye 800CW goat anti-mouse	goat	1:10000	LI-COR	P/N 926-32210

Mito Stress Test

The Mito Stress Test was performed in the Seahorse XFp analyzer (Agilent, USA) with the Cell Mito Stress Test kit (Agilent, USA, No. 103015-100) according to the manufacturer's protocol. 20,000 MCF7 cells in 100 µL medium were seeded into the XFp cell culture plates (Agilent, USA) and incubated overnight (37 °C, 5% CO_2). The XFp cartridges were hydrated with the XF Calibrant solution (Agilent, USA) and also incubated overnight at 37 °C without CO_2.

The medium was substituted with 180 µL assay medium (XF base medium (pH 7.4, Agilent, USA), 2 mM GlutaMAX (ThermoFisher), 1 mM sodium pyruvate (PAN Biotech, Germany) and 25 mM glucose (Sigma-Aldrich, Germany)). The plate was equilibrated for 45 min in an incubator (37 °C, no CO_2) and to the stock solutions of the kit assay medium was added to give the desired concentrations of 50 mM for oligomycin and FCCP and 25 µM for rotenone/antimycin A. The compounds were diluted in assay medium according their desired concentrations and loaded onto the respective injection ports. The plates were submitted to the Seahorse XFp analyzer and the oxygen consumption rate (OCR) and extracellular acidification rate (ECAR) were measured every 6 min. After the detection of five cycles for the baseline, the test compounds and DMSO as controls were added at the desired concentrations and measured for ten intervals. Afterwards oligomycin, FCCP and rotenone/antimycin A were injected successive, whereas each addition was detected for three cycles. The results were analyzed with the Wave software, where the background was subtracted and normalized to the fifth base line measurement (=100%).

In-vitro Tubulin Polymerization assay

Porcine *α/β*-tubulin (> 99% pure, Cytoskeleton, Denver, USA) was dissolved in buffer (80 mM Na-PIPES pH 6.9, 1 mM $MgCl_2$, 1 mM EGTA and 0,88 mM Na-glutamate). The compounds were added at the desired concentrations and the mixture was incubated on ice for 30 min. The addition of GTP (0.4 mM) induced the start of the polymerization, which was detected by the absorption at 340 nm in the Inifinite® M200 plate reader (Tecan, Grödig, Austria) at 37 °C for 75 min. For the analysis the background was subtracted.

In cell histone staining for the mitotic arrest

5000 cells in a volume of 100 µL medium per well were seeded in clear flat-bottom 96 well plates (Corning) and incubated overnight (37 °C, 5% CO_2). The medium was substituted by 100 µL medium with compound or controls and incubated for another 3 h (37 °C, 5% CO_2). The media was removed and 100 µL 3.7% formaldehyde in PBS were added. After an incubation of 10 min, 100 µL of 0.1% Triton X-100 in PBS was added and incubated for 15 min. The Triton solution was removed and the cells were washed with PBS-T. 100 µL 2% BSA in PBS-T was added to the cells and the cells were incubated for 1 h. The corresponding antibodies were added in 40 µL 2% BSA in PBS-T (see table below). After the antibody solution was removed, the cells were washed once more with PBS-T. Finally, 100 µL PBS-T were added for imaging. On the screening microscope the cells were imaged at 20-fold magnification in the 4', 6-diamidino-2-phenylindole, dihydrochloride (DAPI) and Texas Red channel. The resulting data were analyzed by quantifying the LysoTracker^{TM} Red DND-99 staining with the software CellProfiler.

antibody/ compound	host	dilution	supplier	Cat. No.
DAPI	-	1:1000	Sigma Aldrich	D9542-10MG
Tubulin-FITC	mouse	1:500	Thermo Fisher Scientific	MA119581
Phospho Histone H3-AF594	rabbit	1:500	Cell Signalling	#8481

Cell Painting Assay Experimental and Analyses

The experimental procedure of the cell painting assay is based of the method reported by Bray et. al.[10]

5 µl U-2OS medium were added to each well of a 384-well plate (PerkinElmer CellCarrier-384 Ultra) and 1600 U-2OS cells in 20 µl medium were seeded per well. After the plate was left for 10 min at the ambient temperature, the cells were incubated for 4 h (37 °C, 5% CO_2). Compound treatment was realized employing the Echo 520 acoustic dispenser (Labcyte) at final concentrations of 10 µM, 3 µM or 1 µM. Subsequently, the treated cells were incubated for 20 h (37 °C, 5% CO_2). Afterwards, mitochondria were stained with Mito Tracker Deep Red (Thermo Fisher Scientific, Cat. No. M22426). The Mito Tracker Deep Red stock solution (1 mM) was diluted in prewarmed medium to a final concentration of 100 nM. After removing the medium from the plate and keeping 10 µl residual volume, 25 µl of the Mito Tracker solution were added to each well. The plate was incubated for 30 min in darkness (37 °C, 5% CO_2). The cells were fixed with 7 µl of 18.5% formaldehyde in PBS, resulting in a final formaldehyde concentration of 3.7%. Subsequently, the plate was incubated for another 20 min (darkness, at ambient temperature) and washed with 70 µl of PBS (3x, Biotek Washer Elx405). To permeabilize the cells, 25 µl 0.1% Triton X-100 were added to each well and incubated for 15 min (darkness, at ambient temperature). The cells were washed with PBS (3x), keeping a final volume of 10 µl per well. 25 µl of a staining solution (1% BSA, 5 µl/ml Phalloidin (Alexa594 conjugate, Thermo Fisher Scientific, A12381), 25 µg/ml Concanavalin A (Alexa488 conjugate, Thermo Fisher Scientific, Cat. No. C11252), 5 µg/ml Hoechst 33342 (Sigma, Cat. No. B2261-25mg), 1.5 µg/ml WGA-Alexa594 conjugate (Thermo Fisher Scientific, Cat. No. W11262), and 1.5 µM SYTO 14 solution (Thermo Fisher Scientific, Cat. No. S7576) were added to each well. The plate was incubated for another 30 min (darkness, at ambient temperature) and washed with 70 µl PBS (3x). The PBS of the final washing step was kept in each well. After the plates were sealed, they were centrifuged (1 min at 500 rpm).

The plates were prepared in triplicates by applying shifted layouts that result in the reduction of plate effects. The plates were imaged employing a Micro XL High-Content Screening System (Molecular Devices) in 5 channels (DAPI: Ex350-400/ Em410-480; FITC: Ex470-500/ Em510-540; Spectrum Gold: Ex520-545/ Em560-585; TxRed: Ex535-585/ Em600-650; Cy5: Ex605-650/ Em670-715) with 9 sites per well and 20x magnification (binning 2).

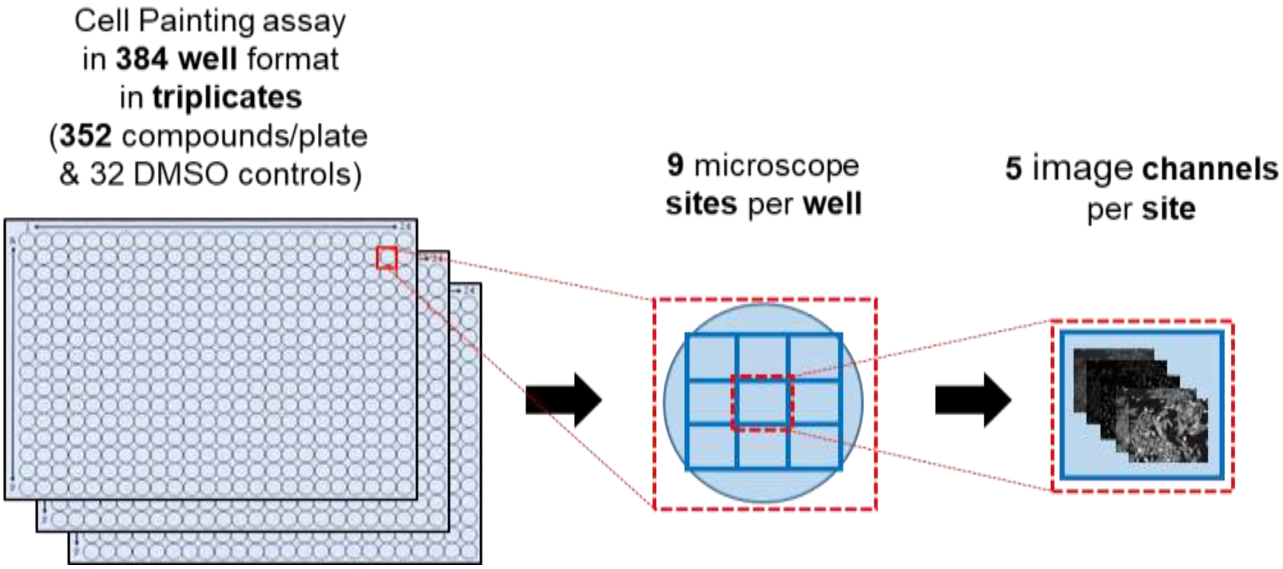

The high content images of the cell painting assay were processed and analyzed with the *CellProfiler* package (https://cellprofiler.org/, version 3.0.0) on a computing cluster of the Max Planck Society to extract 1716 cell features (refers as parameters) per microscope site. Subsequently, the data were aggregated as medians per well (9 sites → 1 well) and over the three replicates.

Further analysis was performed with custom *Python* (https://www.python.org/) scripts using the *Pandas* (https://pandas.pydata.org/) and *Dask* (https://dask.org/) data processing libraries as well as the *Scientific Python* (https://scipy.org/) package.

From the 1716 extracted features, a subset of highly reproducible and robust features was identified employing a procedure reported by Woehrmann et al.[11]: Two biological repeats of a plate with reference compounds were analyzed. For every feature, the full profile over each whole plate was calculated. If the profiles from the two repeats showed a biological similarity >= 0.8 (see below), the feature was included in the subset.

The procedure was performed once and identified 579 robust features, which were used for the profiles and comparisons.

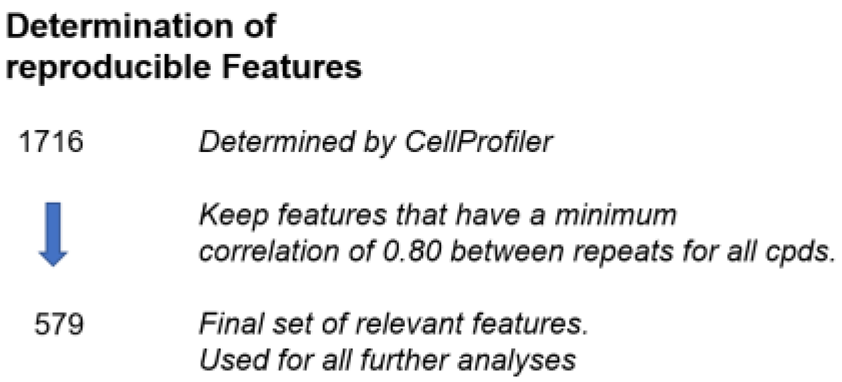

The morphological profile of a compound represents a list of the z-scores of all parameters. Z-scores were calculated for each feature by the following equation as the difference to the median of the controls divided by their MAD.

$$\text{z-score} = \frac{(\text{value}_{\text{meas.}} - \text{median}_{\text{controls}})}{\text{MAD}_{\text{controls}}}$$

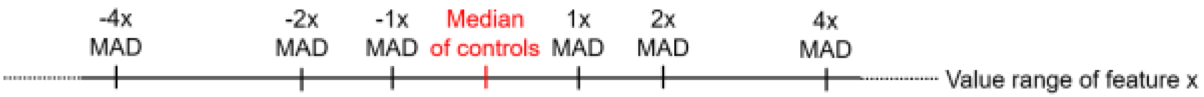

The induction (Ind.) was introduced and represents the of significantly changed features for each compound compared to the control. The value is indicated in percent:

$$\text{induction} = \frac{(\text{number of parameters with abs. values} > 3)}{\text{total number of parameters}}$$

Similarities of phenotypic profiles were determined from the correlation distances between two profiles (https://docs.scipy.org/doc/scipy/reference/generated/scipy.spatial.distance.correlation.html; Similarity = 1 - Correlation Distance) and referred as biological similarity (Bio. Sim.).

A comparison with 3000 references that were also measured in this assay enables the identification with the most similar profiles.

Profile similarity is demonstrated on two examples:

Compound 1 and 2 show highly similar profiles with a resemblance of 96%.

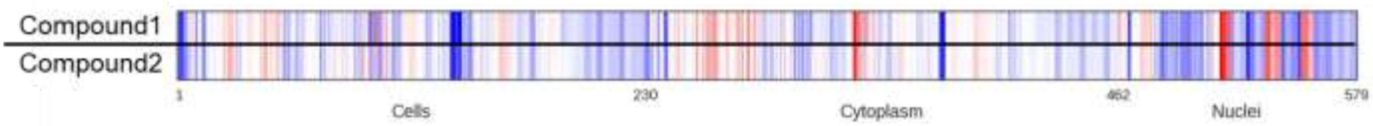

Compound 1 and 3 show a low biological similarity of 0%.

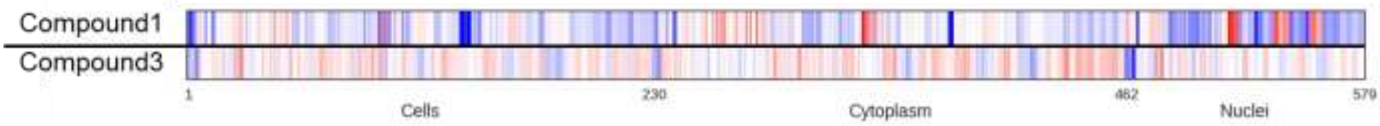

Each colored band represents one Z-score of a feature.

To draw comparisons, the profile similarity is translated as ‘biological similarity’ (Bio. Sim which corresponds to the resemblance between morphological profiles in percent. Compounds with a biological similarity above 75% are considered to be ‘similar’, whereas comparisons with a lower value (<75%) are defined as ‘dissimilar’.

To enable a meaningful analysis, effects that appear to be not compound related needed to be eliminated to exclude false positive effects. One example was identified as dominating profile, which is especially dominant for compounds with high induction values (>80%). The dominating profile is not completely understood yet and is still under investigation. To avoid misrepresentation, the induction limit for analysis was set to 60. Another example is an induction-dependent biosimilarity between compounds. The comparison of compounds with broad induction ranges, compounds were found to show high biological similarities due to similar induction. Induction ranges of 25% or less were found to show meaningful results and were used for the profile comparison.

References

[1] G. R. Fulmer, A. J. M. Miller, N. H. Sherden, H. E. Gottlieb, A. Nudelman, B. M. Stoltz, J. E. Bercaw, K. I. Goldberg, *Organometallics* **2010**, *29*, 2176.

[2] T. Bach, L. Jiao, *Synthesis* **2013**, *46*, 35.

[3] M. H. Rønnest, P. Harris, C. H. Gotfredsen, T. O. Larsen, M. H. Clausen, *Tetrahedron Lett.* **2010**, *51*, 5881.

[4] O. V. Dolomanov, L. J. Bourhis, R. J. Gildea, J. A. K. Howard, H. Puschmann, *J. Appl. Crystallogr.* **2009**, *42*, 339.

[5] G. M. Sheldrick, *Acta Cryst.* **2015**, *A71*, 3.

[6] G. M. Sheldrick, *Acta Cryst.* **2015**, *C71*, 3.

[7] L. J. F. Ortep–3 V 2.02 for Windows, *J. Appl. Cryst.* 1997, 30, 565.

[8] A. D. Balgi, B. D. Fonseca, E. Donohue, T. C. Tsang, P. Lajoie, C. G. Proud, I. R. Nabi, M. Roberge, *PLoS One* **2009**, *4*, e7124.

[9] F. He, *Bio. Protoc.* **2011**, *1*.

[10] M. A. Bray, S. Singh, H. Han, C. T. Davis, B. Borgeson, C. Hartland, M. Kost-Alimova, S. M. Gustafsdottir, C. C. Gibson, A. E. Carpenter, *Nat. Protoc.* **2016**, *11*, 1757.

[11] M. H. Woehrmann, W. M. Bray, J. K. Durbin, S. C. Nisam, A. K. Michael, E. Glassey, J. M. Stuart, R. S. Lokey, *Mol. Biosyst.* **2013**, *9*, 2604.

LC-MS Traces of Indofulvins

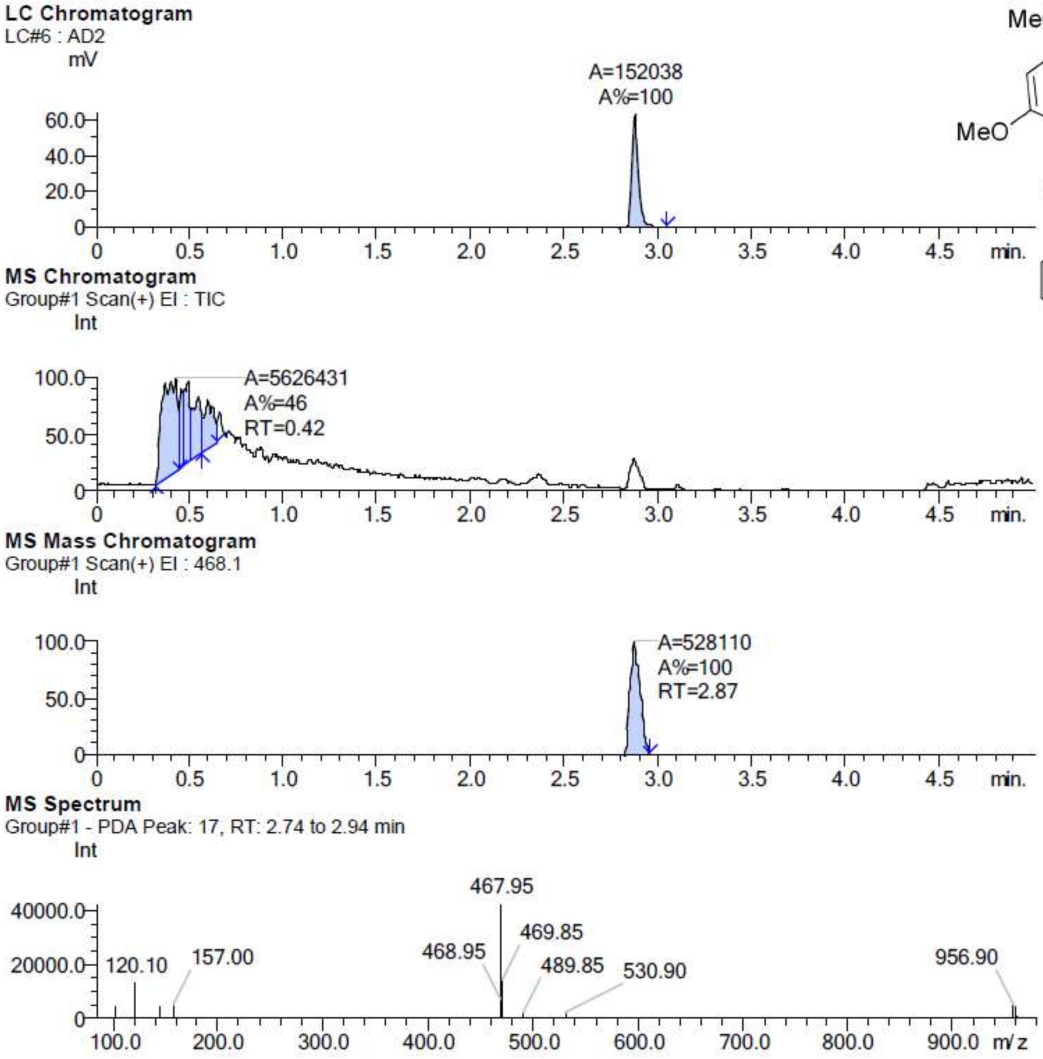

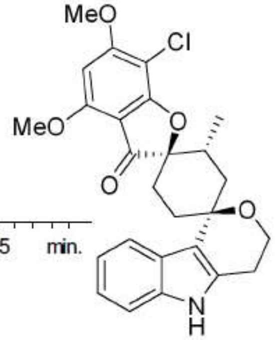

7a

LC Chromatogram
LC#6 : AD2

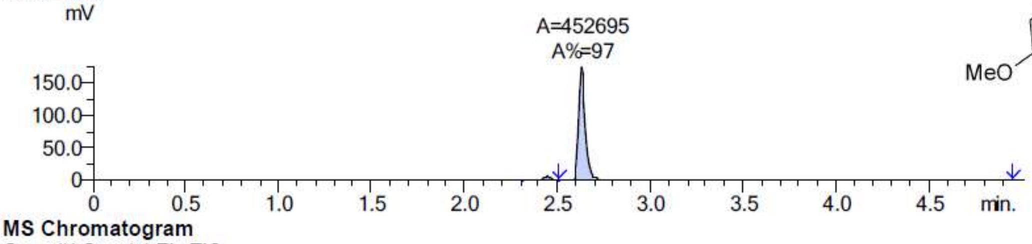

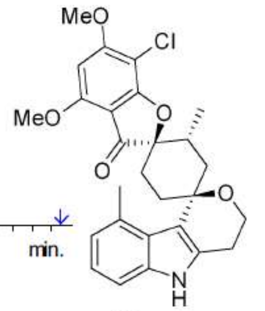

7b

MS Chromatogram
Group#1 Scan(+) EI : TIC

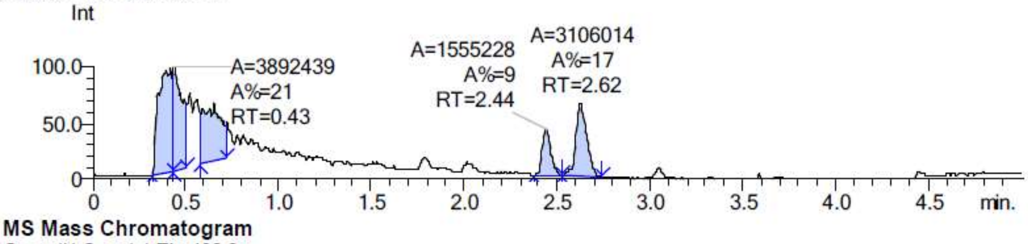

MS Mass Chromatogram
Group#1 Scan(+) EI : 482.2

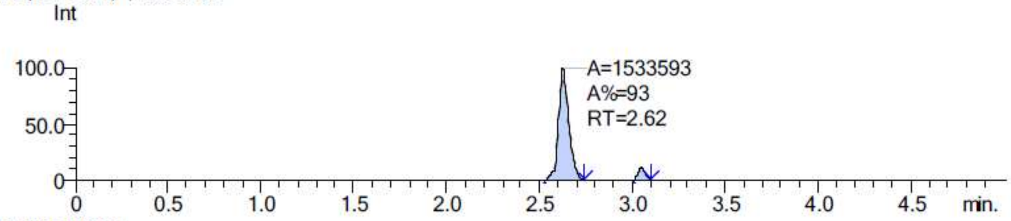

MS Spectrum
Group#1 - PDA Peak: 25, RT: 2.51 to 2.69 min

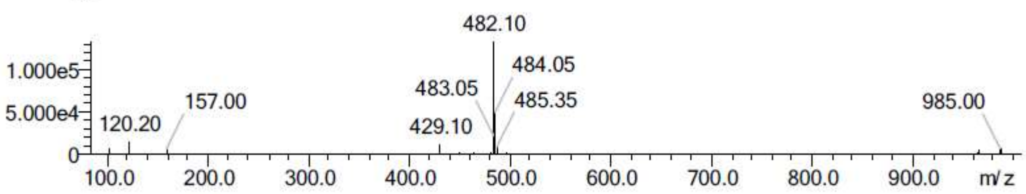

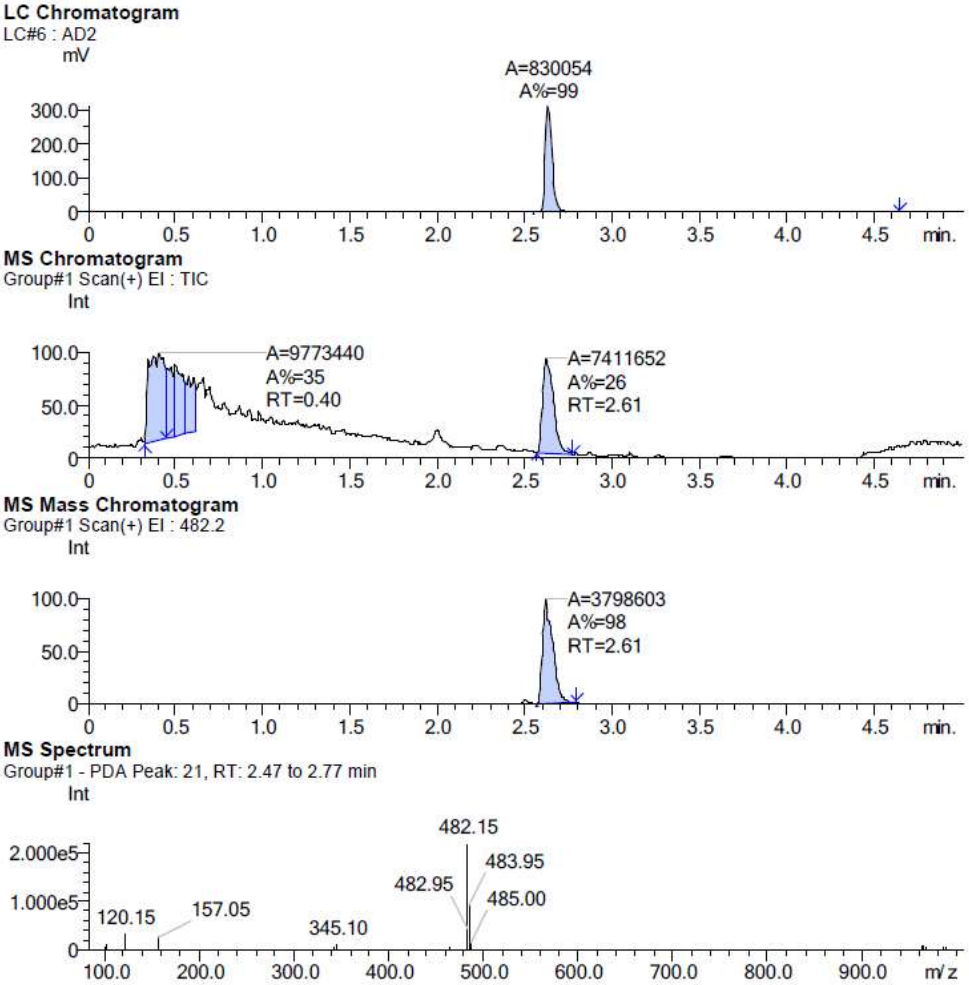
LC Chromatogram
LC#6 : AD2
mV
A=830054
A%=99
300.0
200.0
100.0
0
0.5
1.0
1.5
2.0
2.5
3.0
3.5
4.0
4.5
min.
MS Chromatogram
Group#1 Scan(+) EI : TIC
Int
100.0
50.0
A=9773440
A%=35
RT=0.40
A=7411652
A%=26
RT=2.61
MS Mass Chromatogram
Group#1 Scan(+) EI : 482.2
Int
A=3798603
A%=98
RT=2.61
MS Spectrum
Group#1 - PDA Peak: 21, RT: 2.47 to 2.77 min
Int
2.000e5
1.000e5
482.15
483.95
482.95
485.00
120.15
157.05
345.10
100.0
200.0
300.0
400.0
500.0
600.0
700.0
800.0
900.0
m/z

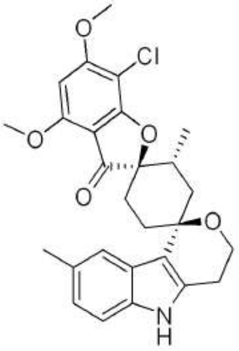
Cl
O
O
O
O
O
NH

7c

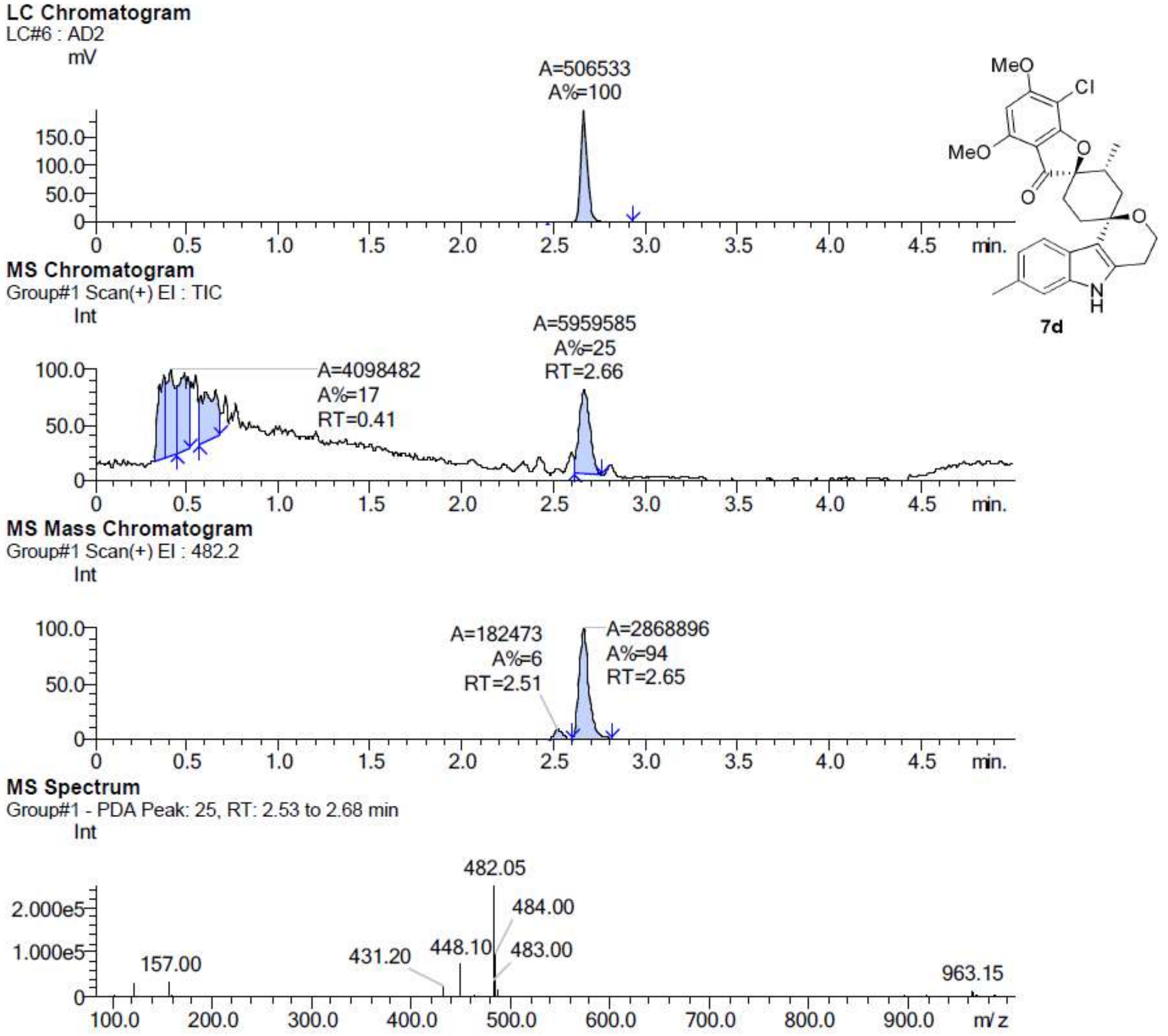
LC Chromatogram
LC#6 : AD2
mV
A=506533
A%=100
150.0
100.0
50.0
0
0 0.5 1.0 1.5 2.0 2.5 3.0 3.5 4.0 4.5 min.
MS Chromatogram
Group#1 Scan(+) EI : TIC
Int
A=5959585
A%=25
RT=2.66
A=4098482
A%=17
RT=0.41
100.0
50.0
0
0 0.5 1.0 1.5 2.0 2.5 3.0 3.5 4.0 4.5 min.
MS Mass Chromatogram
Group#1 Scan(+) EI : 482.2
Int
A=182473
A%=6
RT=2.51
A=2868896
A%=94
RT=2.65
100.0
50.0
0
0 0.5 1.0 1.5 2.0 2.5 3.0 3.5 4.0 4.5 min.
MS Spectrum
Group#1 - PDA Peak: 25, RT: 2.53 to 2.68 min
Int
482.05
484.00
483.00
448.10
431.20
157.00
963.15
2.000e5
1.000e5
0
100.0 200.0 300.0 400.0 500.0 600.0 700.0 800.0 900.0 m/z
MeO
Cl
MeO
O
O
O
N
H

7d

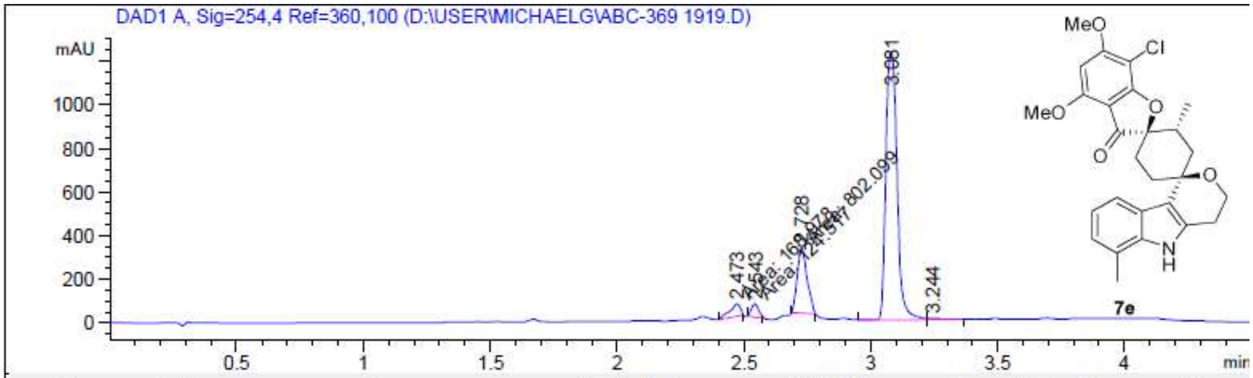

#	Time	Area	Height	Width	Area%	Symmetry
1	2.473	169	57.4	0.0491	3.448	2.391
2	2.543	124.5	61.8	0.0336	2.541	0.835
3	2.728	802.1	297.3	0.045	16.367	0.784
4	3.081	3778.3	1229	0.0489	77.100	0.854
5	3.244	26.7	6.2	0.0585	0.544	0.296

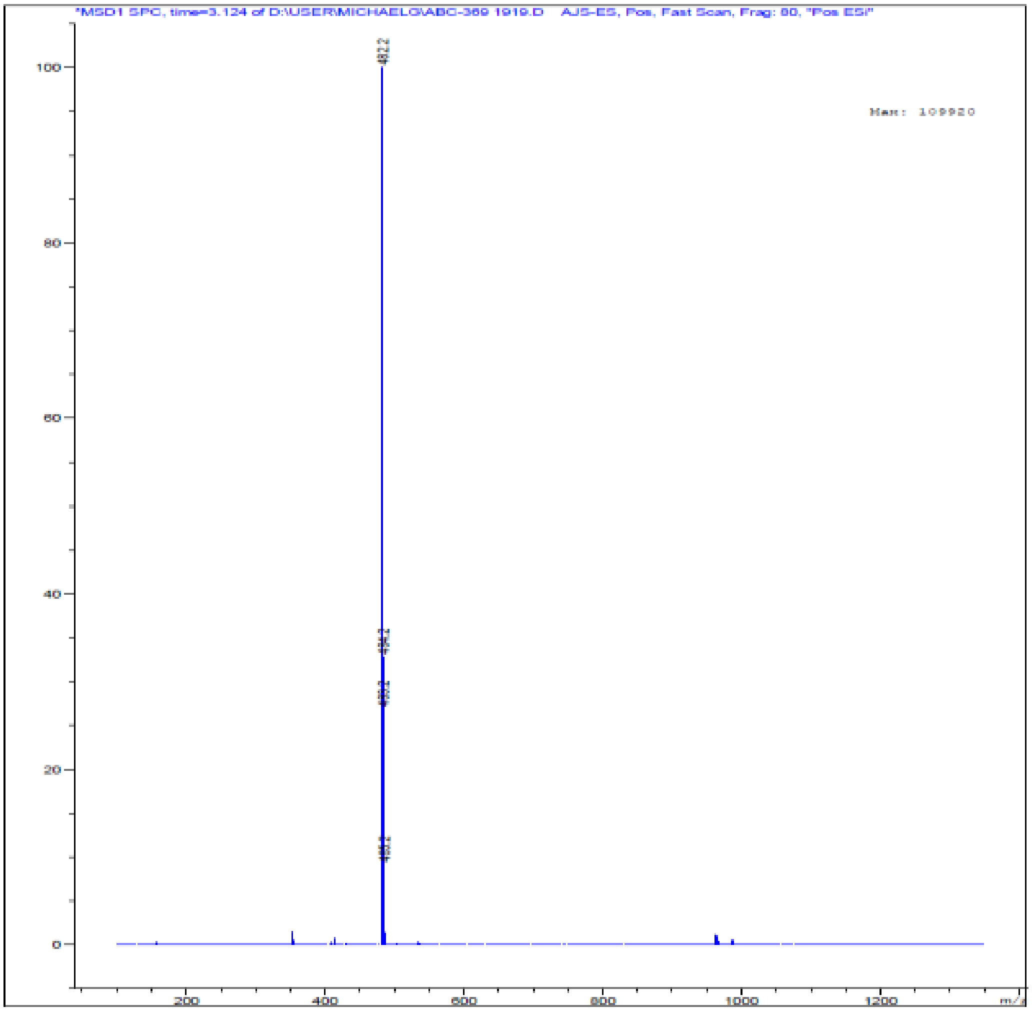

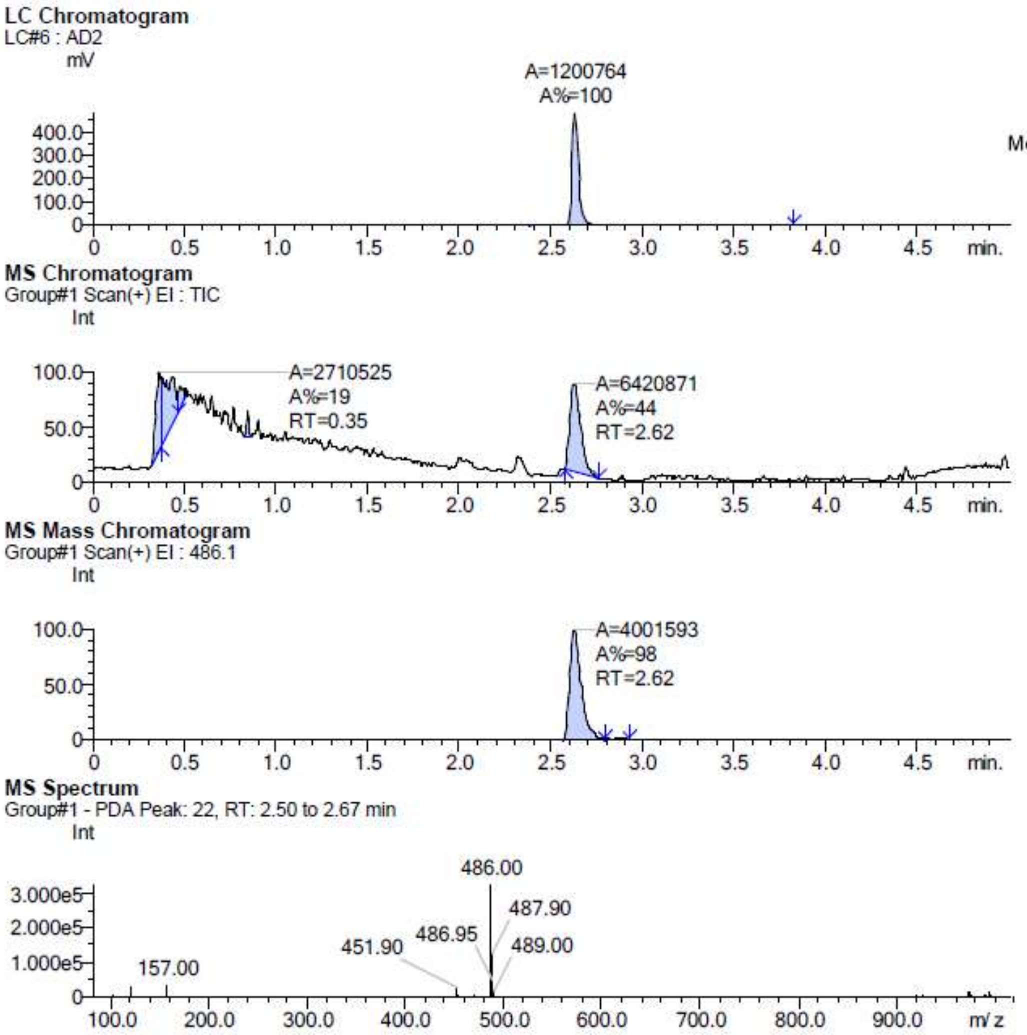
LC Chromatogram
LC#6 : AD2
mV
A=1200764
A%=100
400.0
300.0
200.0
100.0
0
0 0.5 1.0 1.5 2.0 2.5 3.0 3.5 4.0 4.5 min.
MS Chromatogram
Group#1 Scan(+) EI : TIC
Int
100.0
50.0
0
A=2710525
A%=19
RT=0.35
A=6420871
A%=44
RT=2.62
0 0.5 1.0 1.5 2.0 2.5 3.0 3.5 4.0 4.5 min.
MS Mass Chromatogram
Group#1 Scan(+) EI : 486.1
Int
100.0
50.0
0
A=4001593
A%=98
RT=2.62
0 0.5 1.0 1.5 2.0 2.5 3.0 3.5 4.0 4.5 min.
MS Spectrum
Group#1 - PDA Peak: 22, RT: 2.50 to 2.67 min
Int
3.000e5
2.000e5
1.000e5
0
486.00
487.90
486.95
489.00
451.90
157.00
100.0 200.0 300.0 400.0 500.0 600.0 700.0 800.0 900.0 m/z

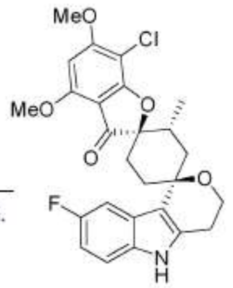
MeO
Cl
MeO
O
O
O
F
N
H

7f

LC Chromatogram
LC#6 : AD2

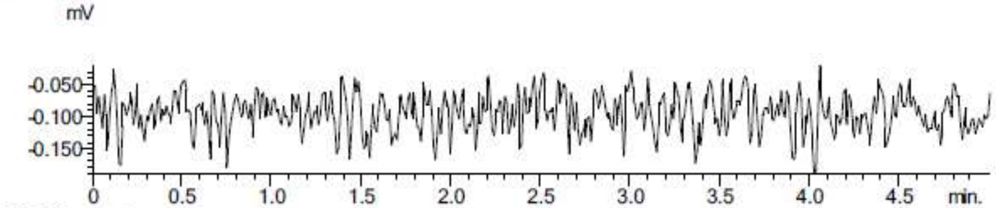

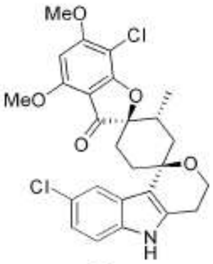

7g

MS Chromatogram
Group#1 Scan(+) EI : TIC

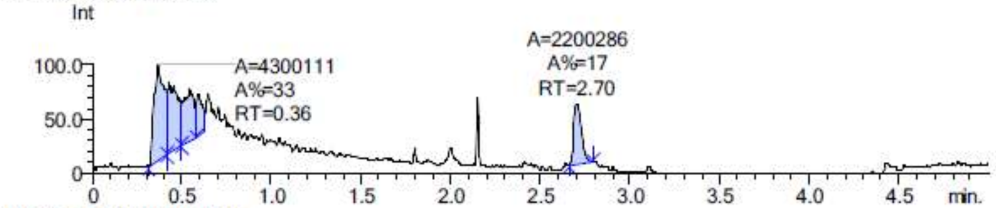

MS Mass Chromatogram
Group#1 Scan(+) EI : 502.1

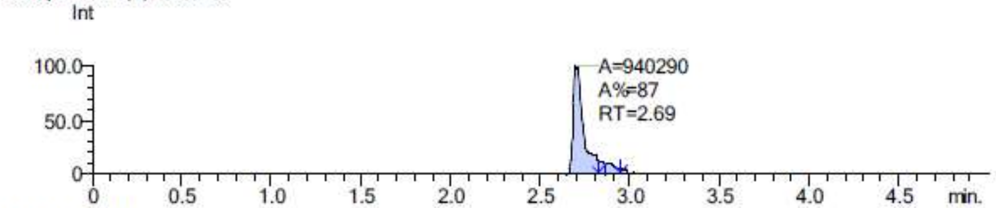

MS Spectrum
Group#1 - PDA Peak: 34, RT: 2.58 to 2.99 min

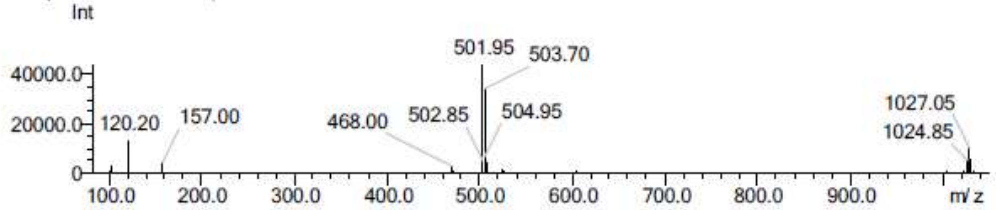

PDA Chromatogram
1: Wavelength 210 nm, Band Width 4 nm

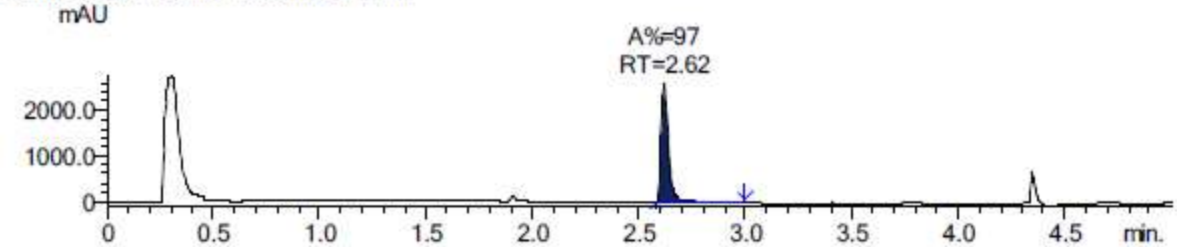

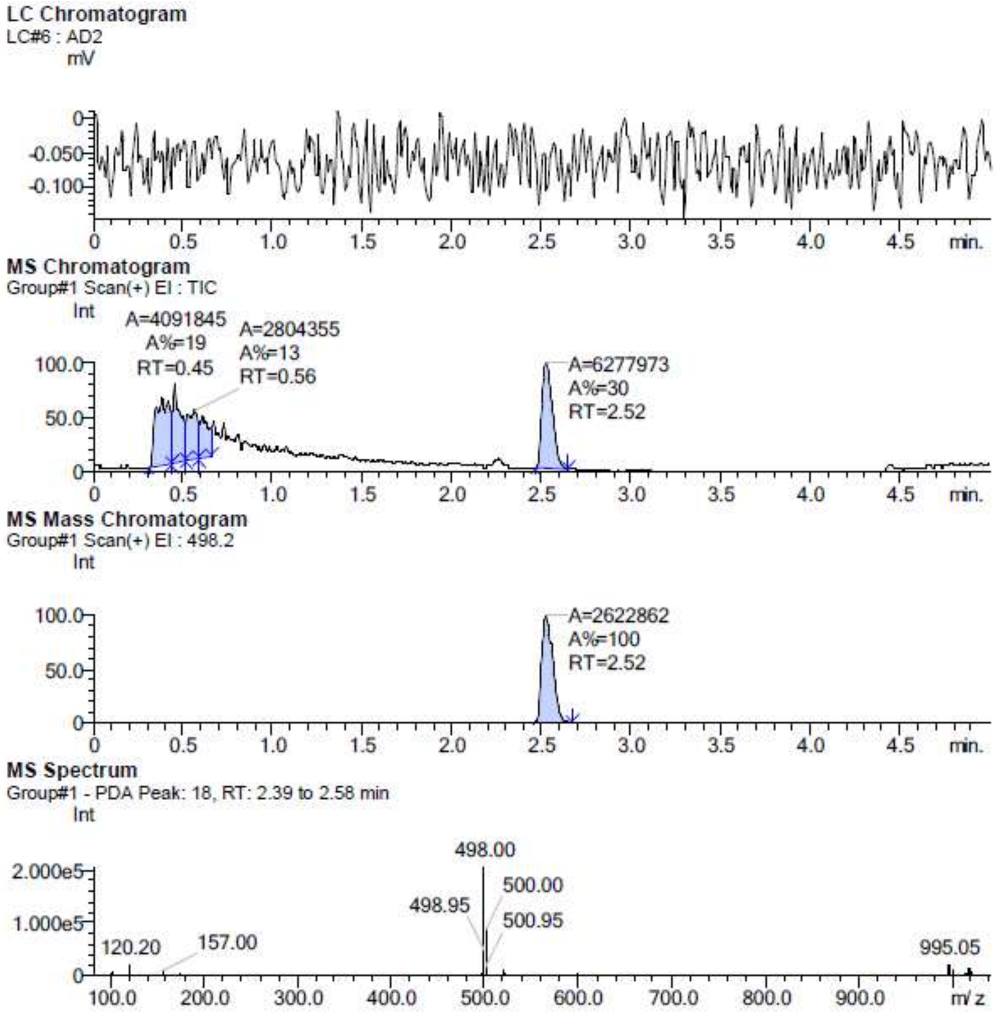
LC Chromatogram
LC#6 : AD2
mV
MS Chromatogram
Group#1 Scan(+) EI : TIC
Int
A=4091845
A%=19
RT=0.45
A=2804355
A%=13
RT=0.56
A=6277973
A%=30
RT=2.52
MS Mass Chromatogram
Group#1 Scan(+) EI : 498.2
Int
A=2622862
A%=100
RT=2.52
MS Spectrum
Group#1 - PDA Peak: 18, RT: 2.39 to 2.58 min
Int
498.00
500.00
498.95
500.95
120.20
157.00
995.05
m/z
min.

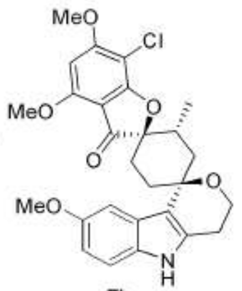
MeO
Cl
MeO
O
O
O
MeO
N
H

7h

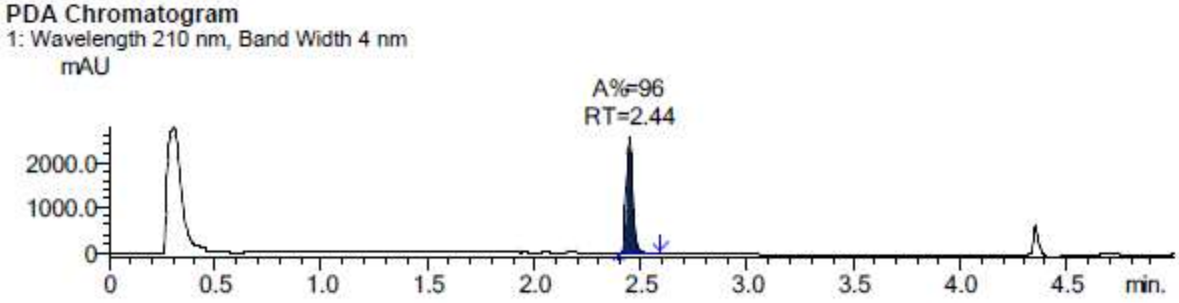
PDA Chromatogram
1: Wavelength 210 nm, Band Width 4 nm
mAU
A%=96
RT=2.44
min.

LC Chromatogram
LC#6 : AD2

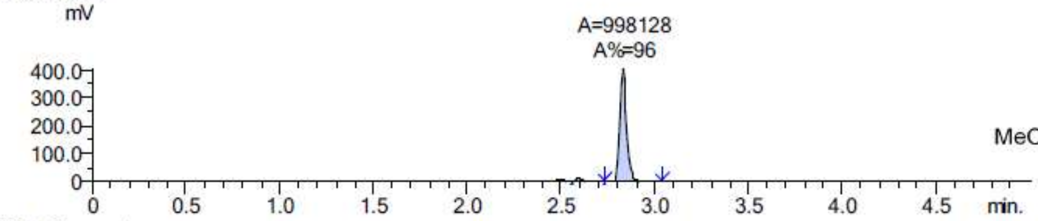

MS Chromatogram
Group#1 Scan(+) EI : TIC

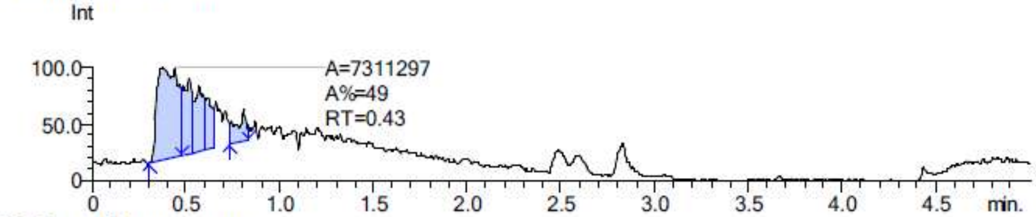

MS Mass Chromatogram
Group#1 Scan(+) EI : 574.2

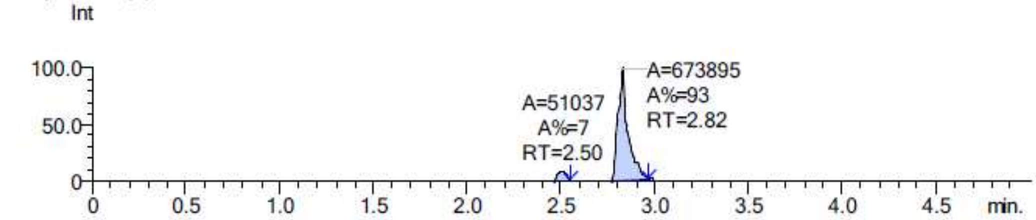

MS Spectrum
Group#1 - PDA Peak: 27, RT: 2.67 to 2.87 min

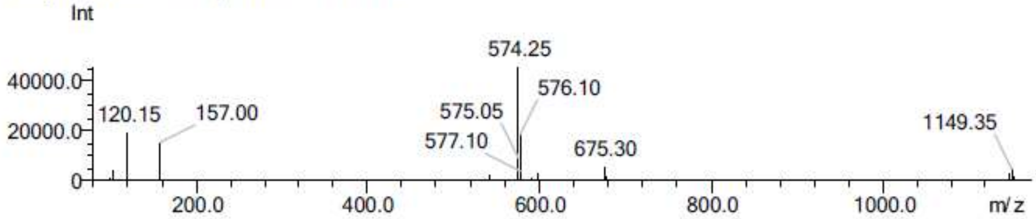

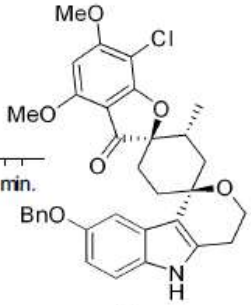

7i

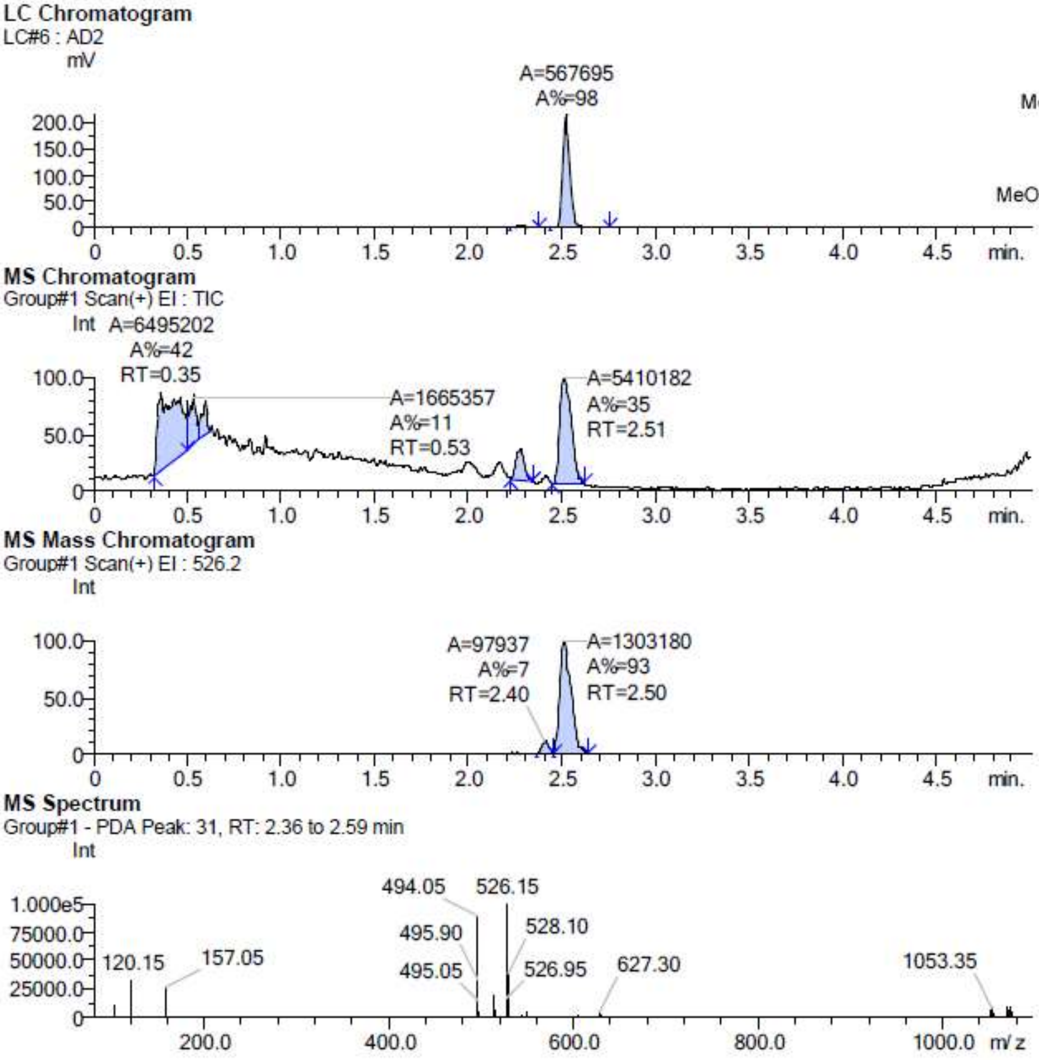

LC Chromatogram
LC#6 : AD2
mV
A=567695
A%=98
MS Chromatogram
Group#1 Scan(+) EI : TIC
Int
A=6495202
A%=42
RT=0.35
A=1665357
A%=11
RT=0.53
A=5410182
A%=35
RT=2.51
MS Mass Chromatogram
Group#1 Scan(+) EI : 526.2
Int
A=97937
A%=7
RT=2.40
A=1303180
A%=93
RT=2.50
MS Spectrum
Group#1 - PDA Peak: 31, RT: 2.36 to 2.59 min
Int
120.15
157.05
494.05
495.90
495.05
526.15
528.10
526.95
627.30
1053.35
min.
m/z

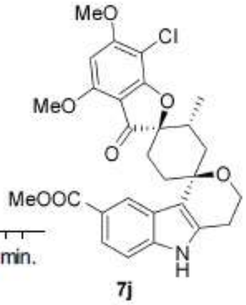

MeO
Cl
MeO
O
O
O
MeOOC
N
H

7j

LC Chromatogram
LC#6 : AD2

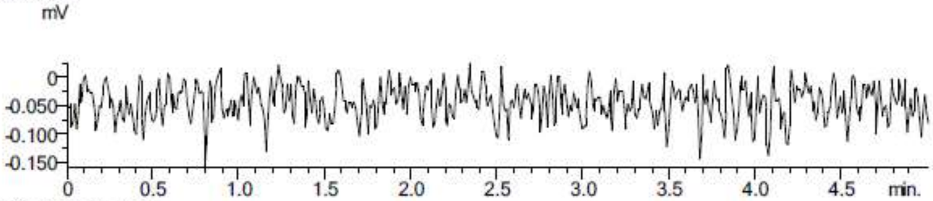

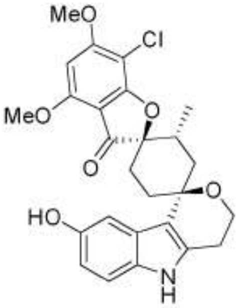

7k

MS Chromatogram
Group#1 Scan(+) EI : TIC

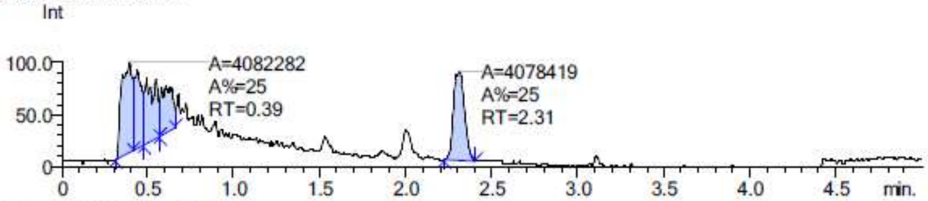

MS Mass Chromatogram
Group#1 Scan(+) EI : 484.1

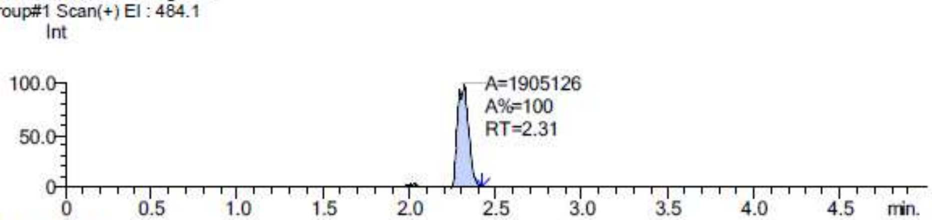

MS Spectrum
Group#1 - PDA Peak: 23, RT: 2.11 to 2.38 min

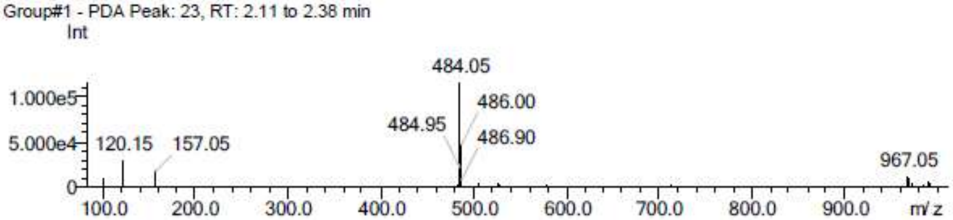

PDA Chromatogram
1: Wavelength 210 nm, Band Width 4 nm

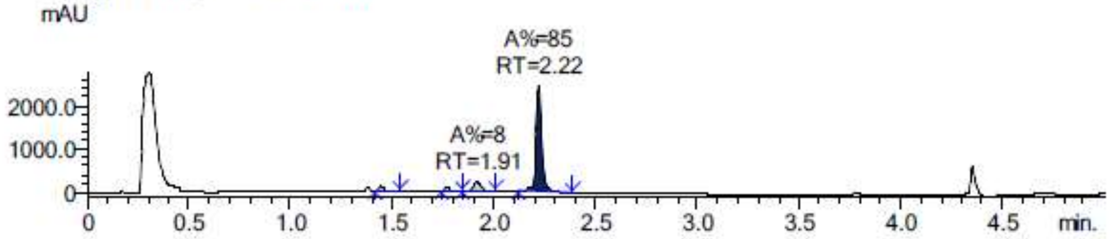

LC Chromatogram
LC#6 : AD2

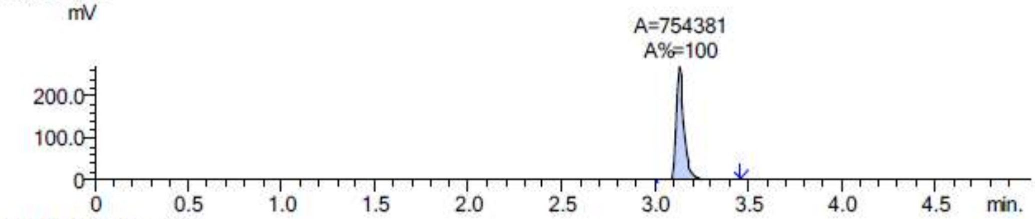

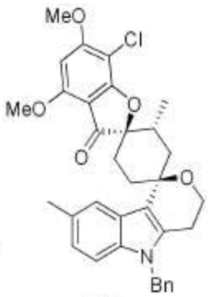

7m

MS Chromatogram
Group#1 Scan(+) EI : TIC

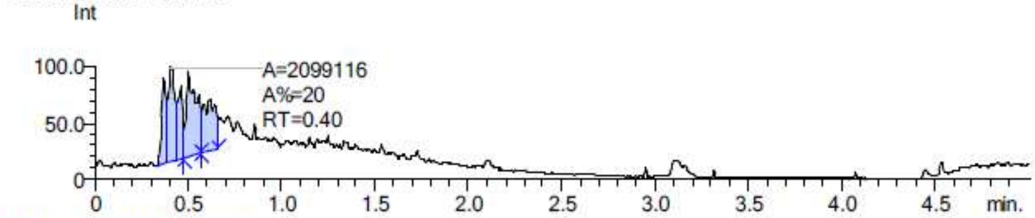

MS Mass Chromatogram
Group#1 Scan(+) EI : 572.2

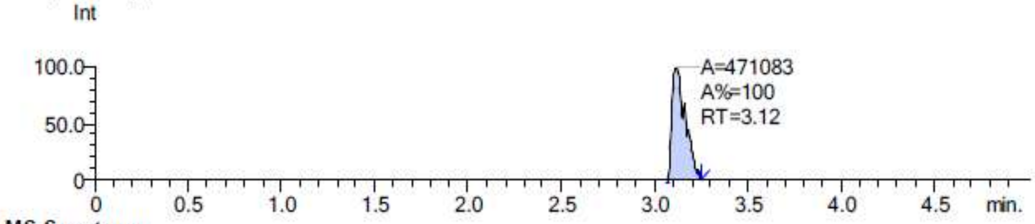

MS Spectrum
Group#1 - PDA Peak: 15, RT: 3.00 to 3.16 min

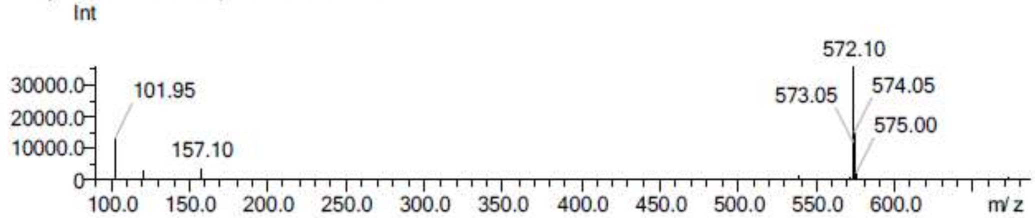

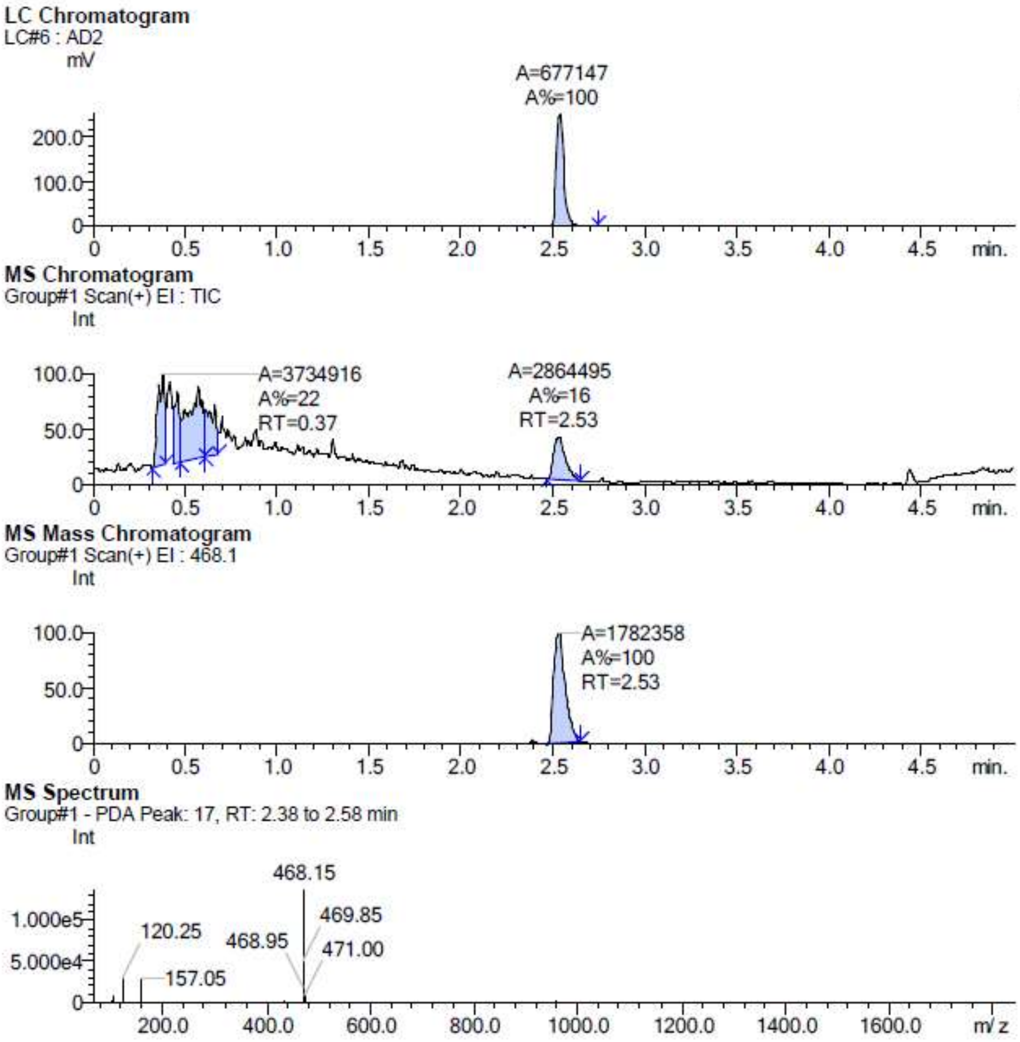
LC Chromatogram
LC#6 : AD2
mV
A=677147
A%=100
200.0
100.0
0
0.5
1.0
1.5
2.0
2.5
3.0
3.5
4.0
4.5
min.
MS Chromatogram
Group#1 Scan(+) EI : TIC
Int
100.0
50.0
A=3734916
A%=22
RT=0.37
A=2864495
A%=16
RT=2.53
MS Mass Chromatogram
Group#1 Scan(+) EI : 468.1
Int
A=1782358
A%=100
RT=2.53
MS Spectrum
Group#1 - PDA Peak: 17, RT: 2.38 to 2.58 min
Int
468.15
1.000e5
5.000e4
120.25
157.05
468.95
469.85
471.00
200.0
400.0
600.0
800.0
1000.0
1200.0
1400.0
1600.0
m/z

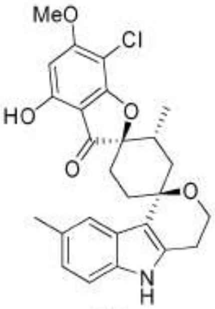
MeO
Cl
HO
O
O
O
N
H

7n

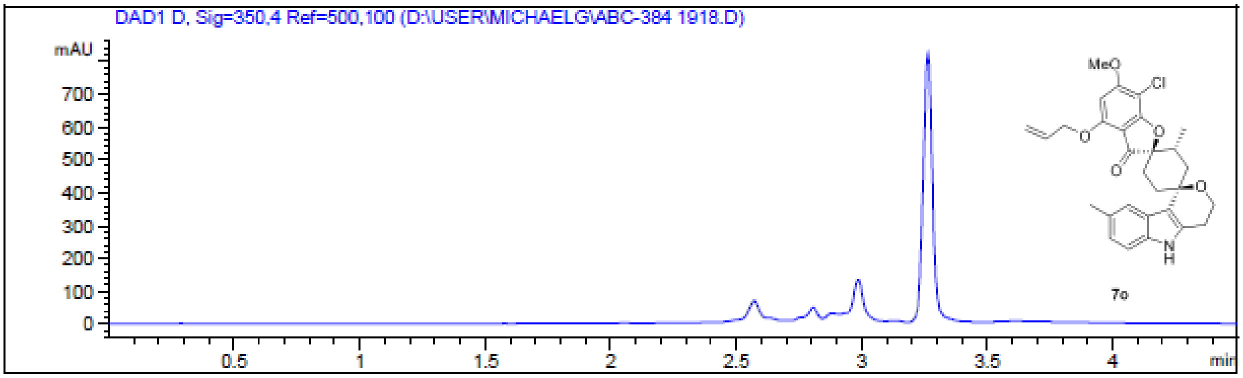

#	Time	Area	Height	Width	Area%	Symmetry
1	2.574	142	56.3	0.042	5.347	1.112
2	2.808	99.5	37.7	0.044	3.746	1.729
3	2.884	29.1	12.1	0.04	1.095	1.083
4	2.988	261.3	108.9	0.04	9.841	0.822
5	3.266	2123.4	824.5	0.0429	79.971	1.004

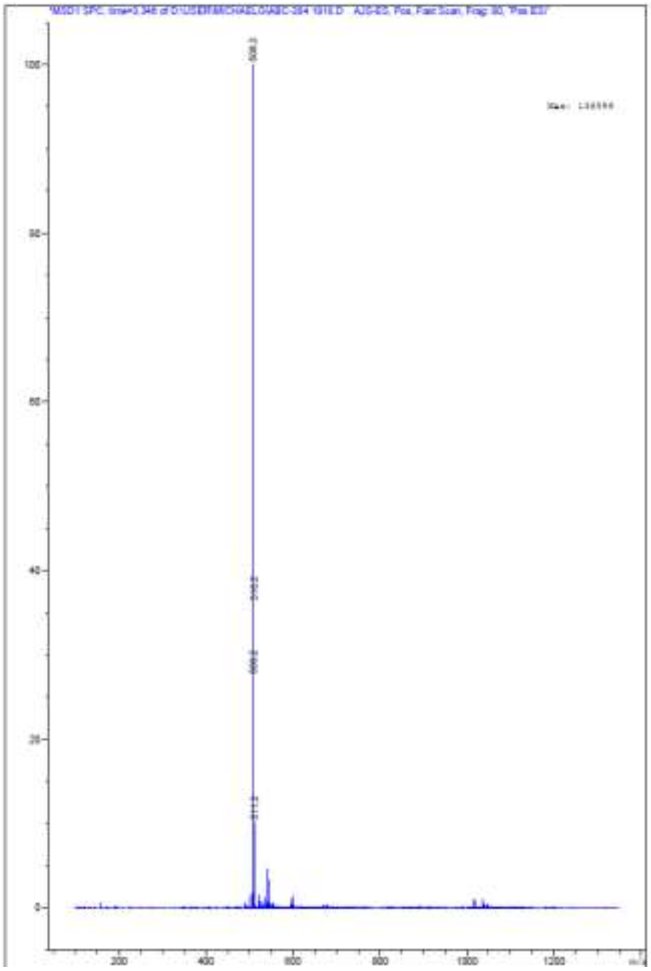

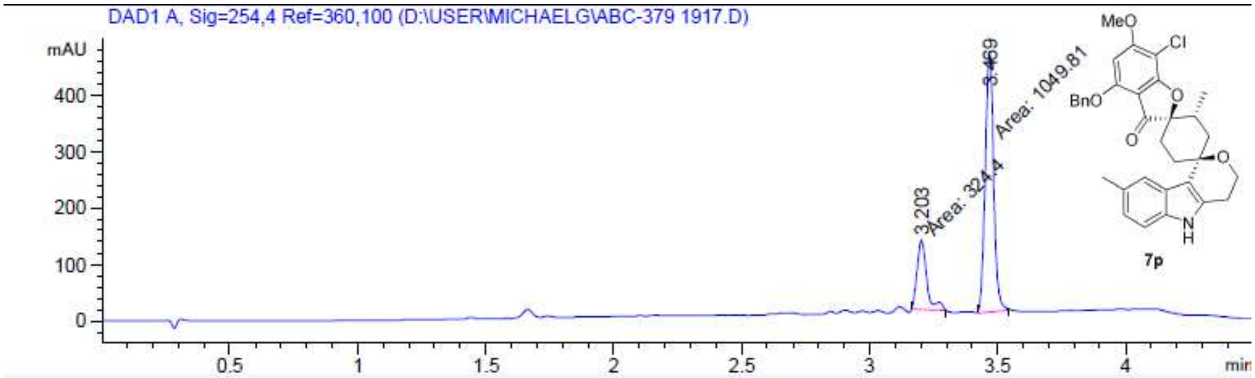

#	Time	Area	Height	Width	Area%	Symmetry
1	3.203	324.4	122.5	0.0441	23.606	0.795
2	3.469	1049.8	460.9	0.038	76.394	1.035

MS Spectrum

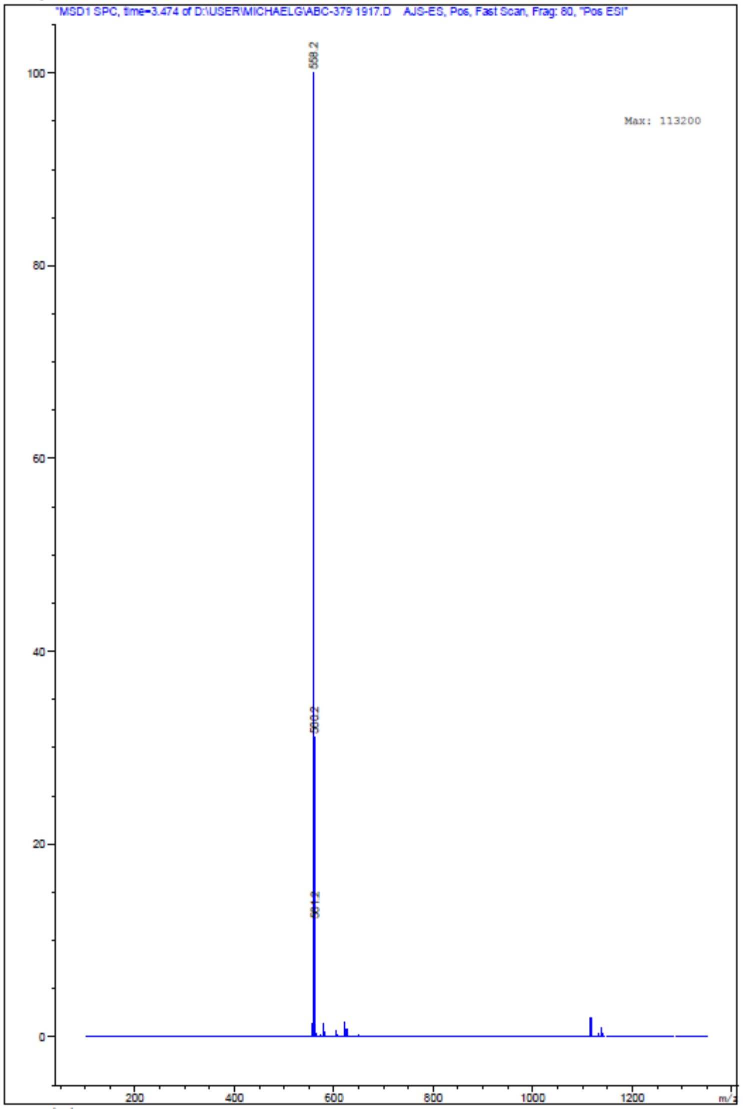

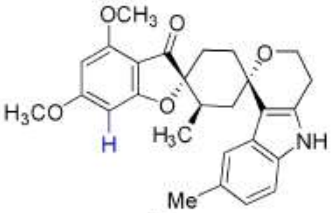

7q

LC Chromatogram
LC#6 : AD2

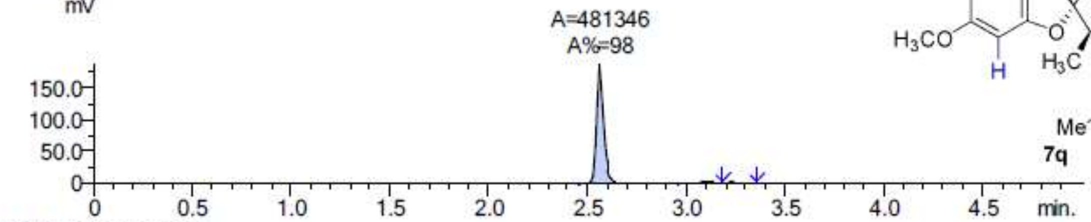

MS Chromatogram
Group#1 Scan(+) EI : TIC

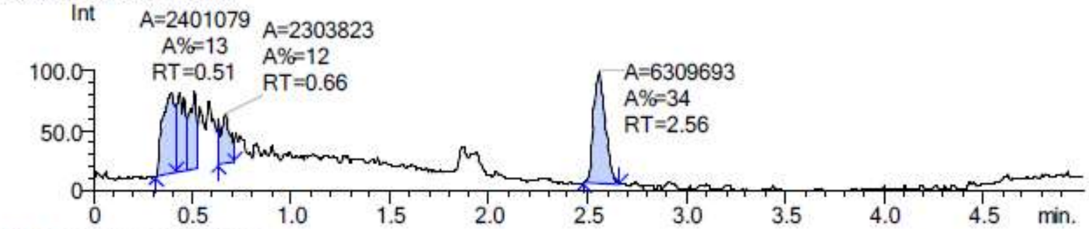

MS Mass Chromatogram
Group#1 Scan(+) EI : 448.2

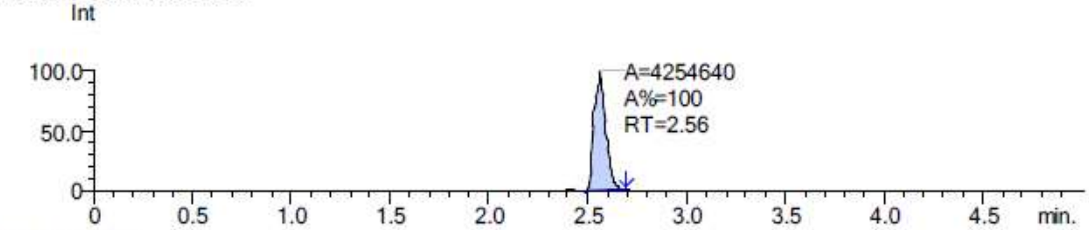

MS Spectrum
Group#1 - PDA Peak: 22, RT: 2.37 to 2.62 min

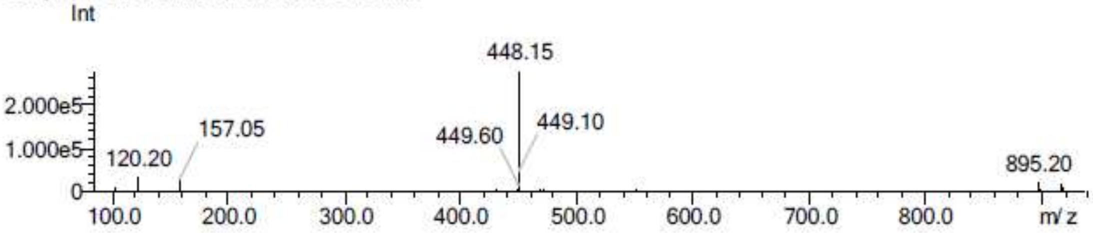

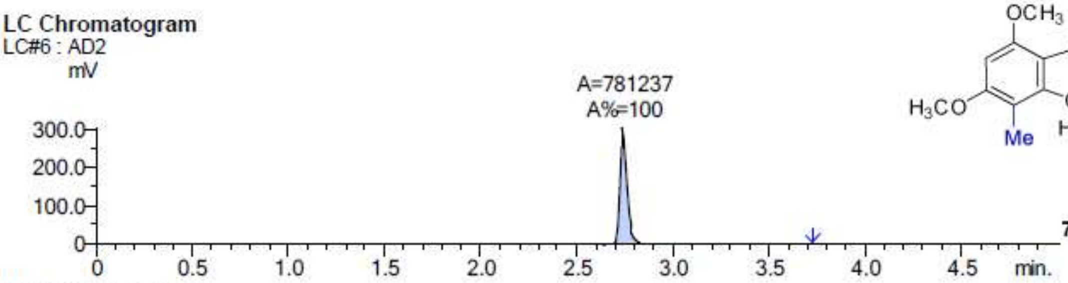

LC Chromatogram
LC#6 : AD2
mV
A=781237
A%=100
min.

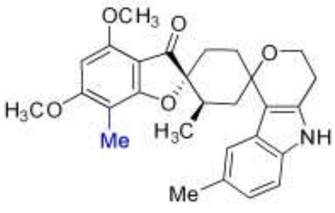

OCH3
O
H3CO
O
Me
H3C
O
NH
Me

7r

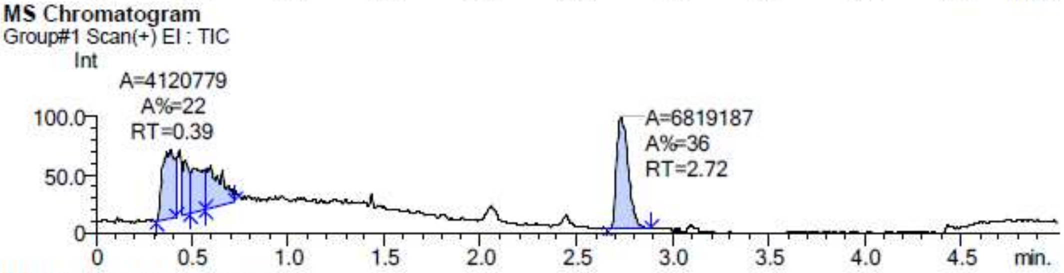

MS Chromatogram
Group#1 Scan(+) EI : TIC
Int
A=4120779
A%=22
RT=0.39
A=6819187
A%=36
RT=2.72
min.

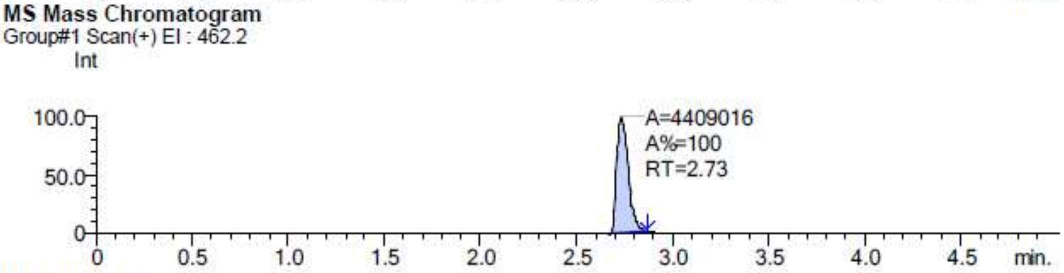

MS Mass Chromatogram
Group#1 Scan(+) EI : 462.2
Int
A=4409016
A%=100
RT=2.73
min.

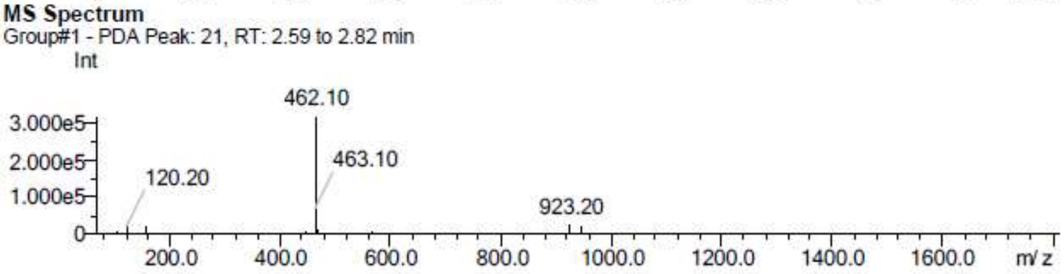

MS Spectrum
Group#1 - PDA Peak: 21, RT: 2.59 to 2.82 min
Int
462.10
463.10
120.20
923.20
m/z

LC Chromatogram
LC#6 : AD2

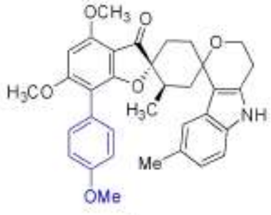

7s

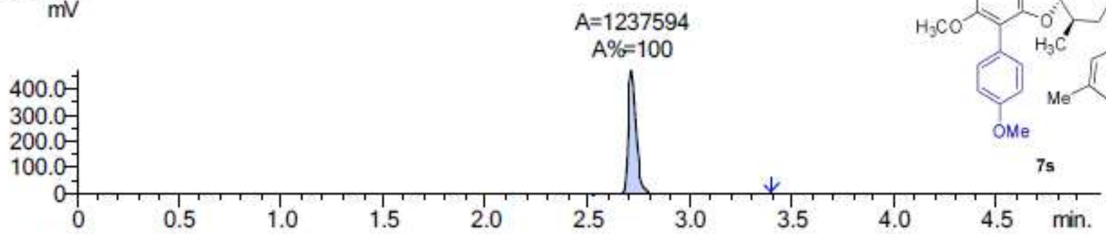

MS Chromatogram
Group#1 Scan(+) EI : TIC

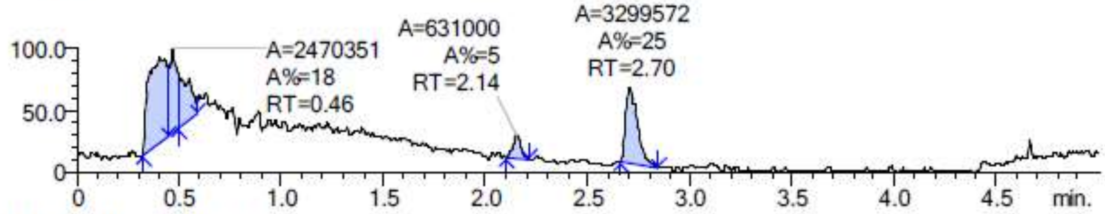

MS Mass Chromatogram
Group#1 Scan(+) EI : 554.2

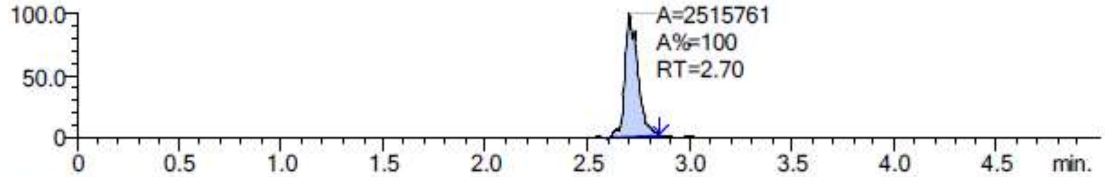

MS Spectrum
Group#1 - PDA Peak: 17, RT: 2.58 to 2.76 min

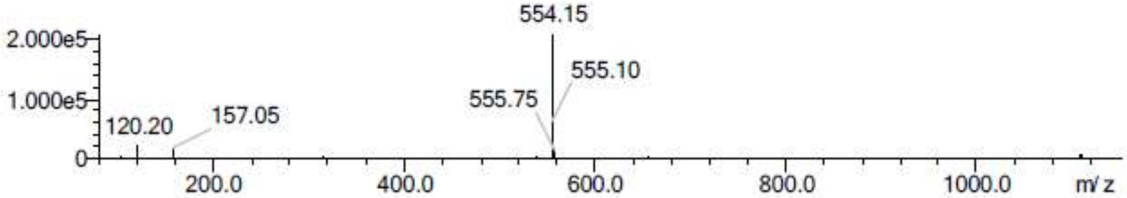

LC Chromatogram
LC#6 : AD2

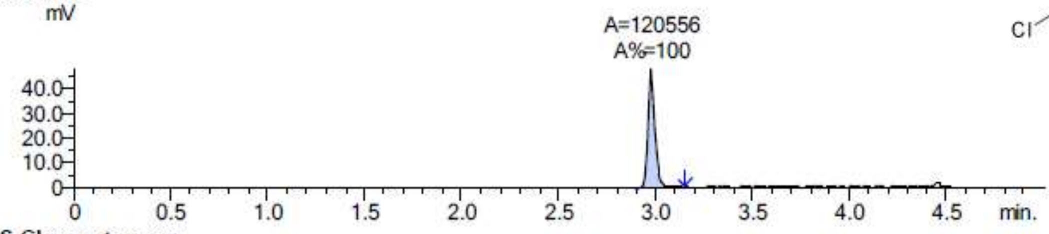

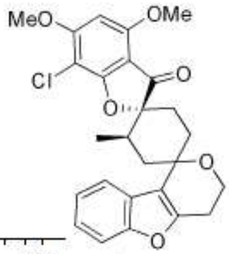

8

MS Chromatogram
Group#1 Scan(+) EI : TIC

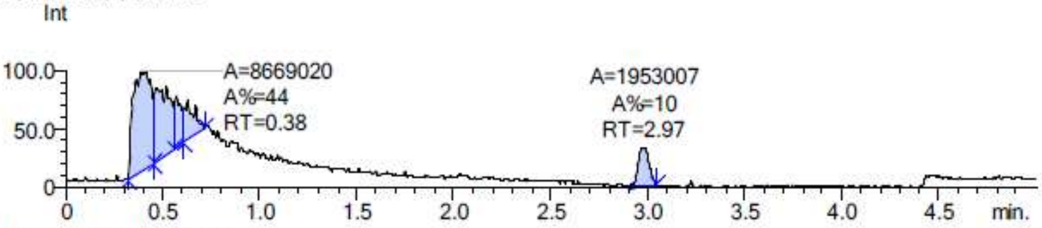

MS Mass Chromatogram
Group#1 Scan(+) EI : 469.1

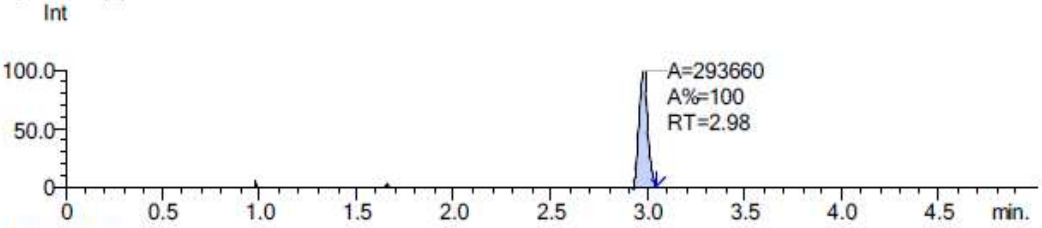

MS Spectrum
Group#1 - PDA Peak: 16, RT: 2.81 to 3.03 min

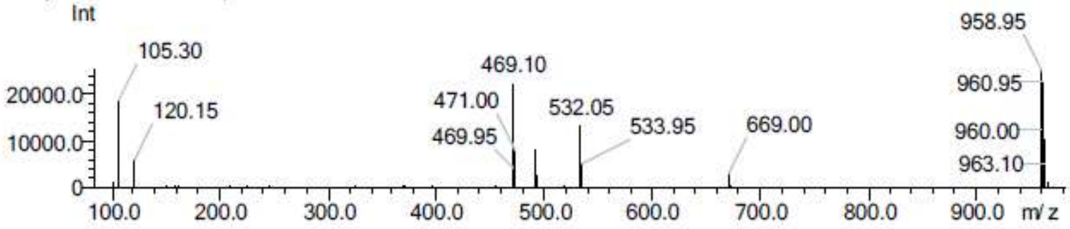

NMR spectra

(2R,2'R)-7-Chloro-4,6-dimethoxy-2'-methyl-3H-spiro[benzofuran-2,1'-cyclohexane]-3,4'-dione **5a**

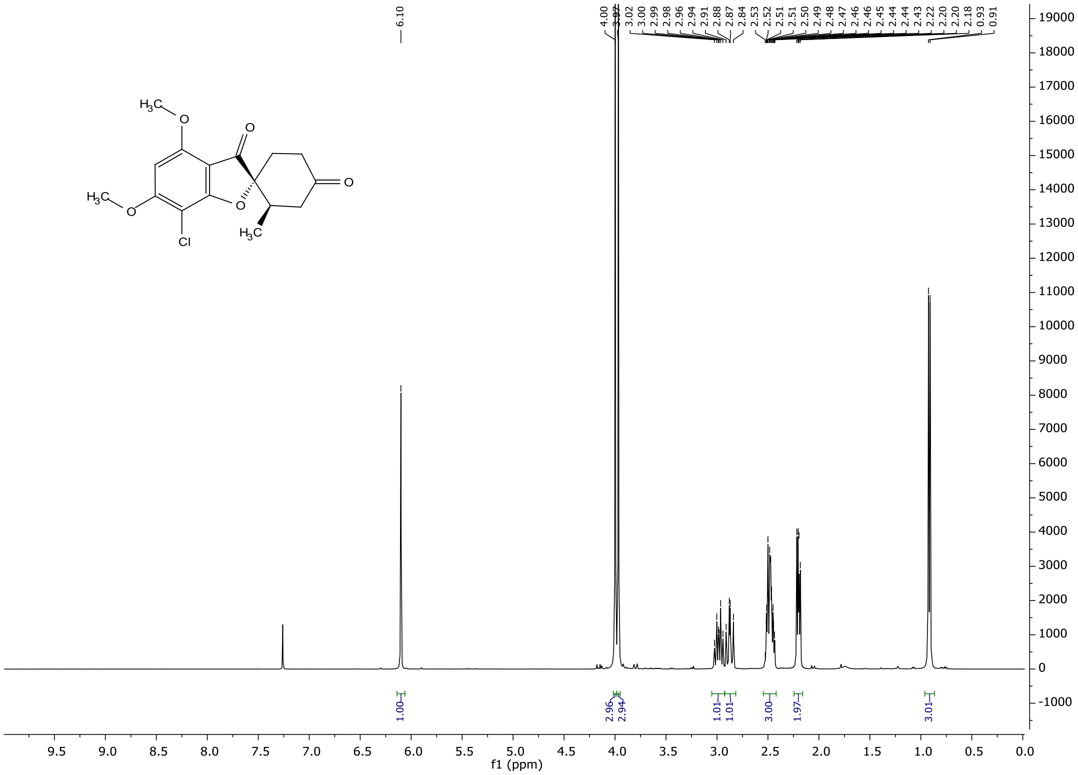

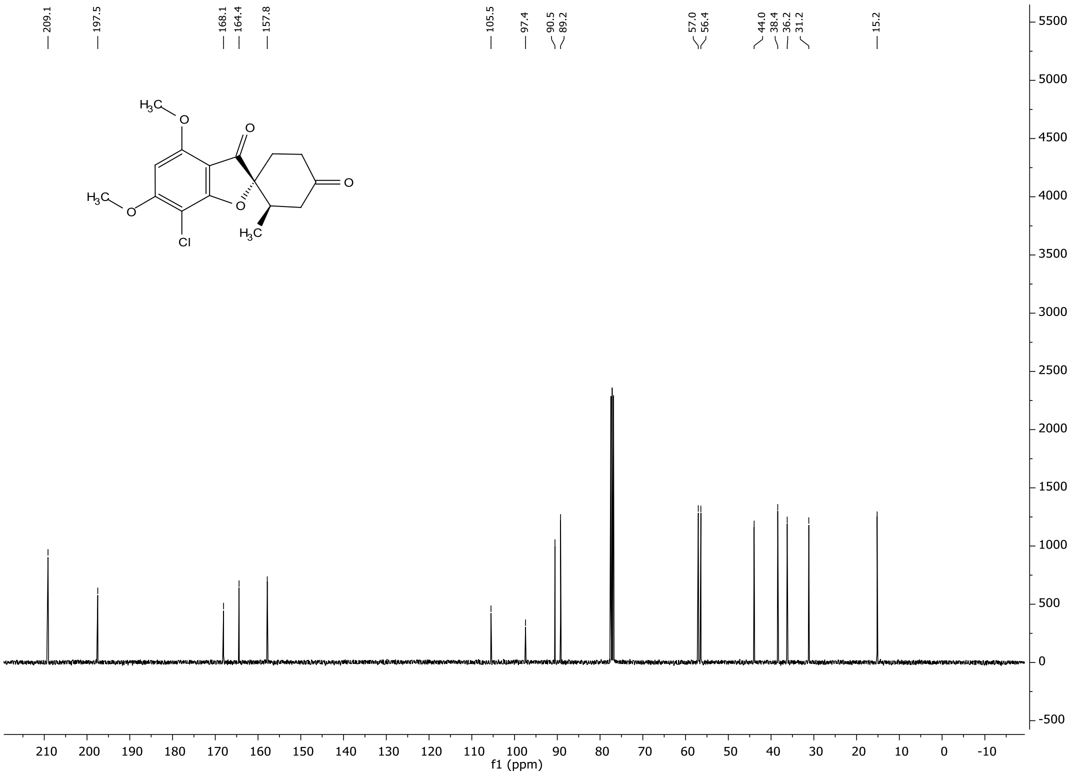

(2*R*,2'*R*)-7-chloro-4-hydroxy-6-methoxy-2'-methyl-3H-spiro[benzofuran-2,1'-cyclohexane]-3,4'-dione **5b**

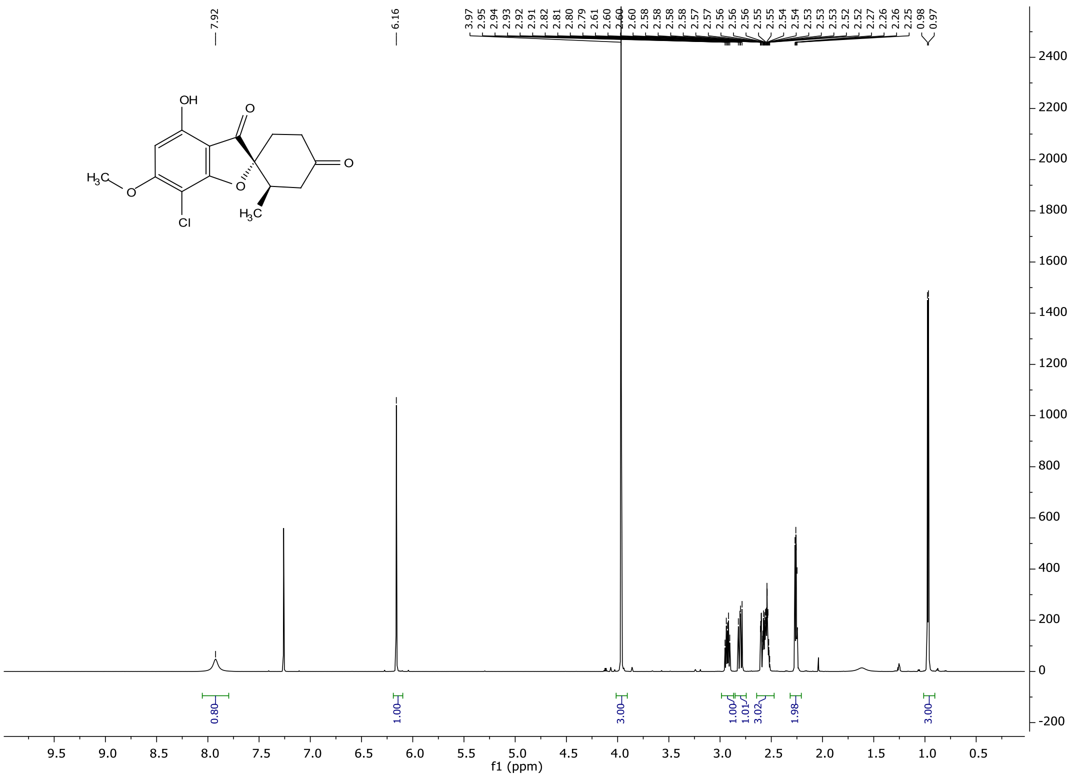

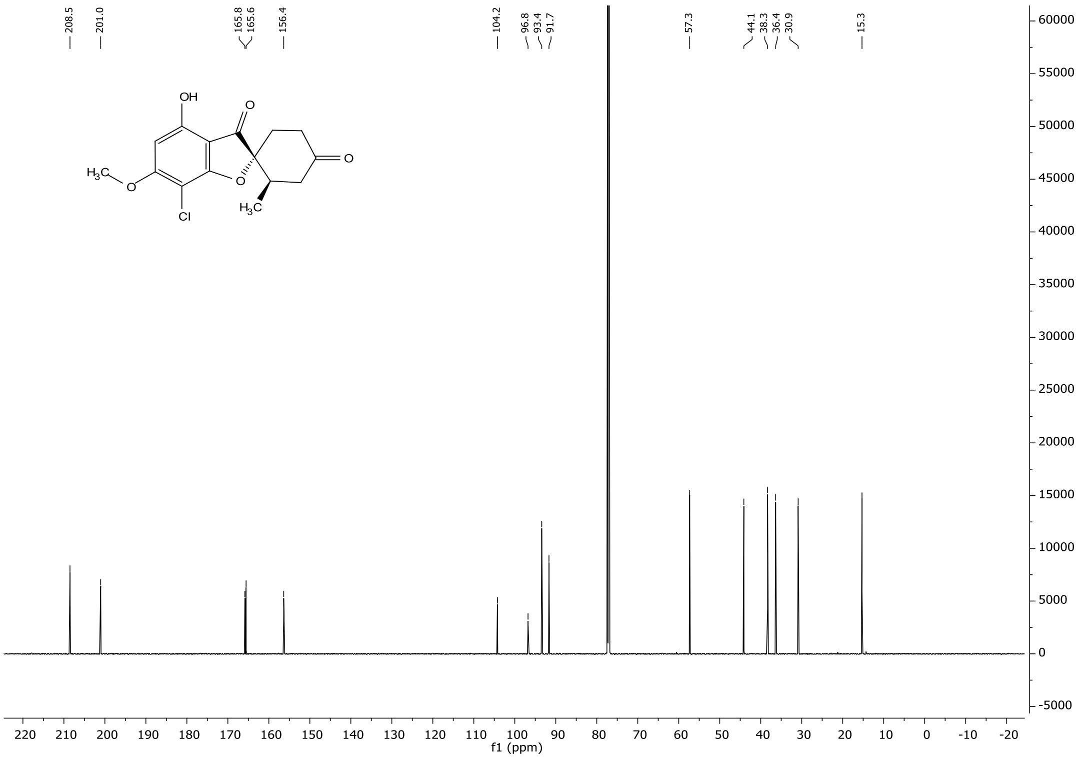

(2*R*,2'*R*)-4-(allyloxy)-7-chloro-2',6-dimethyl-3*H*-spiro[benzofuran-2,1'-cyclohexane]-3,4'-dione **5c**

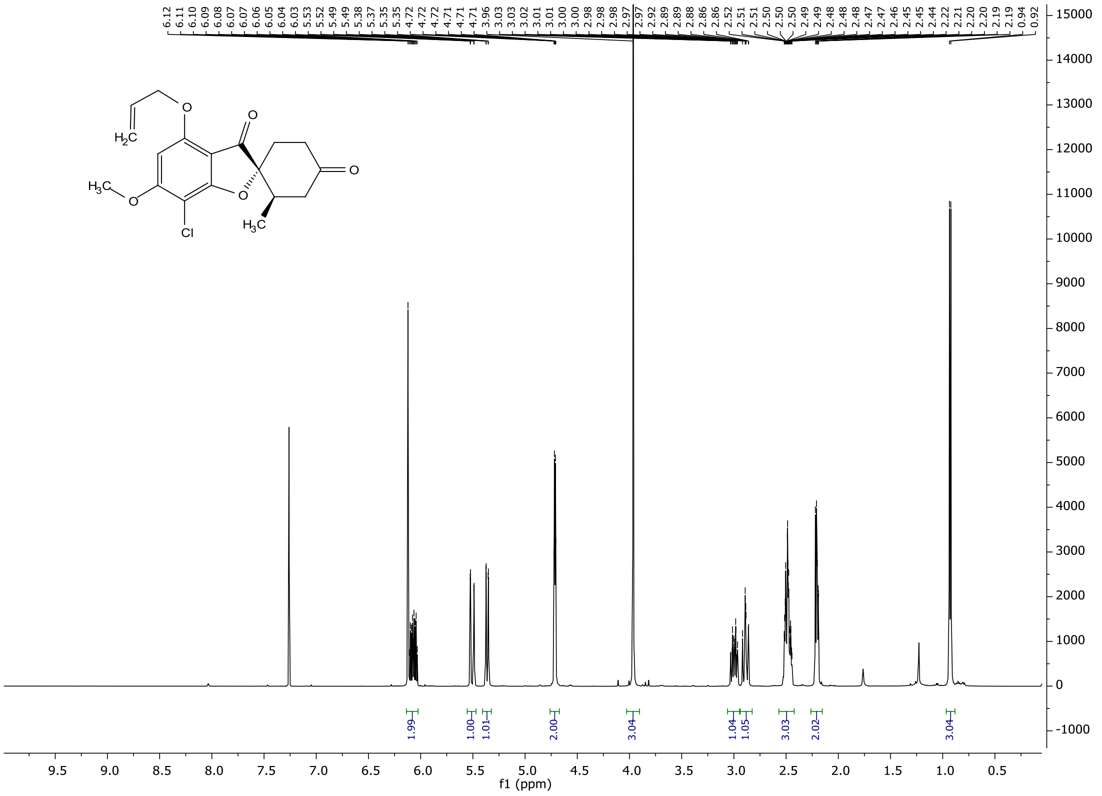

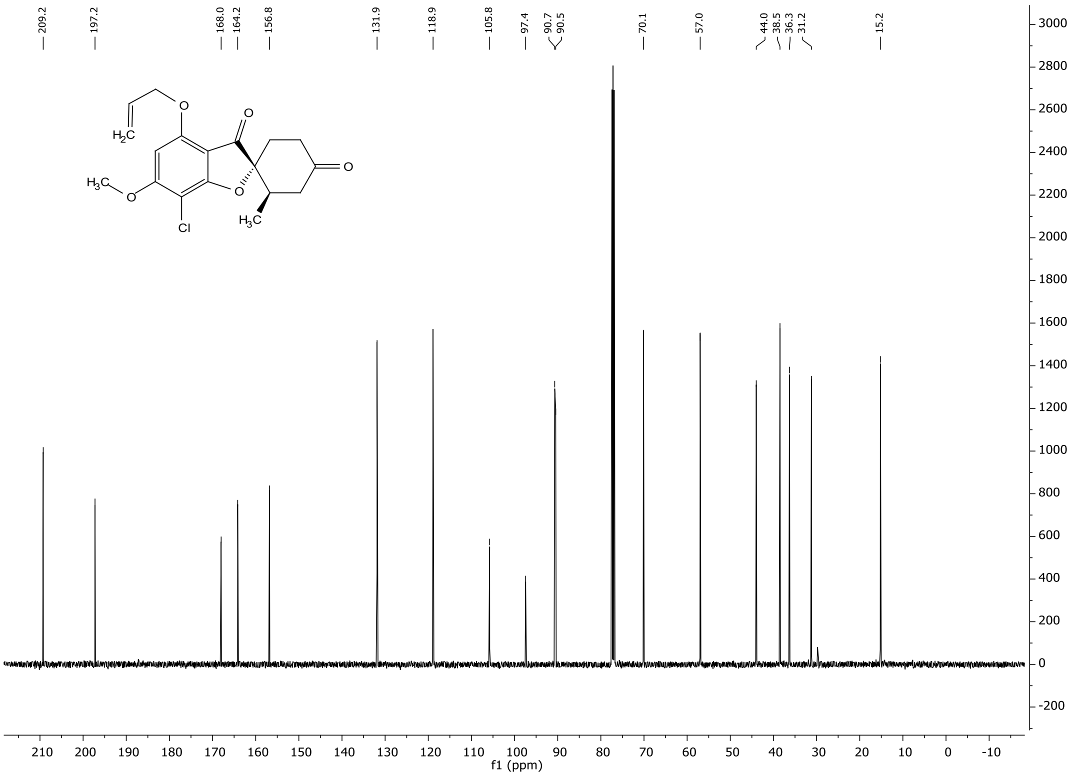

(2*R*,2'*R*)-4-(benzyloxy)-7-chloro-2',6-dimethyl-3H-spiro[benzofuran-2,1'-cyclohexane]-3,4'-dione

5d

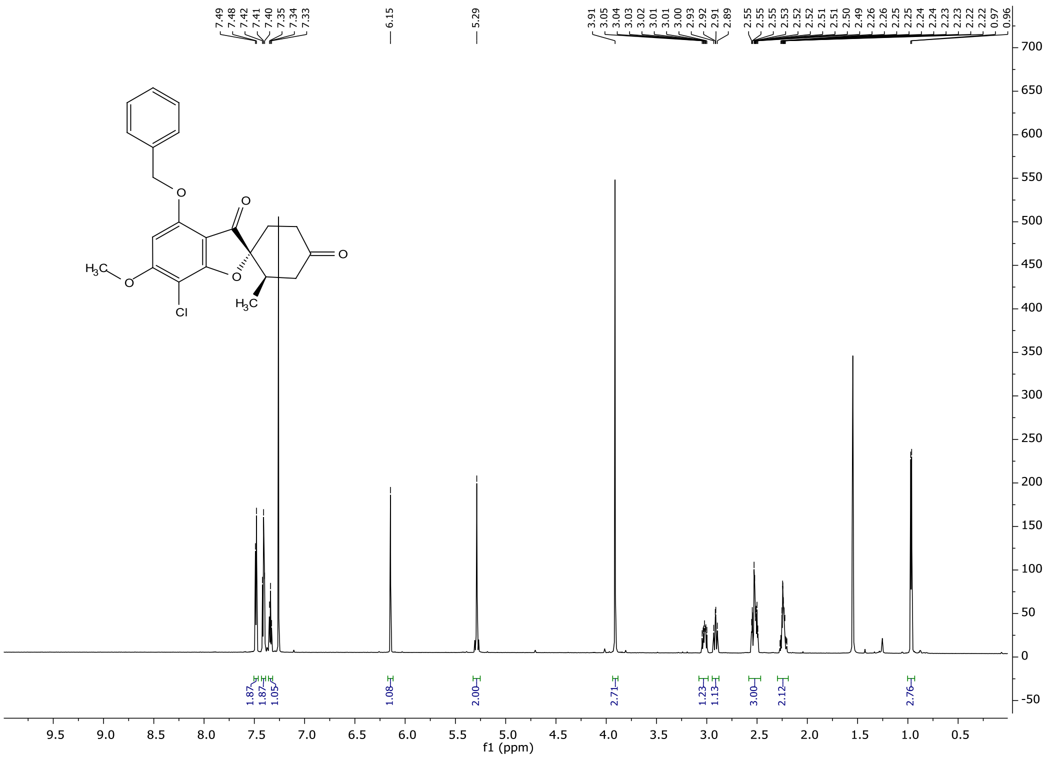

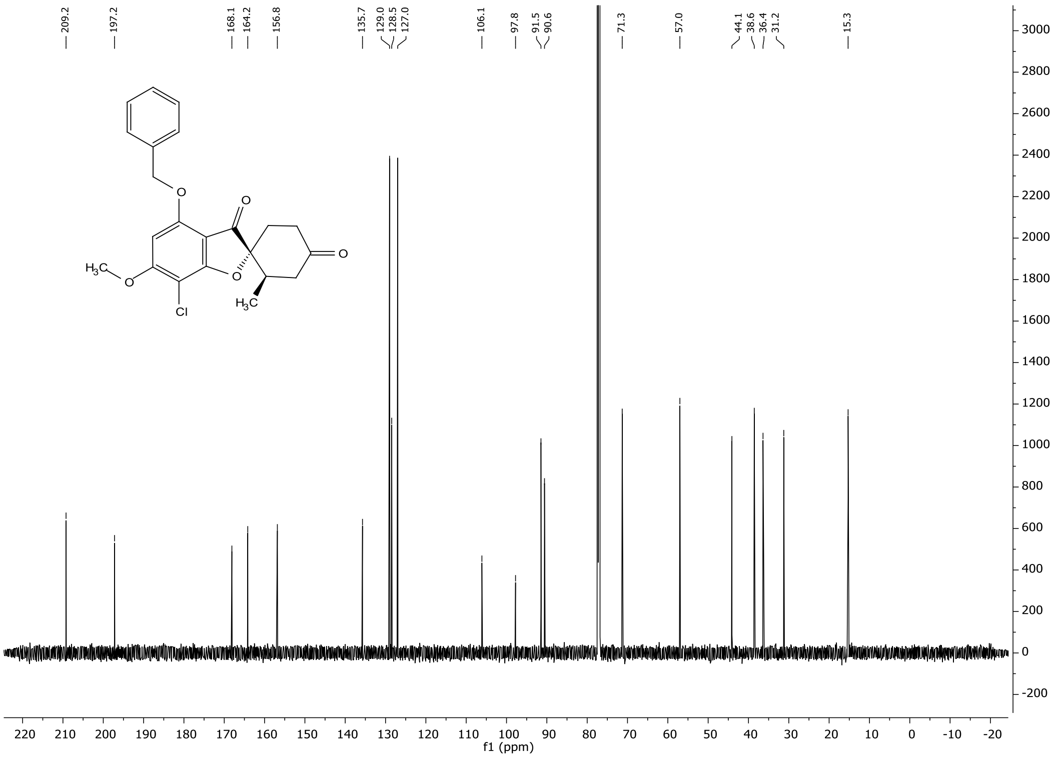

(2*R*,2'*R*)-4,6-dimethoxy-2',7-dimethyl-3H-spiro[benzofuran-2,1'-cyclohexane]-3,4'-dione **5e**

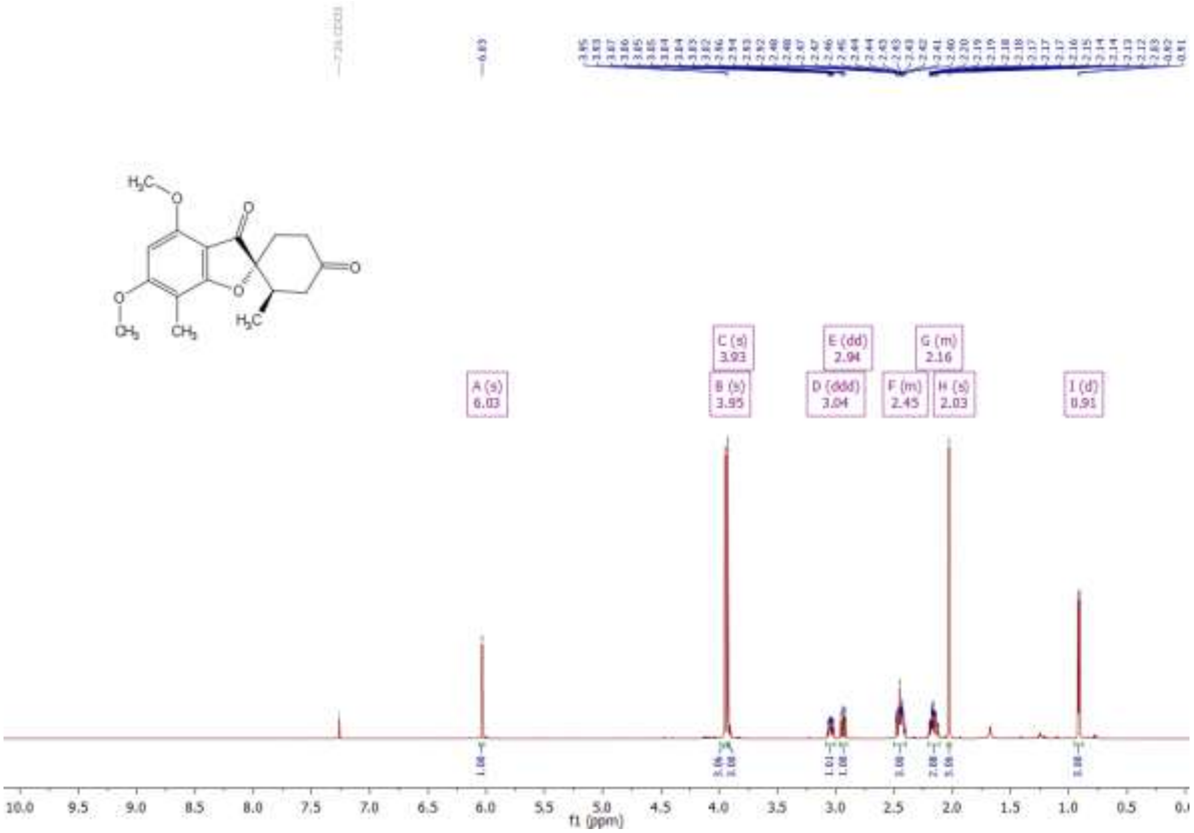

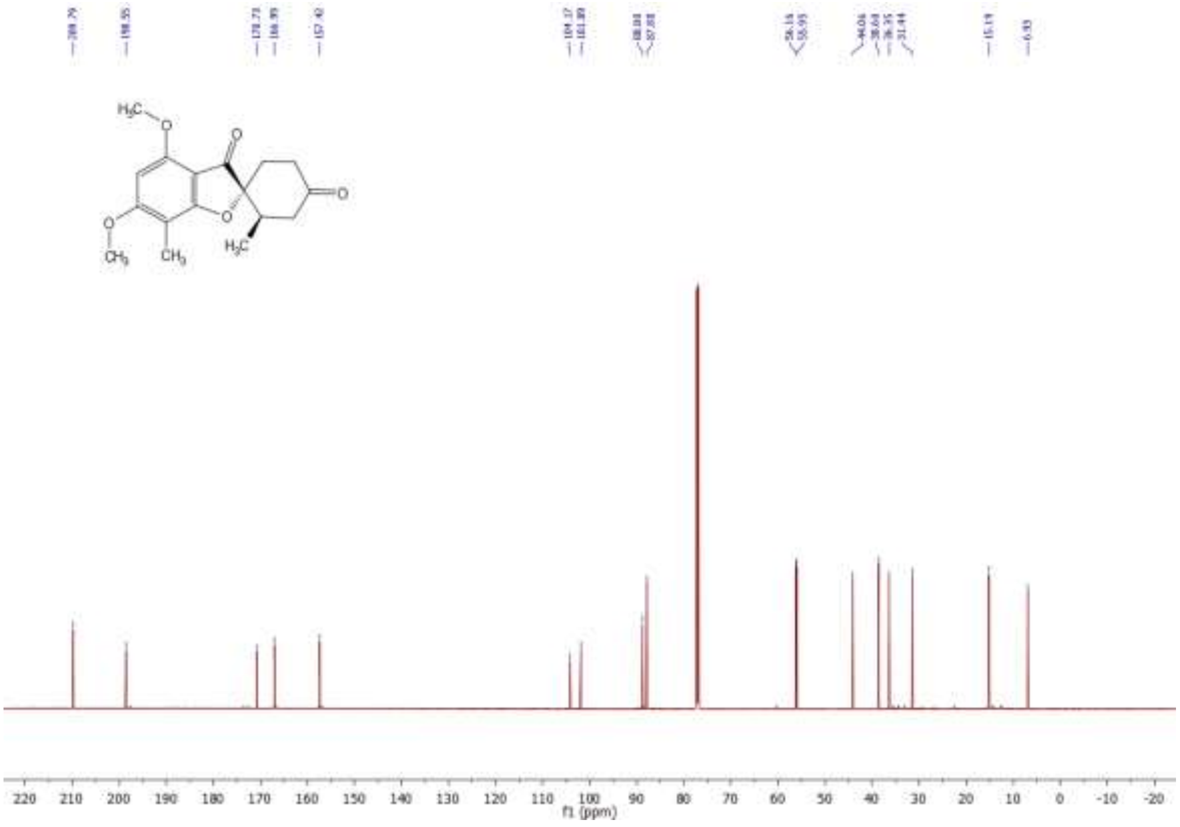

(2*R*,2'*R*)-4,6-dimethoxy-7-(4-methoxyphenyl)-2'-methyl-3H-spiro[benzofuran-2,1'-cyclohexane]-3,4'-dione **5f**

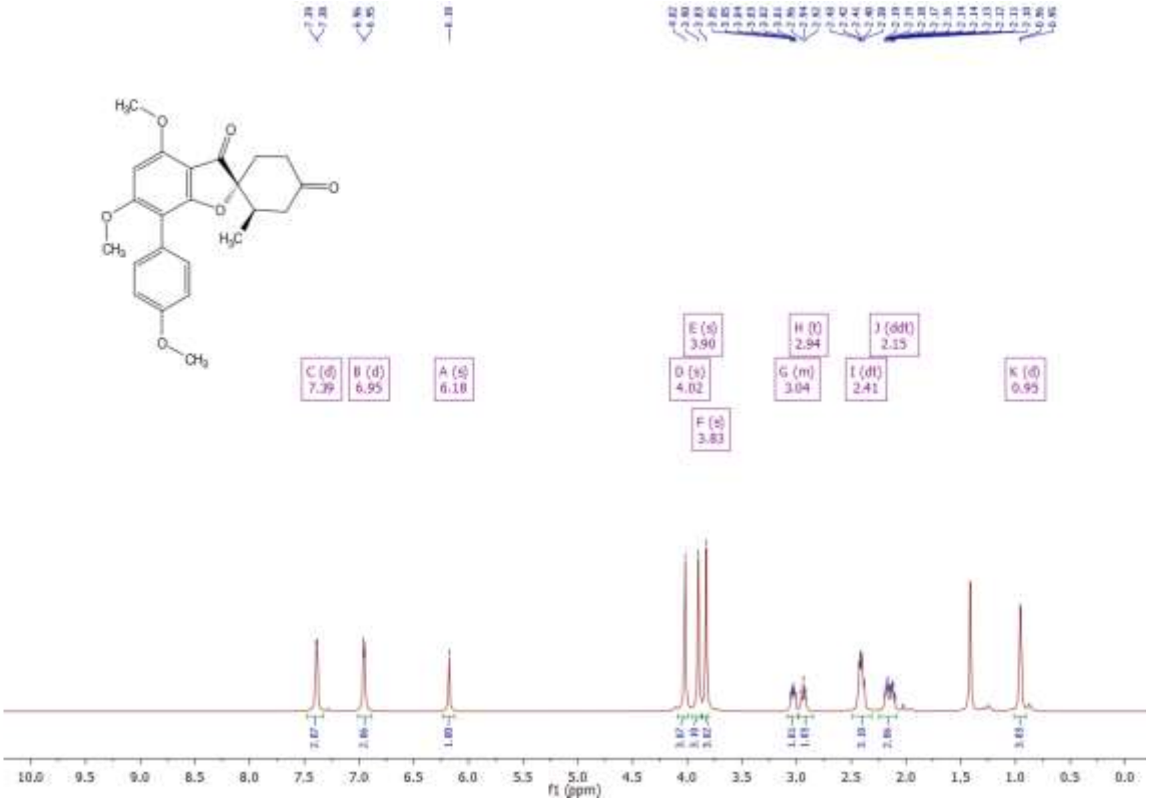

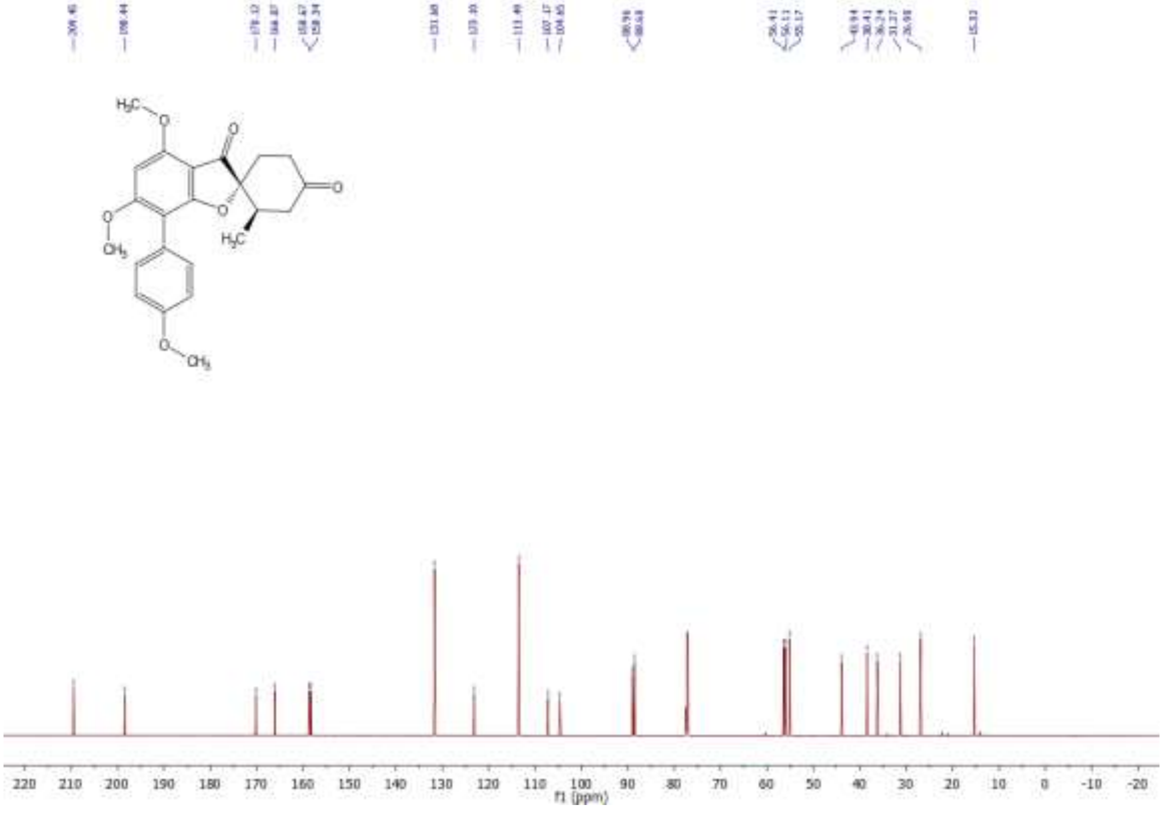

4',5'-dihydro-3'H-spiro[cyclopentane-1,1'-pyrano[4,3-b]indole] **3a**

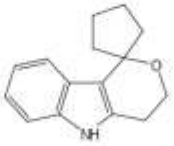

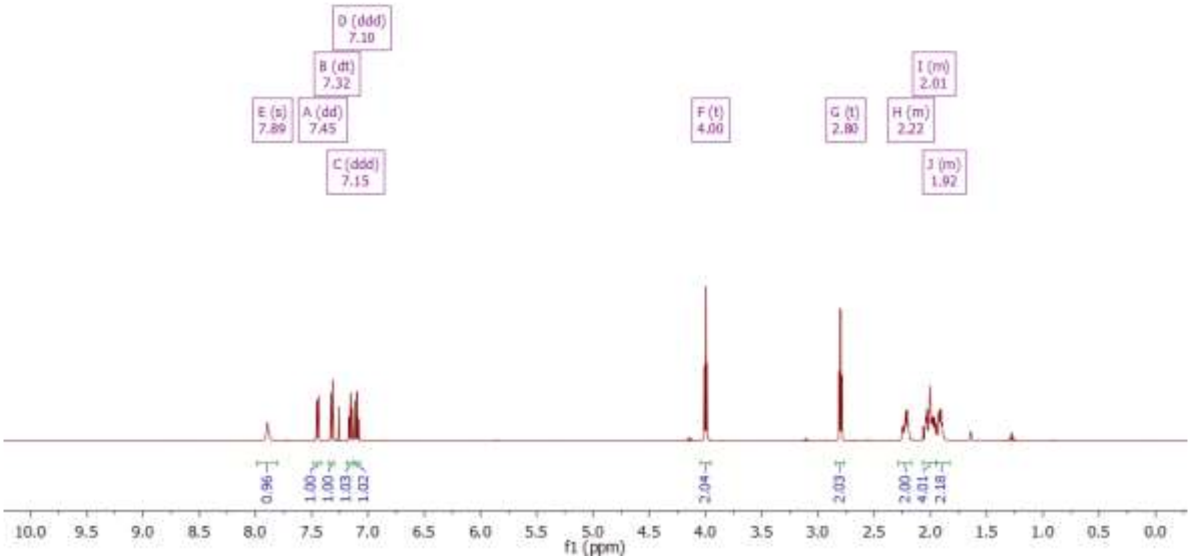

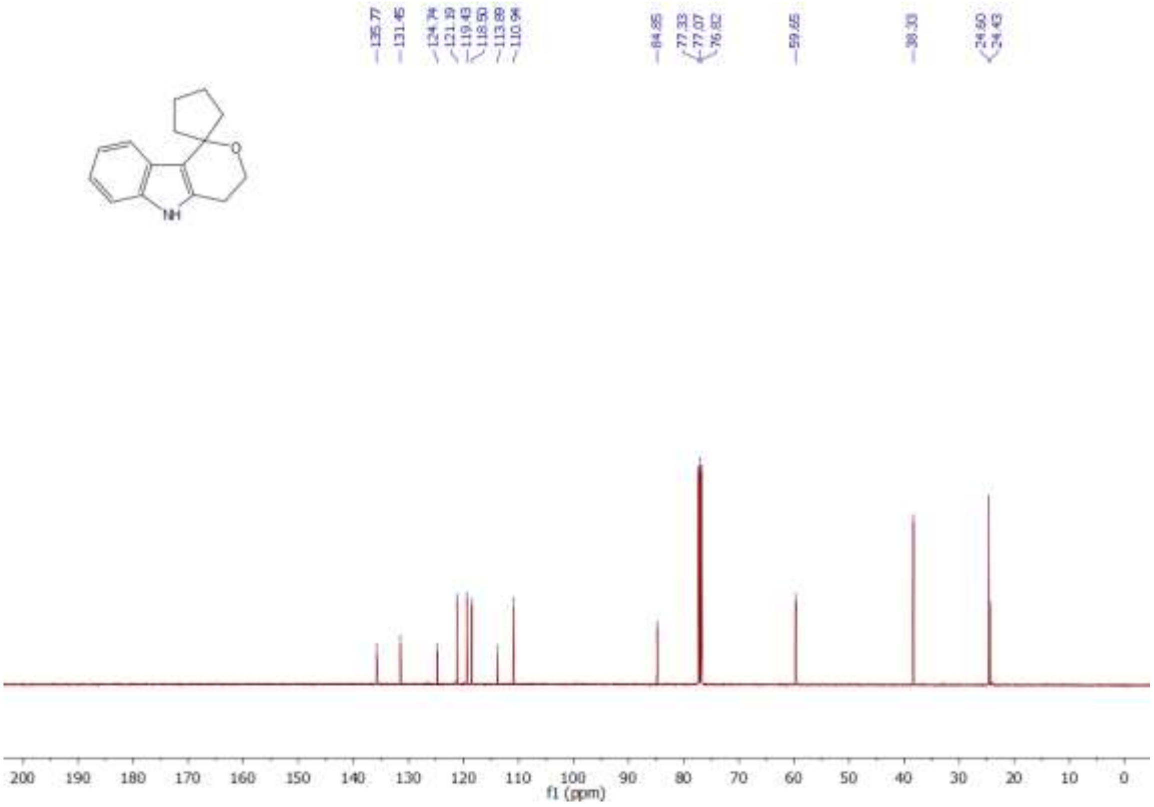

4',5'-dihydro-3'*H*-spiro[cyclohexane-1,1'-pyrano[4,3-*b*]indole] **3b**

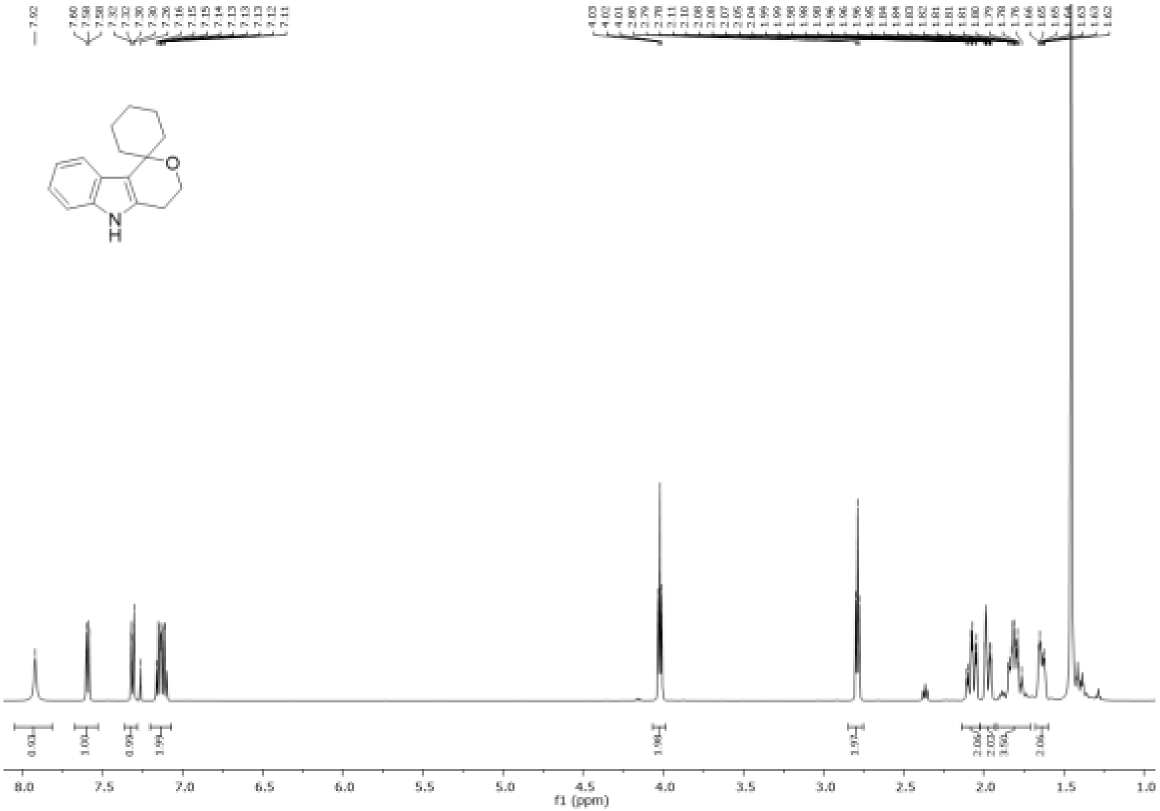

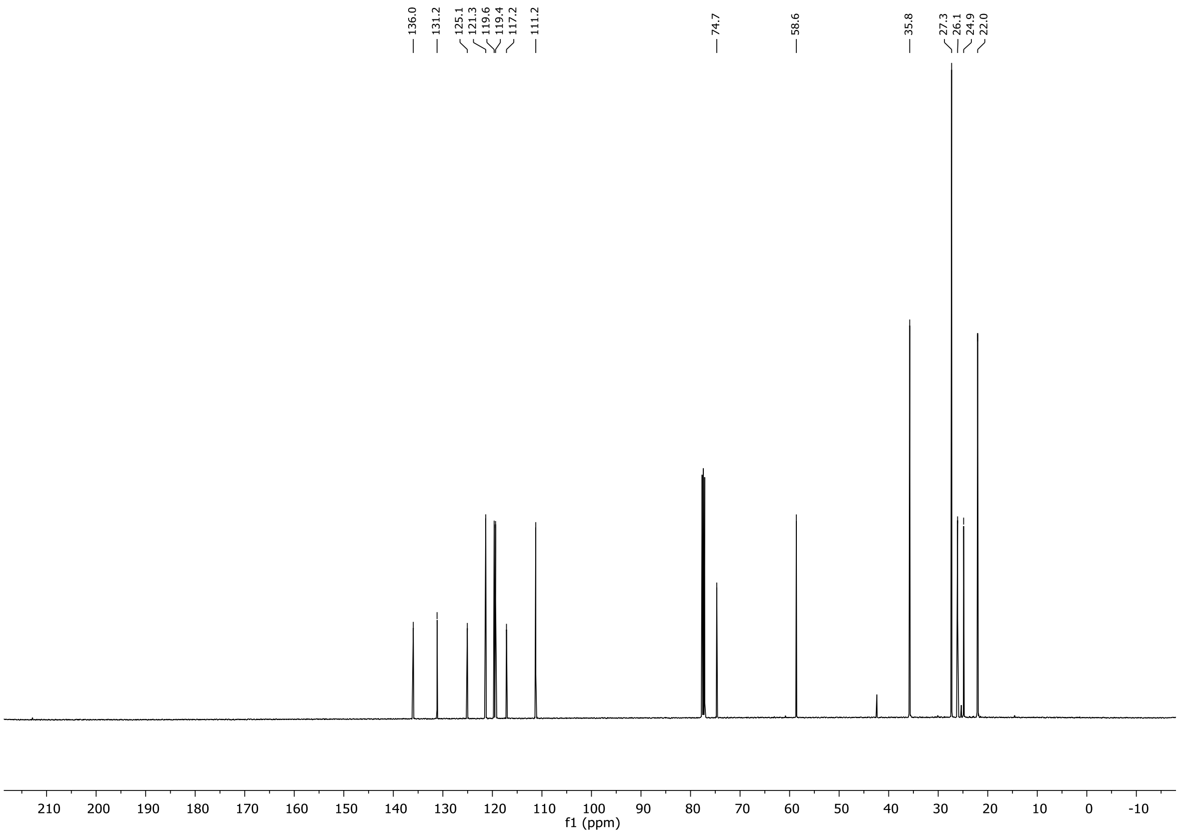

4',5'-dihydro-3'H-spiro[cycloheptane-1,1'-pyrano[4,3-b]indole] **3c**

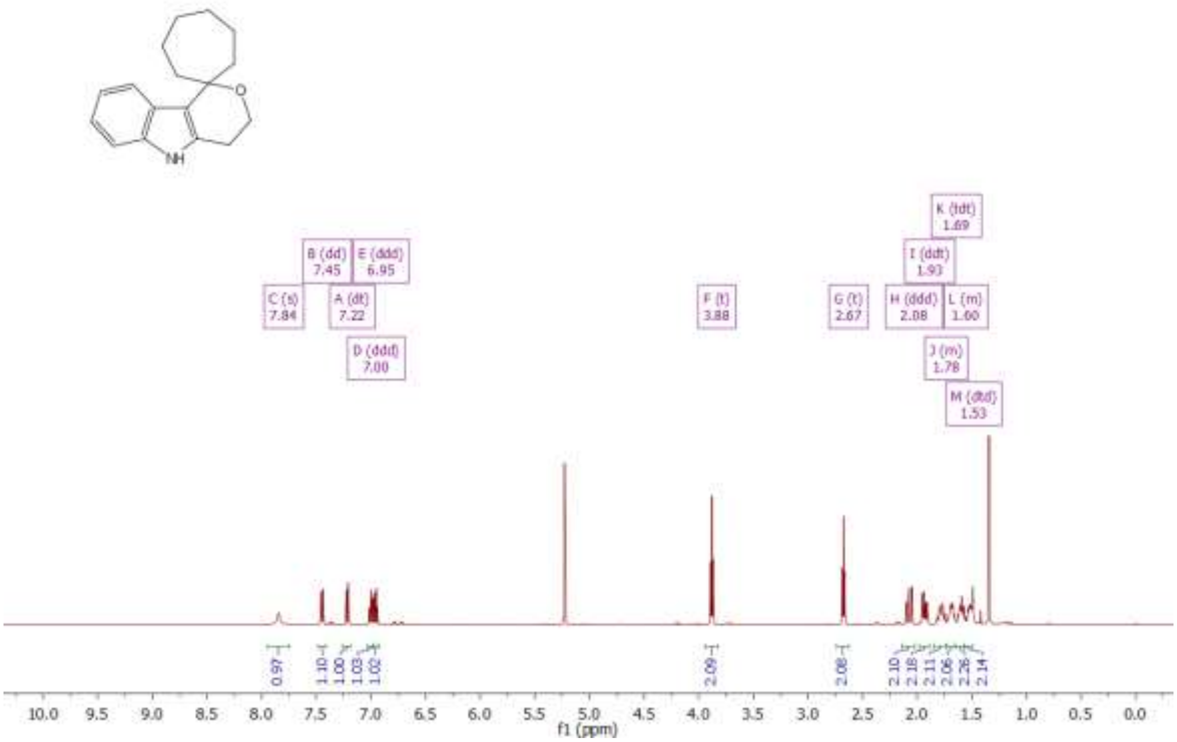

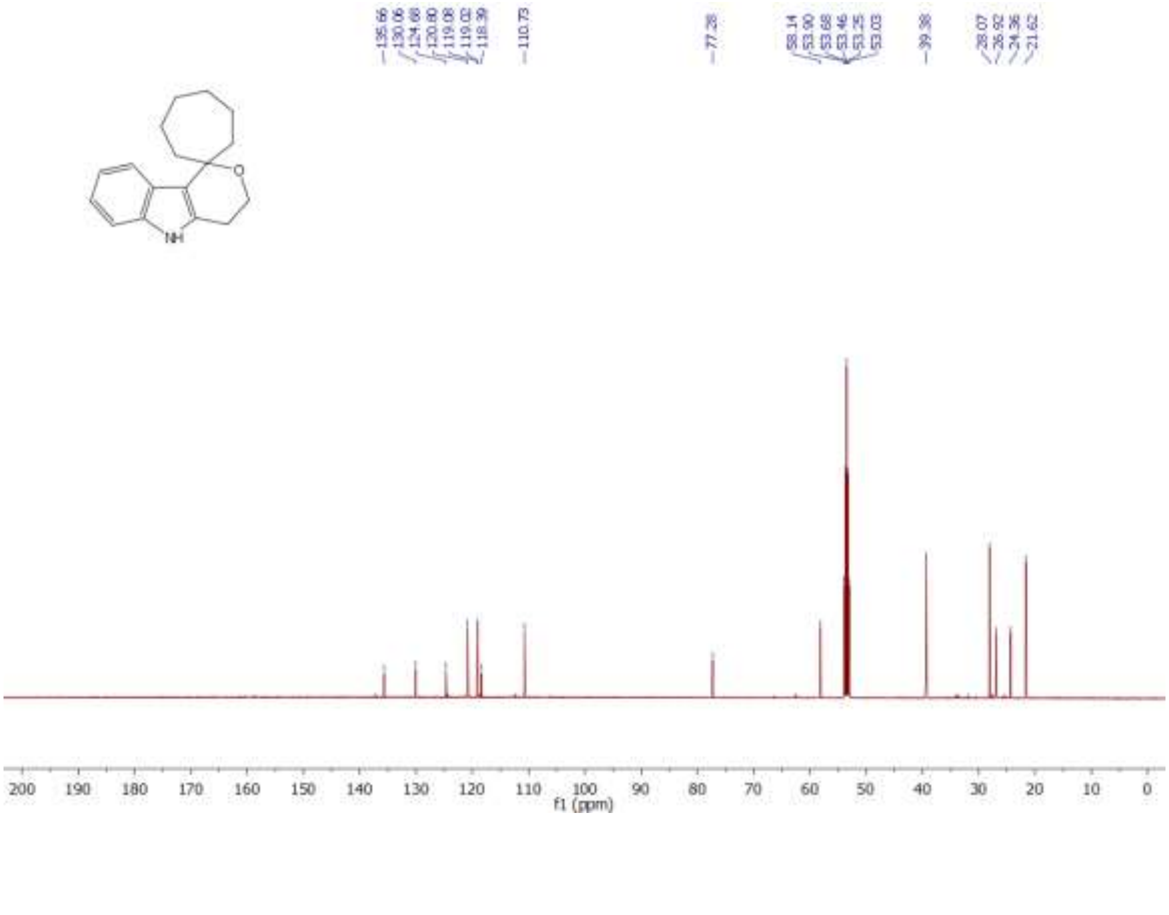

(3*S*)-3-methyl-4',5'-dihydro-3'*H*-spiro[cyclohexane-1,1'-pyrano[4,3-*b*]indole] **3d** (10:1 dr)

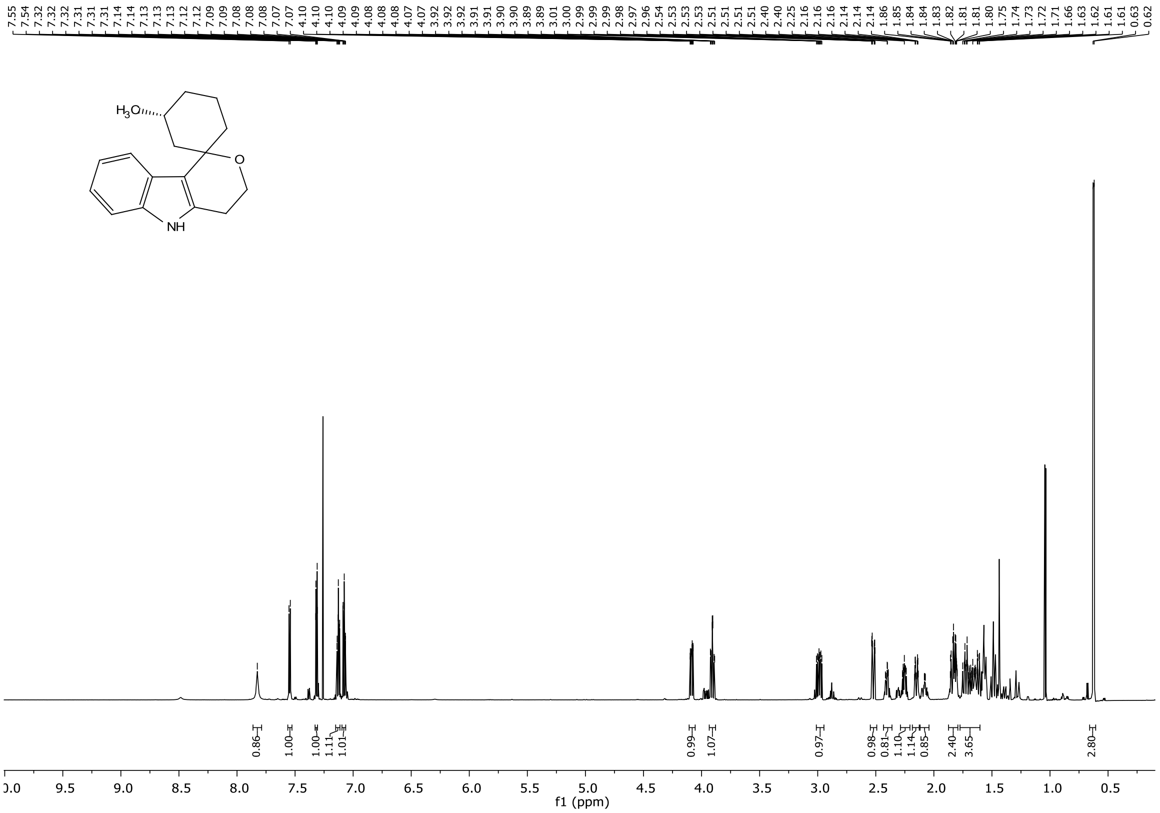

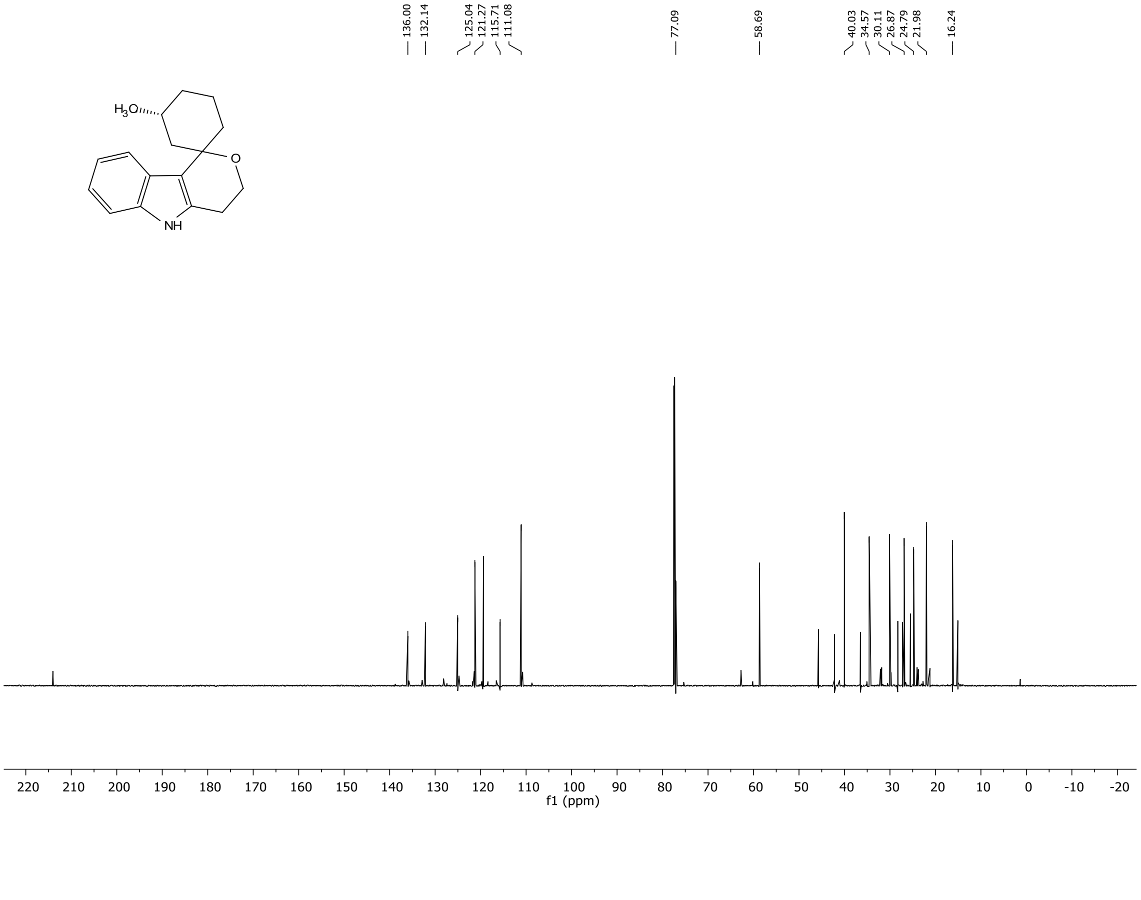

(2*S*,5*R*)-2-isopropyl-5-methyl-4',5'-dihydro-3'*H*-spiro[cyclohexane-1,1'-pyrano[4,3-*b*]indole] **3e**

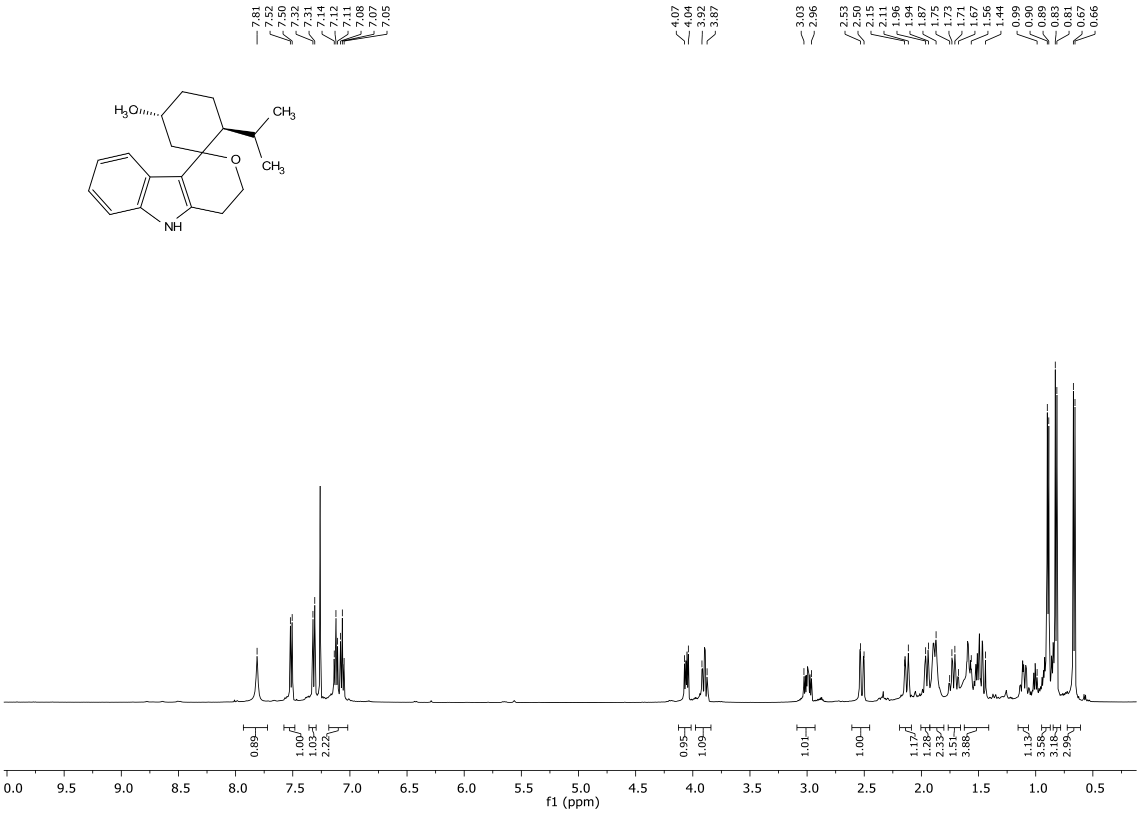

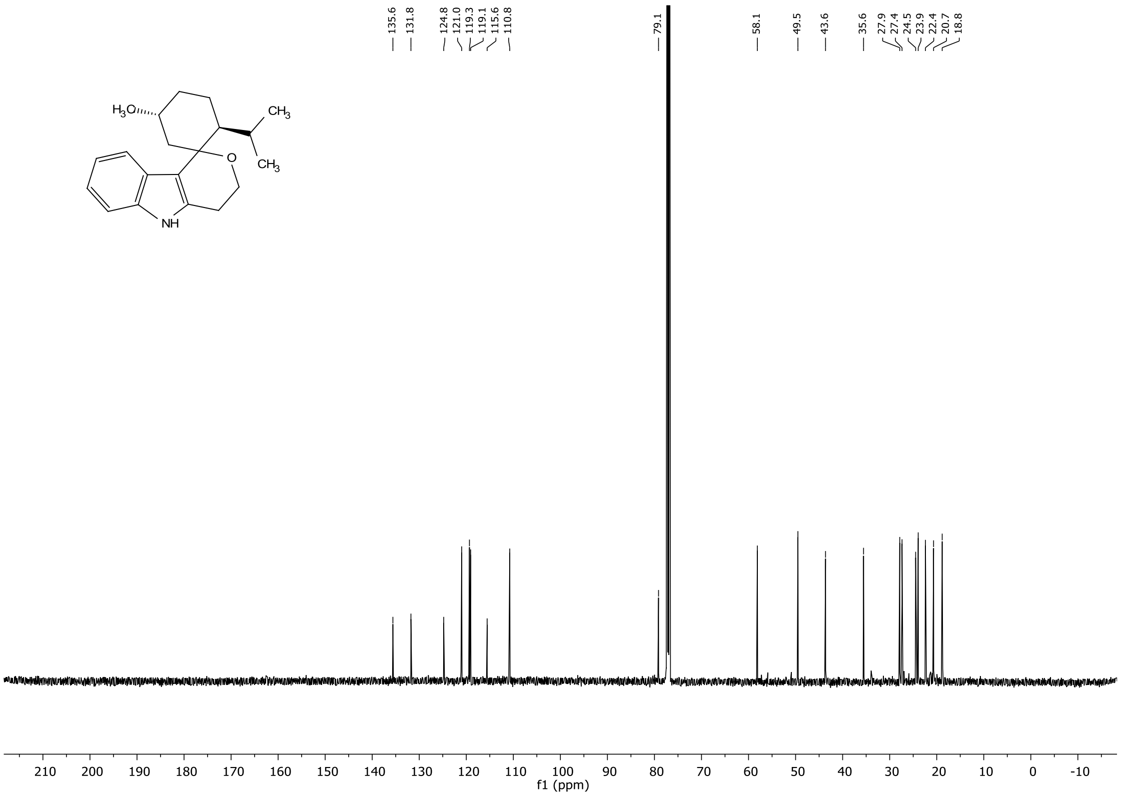

(5*R*,8*S*,9*R*,10*R*,13*S*,14*R*,17*S*)-10,13-dimethyl-17-((*S*)-6-methylheptan-2-yl)-1,2,4,4',5,5',6,7,8,9,10,11,12,13,14,15,16,17-octadecahydro-3'*H*-spiro[cyclopenta[*a*]phenanthrene-3,1'-pyrano[4,3-*b*]indole] **3f**

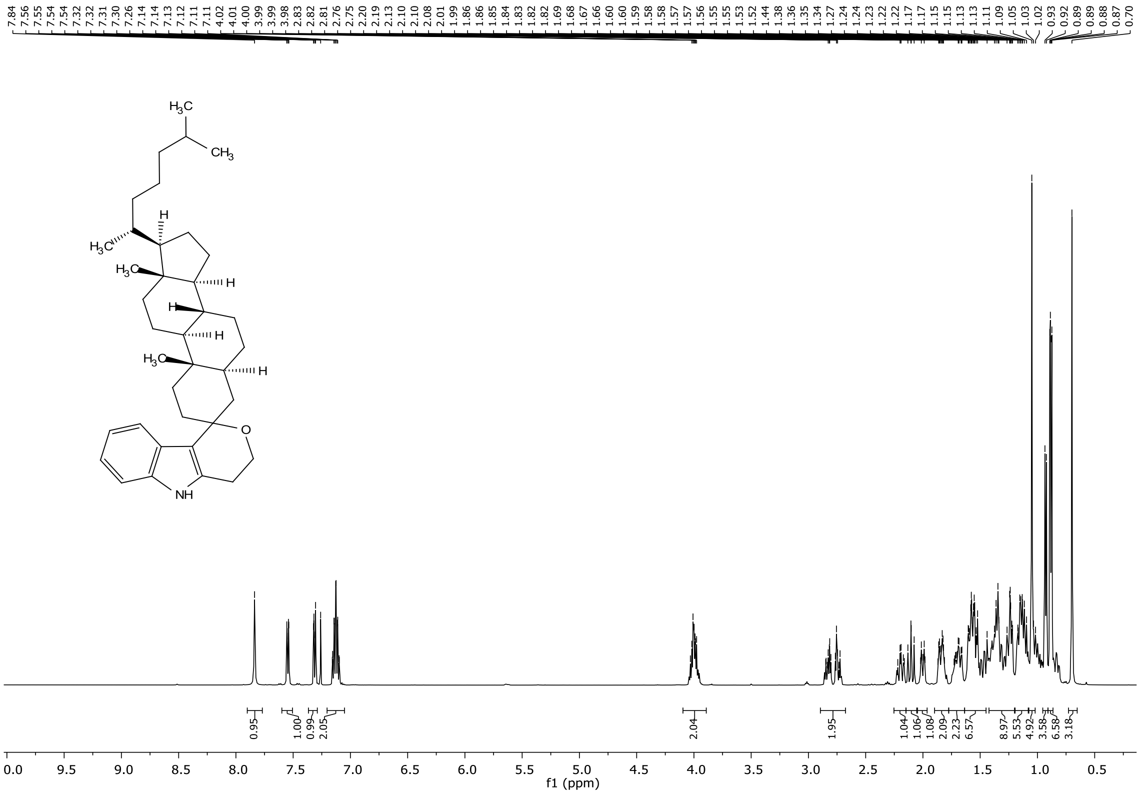

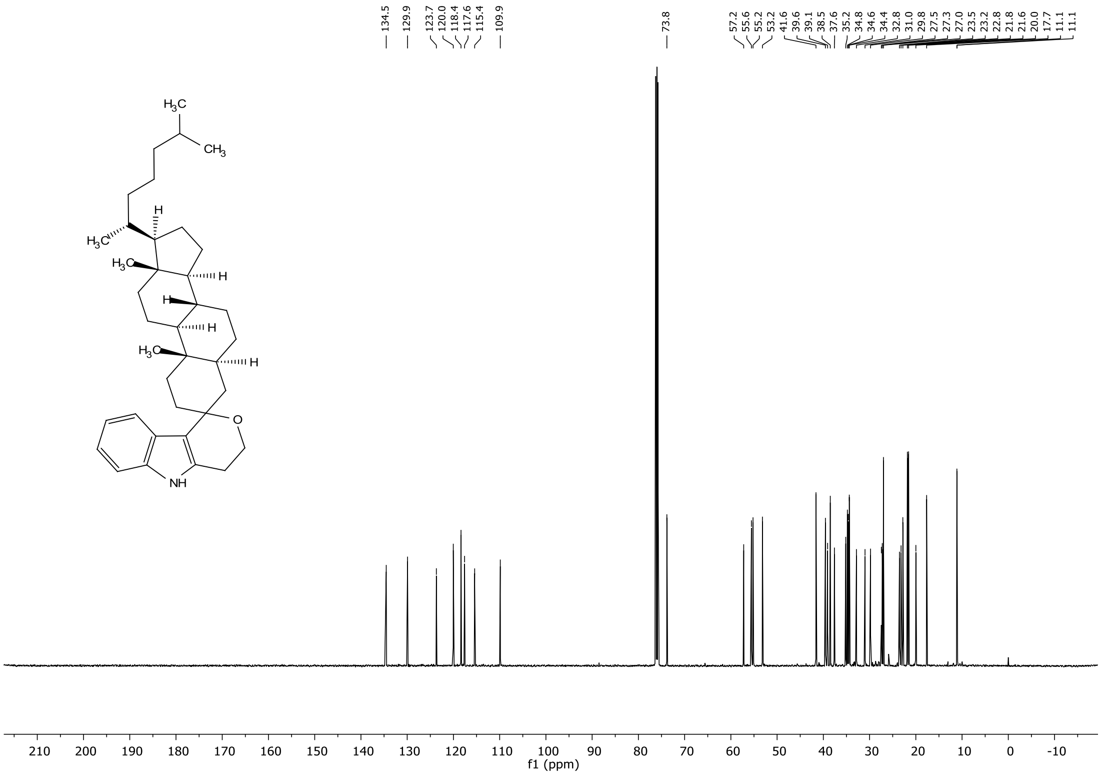

4',5'-dihydro-3'*H*-spiro[indoline-3,1'-pyrano[4,3-*b*]indol]-2-one **3g**

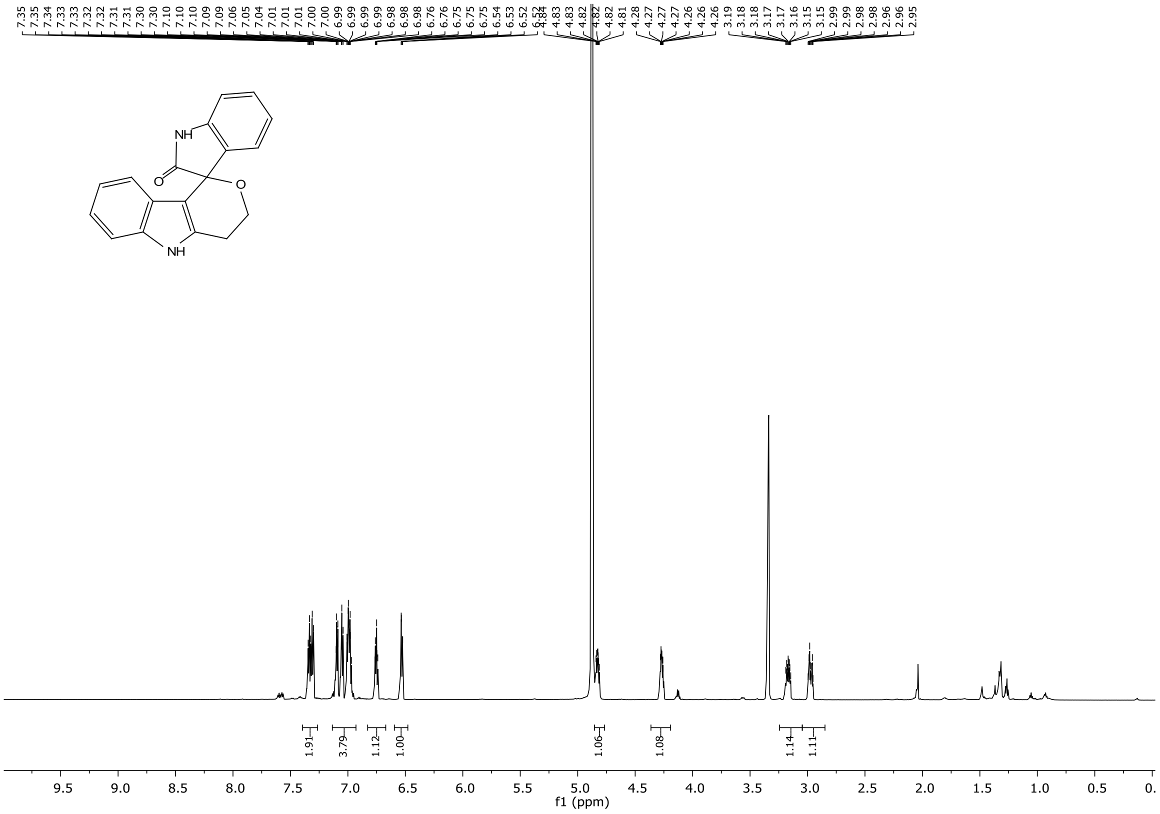

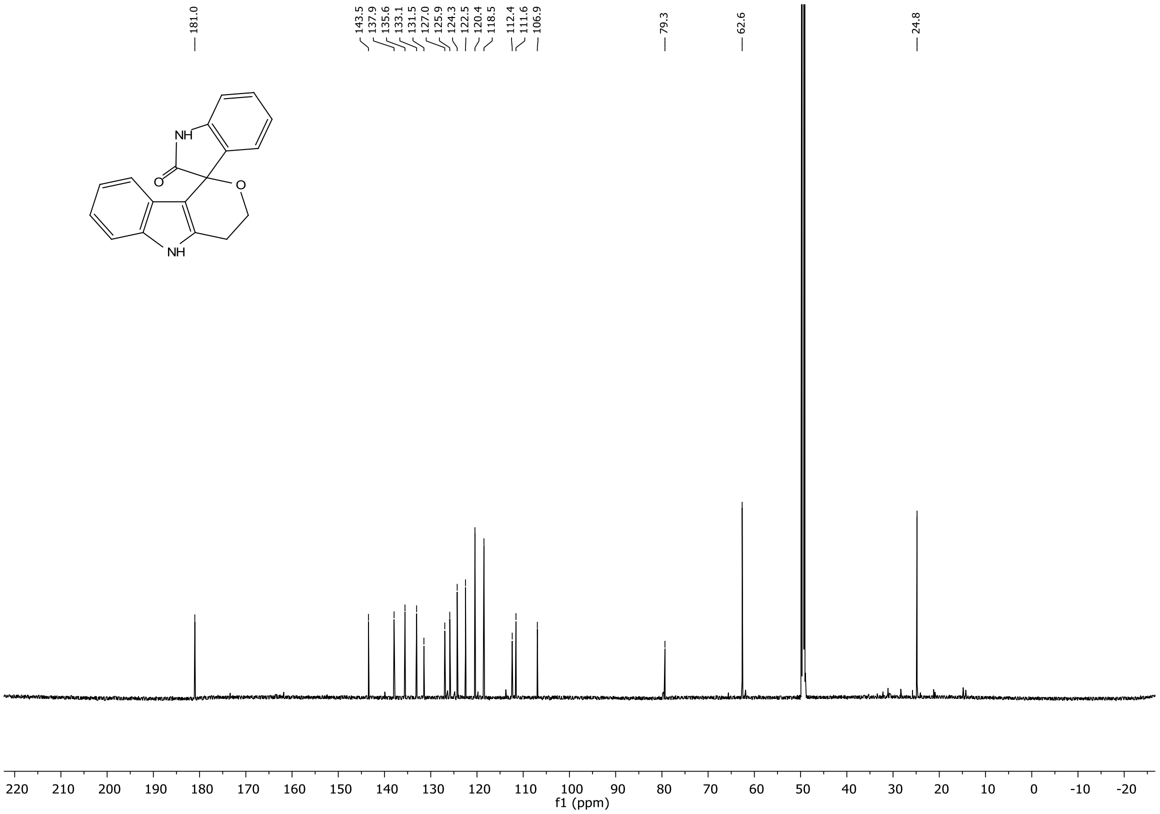

4',5'-dihydro-3'*H*-spiro[chromane-4,1'-pyrano[4,3-*b*]indole] **3h**

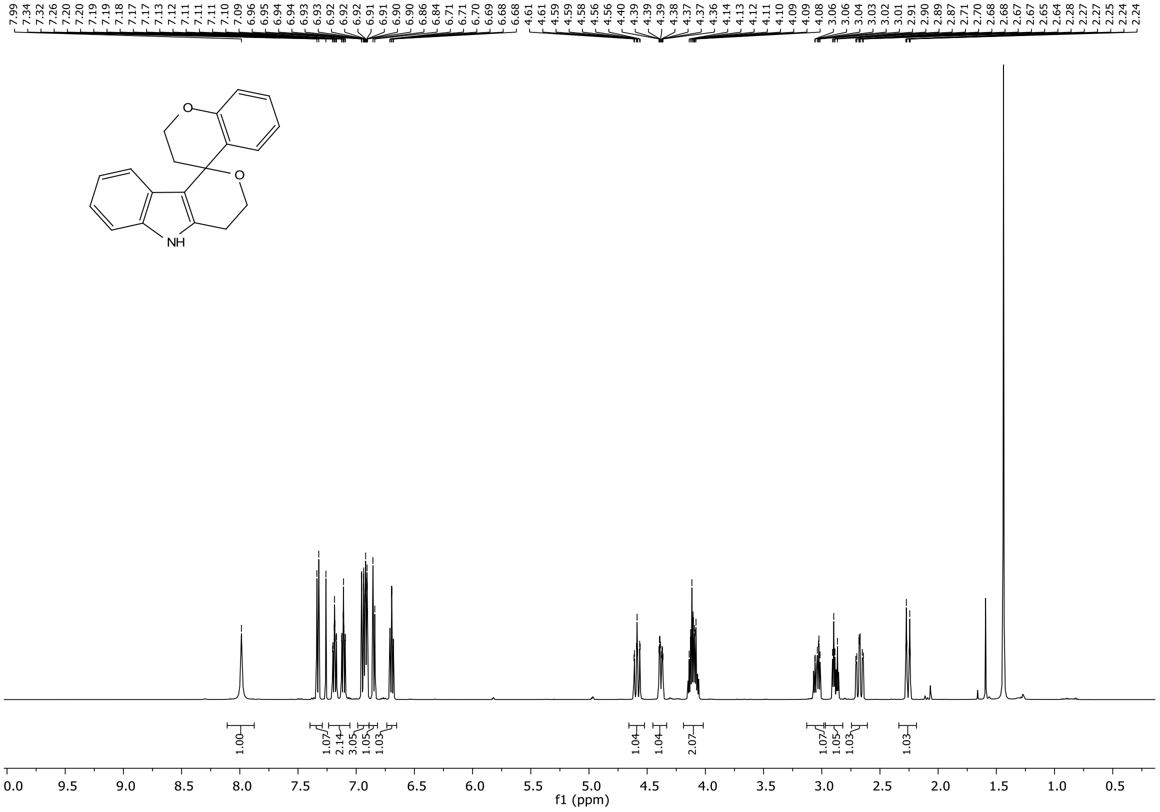

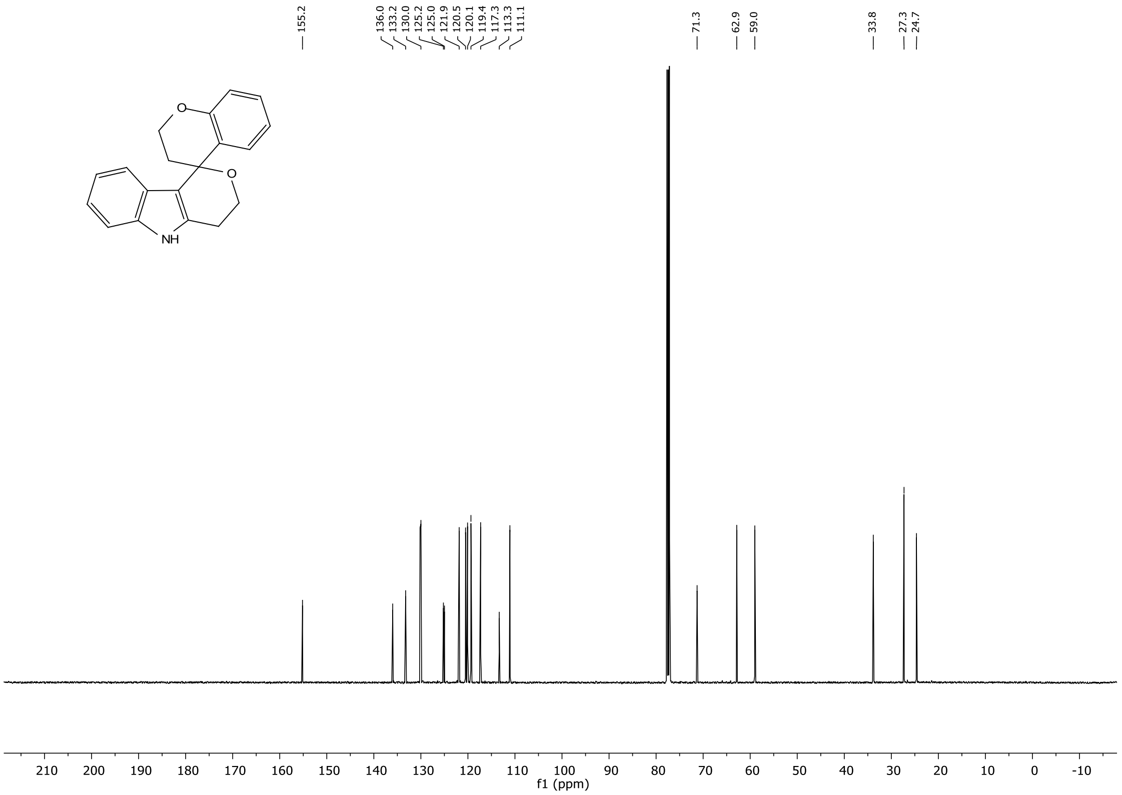

2,3,4',5,5',6-hexahydro-3'*H*-spiro[pyran-4,1'-pyrano[4,3-*b*]indole] **3i**

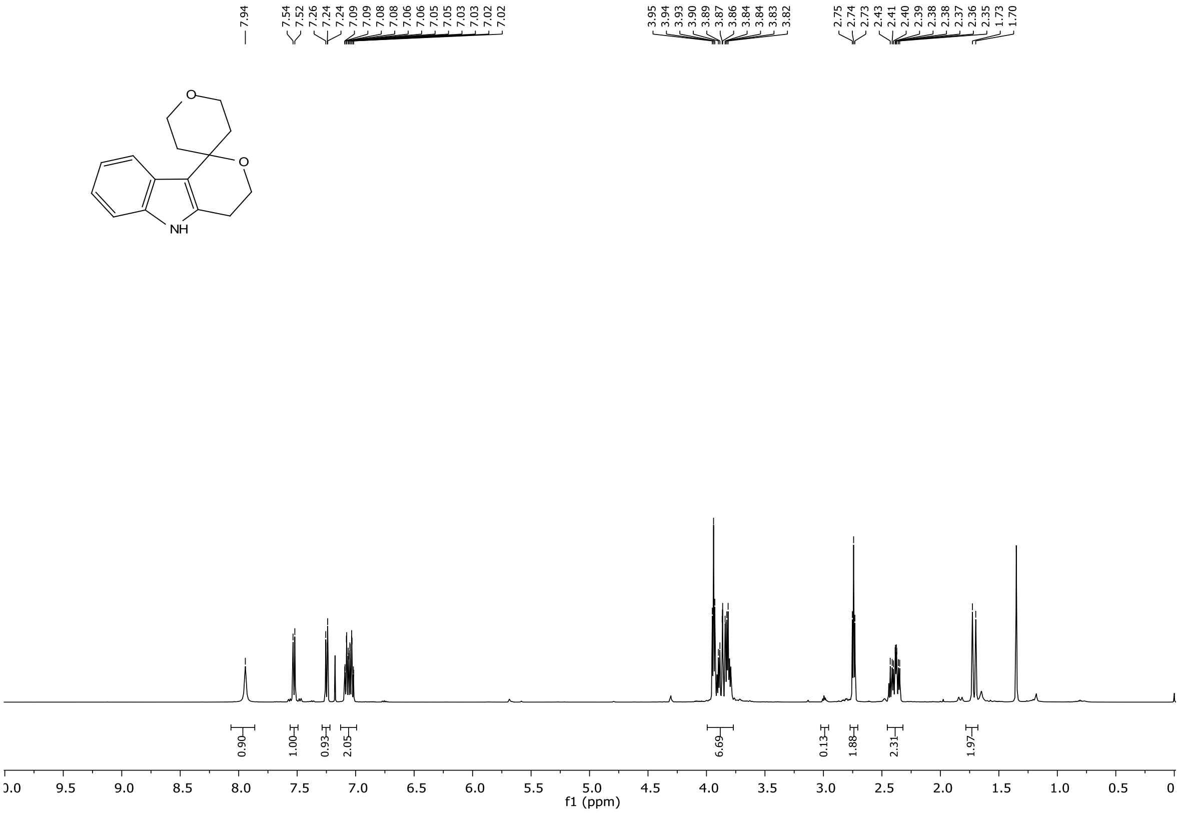

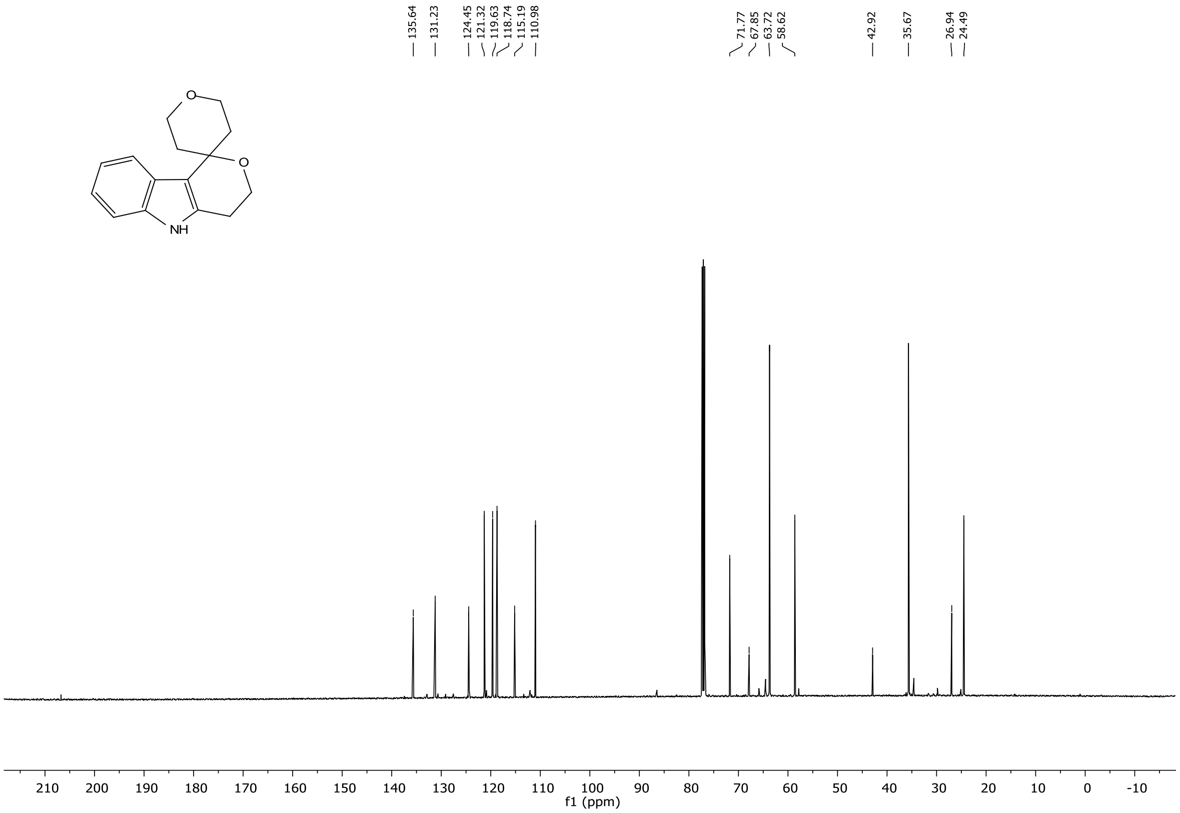

2',3',4,5,5',6'-hexahydro-3H-spiro[pyrano[4,3-b]indole-1,4'-thiopyran] **3j**

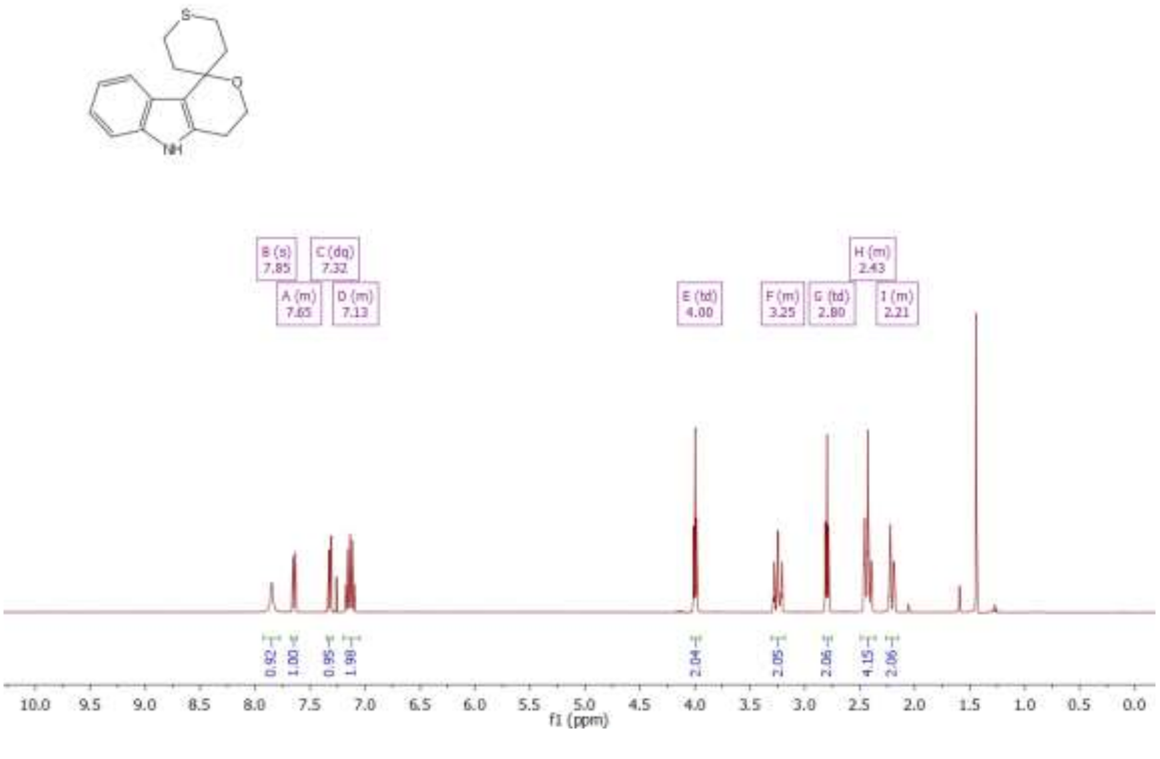

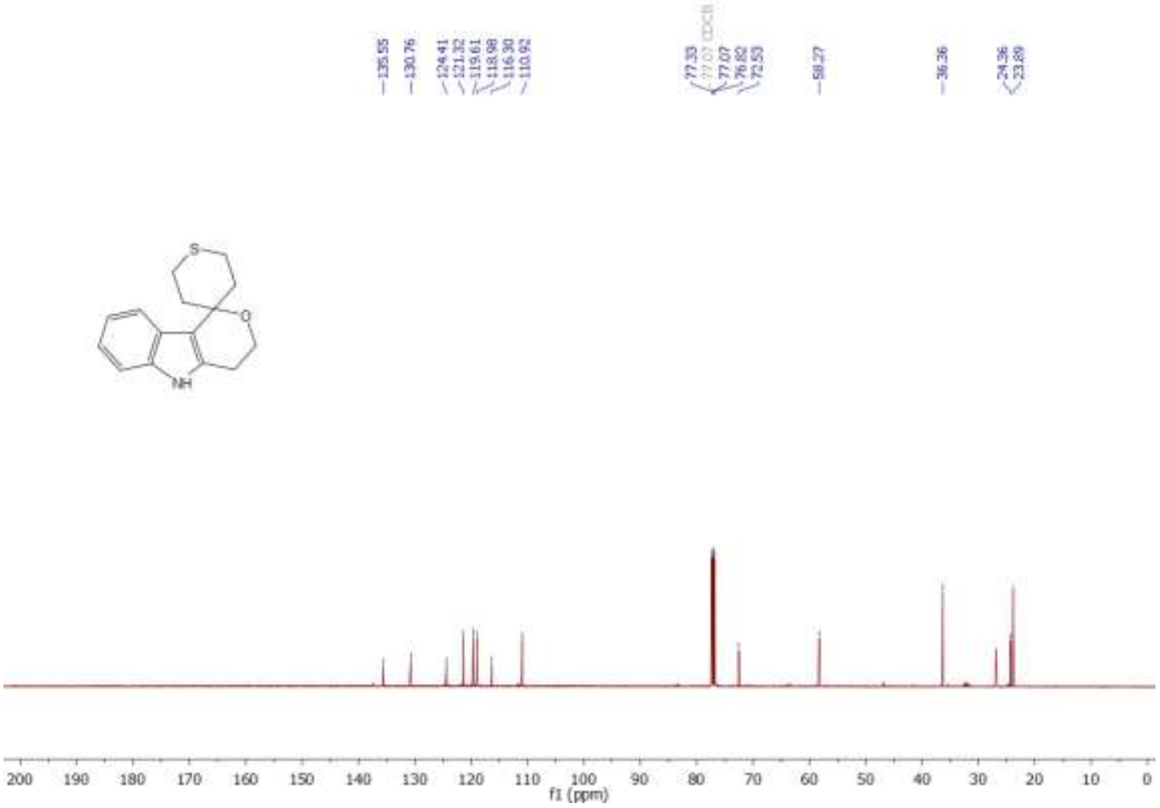

1-(4',5'-dihydro-3'H-spiro[piperidine-4,1'-pyrano[4,3-b]indol]-1-yl)ethan-1-one **3l**

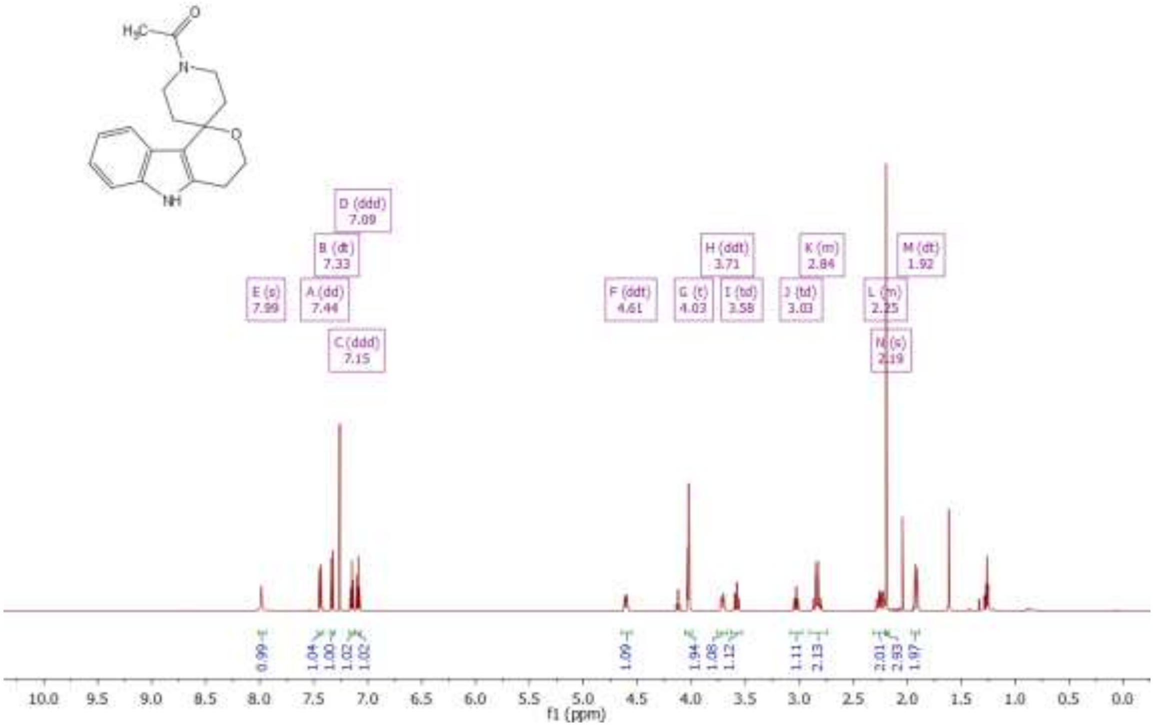

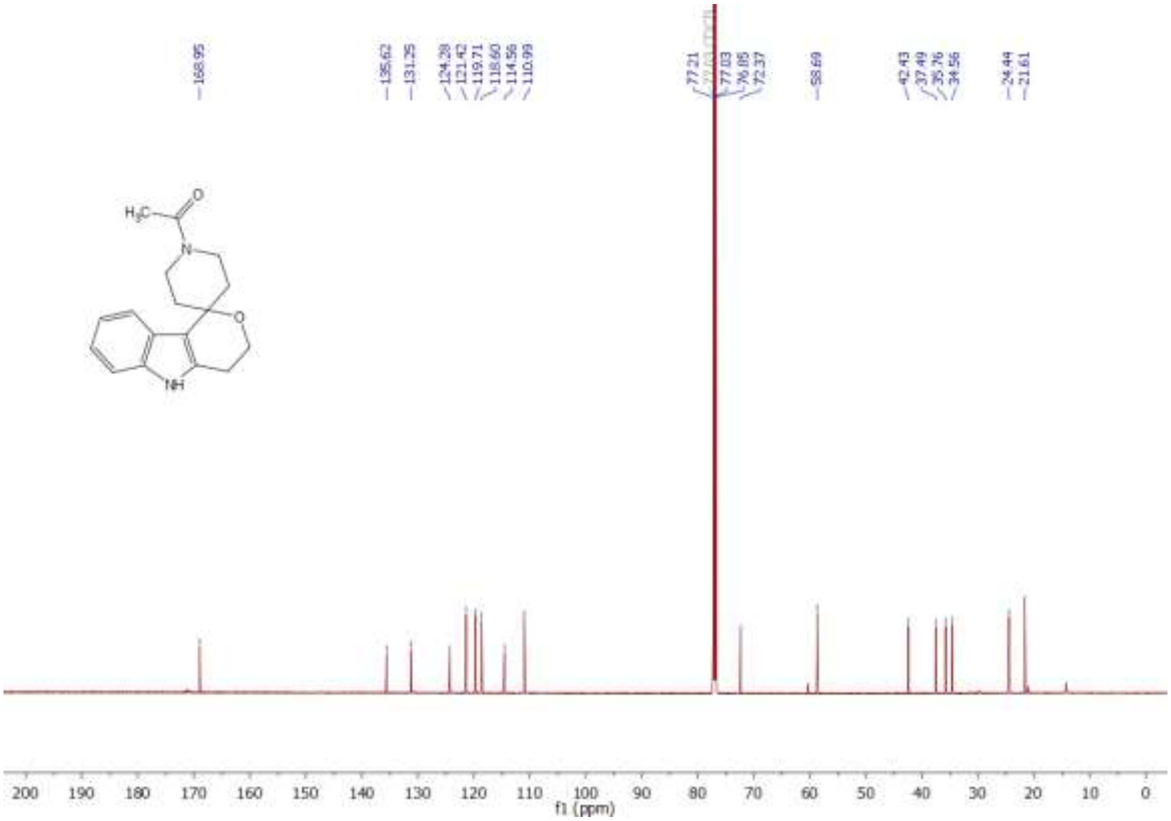

(4',5'-dihydro-3'H-spiro[piperidine-4,1'-pyrano[4,3-b]indol]-1-yl)(phenyl)methanone **3m**

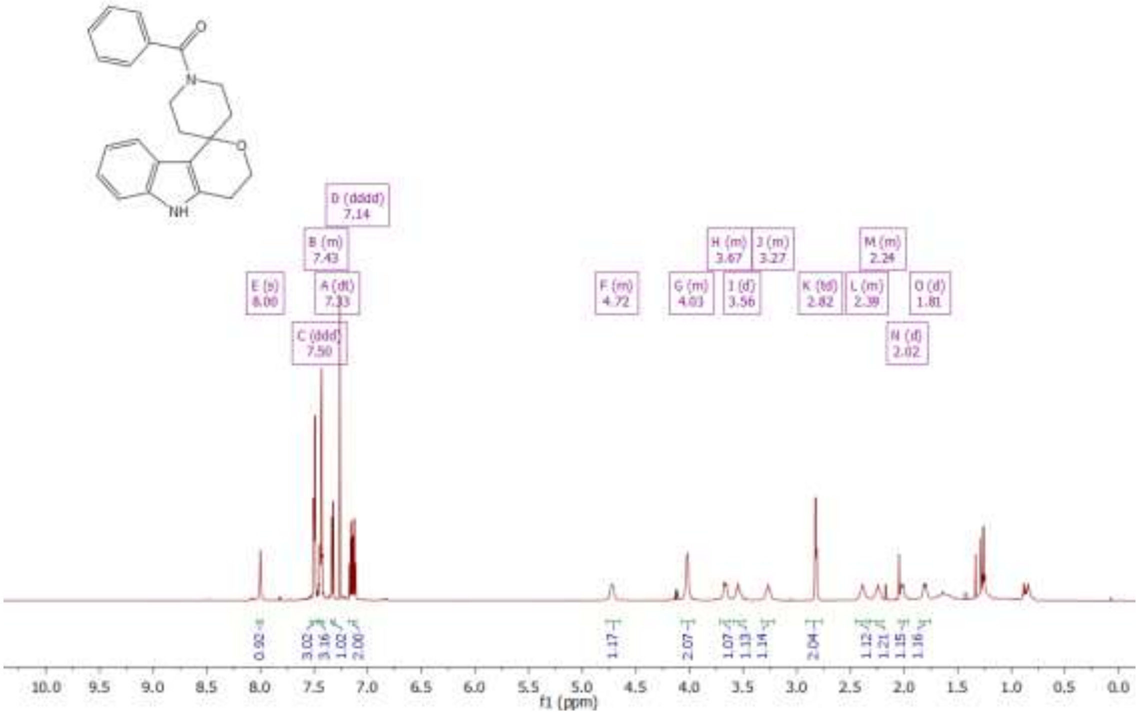

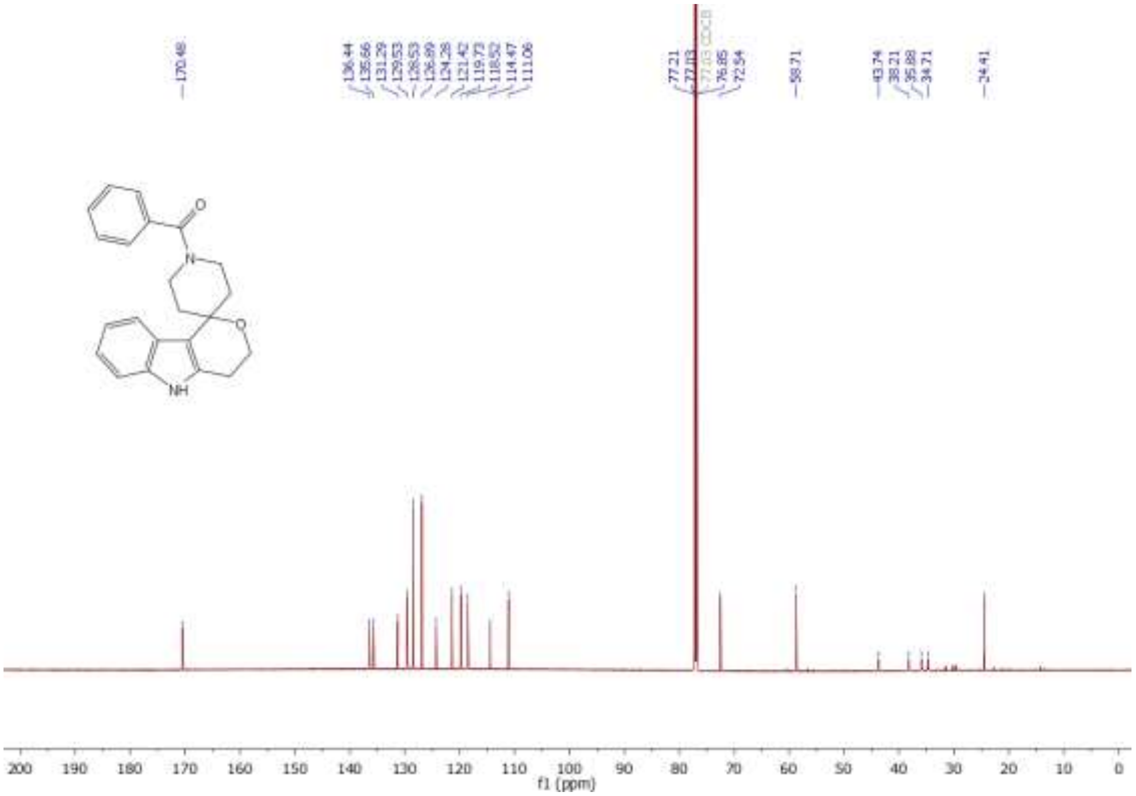

tert-butyl 4',5'-dihydro-3'H-spiro[piperidine-4,1'-pyrano[4,3-b]indole]-1-carboxylate **3n**

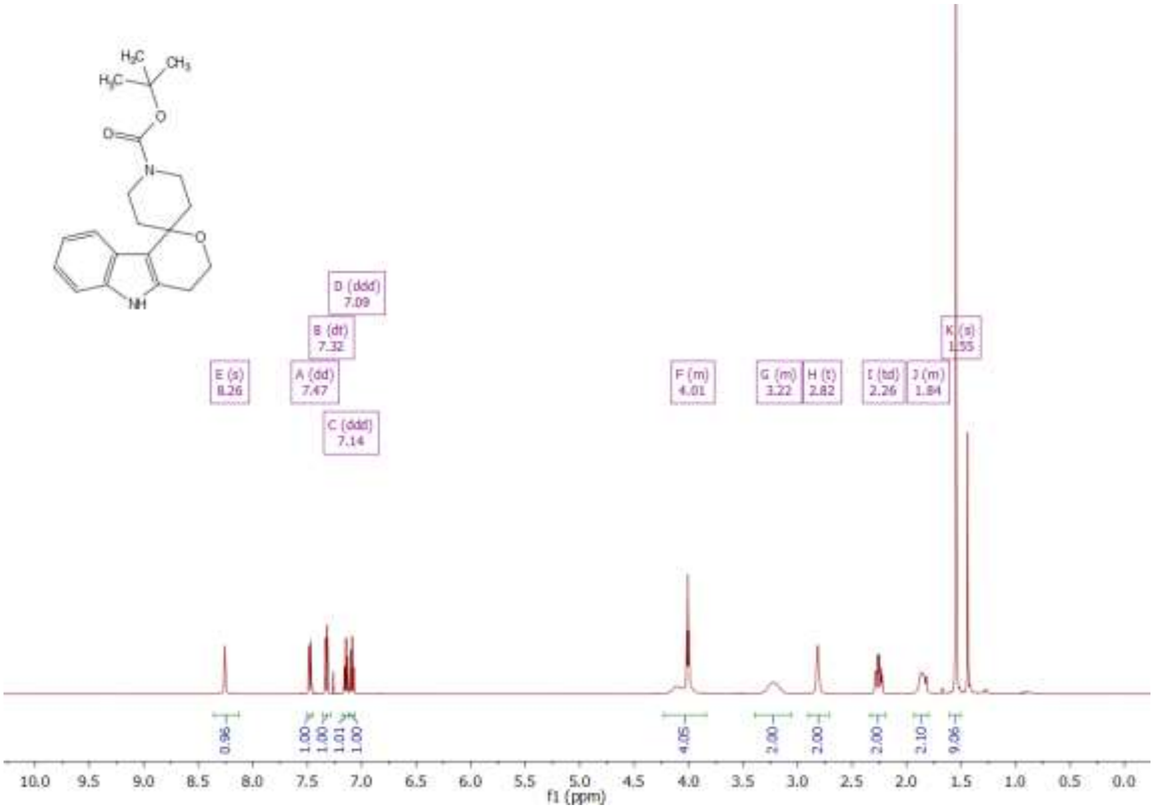

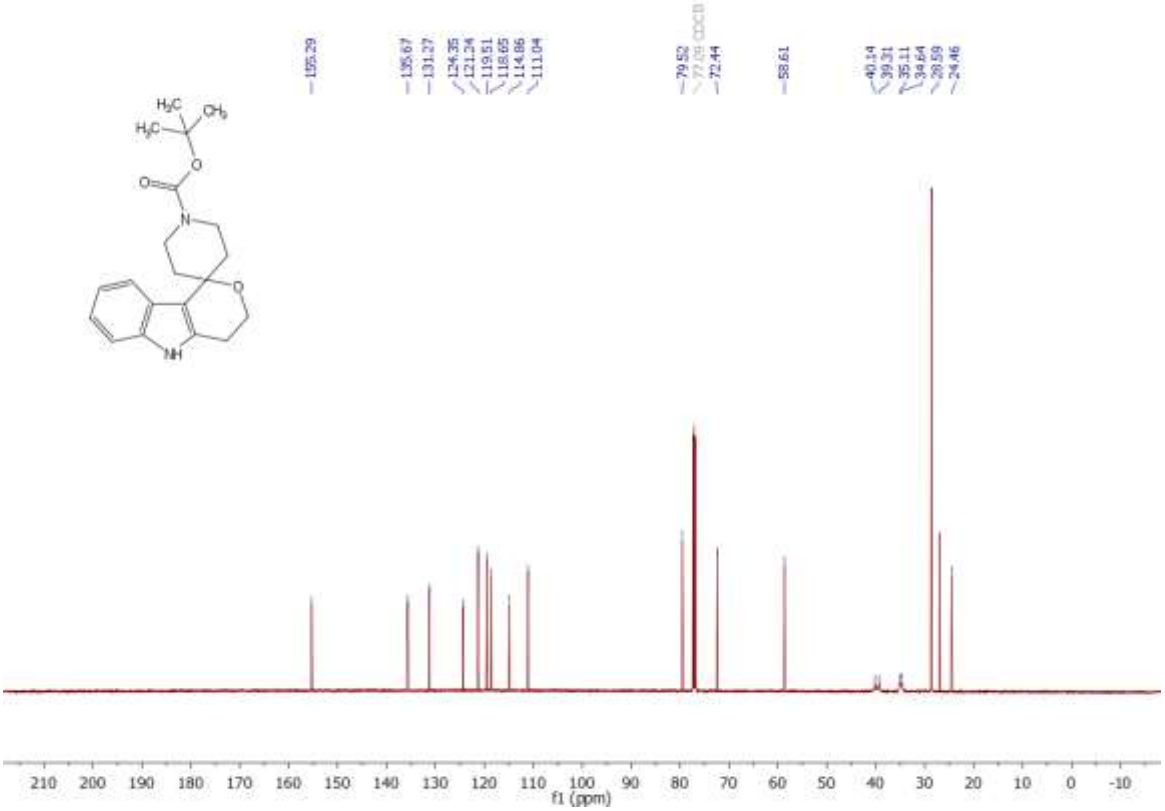

tert-butyl 4',5'-dihydro-3'H-8-azaspiro[bicyclo[3.2.1]octane-3,1'-pyrano[4,3-b]indole]-8-carboxylate **3p**

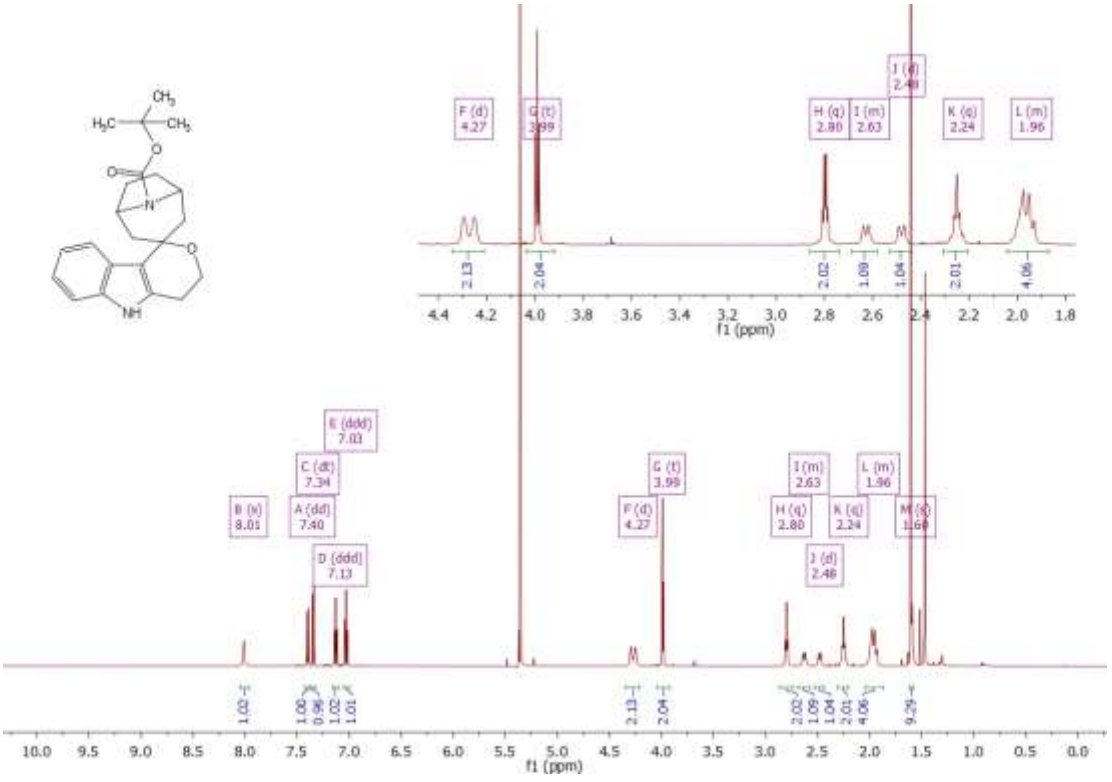

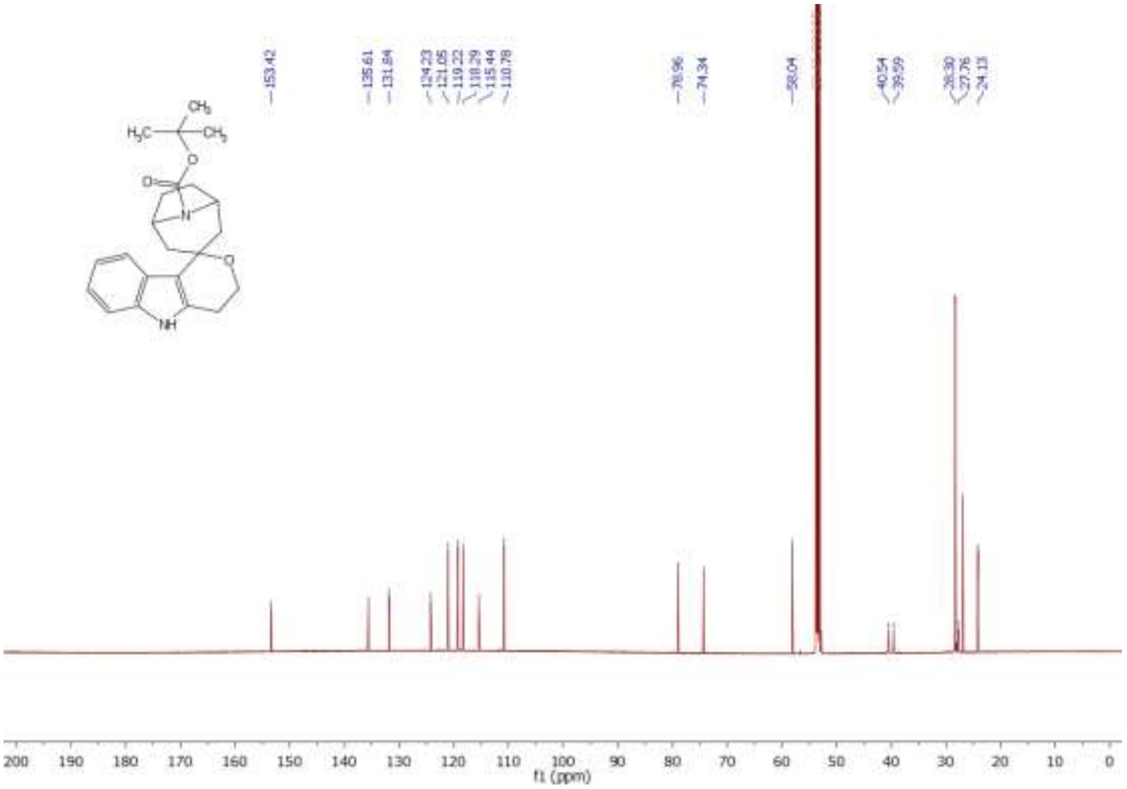

4-(2-(1-methyl-1,3,4,5-tetrahydropyrano[4,3-b]indol-1-yl)ethyl)phenol **3q**

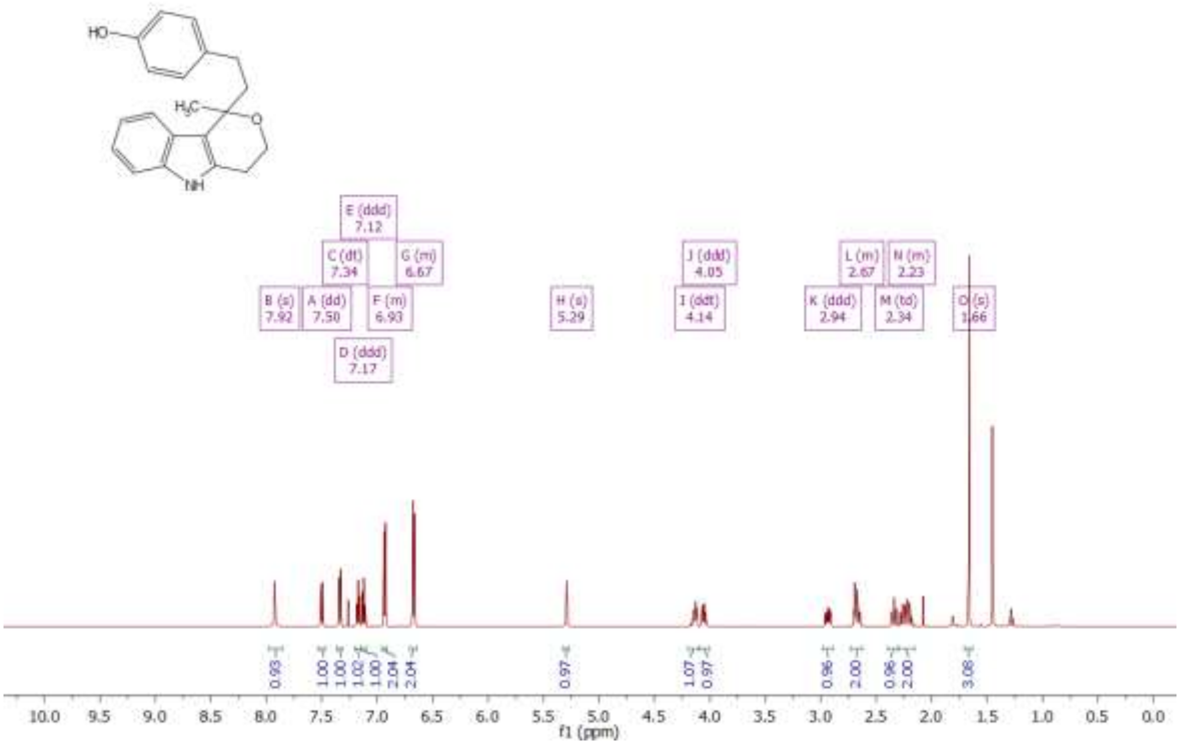

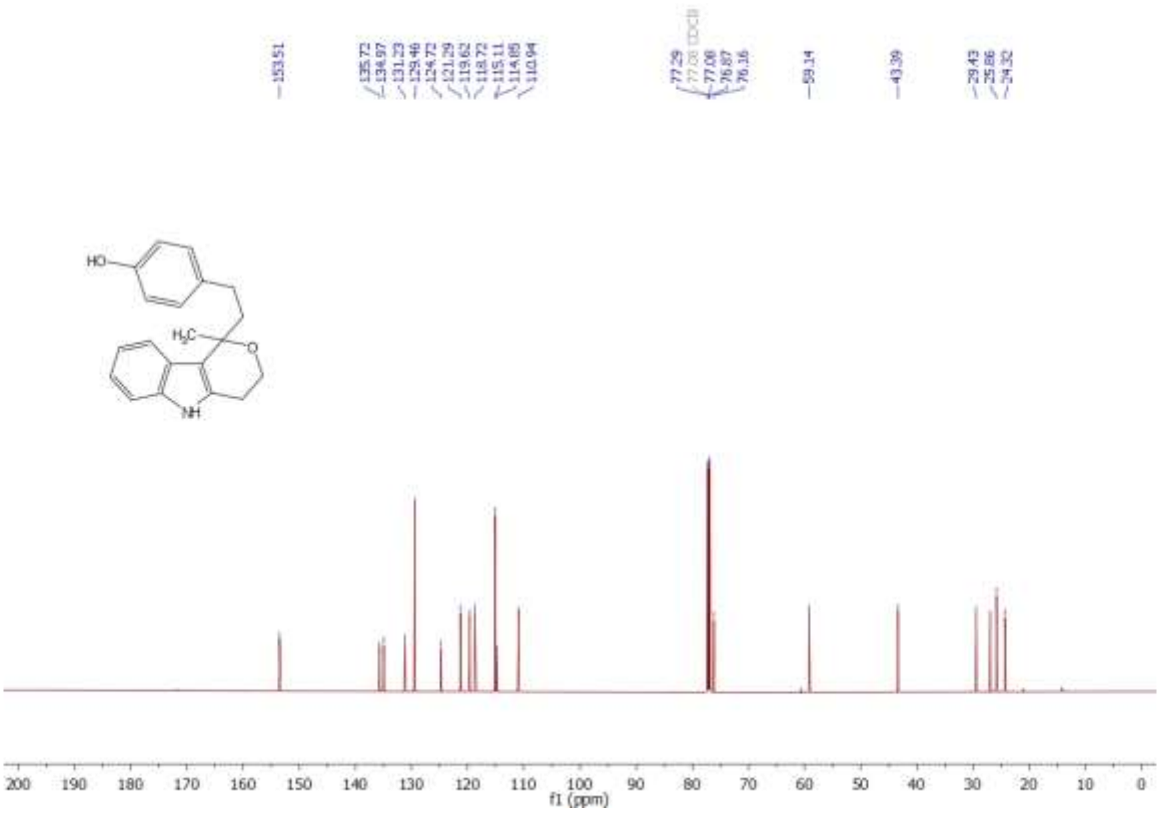

methyl 1-methyl-1,3,4,5-tetrahydropyrano[4,3-b]indole-1-carboxylate **3r**

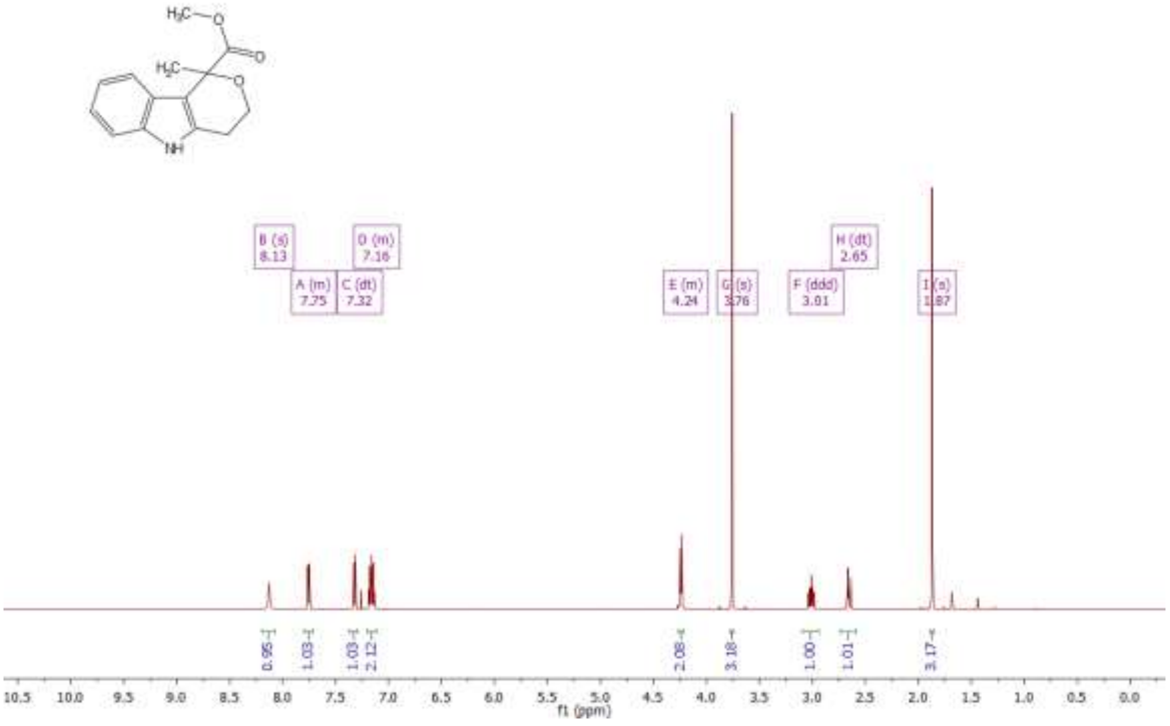

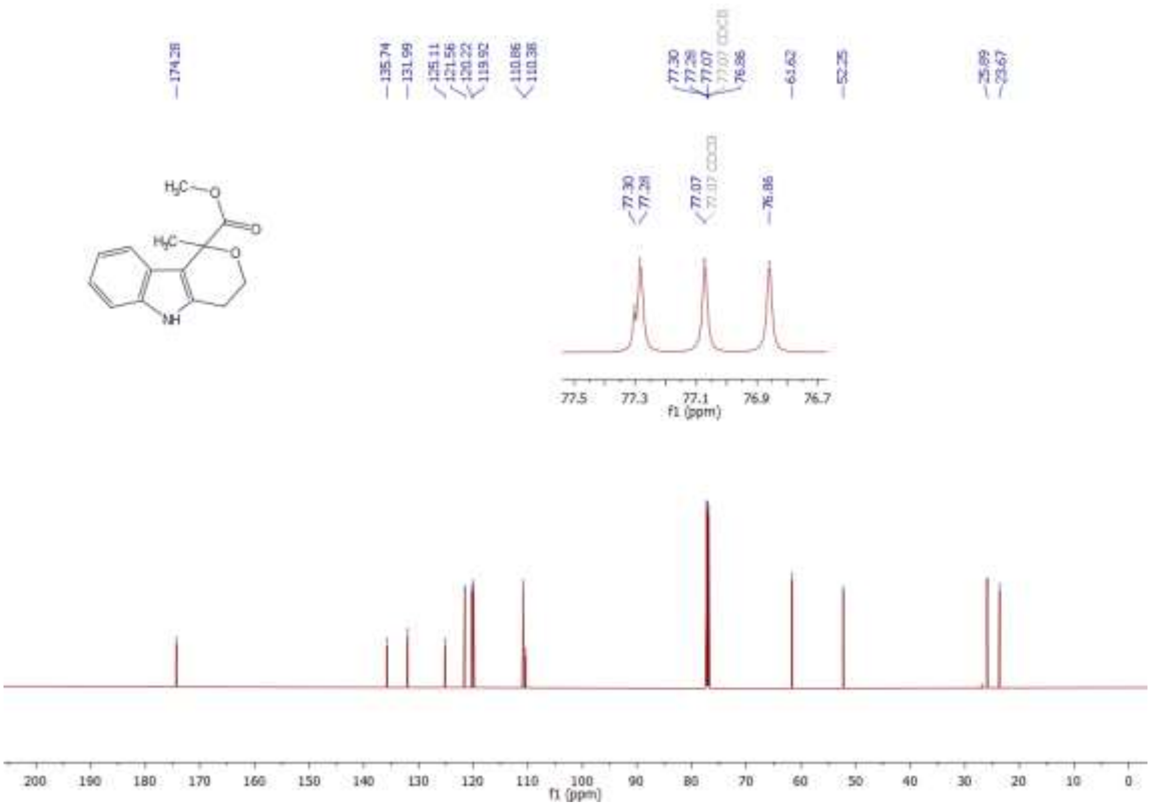

1-phenyl-1,3,4,5-tetrahydropyrano[4,3-b]indole **3s**

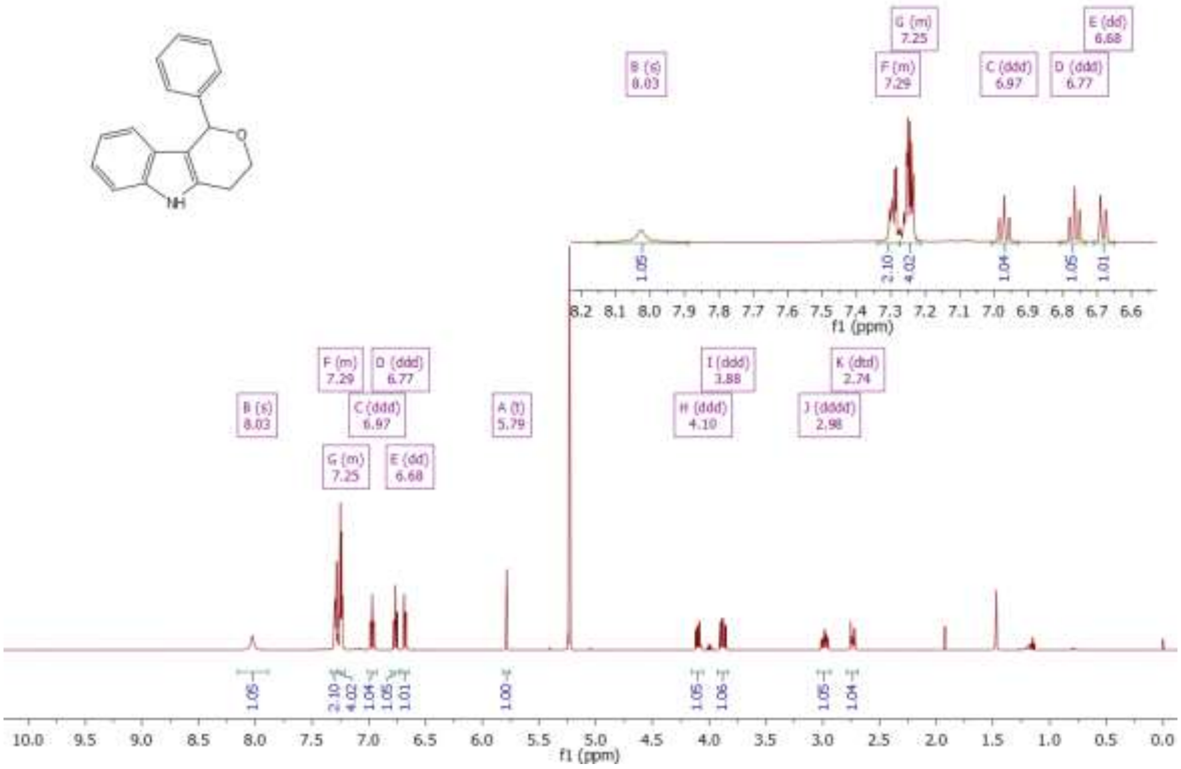

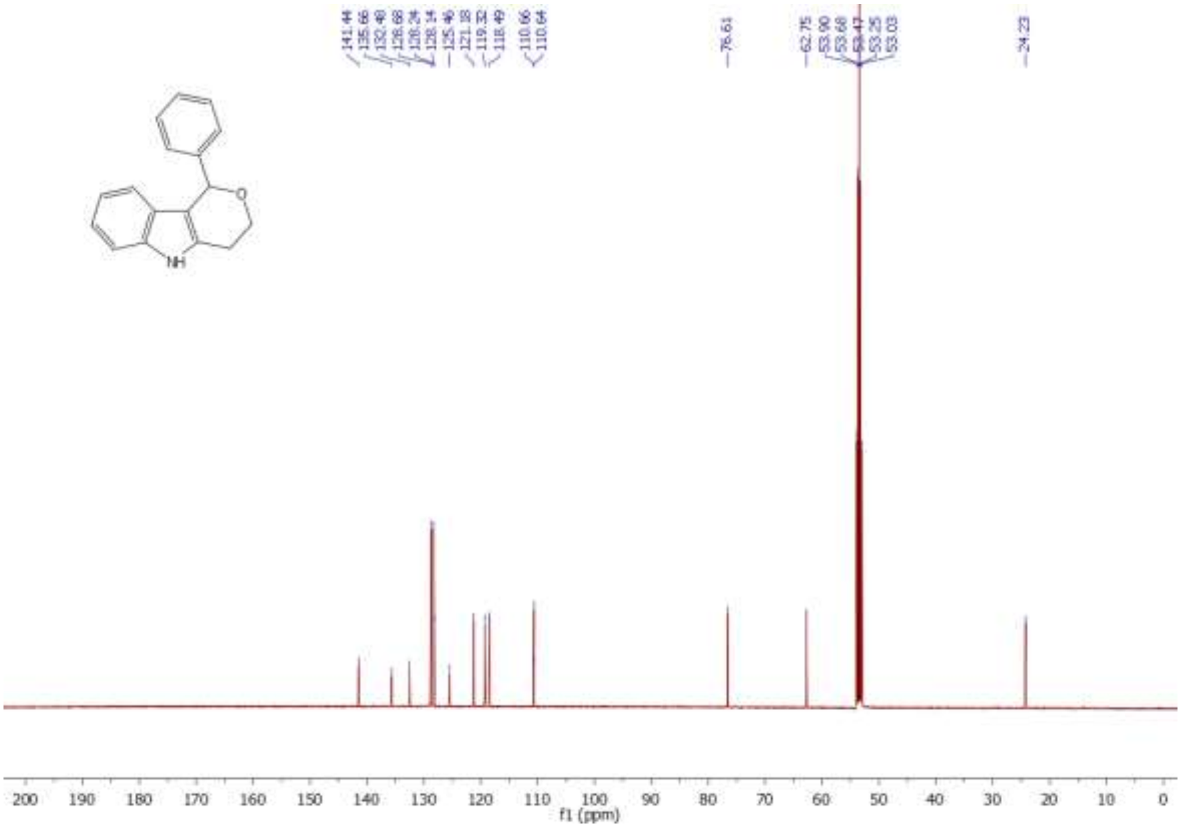

1-(2-bromophenyl)-1,3,4,5-tetrahydropyrano[4,3-b]indole **3t**

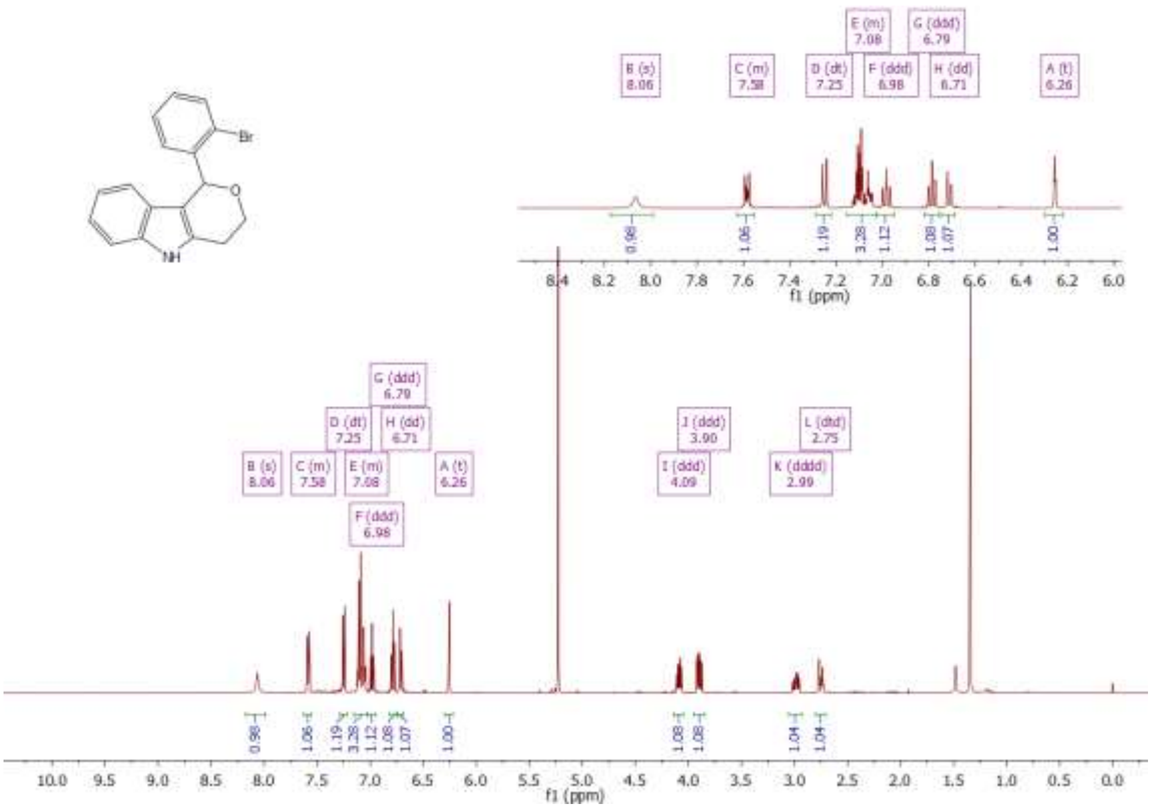

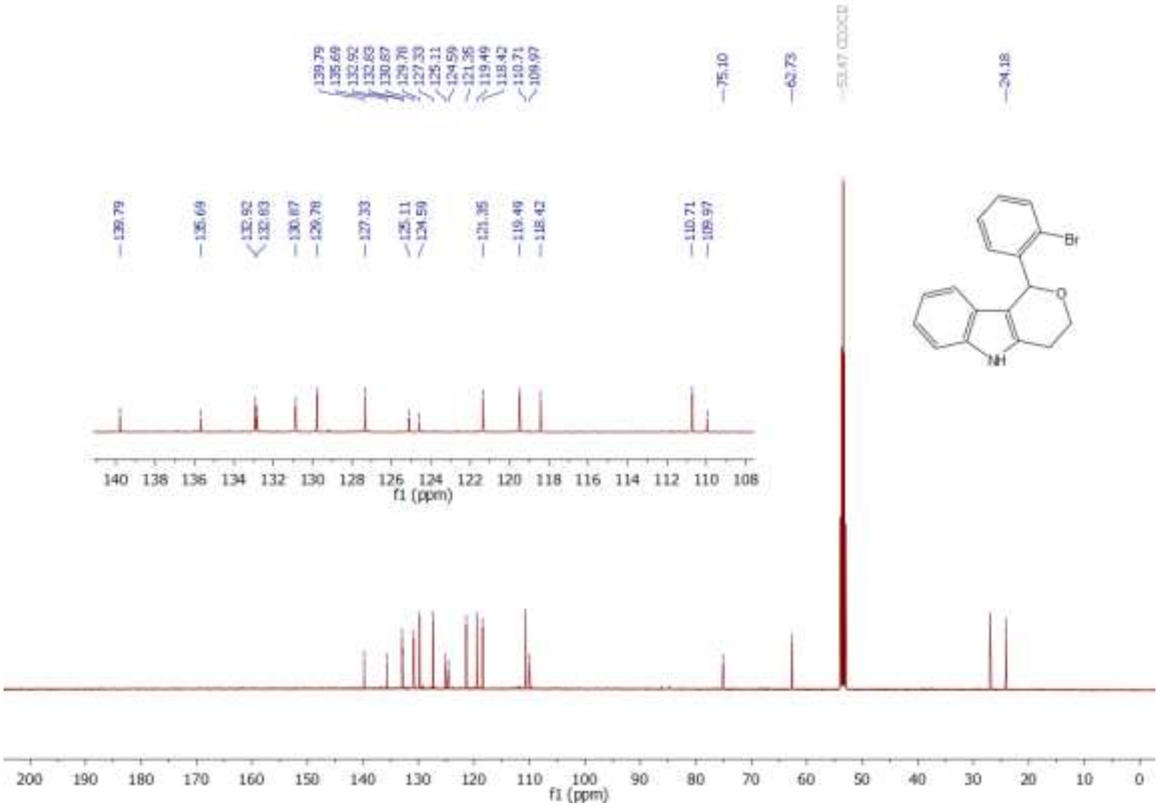

(2*R*,2'*R*,4'*S*)-7-chloro-4,6-dimethoxy-2'-methyl-4'',5''-dihydro-3*H*,3''*H*-dispiro[benzofuran-2,1'-cyclohexane-4',1''-pyrano[4,3-*b*]indol]-3-one **7a**

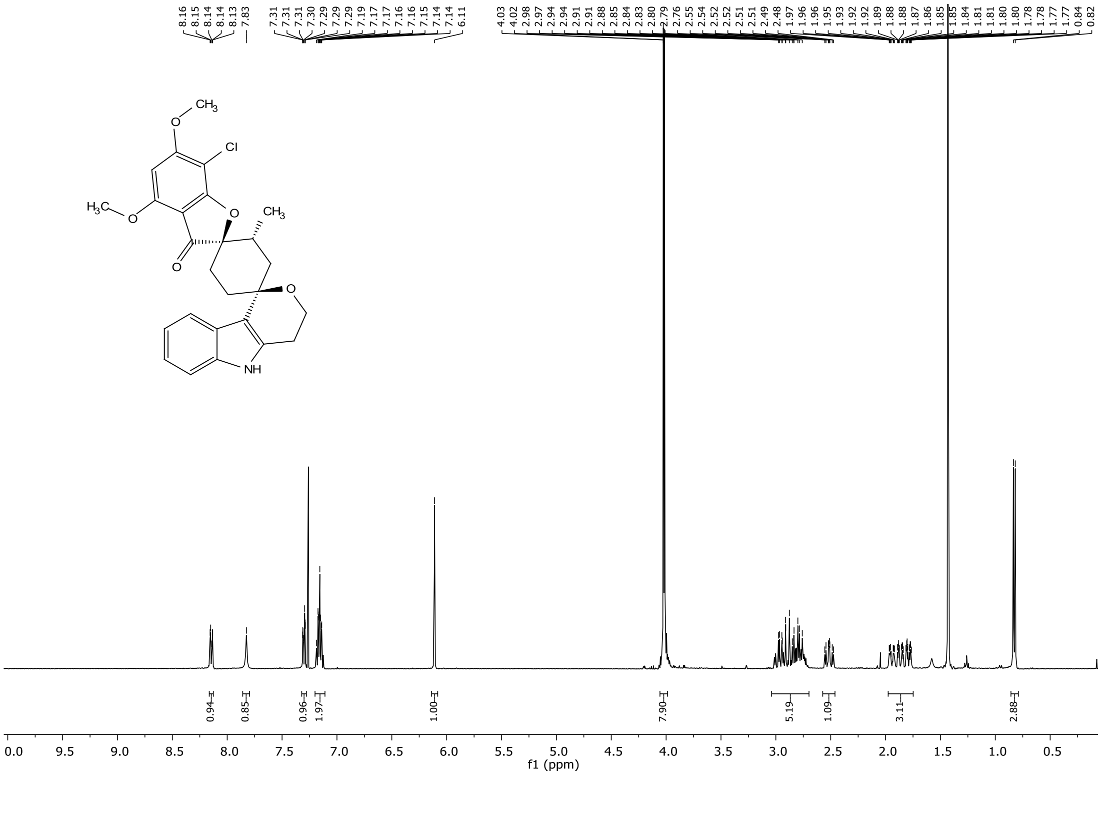

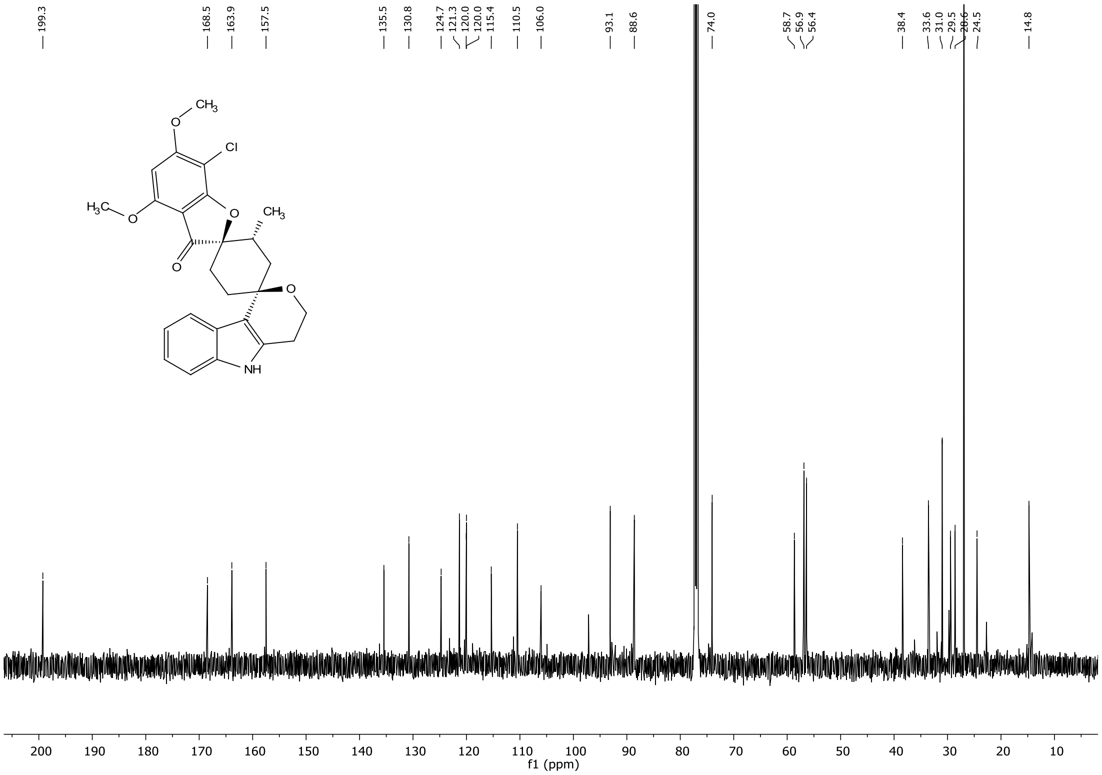

(2*R*,2'*R*,4'*S*)-7-chloro-4,6-dimethoxy-2',9''-dimethyl-4'',5''-dihydro-3*H*,3''*H*-dispiro[benzofuran-2,1'-cyclohexane-4',1''-pyrano[4,3-*b*]indol]-3-one **7b**

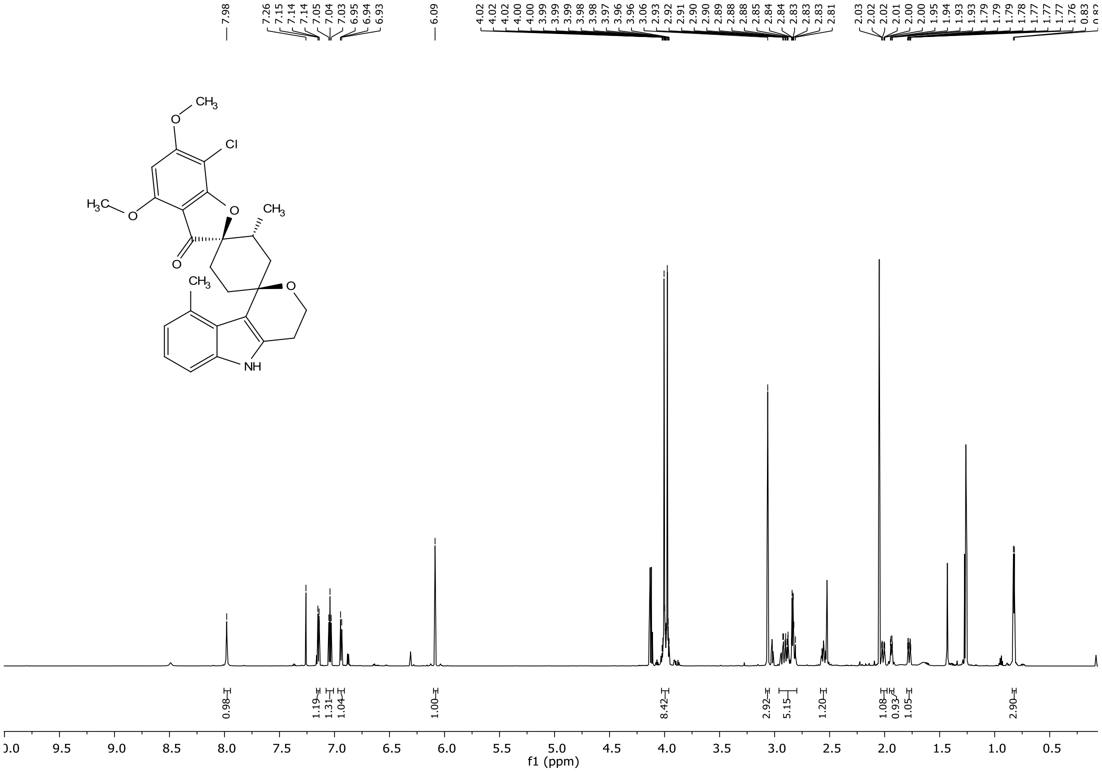

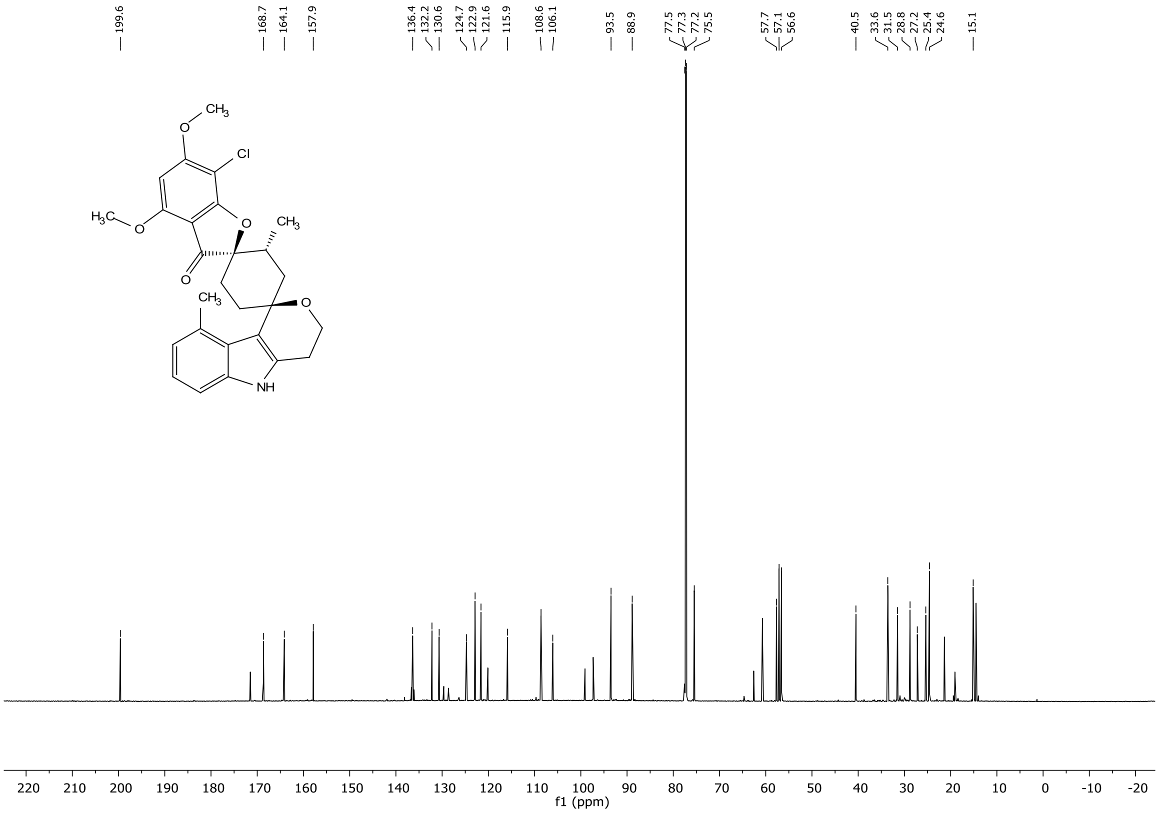

(2*R*,2'*R*,4'*S*)-7-chloro-4,6-dimethoxy-2',8''-dimethyl-4'',5''-dihydro-3*H*,3''*H*-dispiro[benzofuran-2,1'-cyclohexane-4',1''-pyrano[4,3-*b*]indol]-3-one **7c**

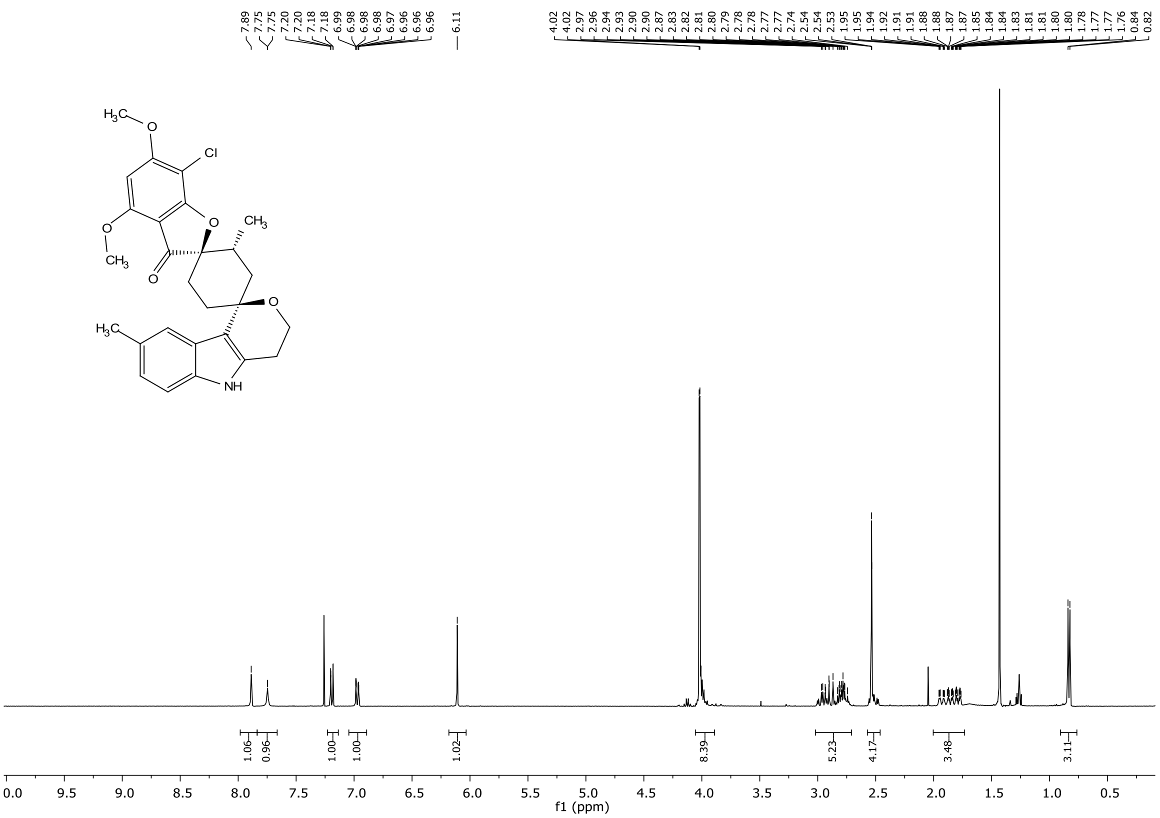

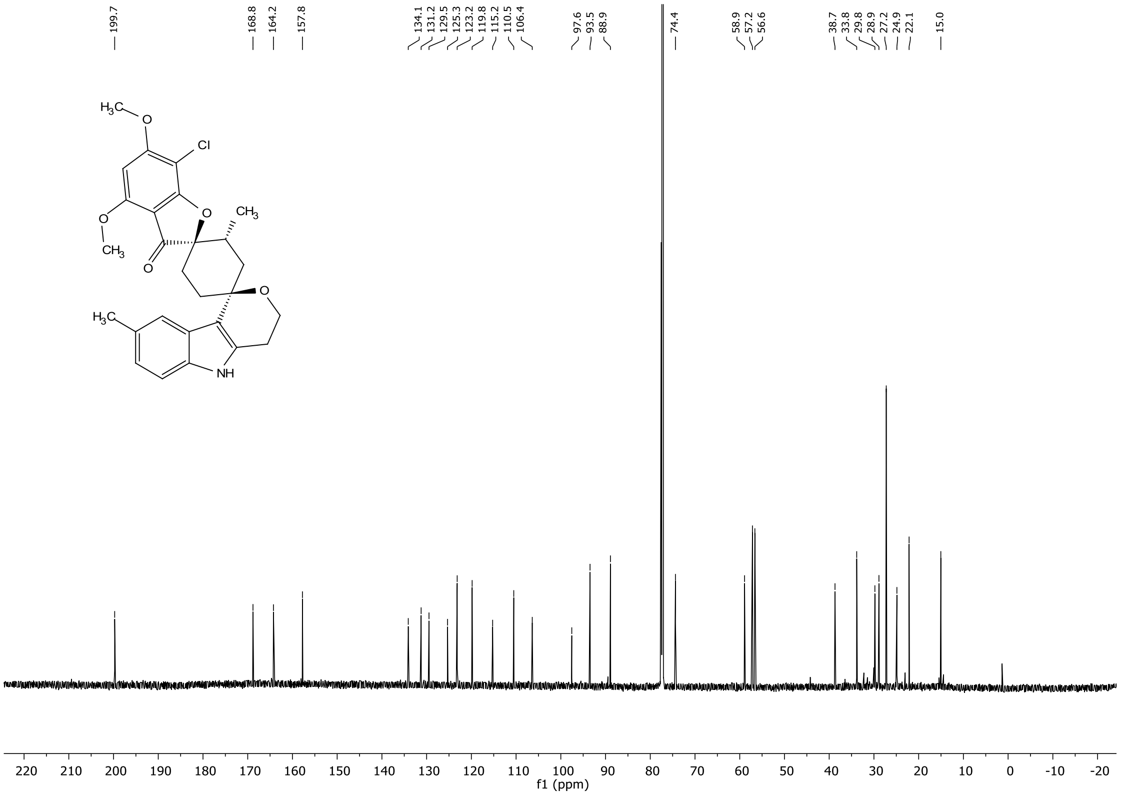

(2*R*,2'*R*,4'*S*)-7-chloro-4,6-dimethoxy-2',7''-dimethyl-4'',5''-dihydro-3*H*,3''*H*-dispiro[benzofuran-2,1'-cyclohexane-4',1''-pyrano[4,3-*b*]indol]-3-one **7d**

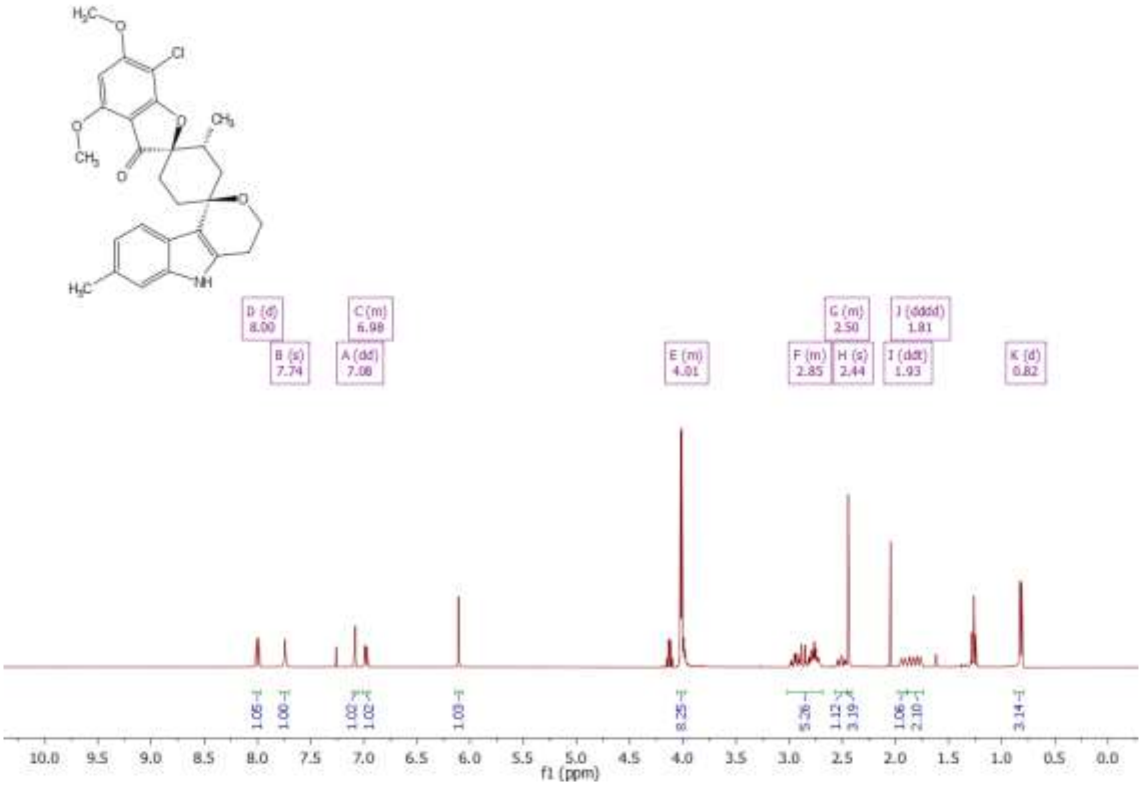

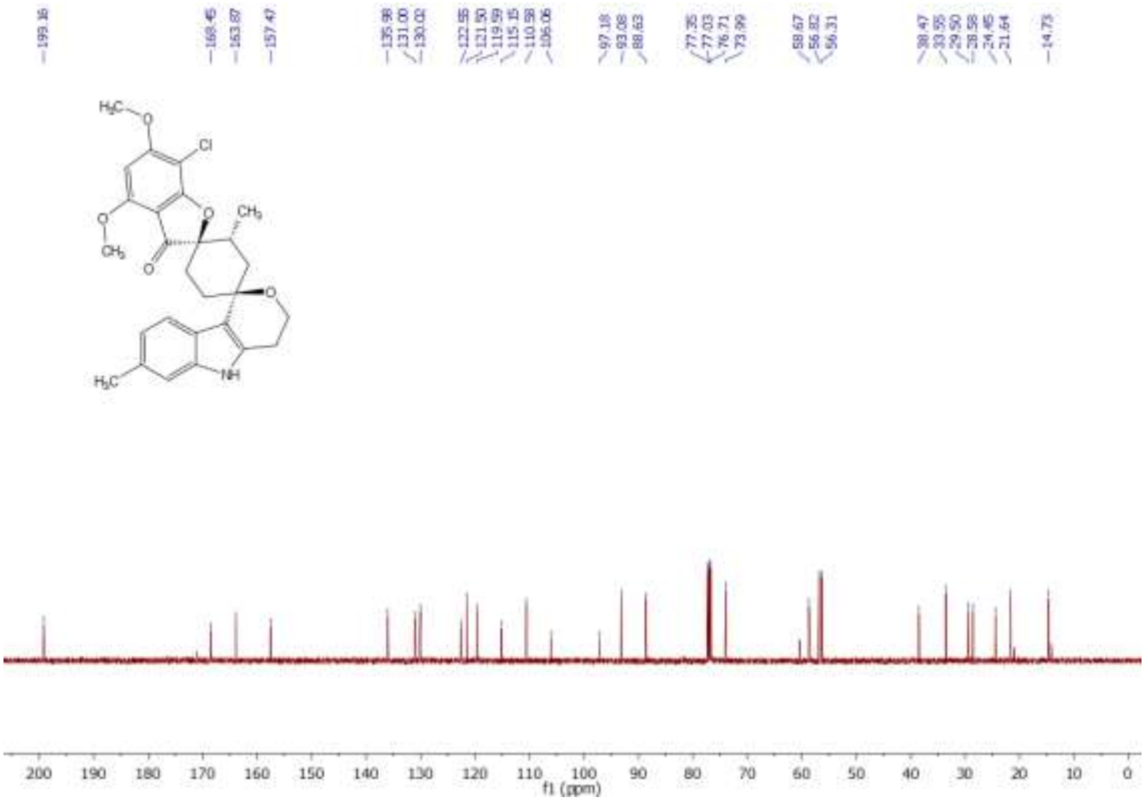

(2*R*,2'*R*,4'*S*)-7-chloro-4,6-dimethoxy-2',6''-dimethyl-4'',5''-dihydro-3*H*,3''*H*-dispiro[benzofuran-2,1'-cyclohexane-4',1''-pyrano[4,3-*b*]indol]-3-one **7e**

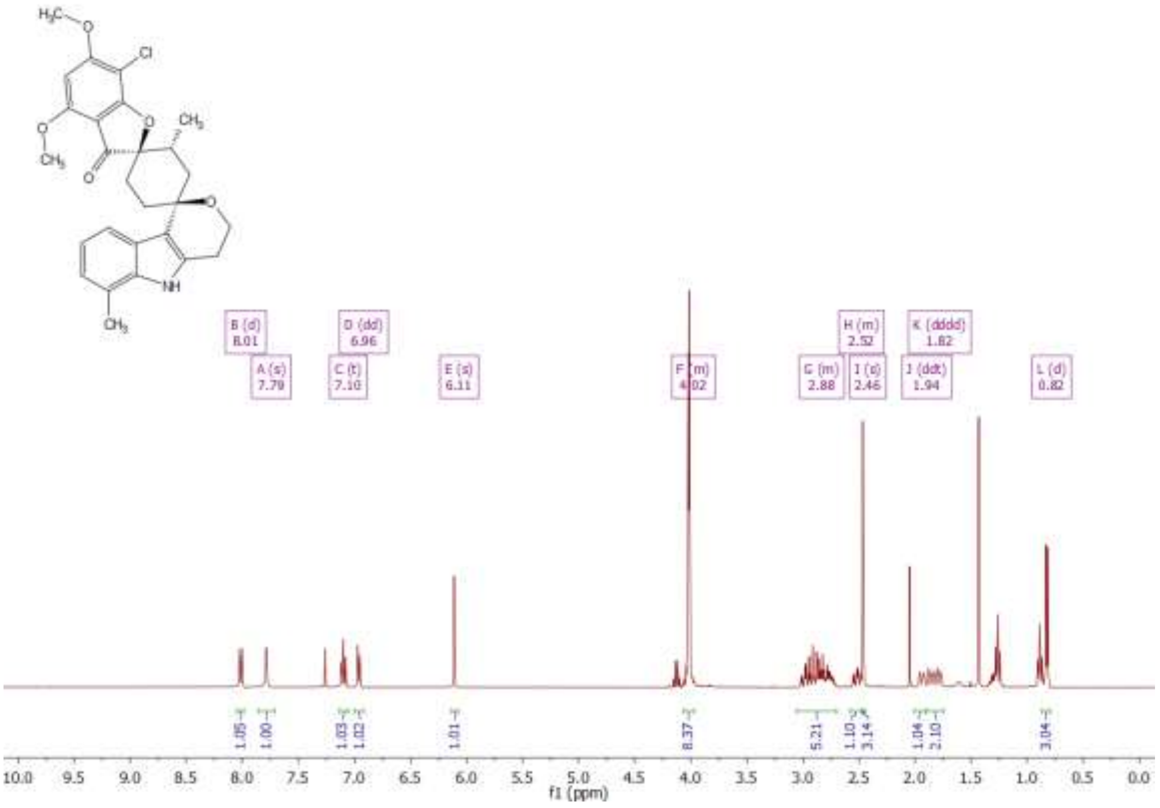

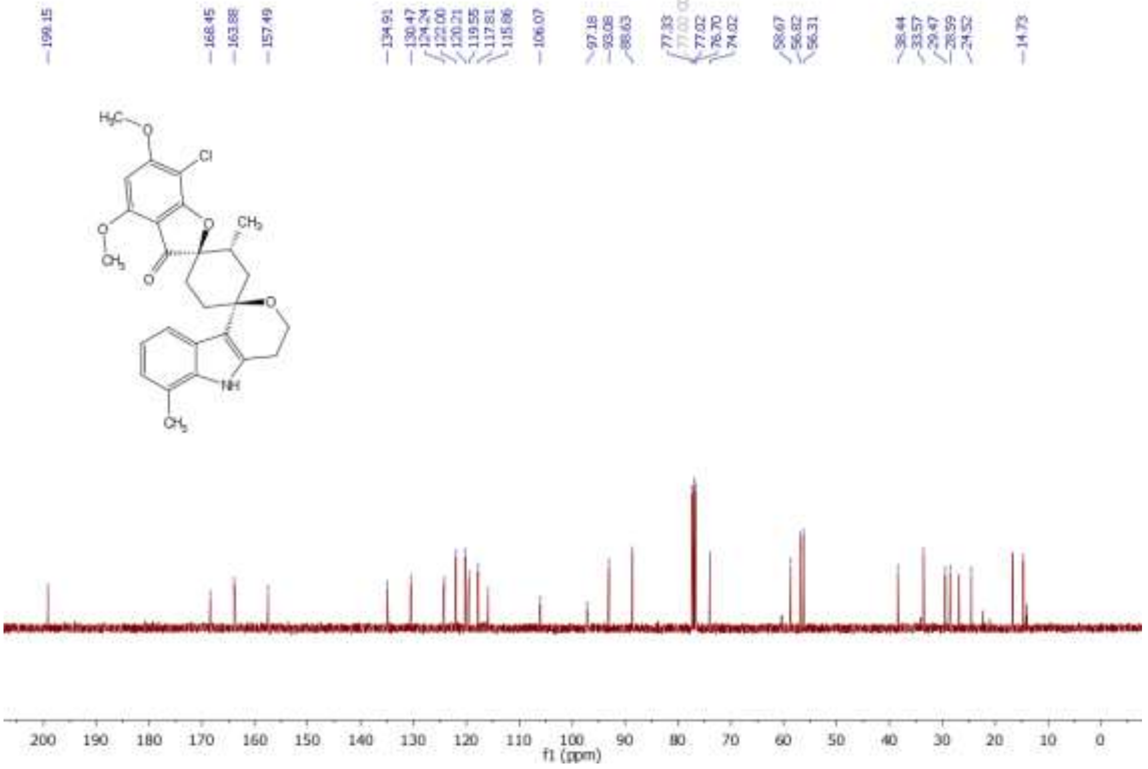

(2*R*,2'*R*,4'*S*)-7-chloro-8''-fluoro-4,6-dimethoxy-2'-methyl-4'',5''-dihydro-3*H*,3''*H*-dispiro[benzofuran-2,1'-cyclohexane-4',1''-pyrano[4,3-*b*]indol]-3-one **7f**

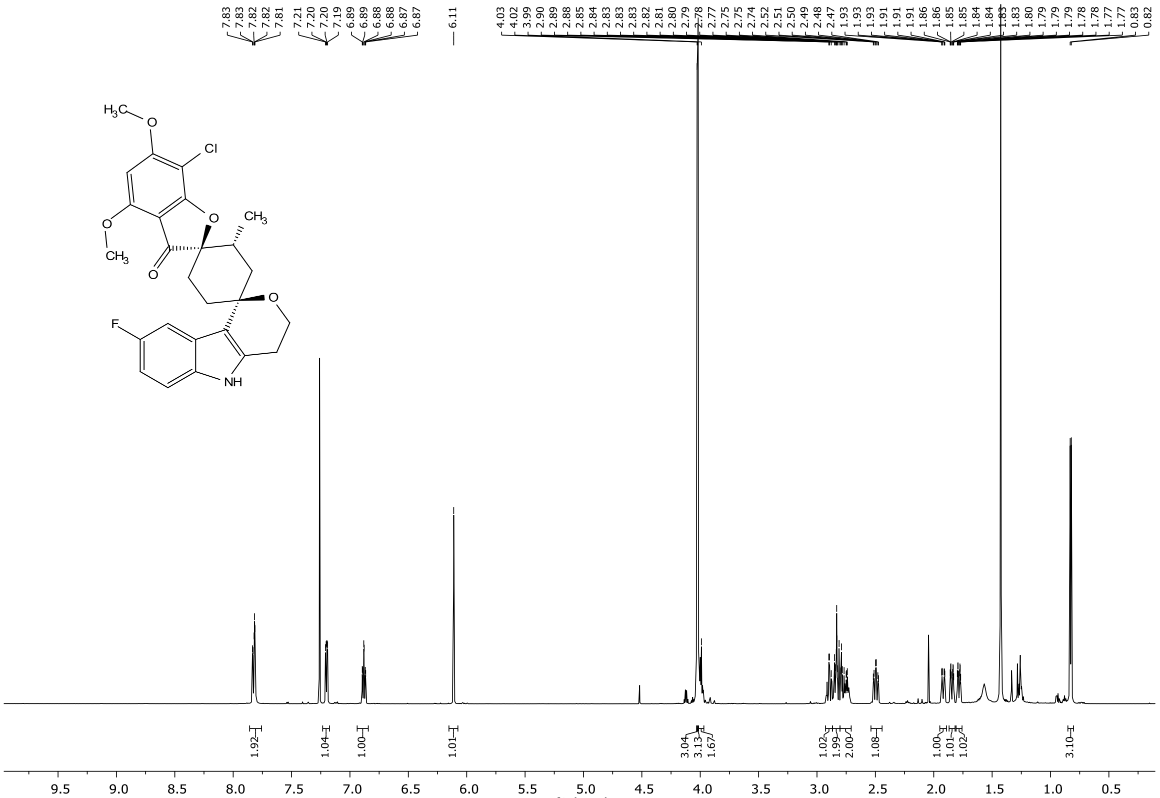
7.83 7.82 7.82 7.81 7.21 7.20 7.20 7.19 6.89 6.89 6.88 6.88 6.87 6.87
6.11
4.03 4.02 3.99 2.90 2.89 2.88 2.85 2.84 2.83 2.83 2.83 2.82 2.81 2.80 2.79 2.78 2.78 2.77 2.75 2.75 2.74 2.52 2.51 2.50 2.49 2.48 2.47 1.93 1.93 1.93 1.93 1.91 1.91 1.91 1.86 1.86 1.85 1.85 1.84 1.84 1.83 1.83 1.80 1.79 1.79 1.79 1.78 1.78 1.77 0.83 0.82
H3C
O
Cl
O
CH3
O
O
O
CH3
F
NH
1.92
1.04
1.00
1.01
3.04
3.13
1.67
1.02
1.99
2.00
1.08
1.00
1.01
1.02
3.10
9.5 9.0 8.5 8.0 7.5 7.0 6.5 6.0 5.5 5.0 4.5 4.0 3.5 3.0 2.5 2.0 1.5 1.0 0.5
f1 (ppm)

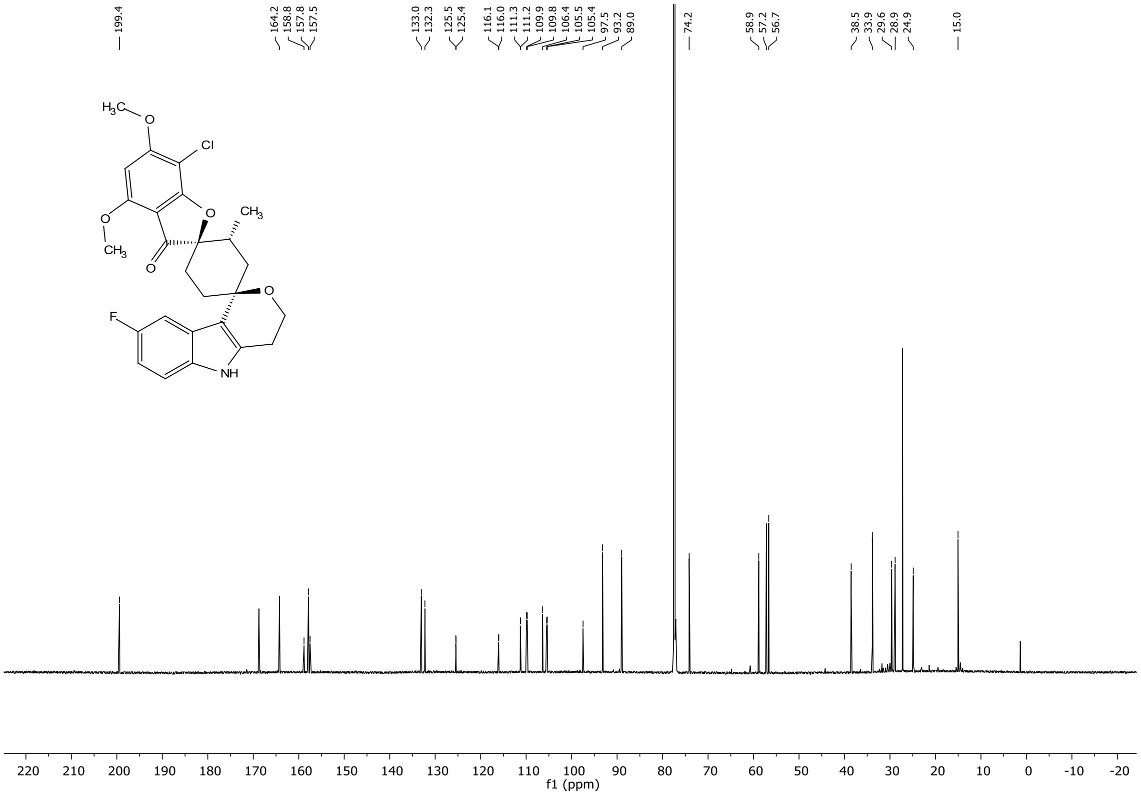
199.4
164.2 158.8 157.8 157.5
133.0 132.3 125.5 125.4 116.1 116.0 111.3 111.2 109.9 109.8 106.4 105.5 105.4 97.5 93.2 89.0
74.2
58.9 57.2 56.7
38.5 33.9 29.6 28.9 24.9
15.0
H3C
O
Cl
O
CH3
O
O
O
CH3
F
NH
220 210 200 190 180 170 160 150 140 130 120 110 100 90 80 70 60 50 40 30 20 10 0 -10 -20
f1 (ppm)

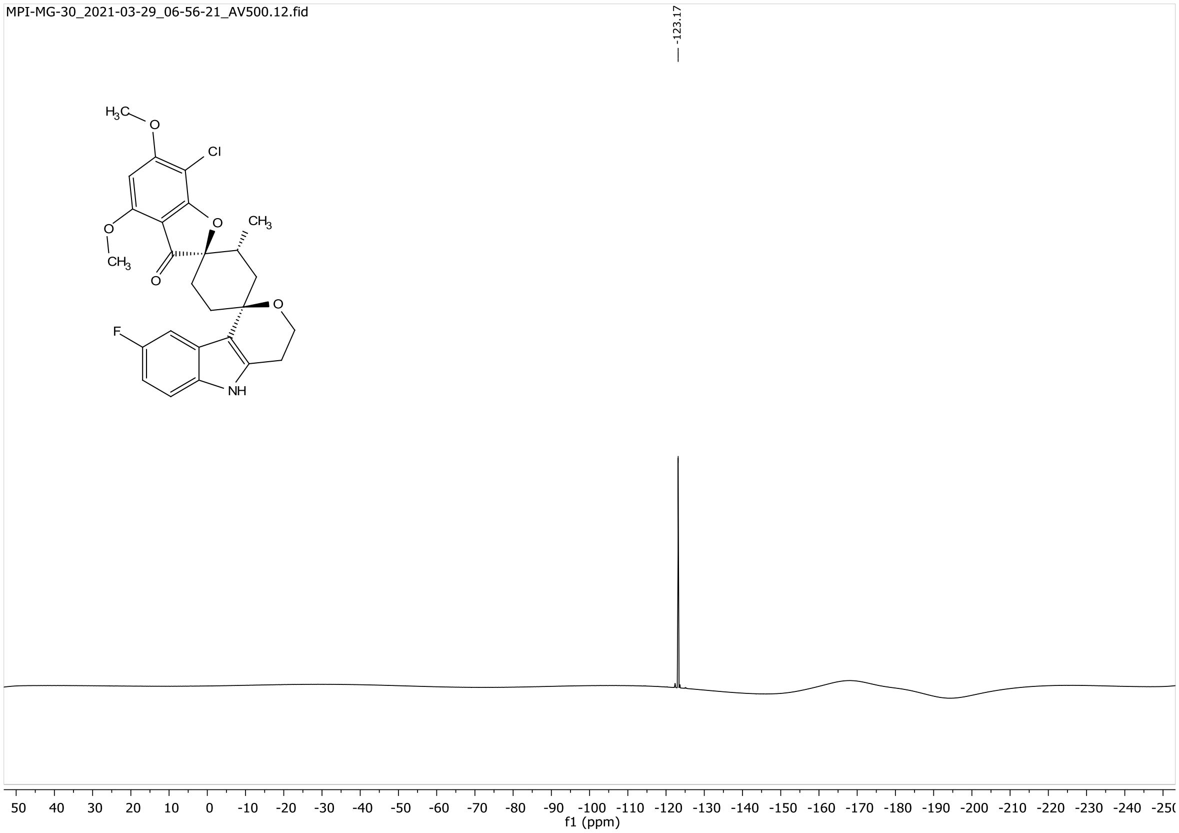
MPI-MG-30_2021-03-29_06-56-21_AV500.12.fid
-123.17
H_3C
O
Cl
O
CH_3
O
CH_3
O
O
F
NH
50 40 30 20 10 0 -10 -20 -30 -40 -50 -60 -70 -80 -90 -100 -110 -120 -130 -140 -150 -160 -170 -180 -190 -200 -210 -220 -230 -240 -250
f1 (ppm)

(2*R*,2'*R*,4'*S*)-7-chloro-8''-chloro-4,6-dimethoxy-2'-methyl-4'',5''-dihydro-3*H*,3''*H*-dispiro[benzofuran-2,1'-cyclohexane-4',1''-pyrano[4,3-*b*]indol]-3-one **7g**

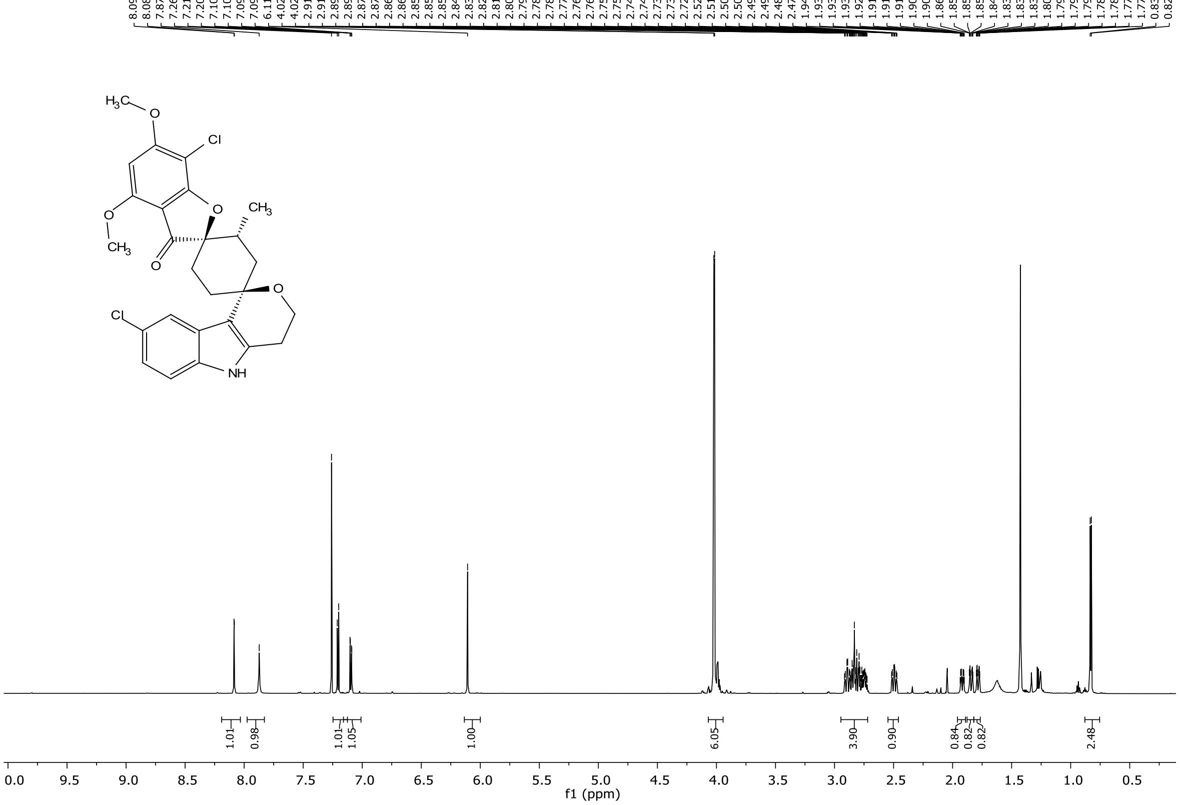

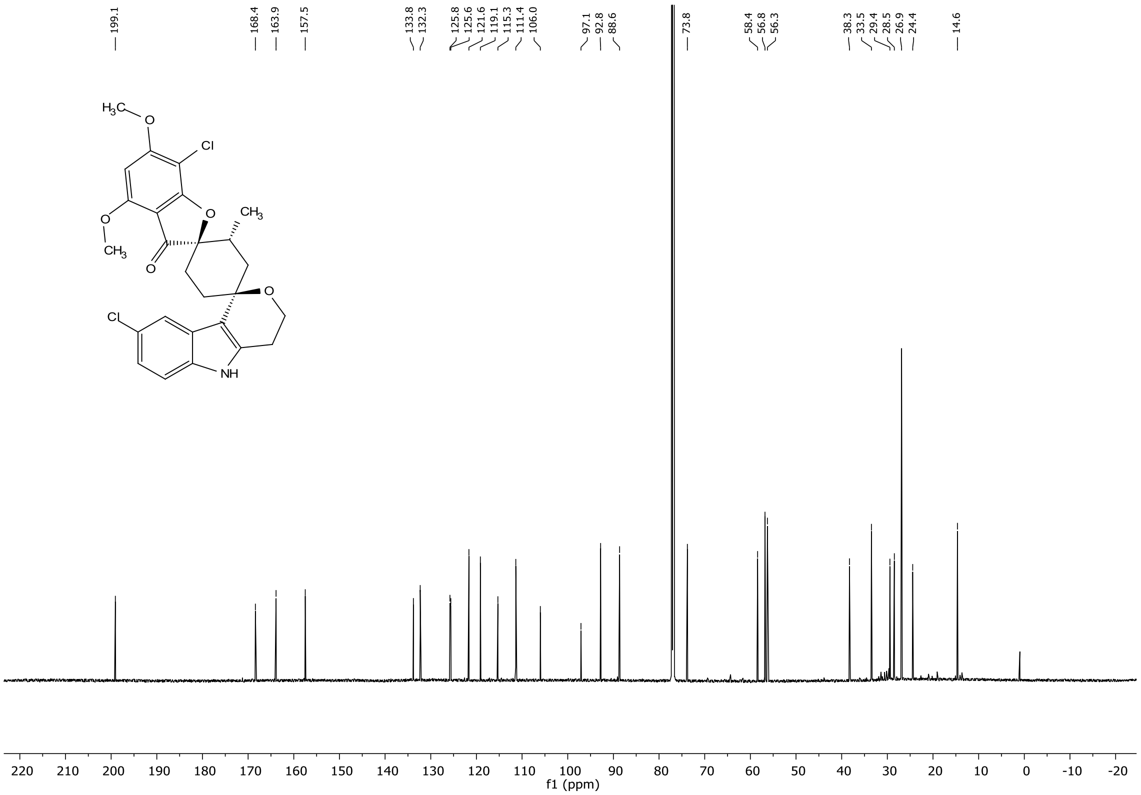

(2*R*,2'*R*,4'*S*)-7-chloro-8''-methoxy-4,6-dimethoxy-2'-methyl-4'',5''-dihydro-3*H*,3''*H*-dispiro[benzofuran-2,1'-cyclohexane-4',1''-pyrano[4,3-*b*]indol]-3-one **7h**

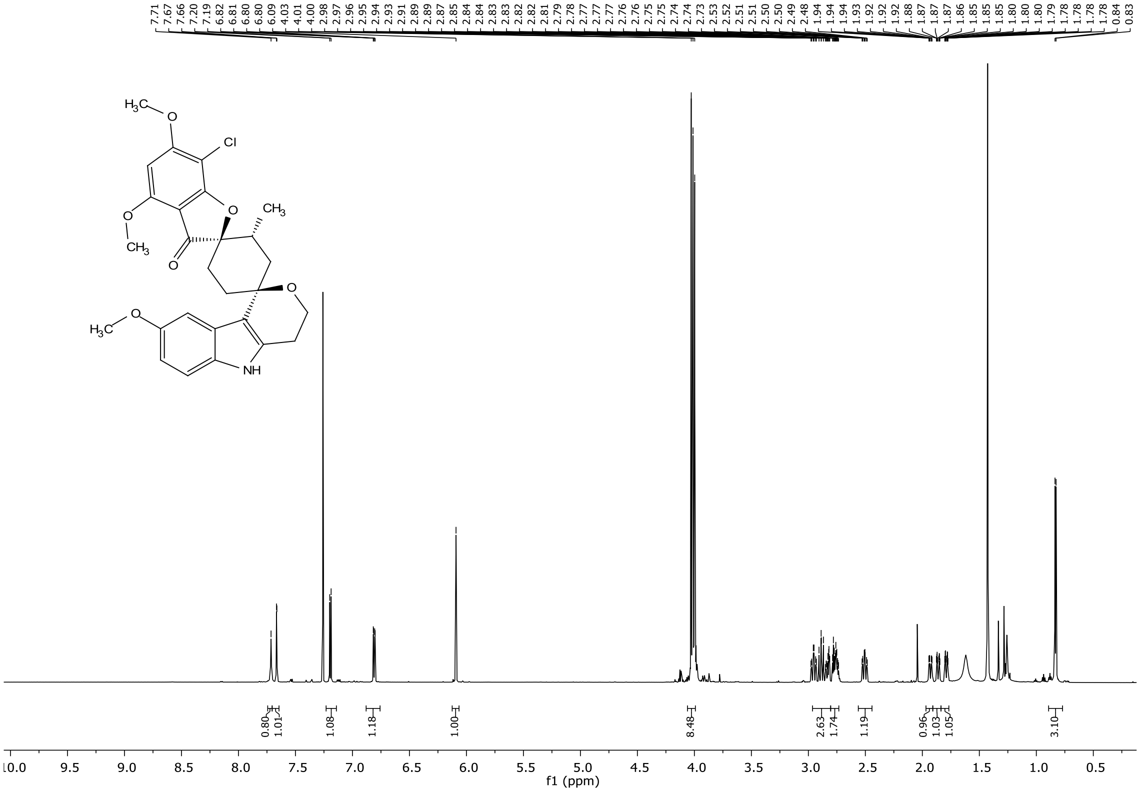

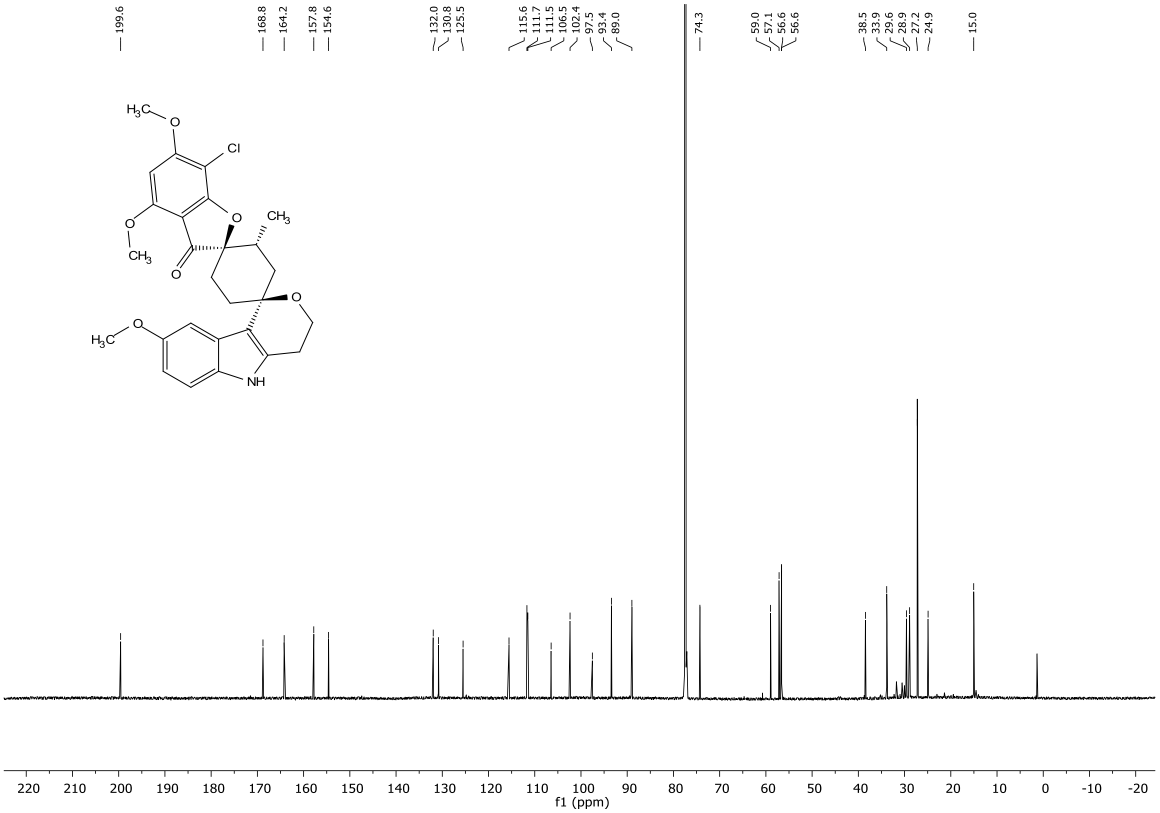

(2*R*,2'*R*,4'*S*)-7-chloro-8''-benzoyl-4,6-dimethoxy-2'-methyl-4'',5''-dihydro-3*H*,3''*H*-dispiro[benzofuran-2,1'-cyclohexane-4',1''-pyrano[4,3-*b*]indol]-3-one **7i**

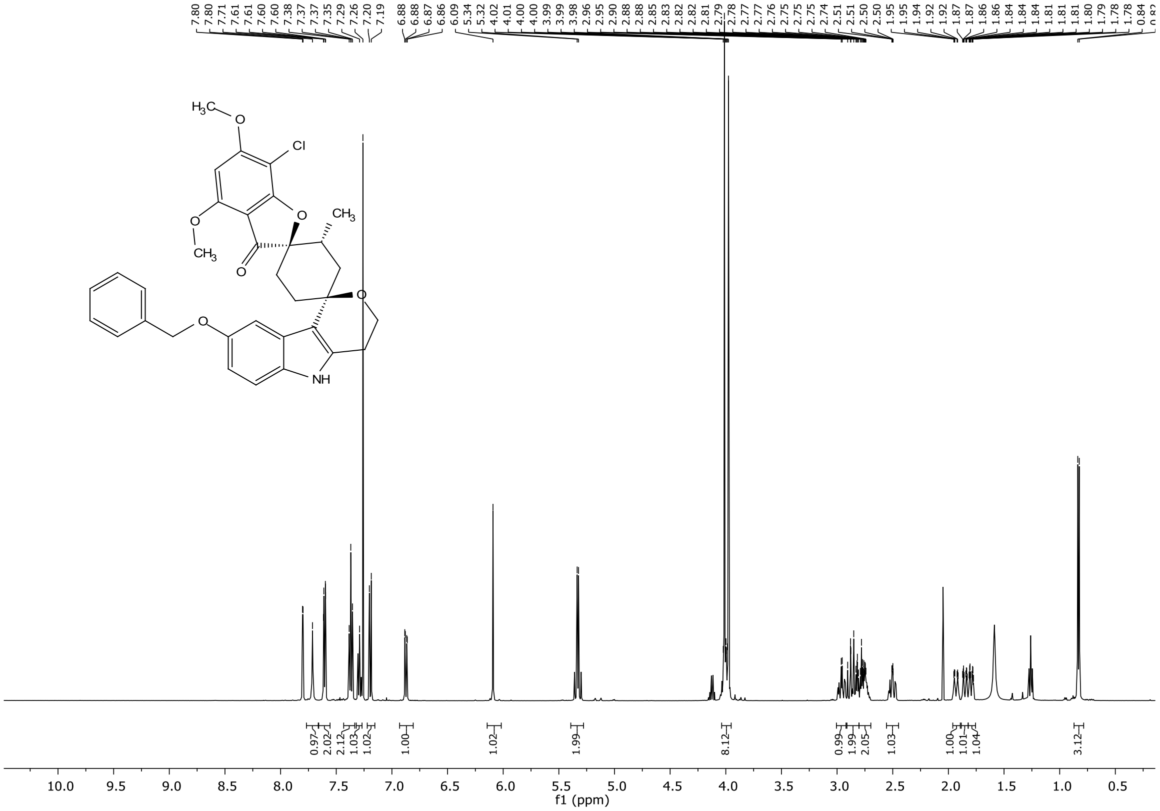

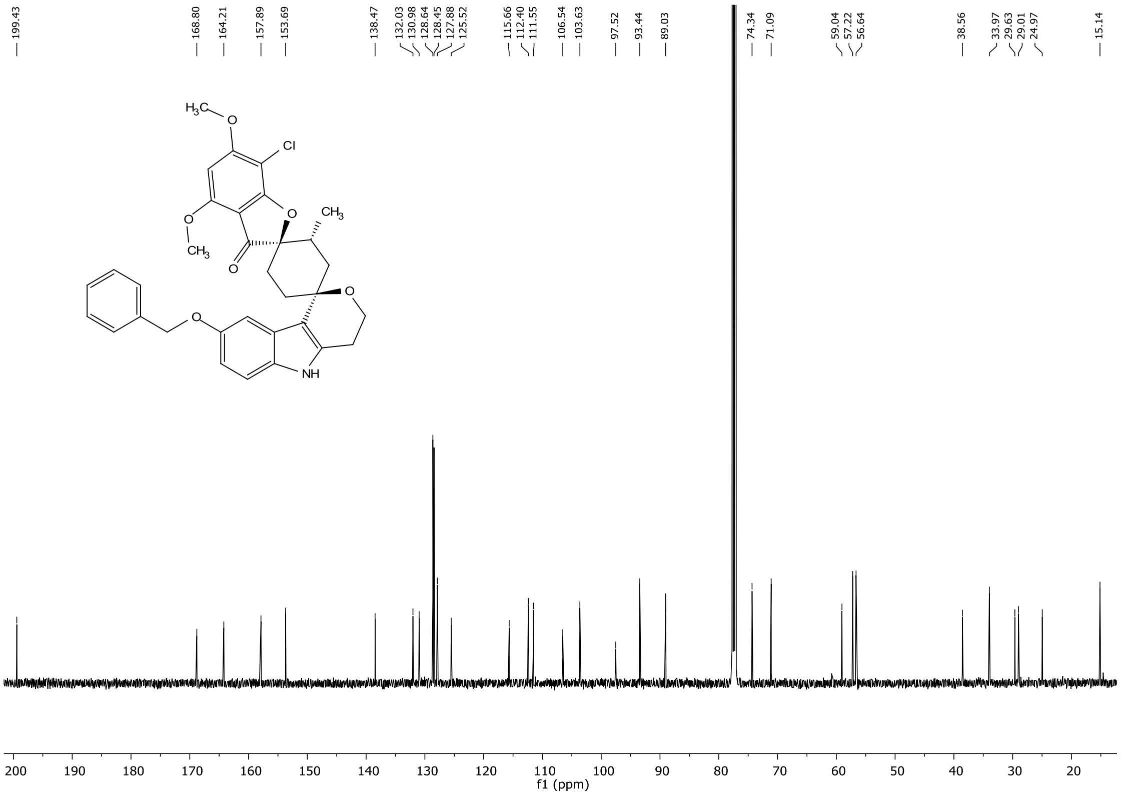

methyl-(2*R*,2'*R*,4'*S*)-7-chloro-4,6-dimethoxy-2'-methyl-3-oxo-4'',5''-dihydro-3*H*,3''*H*-dispiro[benzofuran-2,1'-cyclohexane-4',1''-pyrano[4,3-*b*]indole]-8''-carboxylate **7j**

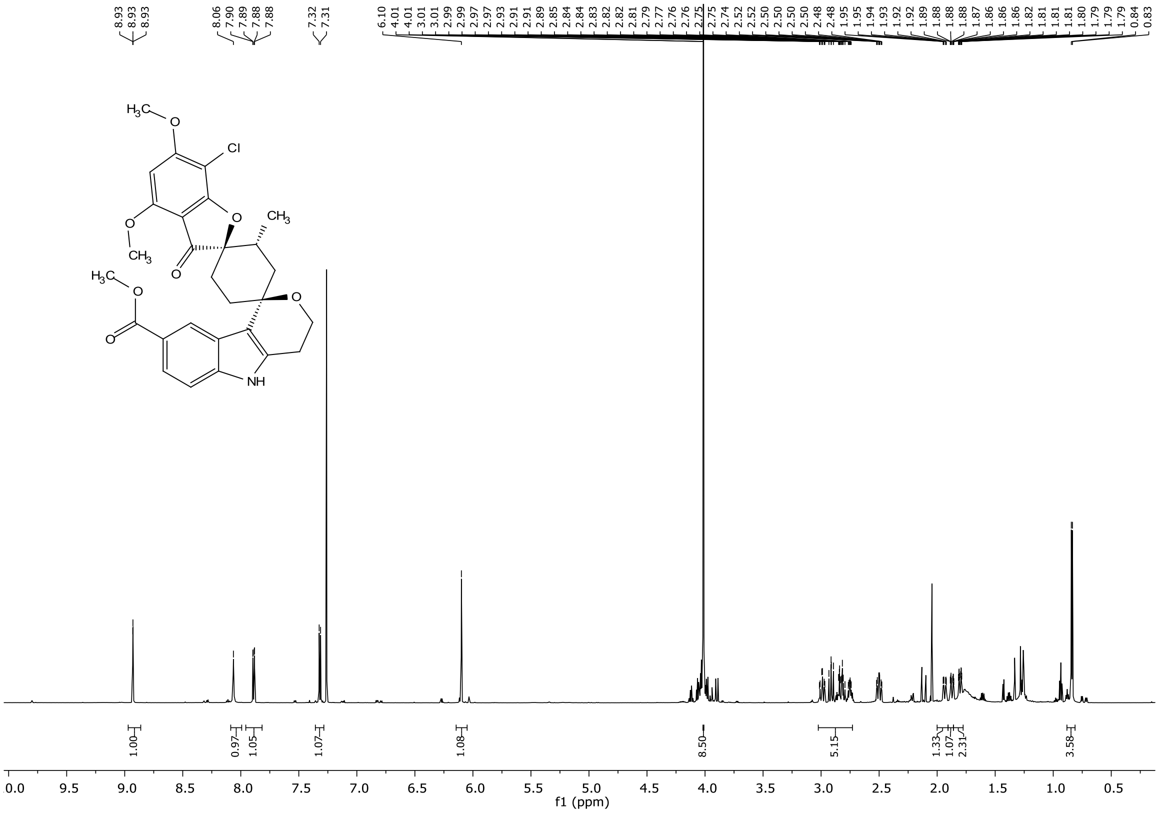

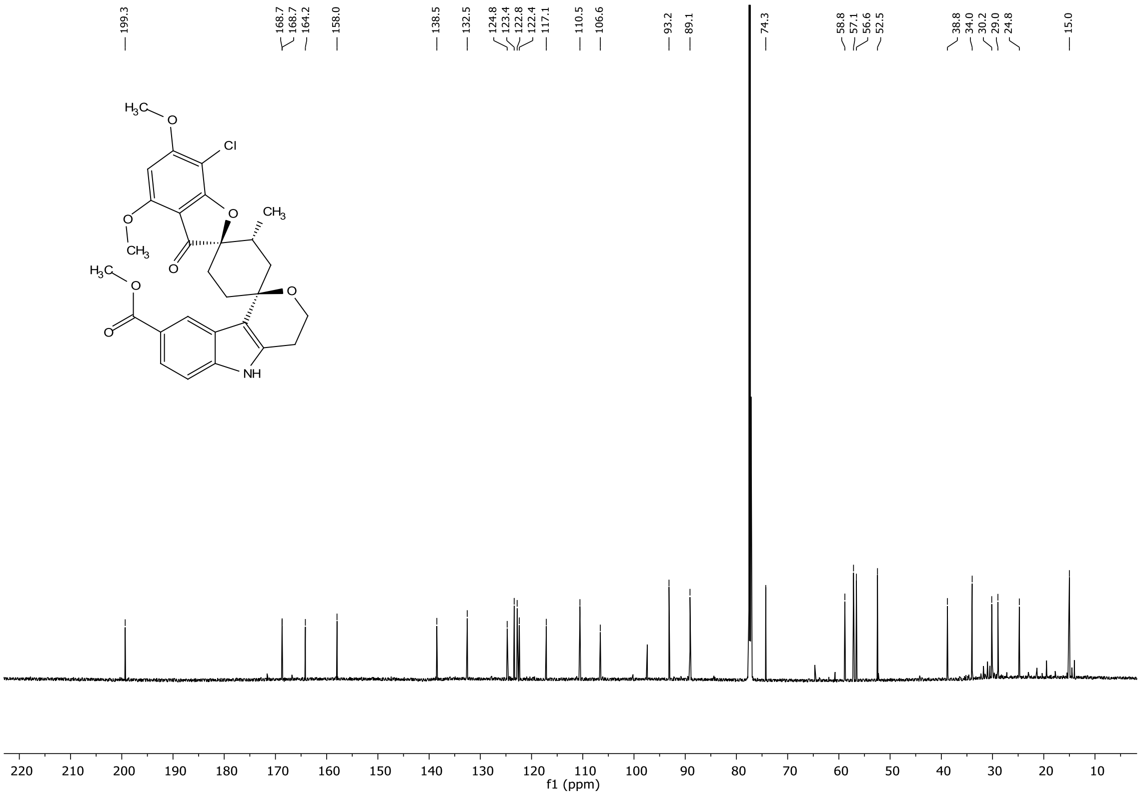

(2*R*,2'*R*,4'*S*)-7-chloro-8''-hydroxy-4,6-dimethoxy-2'-methyl-4'',5''-dihydro-3*H*,3''*H*-dispiro[benzofuran-2,1'-cyclohexane-4',1''-pyrano[4,3-*b*]indol]-3-one **7k**

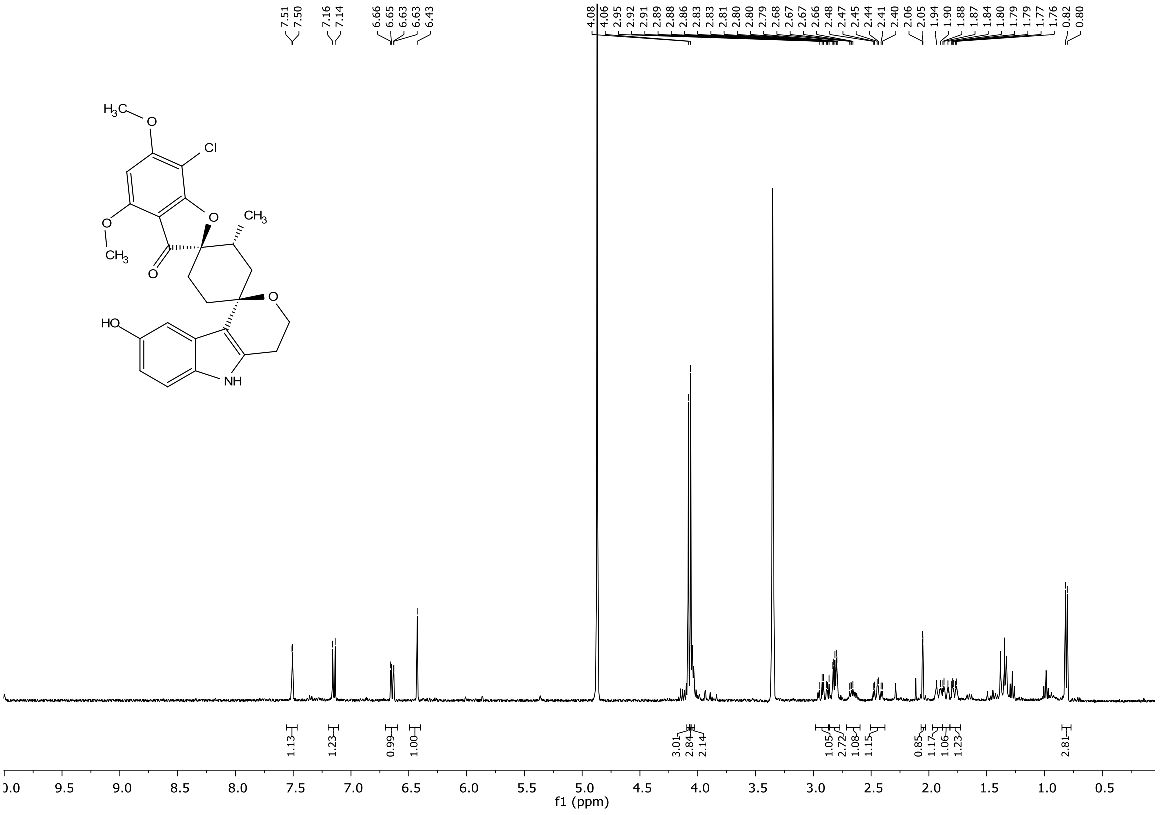

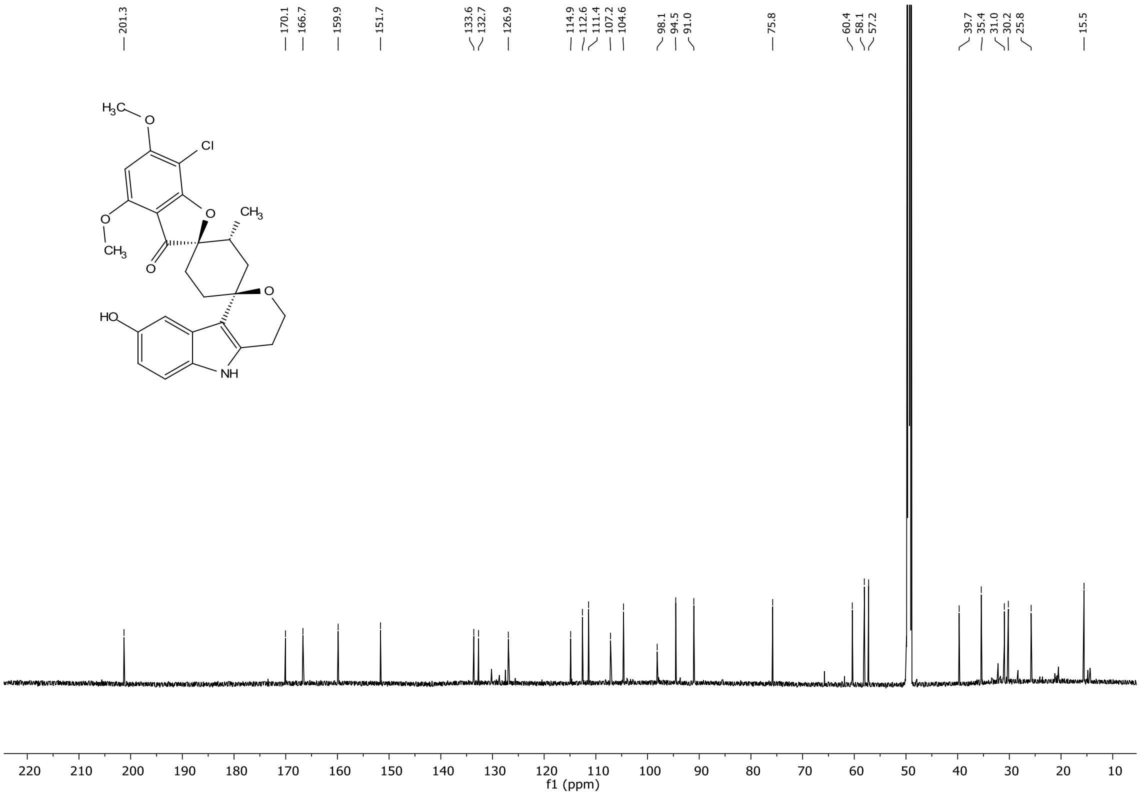

(2*R*,2'*R*,4'*S*)-7-chloro-4,6-dimethoxy-2',5'',8''-trimethyl-4'',5''-dihydro-3*H*,3''*H*-dispiro[benzofuran-2,1'-cyclohexane-4',1''-pyrano[4,3-*b*]indol]-3-one **7l**

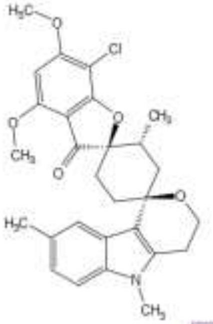

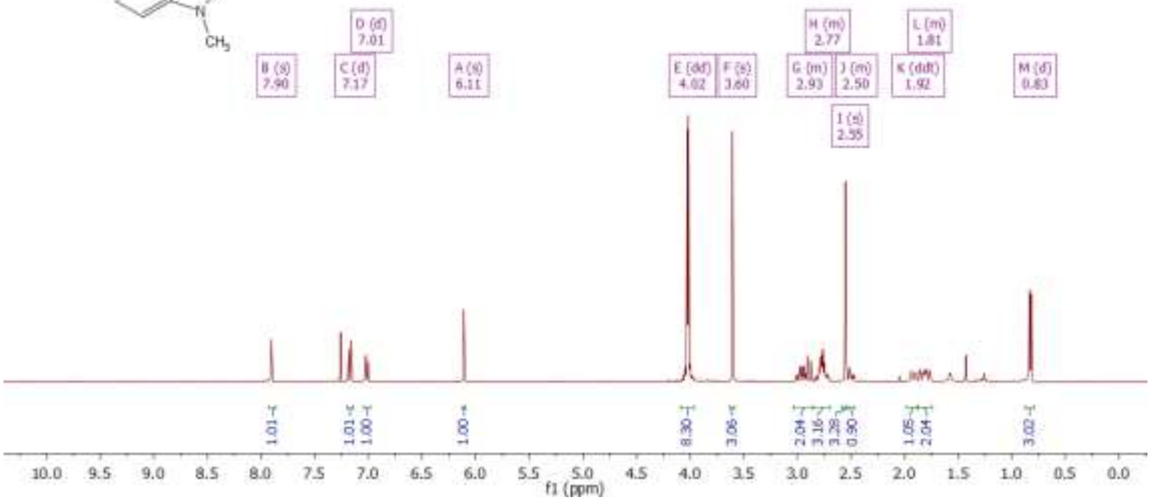

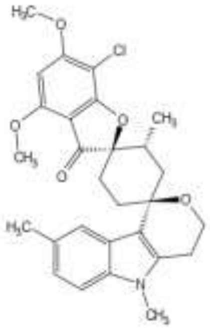

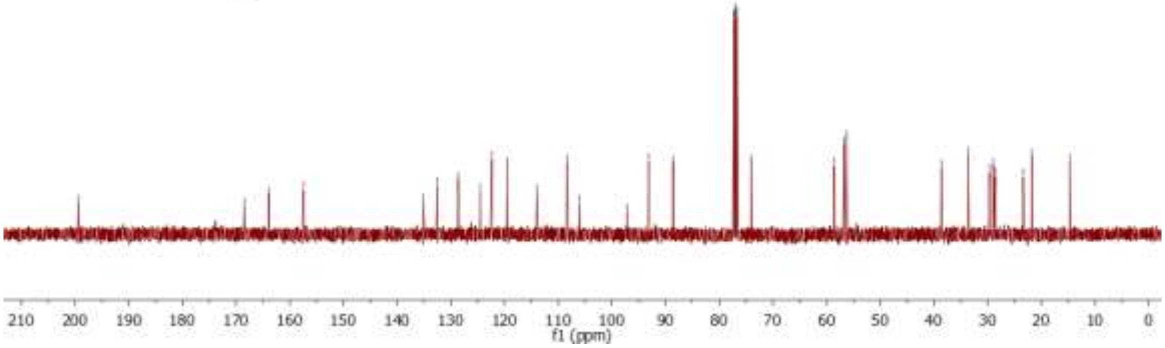

(2*R*,2'*R*,4'*S*)-5''-benzyl-7-chloro-4,6-dimethoxy-2',8''-dimethyl-4'',5''-dihydro-3*H*,3''*H*-dispiro[benzofuran-2,1'-cyclohexane-4',1''-pyrano[4,3-*b*]indol]-3-one **7m**

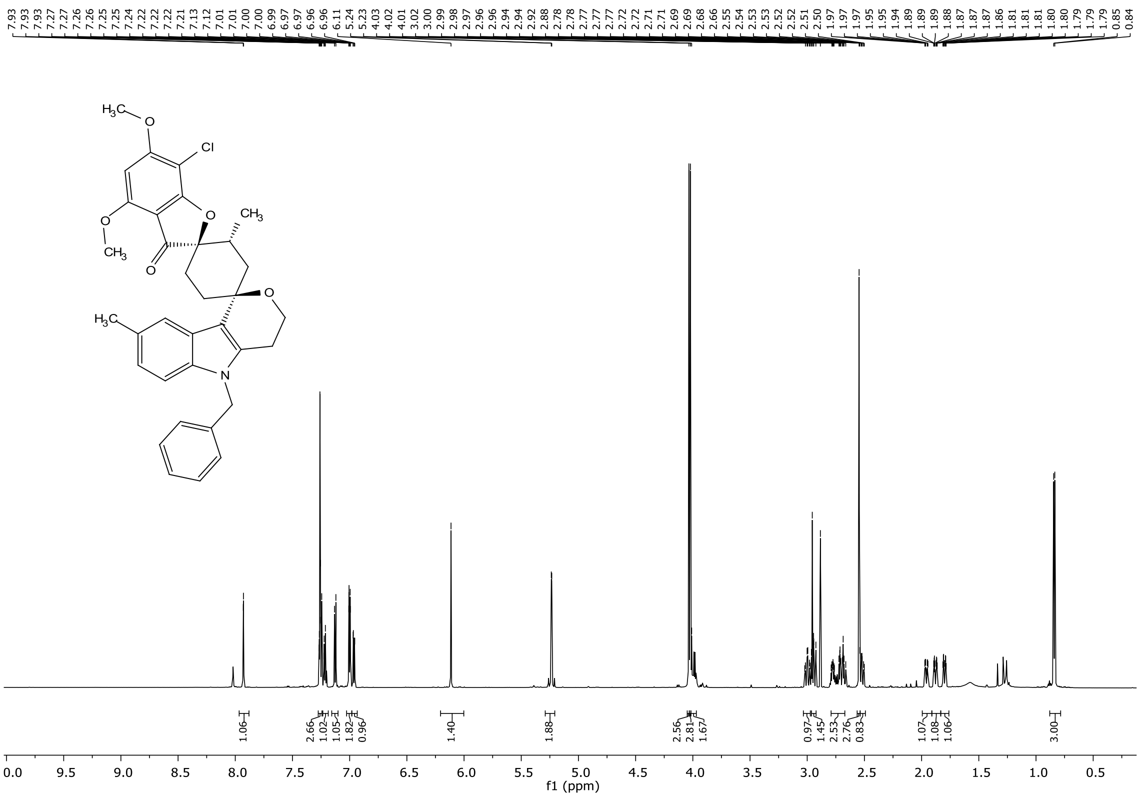

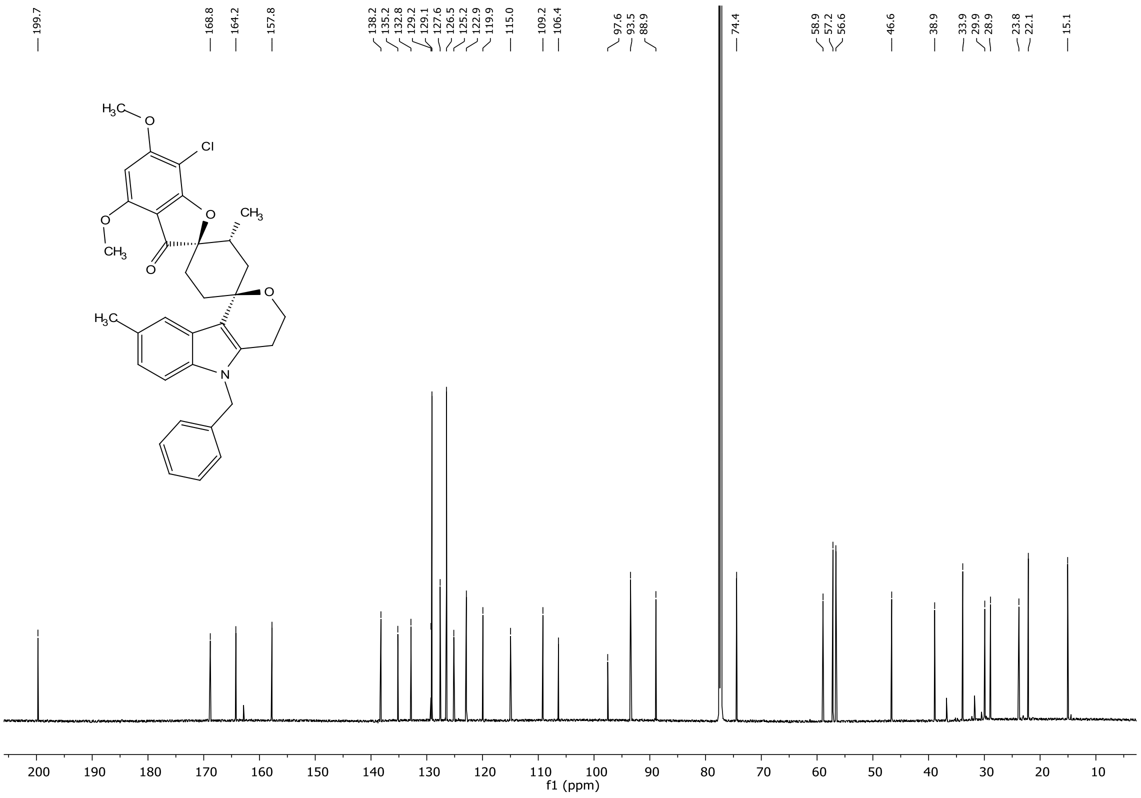

(2*R*,2'*R*,4'*S*)-7-chloro-4-hydroxy-6-methoxy-2',8''-dimethyl-4'',5''-dihydro-3*H*,3''*H*-dispiro[benzofuran-2,1'-cyclohexane-4',1''-pyrano[4,3-*b*]indol]-3-one **7n**

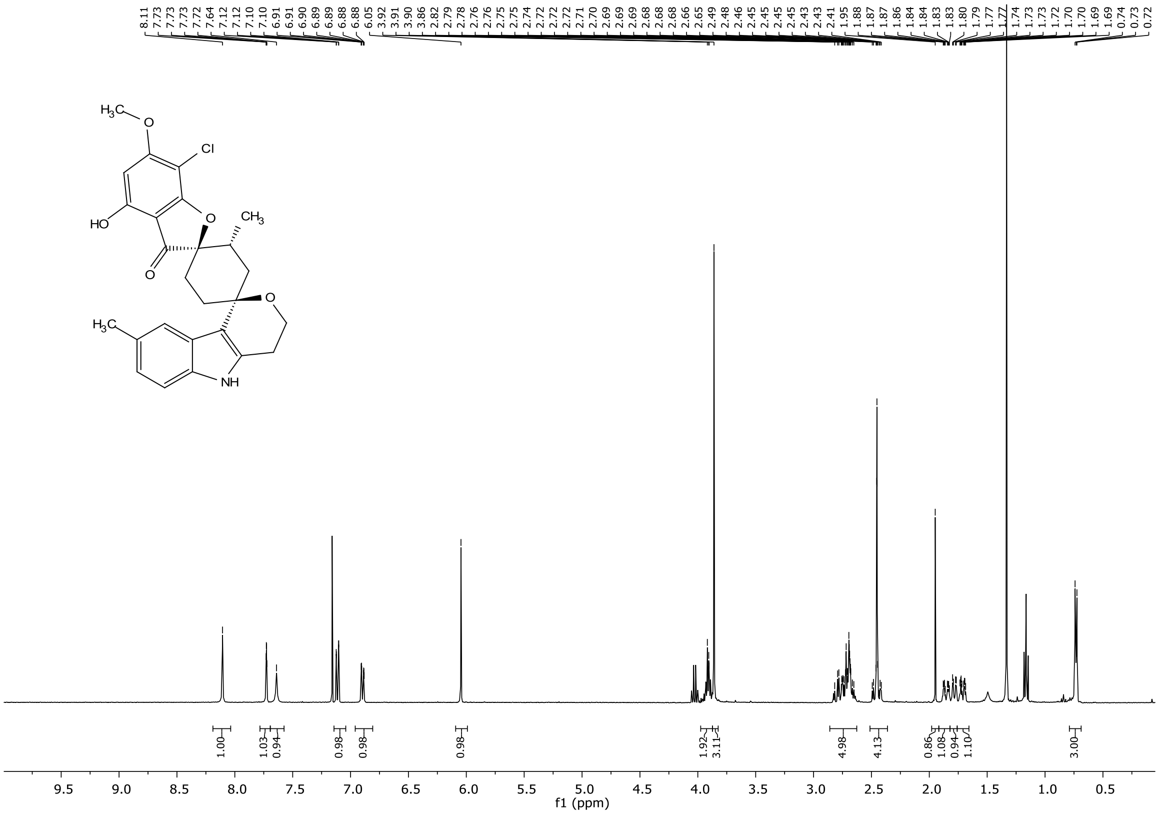

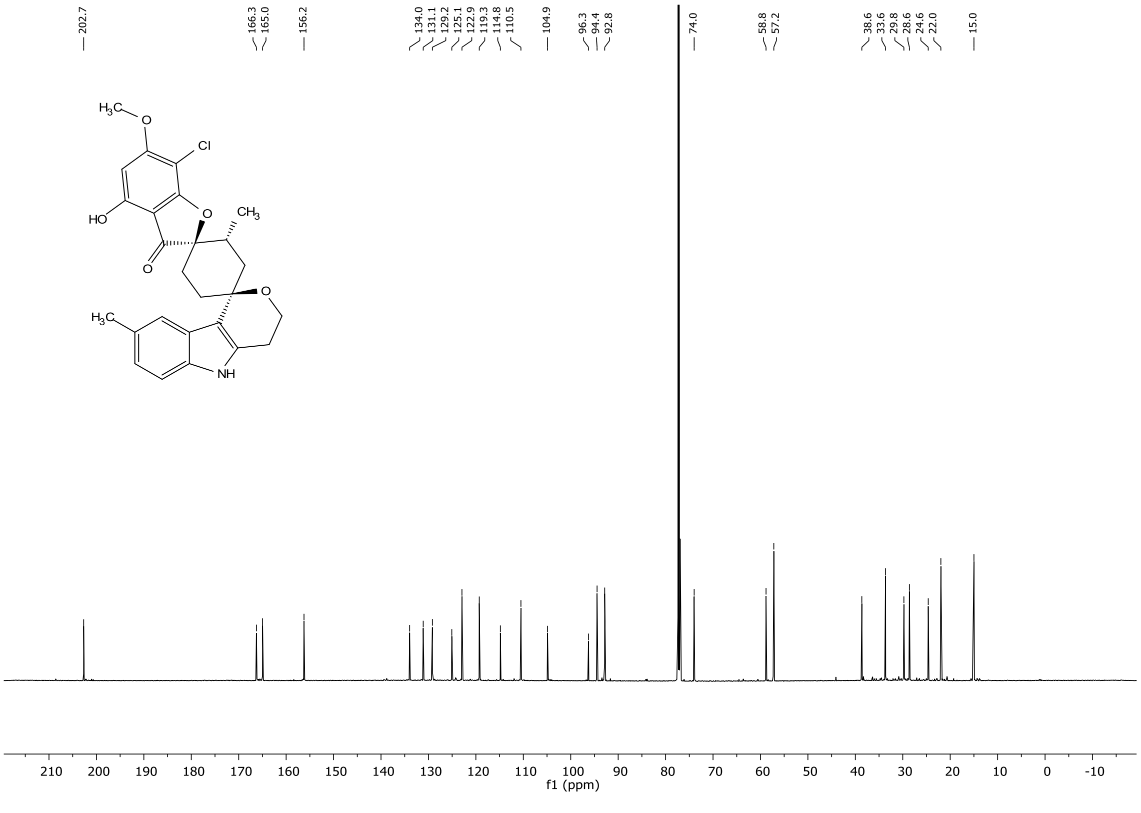

(2*R*,2'*R*,4'*S*)-4-(allyloxy)-7-chloro-6-methoxy-2',8''-dimethyl-4'',5''-dihydro-3*H*,3''*H*-dispiro[benzofuran-2,1'-cyclohexane-4',1''-pyrano[4,3-*b*]indol]-3-one **7o**

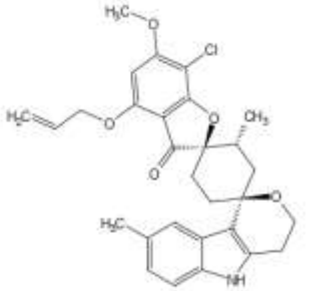

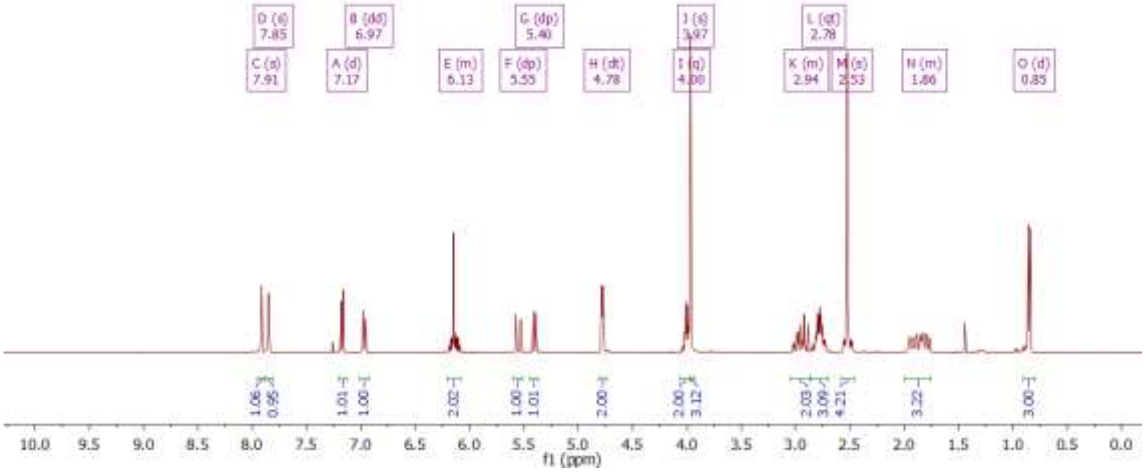

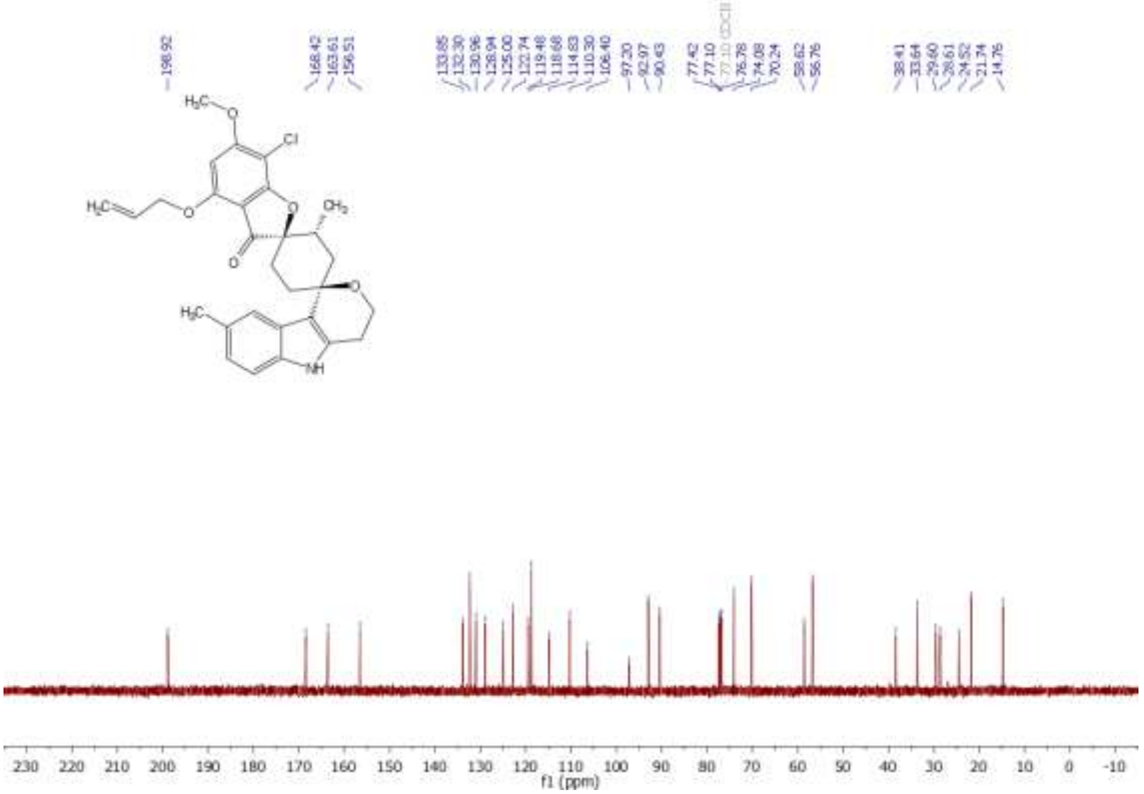

(2*R*,2'*R*,4'*S*)-4-(benzyloxy)-7-chloro-6-methoxy-2',8''-dimethyl-4'',5''-dihydro-3*H*,3''*H*-dispiro[benzofuran-2,1'-cyclohexane-4',1''-pyrano[4,3-*b*]indol]-3-one **7p**

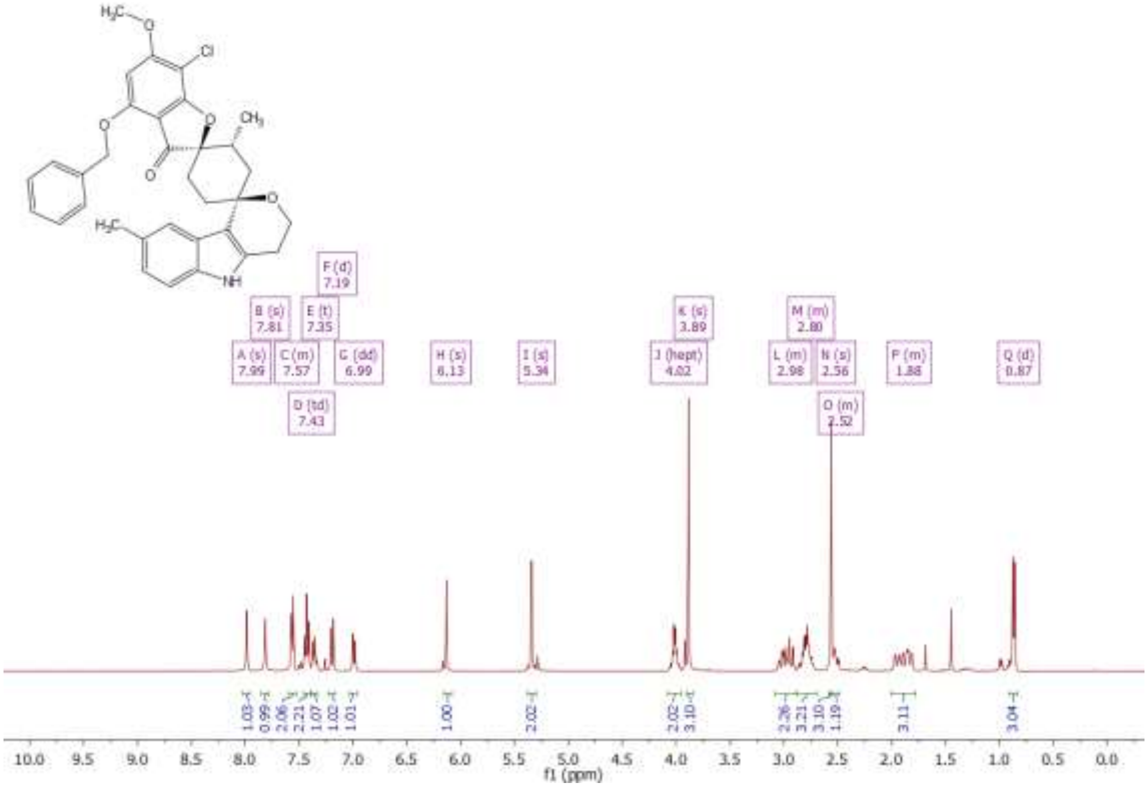

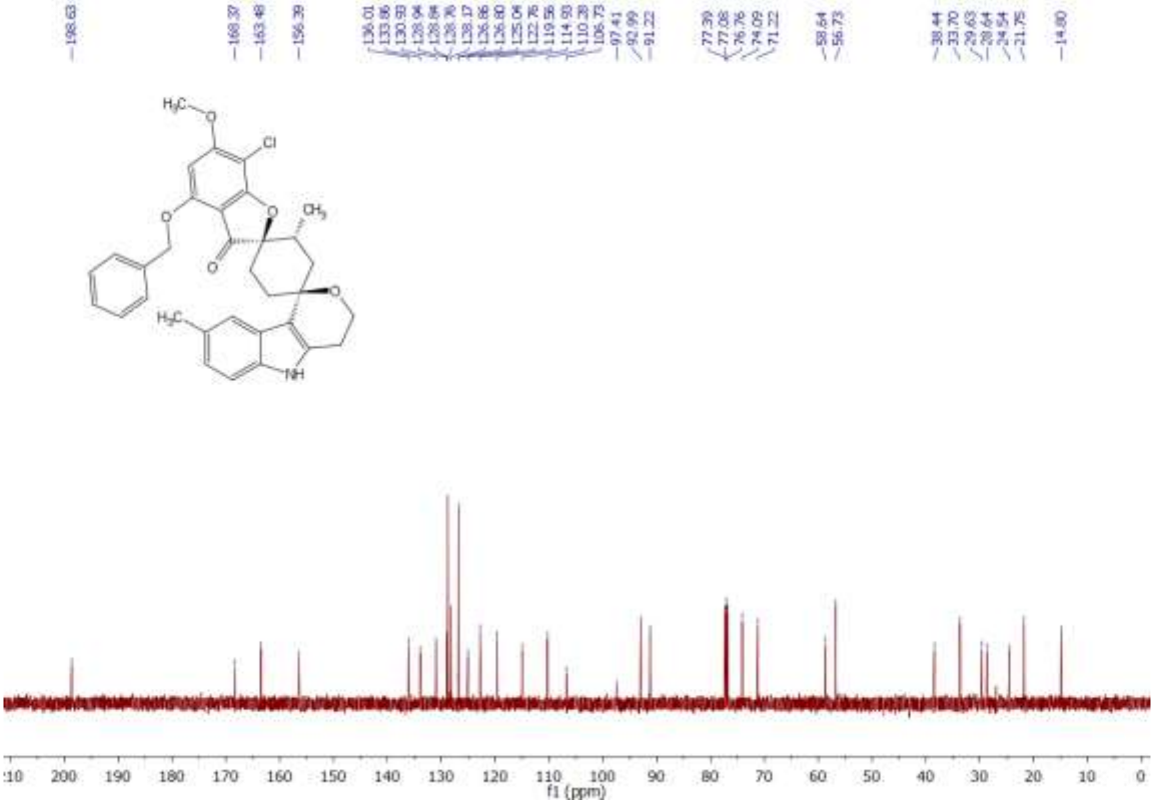

(2*R*,2'*R*)-4,6-dimethoxy-2',8''-dimethyl-4'',5''-dihydro-3H,3''H-dispiro[benzofuran-2,1'-cyclohexane-4',1''-pyrano[4,3-b]indol]-3-one **7q**

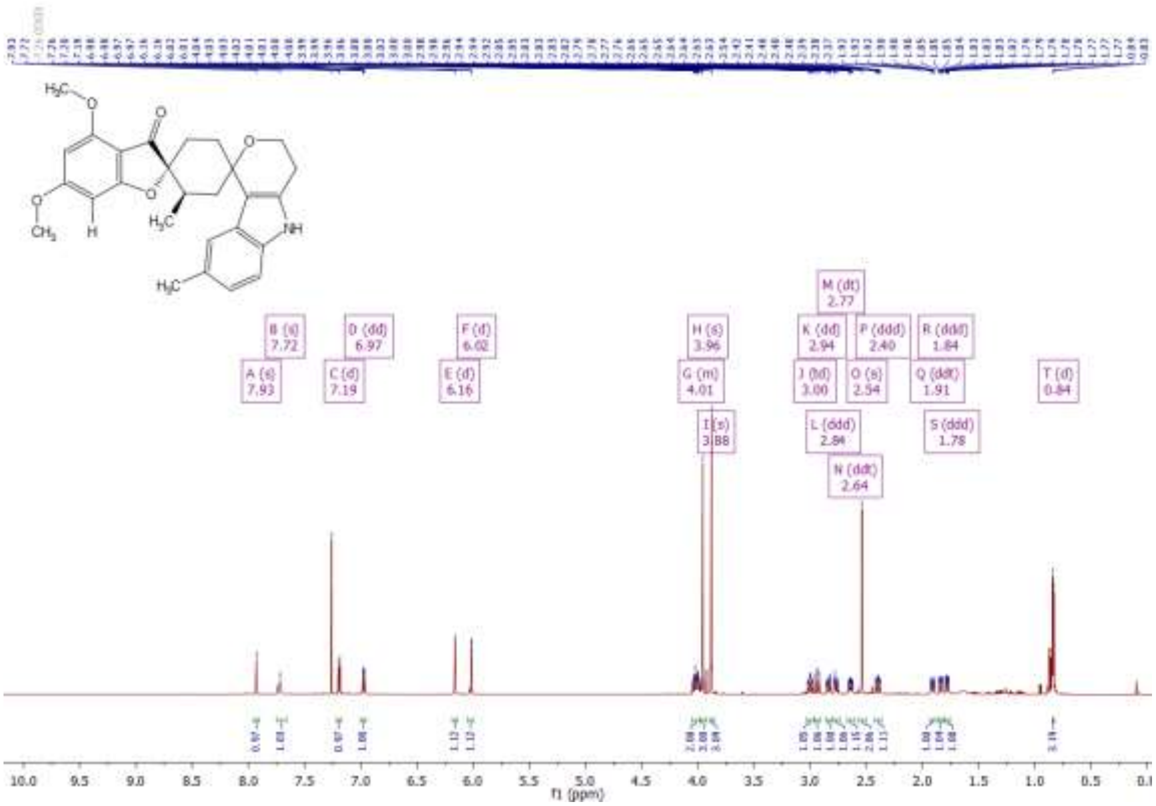

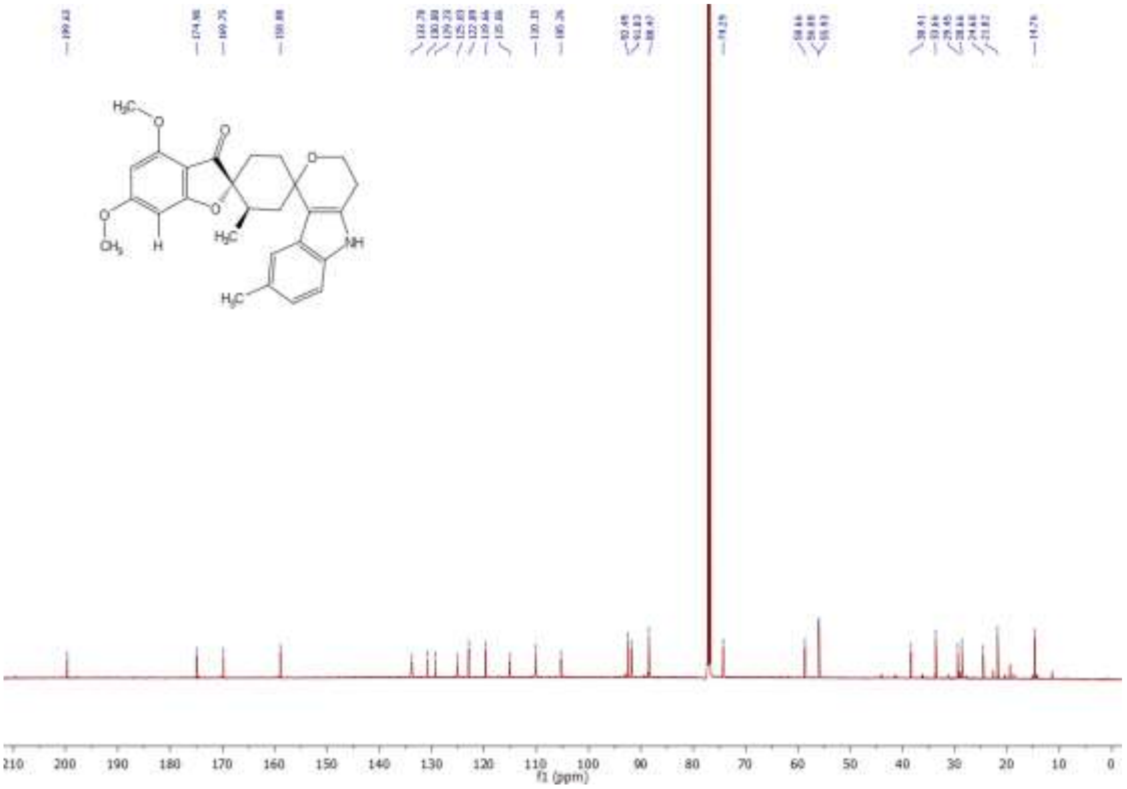

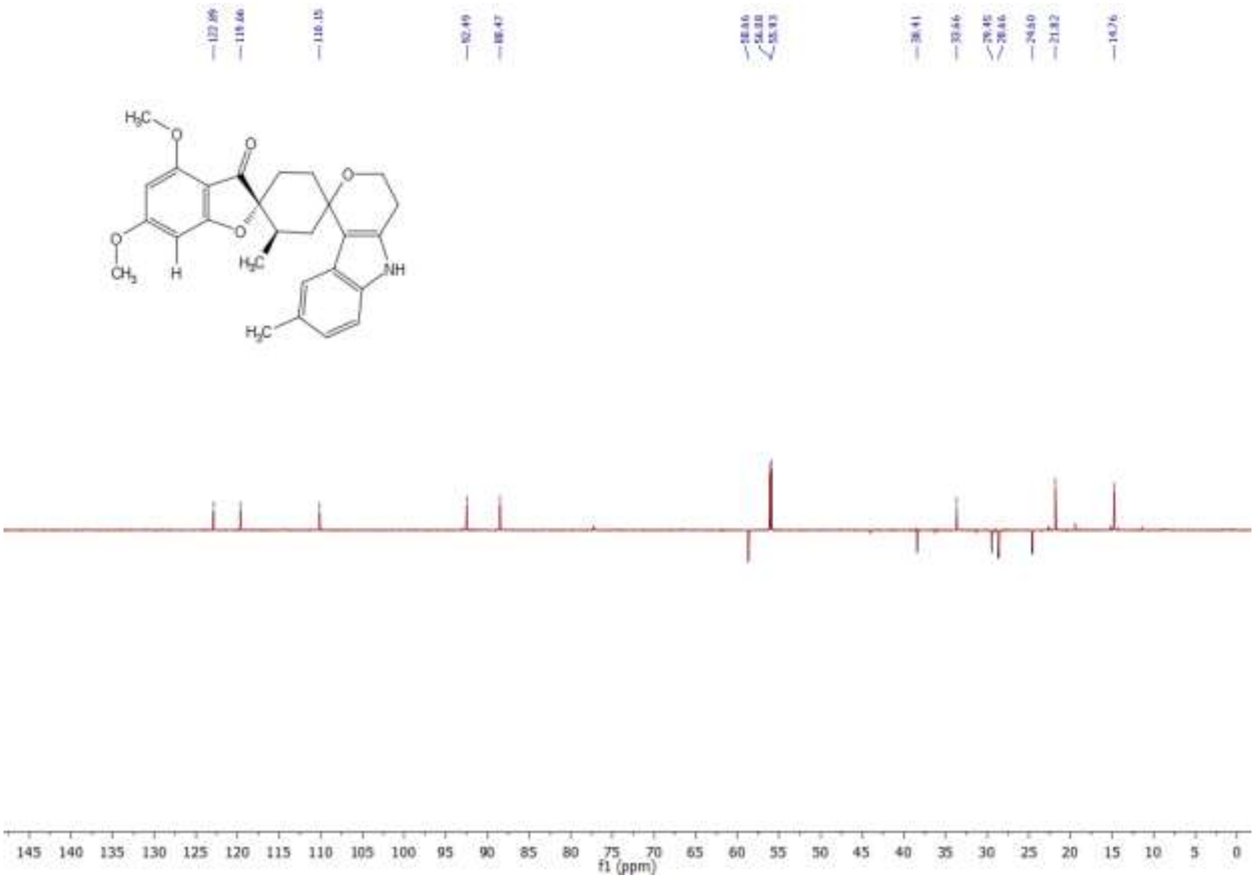
145 140 135 130 125 120 115 110 105 100 95 90 85 80 75 70 65 60 55 50 45 40 35 30 25 20 15 10 5 0
f1 (ppm)

(2R,2'R)-4,6-dimethoxy-2',7,8''-trimethyl-4'',5''-dihydro-3H,3''H-dispiro[benzofuran-2,1'-cyclohexane-4',1''-pyrano[4,3-b]indol]-3-one **7r**

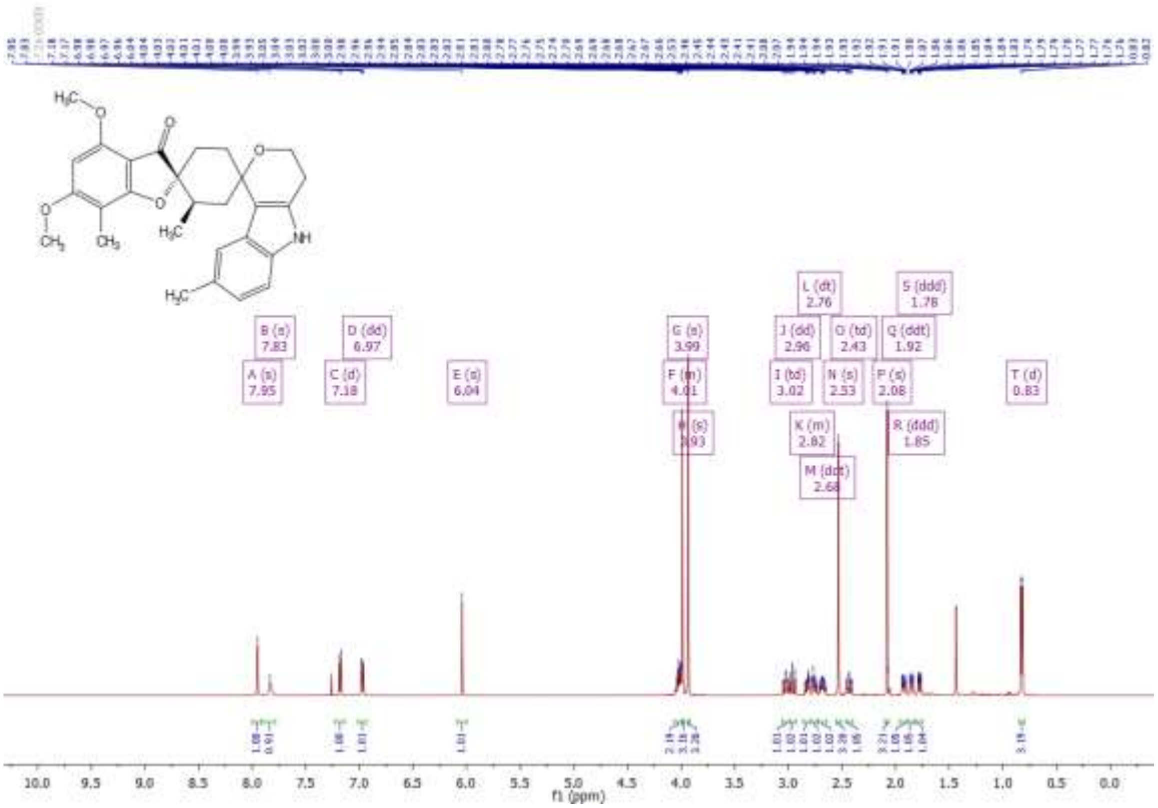

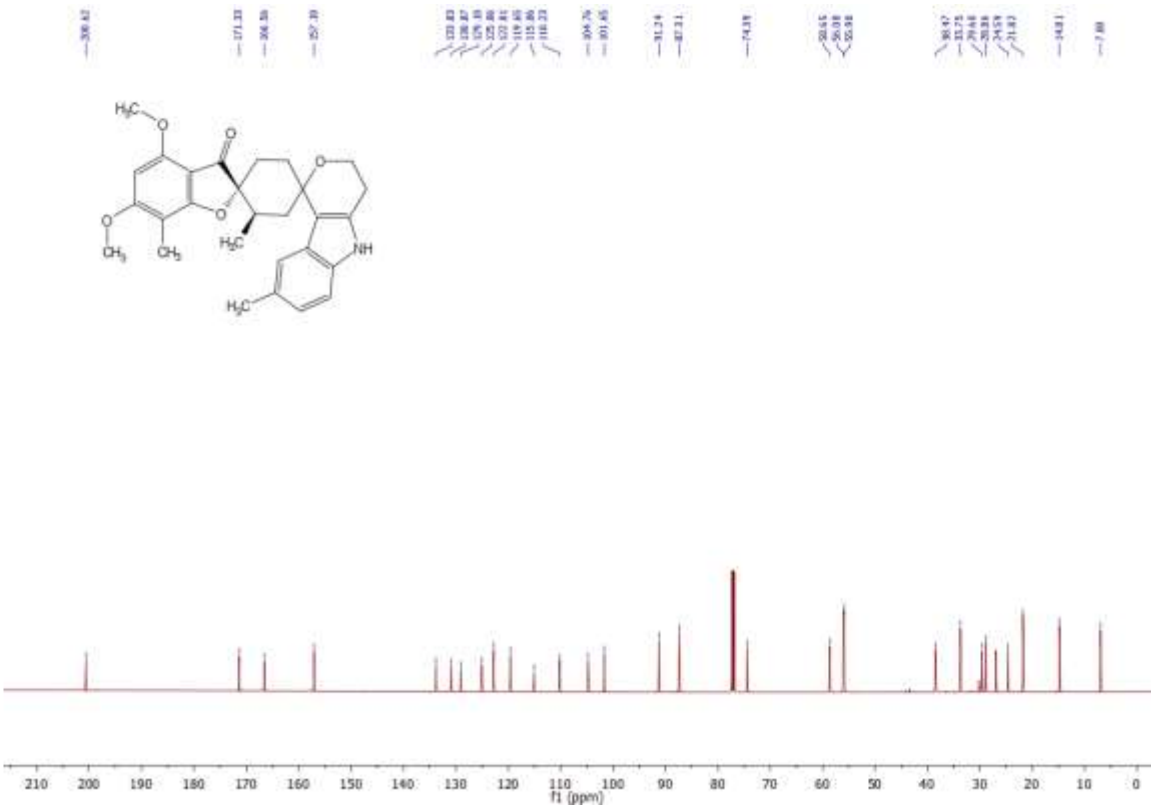

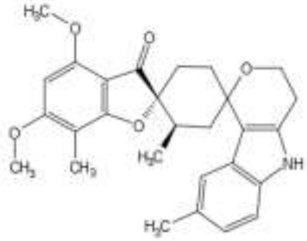

O
NH

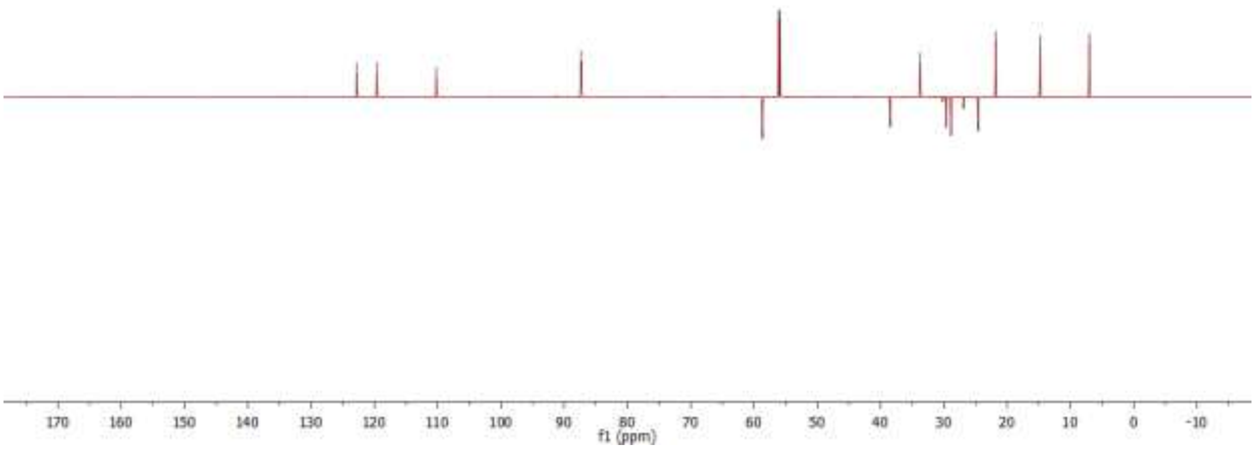

170
160
150
140
130
120
110
100
90
80
70
60
50
40
30
20
10
0
-10
f1 (ppm)

(2R,2'R)-4,6-dimethoxy-7-(4-methoxyphenyl)-2',8''-dimethyl-4'',5''-dihydro-3H,3''H-dispiro[benzofuran-2,1'-cyclohexane-4',1''-pyrano[4,3-b]indol]-3-one **7s**

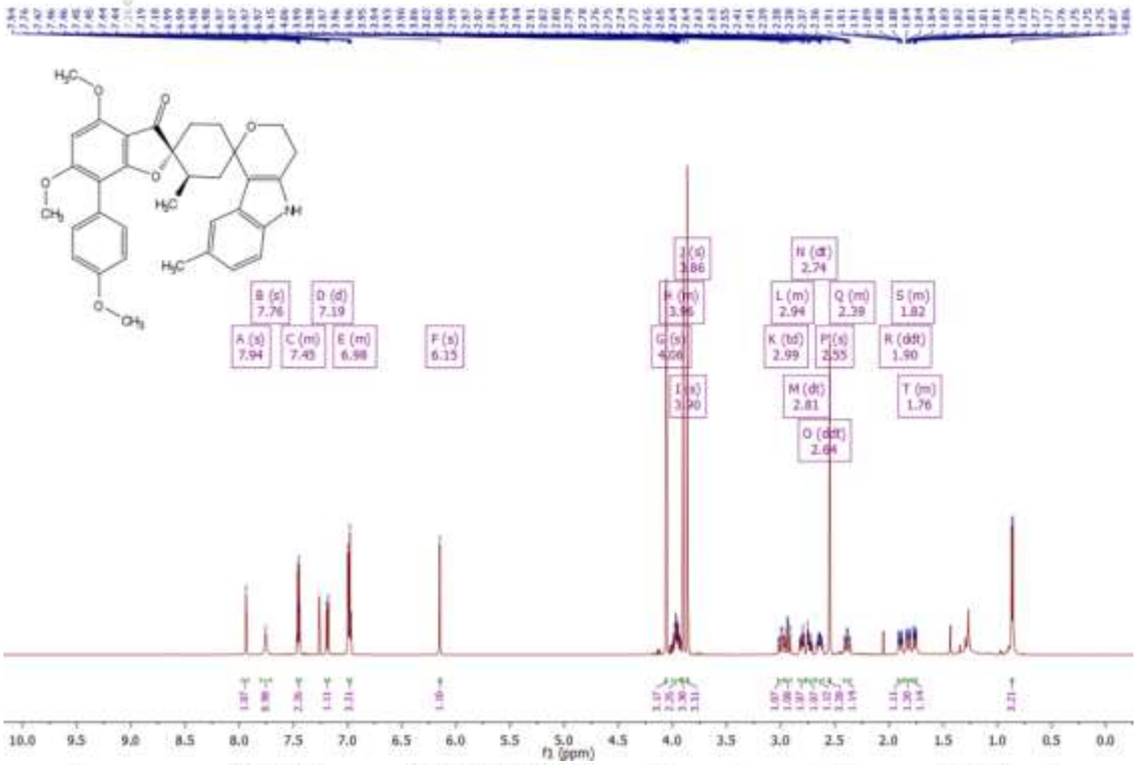

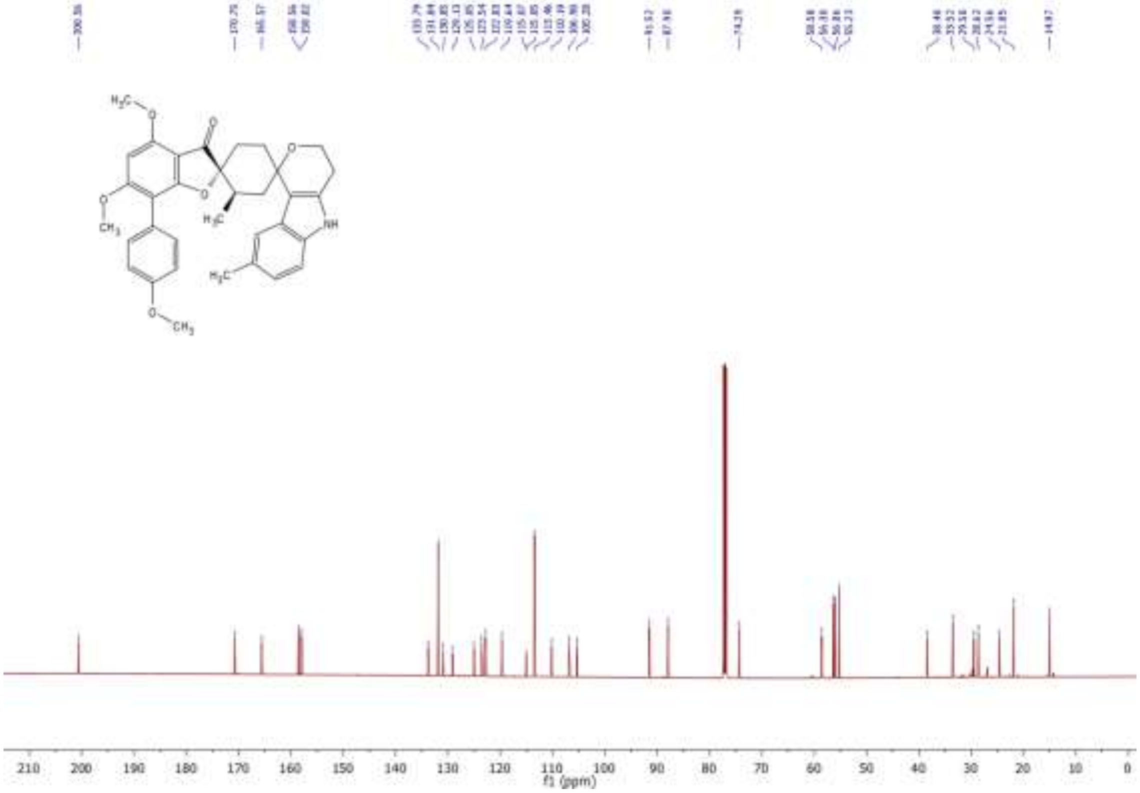

(2*R*,2'*R*)-7-chloro-4,6-dimethoxy-2'-methyl-3'',4''-dihydro-3*H*-dispiro[benzofuran-2,1'-cyclohexane-4',1''-pyrano[4,3-*b*]benzofuran]-3-one **8**

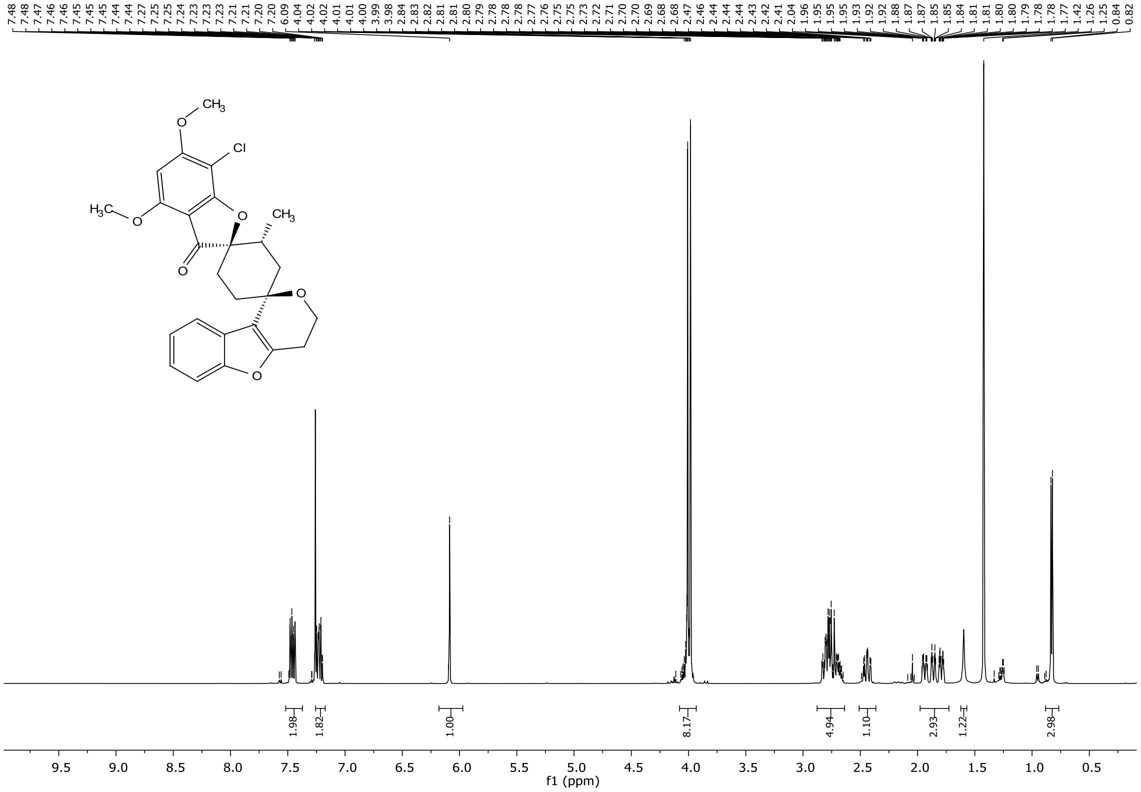

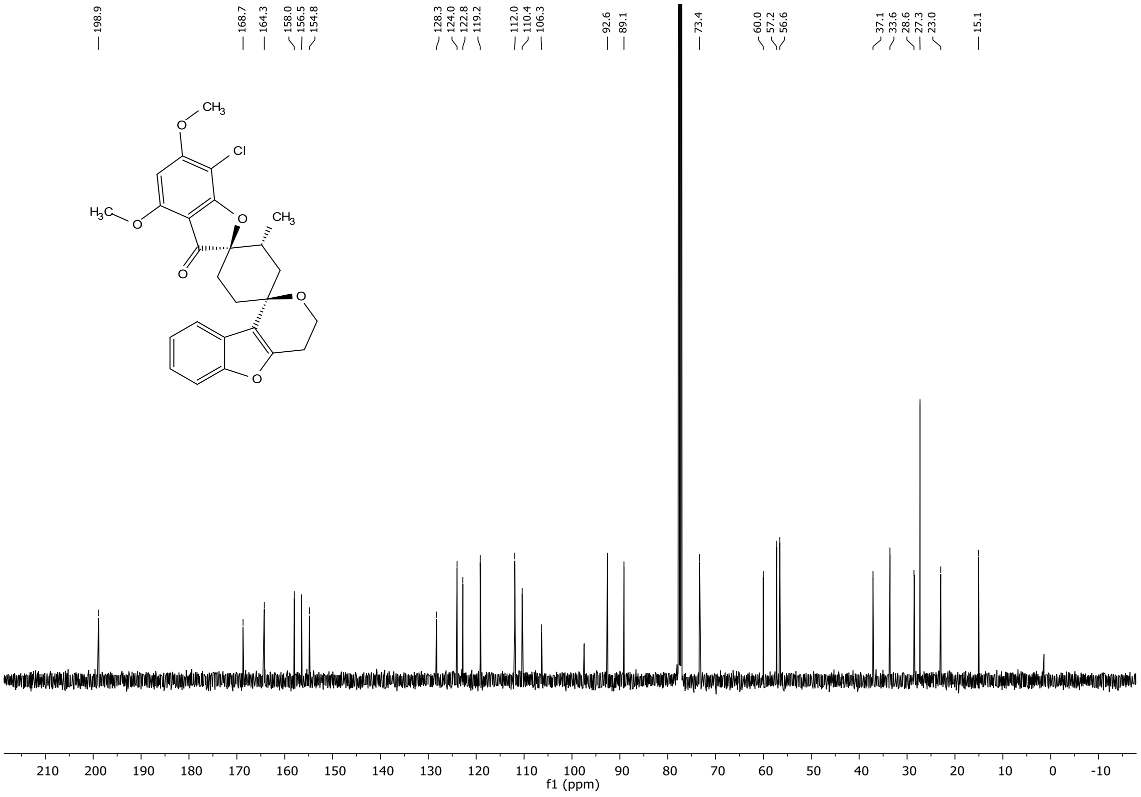

(2R,2'R,4'S)-7-chloro-4,6-dimethoxy-2'-methyl-3'',4''-dihydro-3H-dispiro[benzofuran-2,1'-cyclohexane-4',1''-benzo[4,5]thieno[2,3-c]pyran]-3-one **9**

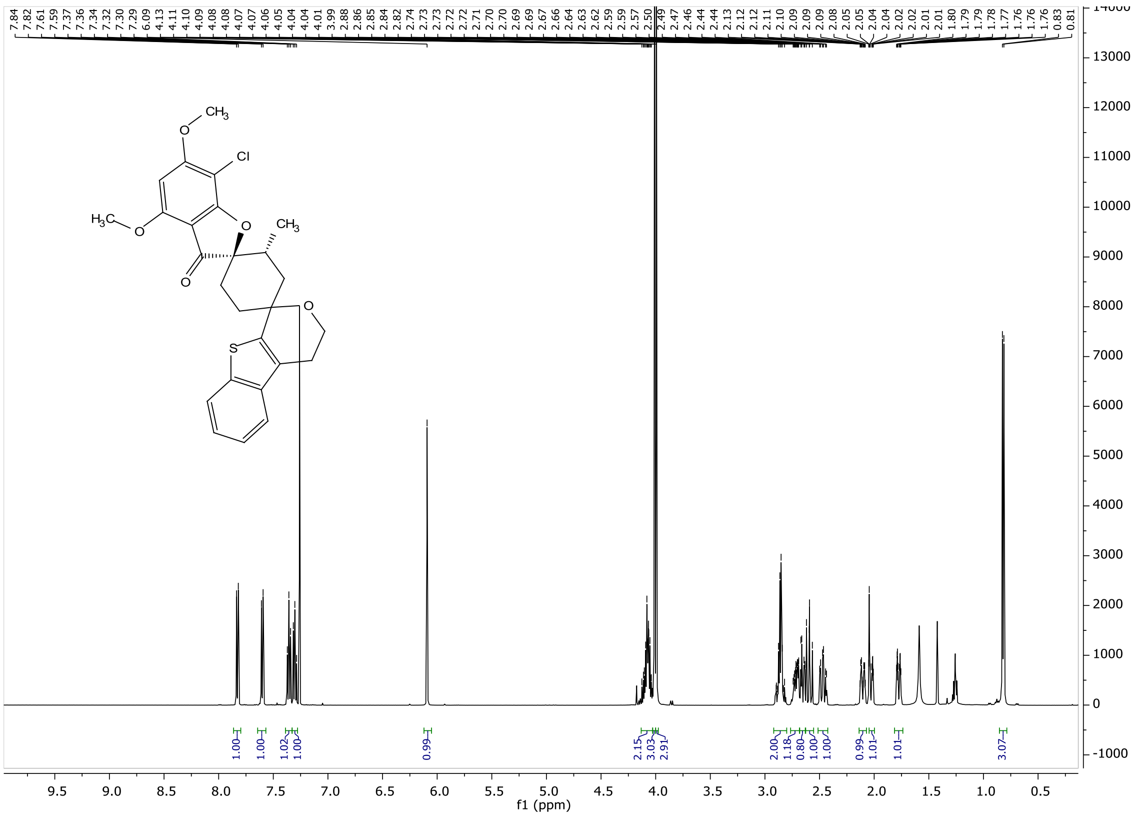

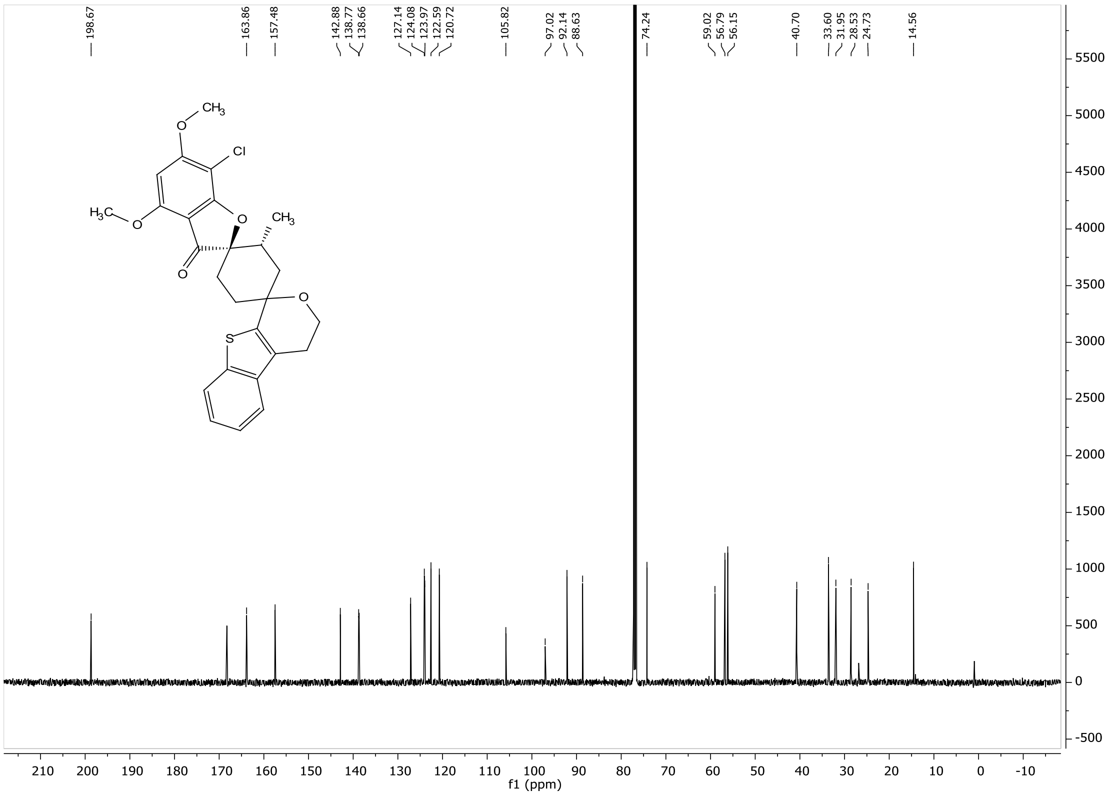

(2R,2'R,4'S)-7-chloro-4,6-dimethoxy-2'-methyl-4'',9''-dihydro-3H,3''H-dispiro[benzofuran-2,1'-cyclohexane-4',1''-pyrano[3,4-b]indol]-3-one **10**

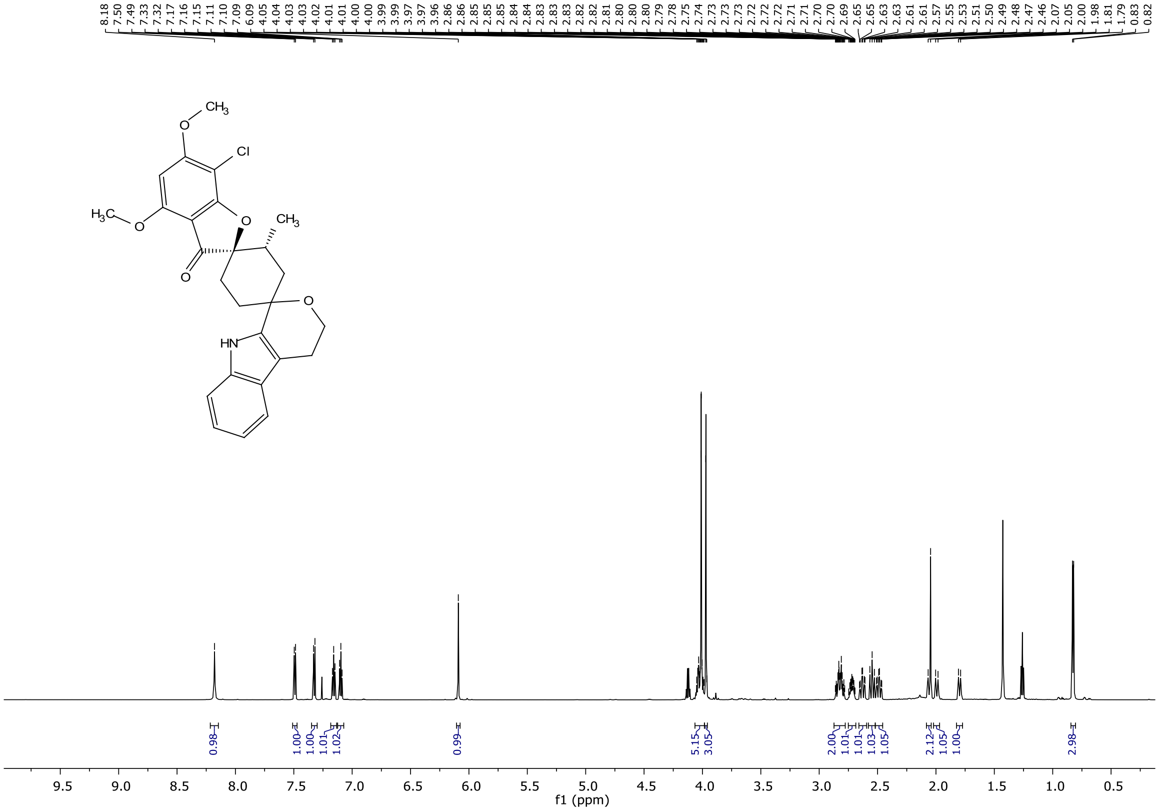

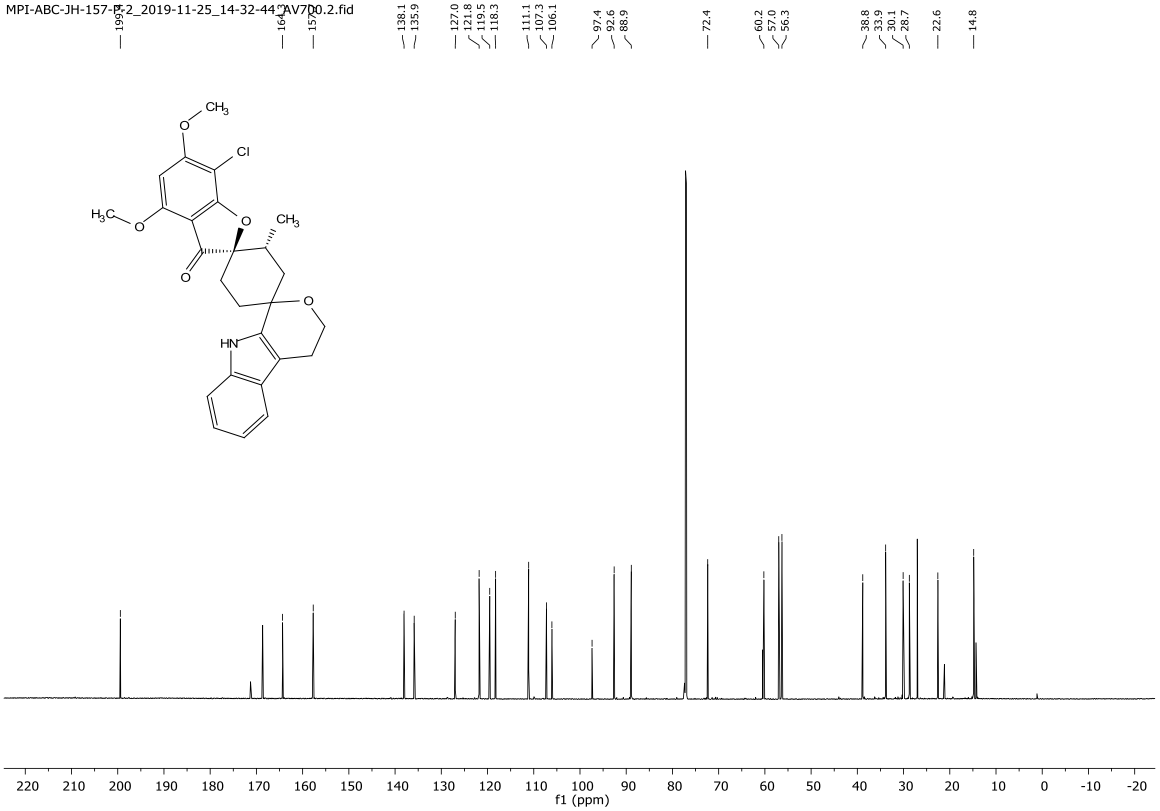

(2R,2'R,4'S)-7-chloro-4,6-dimethoxy-2',10''-dimethyl-3'',4''-dihydro-3H-dispiro[benzofuran-2,1'-cyclohexane-4',1''-[1,4]oxazino[4,3-a]indol]-3-one **11**

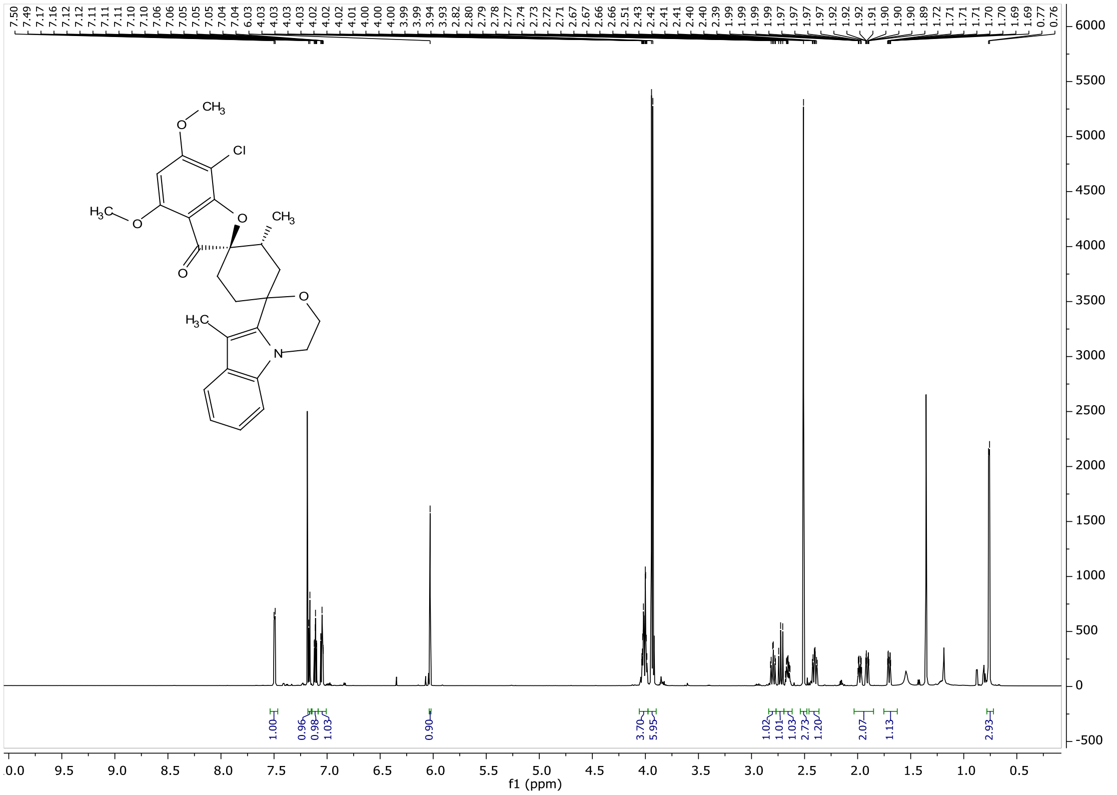

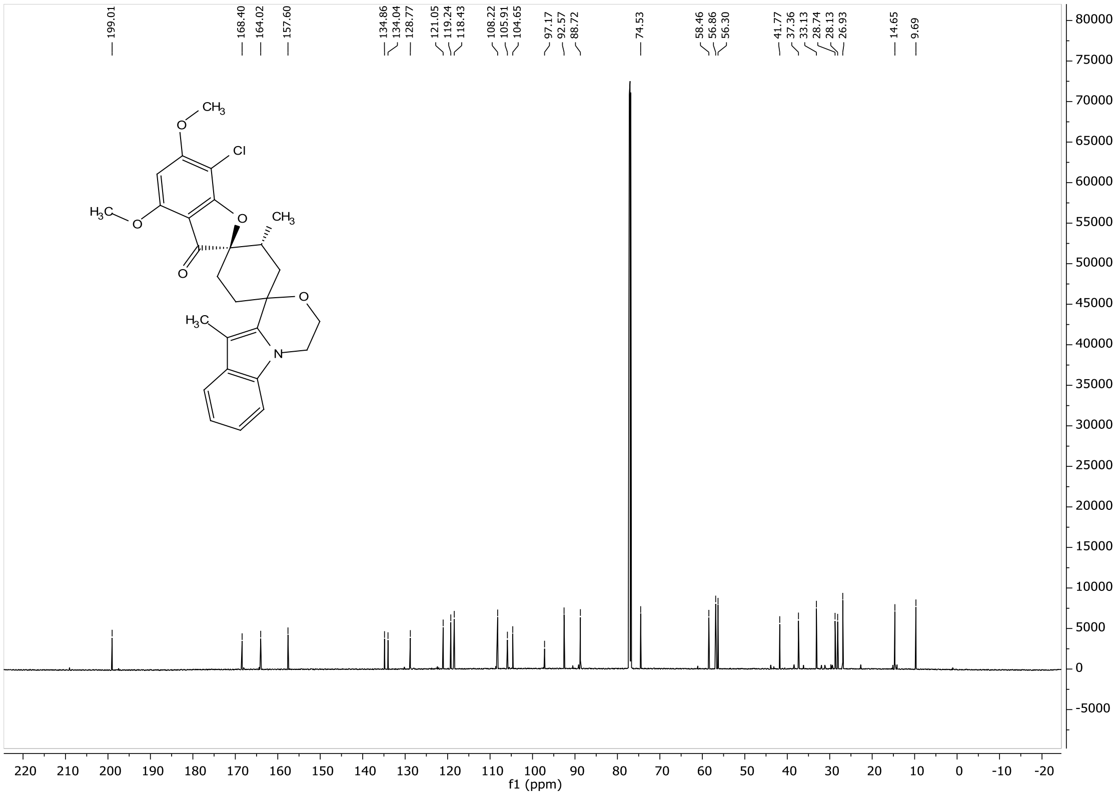

(2R,2'R,4'S)-7-chloro-4,6-dimethoxy-2'-methyl-6'',7''-dihydro-3H-dispiro[benzofuran-2,1'-cyclohexane-4',4''-thieno[3,2-c]pyran]-3-one **12**

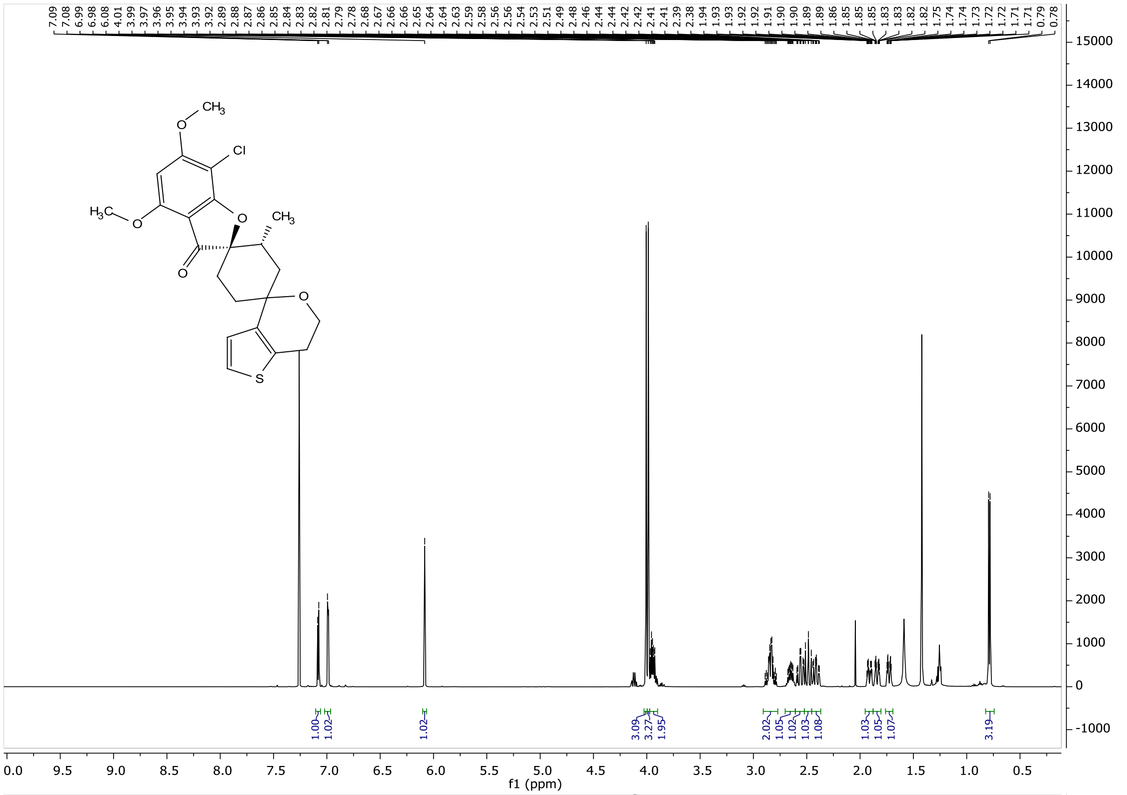

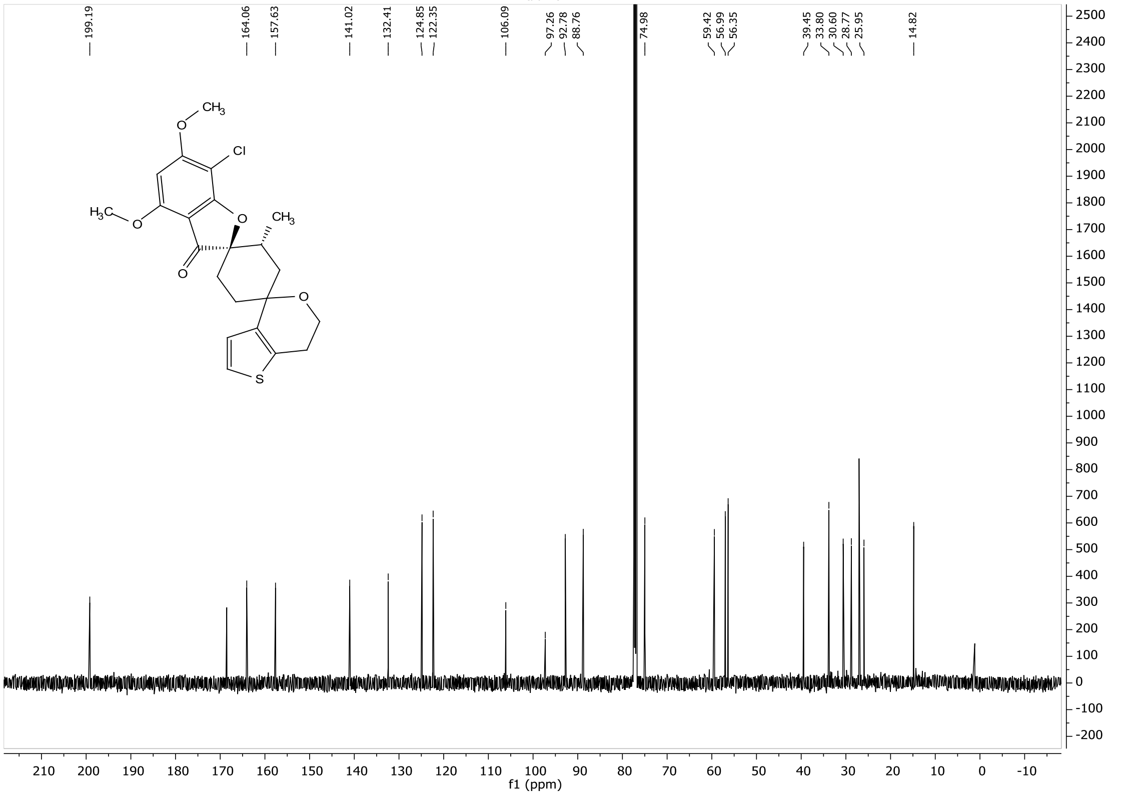

(2R,2'R,4'S)-7-chloro-4,6-dimethoxy-2'-methyl-4'',5''-dihydro-3H-dispiro[benzofuran-2,1'-cyclohexane-4',7''-thieno[2,3-c]pyran]-3-one **13**

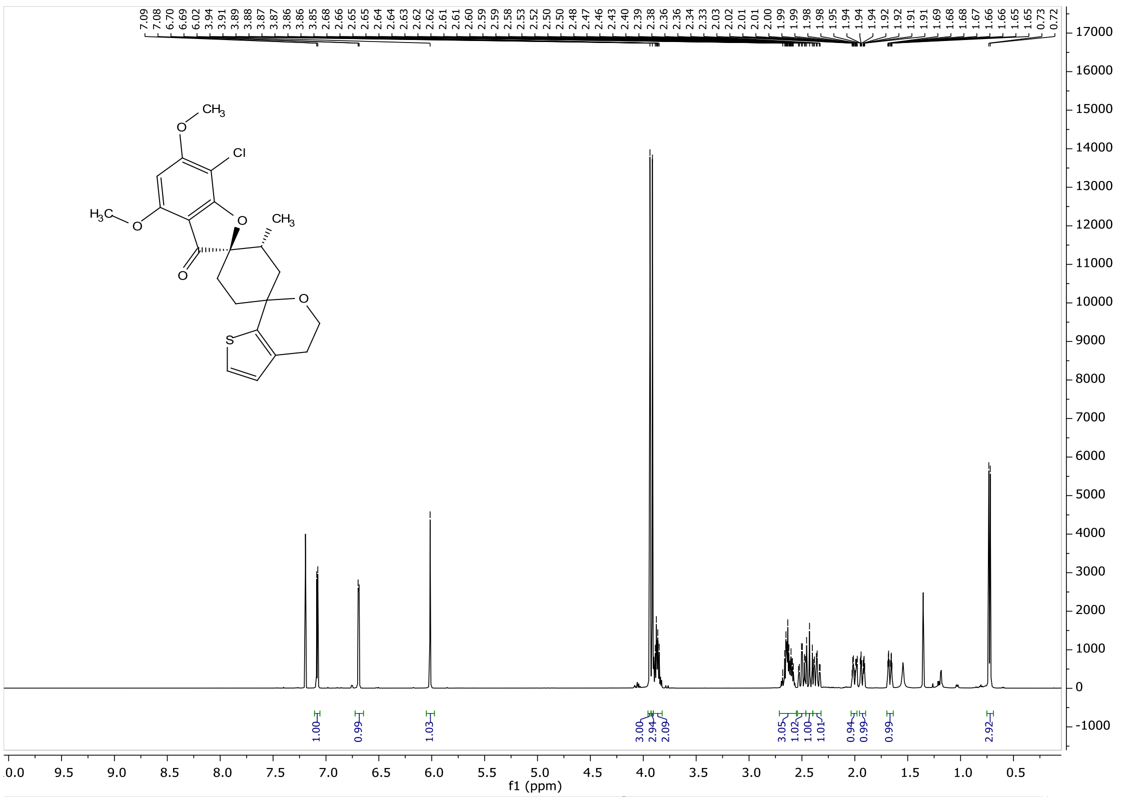

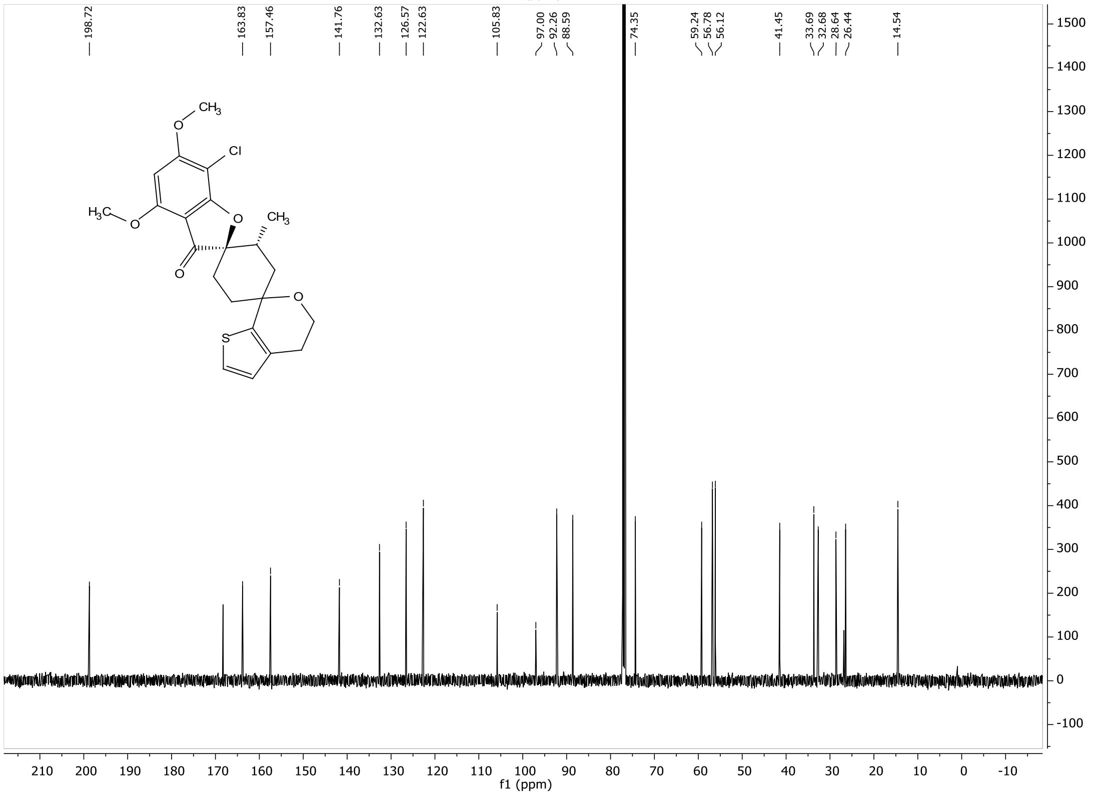

(2R,2'R)-7-chloro-4,6-dimethoxy-2'-methyl-7'',8''-dihydro-3H-dispiro[benzofuran-2,1'-cyclohexane-4',5''-[1,3]dioxolo[4,5-g]isochromen]-3-one **14**

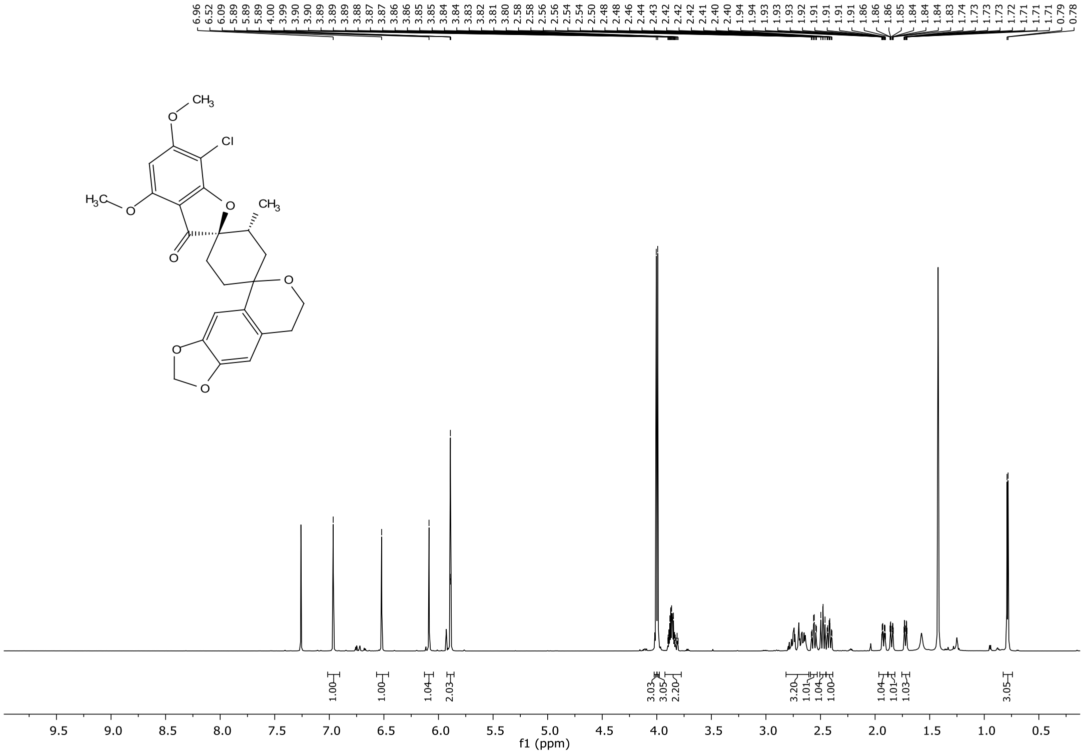

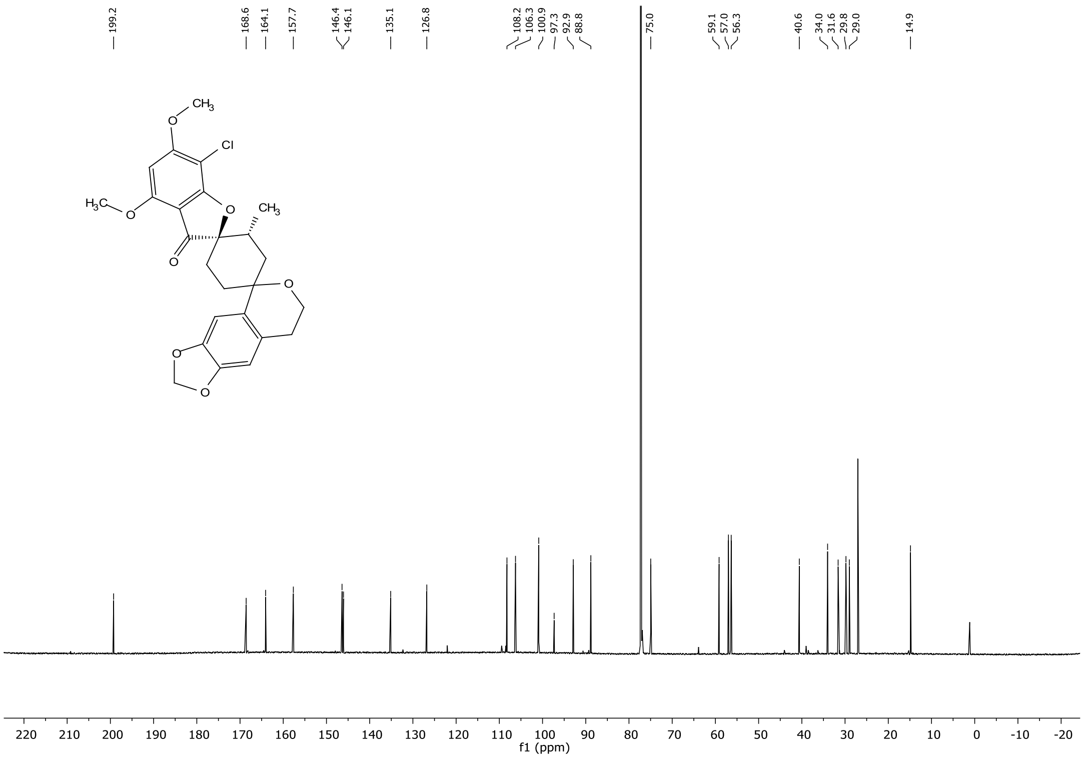

Supplement: Supplementary file 1 — Supporting Information [file ADVS-8-2102042-s001.pdf]
